# The Holo-Transcriptome of the Zoantharian *Protopalythoa variabilis* (Cnidaria: Anthozoa): A Plentiful Source of Enzymes for Potential Application in Green Chemistry, Industrial and Pharmaceutical Biotechnology

**DOI:** 10.3390/md16060207

**Published:** 2018-06-13

**Authors:** Jean-Étienne R. L. Morlighem, Chen Huang, Qiwen Liao, Paula Braga Gomes, Carlos Daniel Pérez, Álvaro Rossan de Brandão Prieto-da-Silva, Simon Ming-Yuen Lee, Gandhi Rádis-Baptista

**Affiliations:** 1 Northeast Biotechnology Network (RENORBIO), Post-Graduation Program in Biotechnology, Federal University of Ceará, Fortaleza 60440-900, Brazil; jean.etienne.brasil@gmail.com; 2 Laboratory of Biochemistry and Biotechnology, Institute for Marine Sciences, Federal University of Ceará, Fortaleza 60165-081, Brazil; 3 State Key Laboratory of Quality Research in Chinese Medicine and Institute of Chinese Medical Sciences, University of Macau, Macau 519020, China; huangchen11@yahoo.com (C.H.); liaoqw2007@126.com (Q.L.); simonlee@umac.mo (S.M.-Y.L.); 4 Department of Biology, Federal Rural University of Pernambuco, Recife 52171-900, Brazil; pgomes@db.ufrpe.br; 5 Academic Center in Vitória, Federal University of Pernambuco, Vitória de Santo Antão 50670-901, Pernambuco, Brazil; 6 Laboratory of Genetics, Butantan Institute, São Paulo 05503-900, Brazil; alvaro.prieto@butantan.gov.br

**Keywords:** Zoanthidea, holo-transcriptome, cnidarian transcriptome, marine enzyme, marine biocatalyst, marine biotechnology, pharmaceutical biotechnology

## Abstract

Marine invertebrates, such as sponges, tunicates and cnidarians (zoantharians and scleractinian corals), form functional assemblages, known as holobionts, with numerous microbes. This type of species-specific symbiotic association can be a repository of myriad valuable low molecular weight organic compounds, bioactive peptides and enzymes. The zoantharian *Protopalythoa variabilis* (Cnidaria: Anthozoa) is one such example of a marine holobiont that inhabits the coastal reefs of the tropical Atlantic coast and is an interesting source of secondary metabolites and biologically active polypeptides. In the present study, we analyzed the entire holo-transcriptome of *P. variabilis*, looking for enzyme precursors expressed in the zoantharian-microbiota assemblage that are potentially useful as industrial biocatalysts and biopharmaceuticals. In addition to hundreds of predicted enzymes that fit into the classes of hydrolases, oxidoreductases and transferases that were found, novel enzyme precursors with multiple activities in single structures and enzymes with incomplete Enzyme Commission numbers were revealed. Our results indicated the predictive expression of thirteen multifunctional enzymes and 694 enzyme sequences with partially characterized activities, distributed in 23 sub-subclasses. These predicted enzyme structures and activities can prospectively be harnessed for applications in diverse areas of industrial and pharmaceutical biotechnology.

## 1. Introduction

The worldwide demand for useful enzymes is continuously increasing in various industries, including the pharmaceutical sector, green chemistry, fine chemicals and basic and applied biomedical research. The market for enzymes that are more catalytically efficient than currently used enzymes, more environmentally friendly, and have potential use as drugs in the pharmaceutical industry, is foreseen to reach over USD 17 billion, by 2024 [1]. Additionally, the global market for enzymes that have applications in molecular biology and analytical kits is estimated to reach approximately USD 12.5 billion by 2021 [2]. In green chemistry, biocatalysts are used in environmentally benign chemical synthesis, in particular, halogenation and transamination, and the future of enzyme-catalyzed reactions is foreseen to rely on the enantio (selective) synthesis and kinetic resolution of chemicals, preferentially from renewable sources [3]. The development of eco-friendly and greener biocatalyzed processes aims to achieve better environmental factor values, i.e., mass of waste per unit of product [4]. Therefore, enzymes with improved properties, including physical and chemical stability, high conversion rates, change of substrate specificity, stereoselectivity, independence of cofactors for activity, ability to produce new chemicals, possibility to assemble multi-enzyme complex, among others, are desirable. Enzymes with these attributes and properties can be prospected by screening microbiomes [5,6] and/or by in vitro mutagenesis followed by direct evolution [7,8].

With the advent of “omics” sciences, the search for novel enzymes has progressed primarily by means of high throughput metagenomics assays of uncultured environmental samples [9]. In addition, the untapped biodiversity of distinct marine environments has become a hot target source for a wide variety of bioactive compounds, particularly, enzymes, that are halotolerant, thermostable and adapted to a range of pressures and substrate selectivity [10]. Some recent examples of marine-derived enzymes include the new flavin-dependent halogenase from a marine sponge metagenome [11] and several new α-amylases isolated from a sea anemone microbial community [12]. In two recent articles, the potential of marine biomes, particularly microbiomes, was emphasized, especially with regard to marine enzymes [13,14]. The successful bioprospection of natural compounds from marine species has been reported primarily from invertebrates of the phyla Porifera and Cnidaria [15]. Most of these compounds, which were secondary metabolites that were made into chemotherapeutics or drug-leads, were originally isolated from symbiotic microorganisms, rather than their host, in the holobiont assemblage [16]. Sponges, cnidarians, tunicates and other marine invertebrates can harbor a great diversity of microbial symbionts [17,18,19]. Despite the microbial origin of many compounds derived from the holobiont assemblage, the own coral tissues comprise unique resources of diverse chemicals with distinct pharmacological activities, such as anti-inflammatory and anti-proliferative activities [20,21,22]. Therefore, prospecting biopharmaceuticals from unusual marine species, as sources of unique enzymes, focusing particularly on improved and novel biocatalysts, is also warranted.

Our local, potential of discovery, biodiverse sites, i.e., the Brazilian large marine ecosystems—namely, the Brazilian Shelves, which are rich in coral reefs and are marine biodiversity hotspots, have been largely under-studied, particularly concerning to the discovery of novel enzymes and even natural products [15,23]. Recently, we conducted whole RNA sequencing of the anthozoan *Protopalythoa variabilis* (Cnidaria: Anthozoa) and described a repertoire of bioactive peptides with cnidarian toxin features in its transcriptome [24]. *P. variabilis* is a zoantharian species that preferentially grows in the shallow, warm water of the Atlantic coast and is present in abundance along the coastal reefs of the Brazilian Northeast [25]. Colonies of *P. variabilis*, like other anthozoans, harbor symbiotic zooxanthellae and a consortium of other microorganisms [26,27,28]. In the present work, we investigated the expressed enzymatic content of the *P. variabilis* holo-transcriptome. This species of zoantharian belongs to the phylum Cnidaria, one of the first groups that diverged from the Bilateria and is positioned at the base of Metazoa. The holo-transcriptomic analysis of *P. variabilis* extends the prospection of marine organisms for biotechnological studies and biopharmaceutical applications, which are less numerous than those of terrestrial and microbial origin [29]. In contrast to other analytical strategies reported in most recent articles, we focused our investigation on the entire transcriptome of *P. variabilis*, from a holobiont perspective, searching for enzyme precursors expressed in the zoantharian-microbiota assemblage that resulted in the prediction of numerous enzyme sequences relevant to biotechnology and green chemistry. These putative enzymes include oxidoreductases, transferases, hydrolases, lyases, isomerases and ligases, which have potential applications in several industrial fields, such as the production of pharmaceutics and fine chemicals, bioconversion and biopolymers, and green chemistry, to mention a few. Moreover, as exemplified in the present work, a single zoantharian species can be viewed as a species-specific repository of a unique collection of marine enzymes.

## 2. Results and Discussion

### 2.1. Biodiversity in the P. variabilis Holobiont

**Figure 1:**
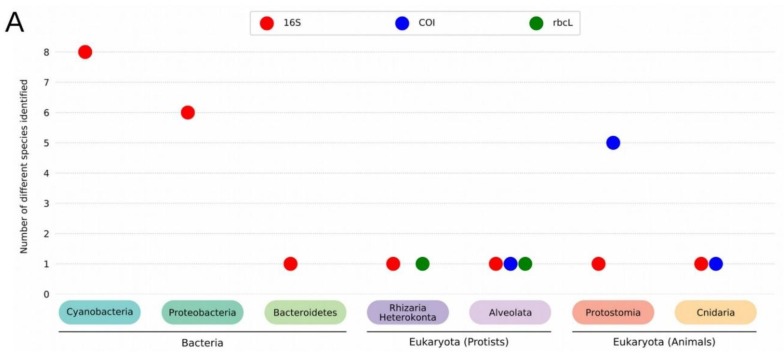

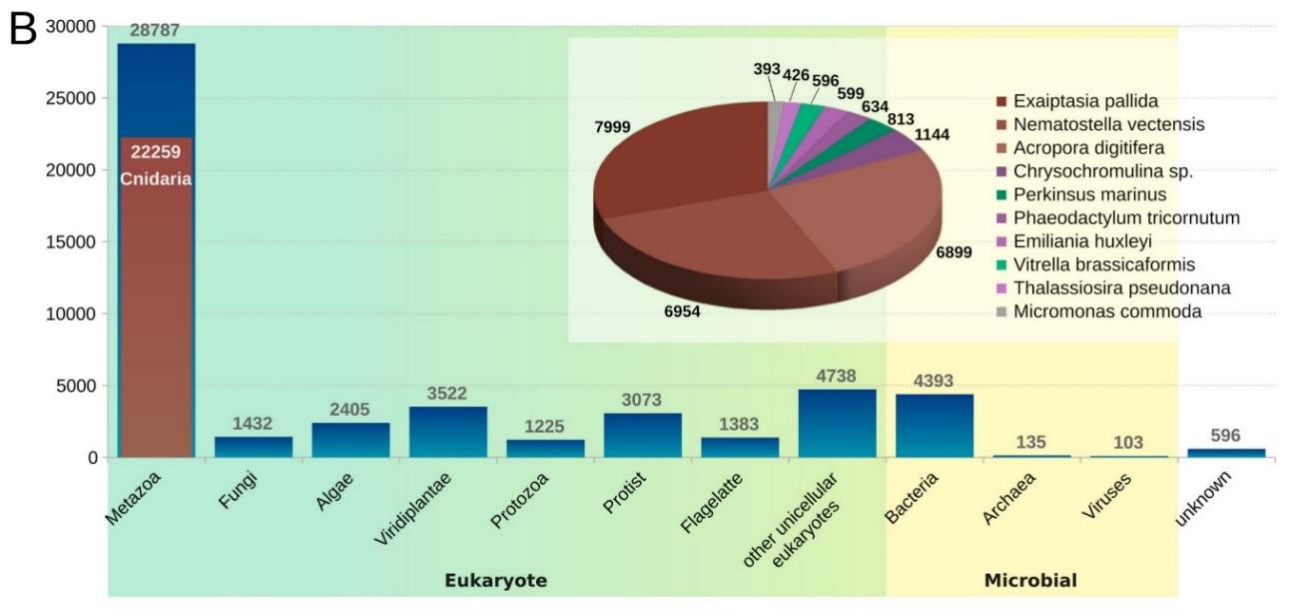
Biodiversity composition and taxonomic classification of unigenes from the *P. variabilis* holo-transcriptome. (**A**) Number of different species identified using the 16S rRNA, COI, and rbcL barcode sequences. (**B**) Taxonomic classification of unigenes from the *P. variabilis* holo-transcriptome after BLASTx analysis. Inserted Box: the distribution of top-hit species in the *P. variabilis* holobiont with the cnidarian species in dark red, the haptophytes and Stramenopiles in purple, the alveolates in green, and the green algae in gray.

Coral reefs are niches for different life forms, ranging from small fishes and crustaceans to associated and endosymbiotic microbial communities. Unraveling the biodiversity of a zoantharian holobiont is interesting not only from the ecological point of view, but also essential for the comprehension of the interconnected metabolic pathways, which ultimately depend on the symbiotic interactions and their enzymatic activities. To verify the overall diversity of life forms in the *P. variabilis* assemblage, we used three gene sequences commonly accepted for barcoding in species identification: the mitochondrial 16S rRNA gene for the taxonomic identification of bacteria and archaea, the mitochondrial cytochrome C oxidase subunit I (COI) gene for animals, algae, and dinoflagellates, and RuBisCo (rbcL) gene for plant and microalgae. The results of the species identification in the holobiont are shown in Figure 1A and the species are listed in Supplementary Table S1. In addition to the presence of the most common dinoflagellate algae of the genus *Symbiodinium*, involved in mutualistic symbiosis with cnidarians, the largest majority of the holobiont community identified is composed of uncultured species of cyanobateria and proteobacteria. Interestingly, four COI sequences found in the *P. variabilis* holobiont transcriptome had their best hits against terrestrial flying insects (Endopterygota), seemingly suggesting the interaction of *P. variabilis* with a group of organisms lacking known genetic information that belong to the phylum Arthropoda.

### 2.2. Annotation and Functional Prediction

Approximately 40% of the unigenes (51,792 sequences) identified in this study matched to entries in at least one of the four public protein databases, corresponding to “predicted or “annotated” hits (89% of all contigs) and “hypothetical” or “putative hits” (11%).

Supplementary Figures S1 and S2 present the complete statistical analysis of the sequence annotations. Compared to other *de novo* transcriptome assembly studies of cnidarian species, the initial level of BLAST annotations observed here was in the expected range [30,31,32,33]. Based on the selected BLASTx matches for the annotations, the taxonomic distribution of species from which the predicted protein sequences originated is depicted in Figure 1B. As can be observed, the three most representative species belong to the phylum Cnidaria, class Anthozoa, subclass Hexacorallia (namely, *Exaiptasia pallida*, *Nematostella vectensis*, and *Acropora digitifera*), which together accounted for 21,852 matched contigs in the annotation process for the *P. variabilis* holobiont transcripts (~42% of all annotated transcripts).

#### 2.2.1. Functional Classification of *P. variabilis* Predicted Enzymes

After a GO-slim reduction, a total of 29,866 unigenes (~58% of all annotated transcripts) were classified within 35 different ontological categories (Supplementary Figure S3A). The functional annotation of the *P. variabilis* holo-transcriptome, which returned GO terms for almost 30,000 unigenes, was substantially higher than that observed in the majority of previous studies but collectively consistent regarding the gene distribution within the categories of cellular or metabolic processes and functions.

#### 2.2.2. Assignment of Metabolic Pathways of the *P. variabilis* Predicted Enzymes

The KEGG pathway mapping analysis of the annotated unigenes retrieved 135 pathway maps (Supplementary Figure S3B), which were similar to the GO annotations in the category of “Biological Process”. However, the coverage differed greatly (Supplementary Table S2). These maps are available in Supplementary File S1.

From these analyses, a relatively high number of contigs were determined to encode enzymes involved in the metabolism of terpenoids and polyketides (Supplementary Table S2). This finding is interesting from the viewpoint of drug discovery, since numerous important bioactive secondary metabolites used in therapy, such as taxol, steroids and macrolide antibiotics, prostaglandins and tetracyclines, are synthesized in these biosynthetic routes. Indeed, the C-15 epimer of prostaglandin A2 (PGA2) and related structures have been identified and isolated from the Caribbean coral *Plexaura homomalla,* which produces and accumulates such compound [34].

### 2.3. Biotechnologically Relevant Enzymes from the P. variabilis Holo-Transcriptome

The identification of industrially relevant enzyme biocatalysts from environmental samples has been concretized by means of metagenomic analysis [9,35,36], a combination of metagenomics and metaproteomics technologies [37], and the combined application of transcriptomics and proteomics [38]. We based our search on analyzing the *P. variabilis* holo-transcriptome, providing an insight into the unexplored enzymes and related biosynthesis pathways expressed in this zoantharian-microbiota assemblage. Among all predicted proteins, 771 predicted enzymes classes with complete Enzyme Commission (EC) numbers were identified from the *P. variabilis* holo-transcriptome, representing a cumulative number of 6571 unigenes, with 606 of the predicted enzymes classes (5385 unigenes) mapped to KEGG pathways (Supplementary Table S3). Breaking down the enzymes by classes, 22% of them were oxidoreductases—EC 1 (170 enzymes for 806 unigenes), 35% were transferases—EC 2 (266 enzymes for 1208 unigenes), 21% were hydrolases—EC 3 (162 enzymes for 3792 unigenes), 10% were lyases—EC 4 (78 enzymes for 280 unigenes), 5% were isomerases—EC 5 (38 enzymes for 247 unigenes)), and 7% were ligases—EC 6 (57 enzymes for 238 unigenes).

Based on these results, we focused our study on enzymes with recognized relevance in chemical and pharmaceutical industries, as well as for green chemistry. For instance, transaminases (or aminotransferases) are stereoselective, cofactor-recycling enzymes that catalyze the transfer or exchange of an amino group from an amino-containing substrate to an acceptor molecule, resulting in the synthesis of chiral amino acids and amines. According to Supplementary Table S3, seven contigs encoding transaminases retrieved from the *P. variabilis* holo-transcriptome mapped mainly to antibiotic and amino acid biosynthesis pathways. The search for and development of optimized and high-performance transaminases that exhibit catalytic polyvalence (activity with a wide range of substrates), reactional stability and the possibility to form multi-enzyme complexes, are in demand for applications in green organic chemistry and the production of fine chemicals, food additives and pharmaceuticals [39]. In the following sections, additional selected examples of predicted enzymatic activities from the *P. variabilis* holobiont are presented, classified according to the major commercial and industrial application and discussed.

#### 2.3.1. Relevance in the Treatment of Rare Diseases and Other Biomedical and Clinical Applications

Several classes of hydrolases, such as galactosidases and proteases, are used in therapy and the preparation of biopharmaceuticals. For instance, fibrinolytic enzymes are used clinically as thrombolytic agents to treat myocardial infarction, asparaginase and arginine deaminase are used to treat leukemia and solid tumors, and α- and β-galactosidases are used to treat lysosomal storage disease [40]. Moreover, naïve or structure-guided directly evolved glycohydrolases have been tested in trials to remove sugar residues from the surface of erythrocytes to obtain universal blood [41]. Supplementary Table S4 presents, a list of predicted *P. variabilis* enzymes for which counterparts are used in the treatment of rare metabolic diseases, cancer, and for experimental blood production and organ transplantation. Several putative enzymes in the *P. variabilis* holo-transcriptome are homologous to therapeutic enzymes currently used to treat lysosomal storage disorders (LSDs). LSDs encompass a large number of metabolic diseases, primarily characterized by a lack of hydrolases and defects in the degradation of glycoproteins, glycolipid, glycosaminoglycan and glycogen [42]. Importantly, replacement therapy with human recombinant enzymes has been proven to be effective treatment in clinical and pre-clinical settings [40,42,43].

In the fields of green chemistry, industrial and pharmaceutical biotechnology, glucosidases have been used to prepare glucopolymers of polyvinyl saccharides, such as poly(glucosyl)-acrylates, which function as drug carrier systems and non-ionic polymeric surfactants. For example, Kloosterman and colleagues (2014) [44] utilized β-glucosidase to synthesize the monomers 4-(β-glucosyloxy)-butyl acrylate, 2-(β-glucosyloxy)-ethyl acrylate and methacrylate, as a means to avoid multiple reaction steps, thereby precluding the formation of undesirable isomers. β-glucosidases were also predicted from the *P. variabilis* holo-transcriptome (Supplementary Table S4).

Two important therapeutic enzymes used in cancer therapy are l-asparaginase and arginine deaminase, for which putative homologues were also identified in this study (Supplementary Table S4). l-asparaginase catalyzes the conversion of l-asparagine into l-aspartate, preventing cancer cell survival in patients with lymphoblastic leukemia, while arginine deaminase catalytically removes, by deamination, excess adenosine molecules in the circulation of patients with cancer, thereby reducing the toxicity to the immune system caused by elevated adenosine levels [45]. In the preparation of universal O-type blood, the selective removal of α-GalNAc and α-Gal residues of the A and B oligosaccharide antigens on the surface of red blood cells can be achieved by α-galactosidases and α-*N*-acetylgalactosaminidases, respectively [46]. Eleven sequences from the *P. variabilis* holo-transcriptome mapped to a putative β-*N*-acetylhexosaminidase (EC: 3.2.1.52). One of the eleven sequences is closely related to the clade Cnidaria, whereas another belongs to the protist-algae group, and three others are in the archaea-bacteria clade. The last one, Unigene28224, fits in between the protist-algae and archaea-bacteria groups (Supplementary Figure S4A). Despite the divergence of the sequences, the proton donor glutamic acid residues in the catalytic site were found to be conserved across all *P. variabilis* sequences (Supplementary Figure S4B).

Many enzymes and therapeutic proteins used in clinical and experimental clinical trials exist in PEGylated forms, in which serum stability and half-life are usually increased, while the immunological response is decreased. The PEGylation of proteins can potentially be achieved by biocatalysis using transglutaminases, which carry out an acyl transfer reaction [47]. As noted on Supplementary Table S4, a transglutaminase (EC 2.3.2.13) was found that may be used to catalyze the covalent transfer of the PEG moiety to therapeutic enzymes and proteins. Transglutaminases are also useful in other biotech industries, such as food processing, biopolymer production and leather and wool treatment [48].

#### 2.3.2. Relevance in Colorant, Aromas, Flavor, Fragrance, Cosmetic, and Hygienic Industries

Another group of polymer-degrading hydrolases, for which homologues are used in the fine chemicals industry, comprises glycosidases, alpha-amylase, polygalacturonase, beta-glucosidase, 1,4-alpha-glucosidase and cellulase (Supplementary Table S5). Moreover, in the industry of fine chemicals, oxidoreductases are widely used biocatalysts, and oxygenases (mono- and dioxygenases and peroxidases) are important enzymes for the modification of terpenoids. In corals, a diverse array of diterpenoids has been reported [49], with activities including anti-inflammatory, antifouling and antifeedant, anti-infective (antimicrobial, antiviral, anti-parasite), anticancer and cytotoxic effects. Thus, different species of marine cnidarian holobionts appear to be interesting sources not only for terpenoids themselves but also for enzymes involved in their biosynthesis. The holo-transcriptome of *P. variabilis* has revealed some of these predicted enzyme sequences.

#### 2.3.3. Relevance in Agrochemical, Food and Feed Industries

In the *P. variabilis* holo-transcriptome, many predicted enzymatic activities were also identified that may be relevant to the food industry (Supplementary Table S6). Hydrolases and oxidoreductases have emerged as preferred biocatalysts on an industrial scale for the generation of chirality and enantio (selective) kinetic resolutions of chemicals, especially from renewable sources [50].

From an ecological viewpoint, the expression of chitinases and enzymes related to chitin degradation, including chitodextrinase, in the *P. variabilis* holobiont is suggestive of a complimentary mechanism of self-defense in zoantharians, equipping the organisms to cope with disease-causing agents. Chitinases have known anti-fungal [51] and algicidal properties, contributing to nutrient cycling in marine environments [49]. In pharmaceutical biotechnology, chitinases are useful for preparing chitosan composites for numerous applications, ranging from biosensors, tissue engineering and drug delivery systems to nanoarrays and lab-on-chip devices [52]. Moreover, the coupled reactions of *N*-acetylhexosaminidase (a chitinase) and deacetylases can produce, a valuable nutraceutical supplement, d-Glucosamine, with high yields from polymeric chitin in a proof-of-concept environmentally friendly dual-catalysis process [53]. Interestingly, a predicted deacetylase (*N*-acetyl-d-glucosamine-6-phosphate deacetylase, EC 3.5.1.25) was found in the holo-transcriptome of *P. variabilis* (Supplementary Table S3).

Again, from an ecological viewpoint, the presence of transcriptional precursors encoding enzymes for the biosynthesis of herbicide intermediates, in the *P. variabilis* holo-transcriptome, can be seen as a possible way for this anthozoan species to restrain macroalgae overgrowth, as seen in corals [54]. Hence, considering the marine environment in which these enzymes presumably work, the possibility to obtain naturally evolved salt-tolerant biocatalysts for industrial bioprocesses is high.

#### 2.3.4. Relevance in Bioconversion and Biopolymer Synthesis

In the *P. variabilis* holo-transcriptome, some, but not all, industrially useful putative glycosidases that could potentially be applied to the processing of lignocellulose were found (Supplementary Table S7). Moreover, enzymatic activities involved in the production of precursors of biopolymers, such as recyclable bioplastic, were also observed. Examples of such enzymes include, 3-oxoacyl-ACP reductase (E.C. 1.1.1.100), acetyl-CoA C-acyltransferase (2.3.1.16) and enoyl-CoA hydratase (4.2.1.17), which catalyzes the biosynthesis of polyhydroxyalkanate (PHA) precursors. The predicted enzymes epoxide hydrolase (3.3.2.9), nitrile hydratase (4.2.1.84) and γ-glutamyltransferase (2.3.2.13) are important for bioconversion in the renewable energy industry and for the synthesis of fine chemicals, in green chemistry and bioremediation, as will be discussed later. Finally, the peroxidases identified in this zoantharian holo-transcriptome could be further evaluated for applications in the conversion and biosynthesis of phenol- and vinyl-containing polymers, accordingly to the current use of enzymes of these catalytic classes.

#### 2.3.5. Relevance in the Cleaning and Recovery Industries

The identification of enzymatic activities in a given microbiome is useful for estimating the capacity of the microbes to recover an environmental site. The disclosed enzymatic activities, serve as indicators of the bioremediation potential for a given set of contaminants, and they also indicate potential enzymatic catalysts that may be isolated for downstream processing. In this regard, among the enzymes listed in Supplementary Table S8, a very interesting enzyme is glutathione γ-glutamylcysteinyltransferase (E.C. 2.3.15.2), also known as phytochelatin synthase, which catalyzes the synthesis of phytochelatins. Phytochelatins are cysteine-rich peptides responsible for the chelation and sequestration of essential microelements (e.g., copper and zinc) and toxic heavy metals (e.g., cadmium, lead and mercury). Members of the phytochelatin family are biosynthesized from glutathione and are structurally characterized by *n* repetitions of the γ-GluCys dipeptide followed by a terminal Gly, i.e., (γ-GluCys)*n*-Gly [55]. The genes encoding enzymes for phytochelatin synthesis have a recognized wide phylogenetic distribution, indicating the importance of coping with metal and metalloid (arsenic, selenium and silver) detoxification across species [55,56]. Thus, the expression of phytochelatin synthase in the *P. variabilis* holo-transcriptome could be related to detoxification of heavy metals and metal homeostasis in zoantharians. From the biotechnological point of view, immobilized phytochelatin synthase has been utilized to prepare highly stable cadmium-sulfur (CdS) nanocrystals of tunable sizes with optical and electrical properties [57]. Prepared in this way, nanocrystals are useful in the fabrication of semiconductor quantum dots (QDs) and for application as fluorescent tags in biological systems for molecular imaging. Another potential technological use for phytochelatin synthases is related to bioremediation; engineered bacteria have been designed that overexpress a mutated enzyme in combination with a cadmium protein transporter, resulting in Cd accumulation [58].

Last but not least, in this group of enzymes relevant to the recovery and processing industry, epoxide hydrolase and nitrile hydratase homologous were found. Epoxide hydrolases (EC 3.3.2.3) are cofactor-independent biocatalysts that convert epoxides to the corresponding diols, and epoxide hydrolases of microbial origin are useful for producing enantioselective intermediates with vicinal diols in the synthesis of fine chemicals and pharmaceuticals, such as (*S*)-Ibuprofen, (*R*)-Mevanolactone and (*R*)-Eliprodil [59,60]. Nitrile hydratases are Fe- or Co-type metalloenzymes that convert nitriles (organo-cyanides) into industrially valuable amides, such as acrylamides, from acrylonitriles. Tons of acrylamide are used as coagulators, soil conditioners and additives in the paper industry, as well as adhesives, paint components and agents for petroleum recovery [61,62]. Additionally, wild-type and engineered nitrile hydratases that act on myriad cyanide-containing substrates can be applied in bioremediation, wastewater treatment and even for the development of cyanide biosensors [63]. These examples provide a glimpse of the biotechnologically significant enzymes found in the holo-transcriptome of *P. variabilis* that may be used as biocatalysts with improved activities and selectivity over those currently in use. Moreover, one can speculate on the eco-physiological role of these enzymes in the cnidarian-holobiont assemblage.

#### 2.3.6. Relevance in Molecular Biology and Analytical Applications

In Supplementary Table S9, homologs of enzymes useful for manipulating nucleic acids found in the *P. variabilis* holo-transcriptome are listed. Among these, two enzymes—deoxyribonuclease I and *N*-acetylglucosaminidase—have also been used to treat cystic fibrosis and lysosomal storage disease, respectively.

In recombinant DNA technology, deoxyribonuclease (DNase) I catalyzes the hydrolysis of phosphodiester bonds and cleaves double-stranded (ds) and single-stranded (ss) DNA in a sequence-nonspecific manner. DNase I is used, for instance, to make “nick translations” and in DNase I footprinting—a technique used to study the interaction of ligands (proteins and drugs) with DNA [64]. Other predicted endonucleases found in the *P. variabilis* holo-transcriptome include DNase II and IV; DNase II is a lysosomal “acidic” DNase that preferentially cleaves ssDNA, whereas DNase IV (flap endonuclease-1) is a multifunctional enzyme that cleaves 5′-ssDNA flaps of DNA or RNA. DNase IV has been investigated as a marker of disease risk, since it is involved in DNA metabolism, genomic stability and apoptosis [65].

Several types of predicted ribonucleases were also found, such as ribonucleases (RNases) H and T (Supplementary Table S9). RNase H is a monomeric enzyme that degrades RNA in RNA:DNA heteroduplexes and is useful for the synthesis of complementary DNA (cDNA); RNase III works in multicomponent assemblies to bind and cleave dsRNAs, especially in the processing of dsRNA maturation and the decay of coding and noncoding RNAs, such as miRNAs and siRNAs [66].

DNA-dependent DNA polymerases catalyze the biosynthesis of polydeoxyribonucleotides; diverse applications for DNA polymerases exist, including nucleotide sequencing, in vitro synthesis of the second cDNA strand, DNA amplification and the preparation of DNA hybridization probes [67]. RNA polymerases (RNA-dependent DNA polymerases) are enzymes that transcribe gene sequences into the corresponding RNAs. RNA polymerases are used to prepare hybridization probes and in vitro-transcribed RNA to direct a high-level of expression of cloned genes, as well as to produce capture reagents for RNA-binding proteins and as antisense probes [68]. In the *P. variabilis* zoantharian holo-transcriptome, predicted and structurally conserved RNA-dependent RNA polymerase (reverse transcriptase) sequences were retrieved. Because reverse transcriptase catalyzes the synthesis of DNA from an RNA template, the uses for this type of enzyme include the preparation of cDNA and the molecular design of inhibitors of retrovirus replication [69]. DNA and RNA ligases catalyze the formation of 3′ → 5′ phosphodiester bonds in nucleic acid (DNA and RNA) molecules. These enzymes have a range of uses in recombinant DNA technology, from elongation or circularization of dsDNA, in the case of DNA ligases, to 3′-labeling of RNA, DNA 5′-tailing of DNA and production of elongated molecules, e.g., in cDNA cloning, in the case of RNA ligases [70]. Transcripts encoding both types of ligases were found to be expressed in the zoantharian holo-transcriptome in this study.

### 2.4. Prediction of Enzymes with Two or More Activities

In searching for different types of enhanced and unique marine biocatalysts, we wondered whether the *P. variabilis* holobiont transcriptome contains enzymes with multiple activities. A widely known example of a dual catalytic enzyme is RuBisCO (ribulose-1,5-bisphosphate carboxylase/oxygenase, EC 4.1.1.39), which catalyzes both CO_2_ fixation in the dark phase of photosynthesis and carbon oxidation in the process of photorespiration [71]. Another interestingly example of a dual catalytic enzyme from plants is the enzyme hydroxycinnamoyl-Coenzyme A:quinate hydroxycinnamoyl transferase (HQT, EC 2.3.1.99), which catalyzes the formation of the strong phenolic antioxidants, chlorogenic and dicaffeoylquinic acids, which are useful as phytonutrients in foods and as pharmaceuticals. It has been demonstrated that in addition to the transesterification of caffeoyl-CoA with quinic acid to produce chlorogenic acid, HQT can form dicaffeoylquinic acid via its chlorogenate:chlorogenate transferase activity [72]. In biotechnology, multi-enzyme systems have been designed with different assembly strategies to mimic natural enzyme complexes and pathways, with the aim of improving catalytic efficiency [73]. We devised an annotation iterative process that resulted in the prediction of 13 putative enzymes with two to three enzymatic activities (Table 1). Six of these enzymes have activities that are partially identified and are related to more than one metabolic pathway (CL12403.contig2, CL2444.contig1, unigene12818, unigene14615, unigene32504, as well as unigene33780), and another four enzymes with dual activities were positioned in a single metabolic pathway. Enzymes that were predicted to possess dual catalytic activity are also shown in Supplementary Figure S5; these were found as a result of our stringent analysis. Some putative dual-activity enzymes might have been missed; however, this strategy proved to be effective, as it included an initial convenient and rapid data mining and screening approach of bi-functional biocatalysts from this zoantharian holo-transcriptome.

### 2.5. Annotation of Novel Predicted Enzyme Sequences with Partial EC Number

**Figure 2:**
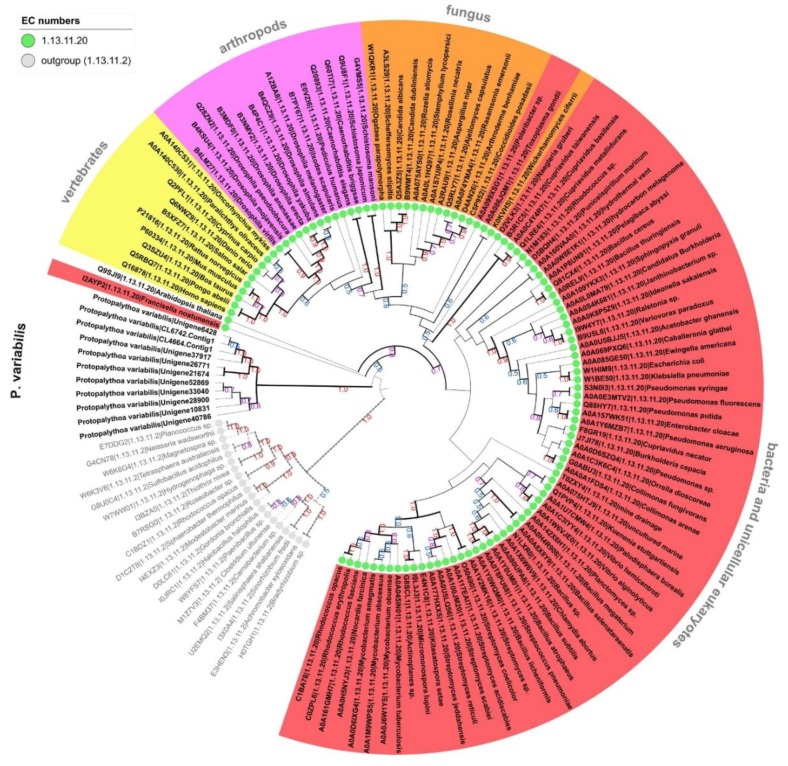
Cladogram depiction of the *P. variabilis* predicted sequences related to cysteine dioxygenase (EC:1.13.11.20) members. Tree based on the distance between the protein sequences of cysteine dioxygenase (EC:1.13.11.20) from 106 species and eleven *P. variabilis* predicted enzymes with an incompletely annotated EC:1.13.11 activity. Twenty catechol 2,3-dioxygenase (EC:1.13.11.2) sequences were used as outgroup. Only bootstrap values greater than 50% are shown at the branch points, in blue, purple, or red color for values comprised between 50–69%, 70–89%, and 90–100% respectively. Enzyme activities are indicated at the name base by circles colored as indicated in the legend.table.

**Figure 3:**
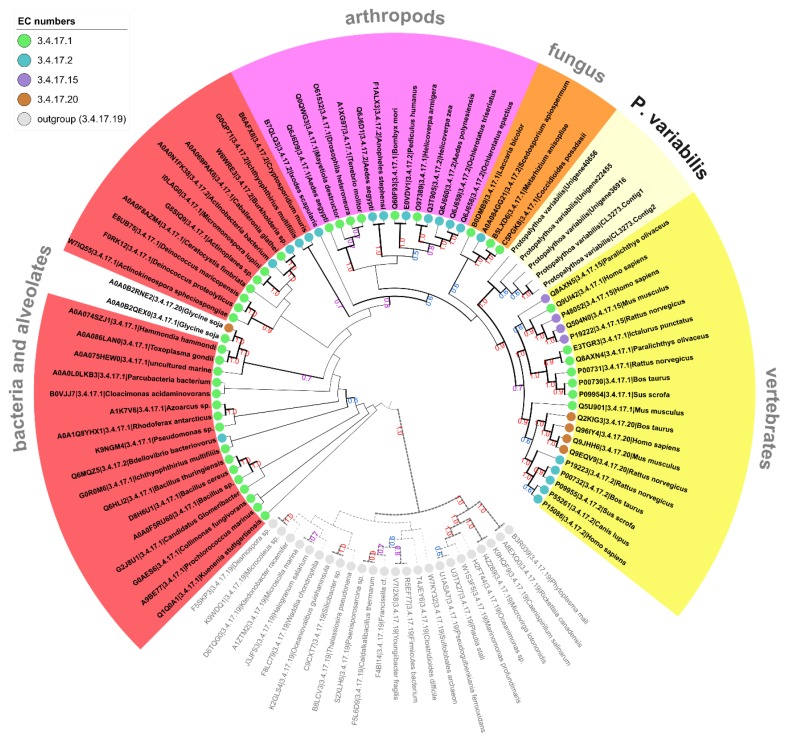
Cladogram depiction of the *P. variabilis* predicted sequences related to carboxypeptidase A, B, A2, and U (EC:3.4.17.1, 2, 15, and 20) members. Tree based on the distance between the protein sequences of carboxypeptidase A, B, A2, and U (EC:3.4.17.1, 2, 15, and 20) from 40, 19, 4, and 5 species respectively and five *P. variabilis* predicted enzymes with an incompletely annotated EC:3.4.17 activity. Sequences from 24 carboxypeptidase Taq (EC:3.4.17.19) sequences were used as outgroup. Tree legend same as in Figure 2.

In addition to the prediction of enzymes with activities that are fully characterized, groups of enzymes that are of general interest comprise expressed sequences that are completely new. These, as analyzed herein, could not be mapped to a specific and detailed catalytic reaction, i.e., they comprise enzymatic precursors with an EC number lacking the fourth categorization numbers. Indeed, several predicted enzymes were found with a sequence similarity that were reasonably close to be classified within a known sub-subclass (EC with at least three numbers), but, still, distinct enough to be completely mapped in given reactional group of characterized enzymes. Predicted precursors that fit in this category could hypothetically point to isozymes with already described reactions but working on different substrates, with distinct kinetic parameters, in distinct catalytic conditions or, even, comprising a totally novel catalytic reaction. Based on these facts, from the *P. variabilis* holo-transcriptome, additional 694 predicted enzyme sequences were found with incomplete EC numbers (unknown fourth serial digit), distributed into 23 sub-subclasses (Table 2), with a large majority related to the class of hydrolases. In this study, based on these preceding findings, we focused our further analysis on two sub-subclasses that *P. variabilis* sequences are grouped in distinct new clades, representing new structures, namely, cysteine dioxygenases (EC:1.13.11.20) and carboxypeptidases A, B, A2, and U (ECs:3.4.17.1, 3.4.17.2, 3.4.17.15, 3.4.17.20). I Initially, all predicted *P. variabilis* sequences mapped to a sub-subclass were evaluated by phylogenetic inference with corresponding counterparts representing each species for each enzyme in this sub-subclass. It was found that eleven *P. variabilis* sequences were related to the cysteine dioxygenases while forming an out-clade, and five sequences with the carboxypeptidases A, B, A2, and U forming a distinct clade all together. Afterwards, a second tree was inferred to confirm that, even if they are related, the *P. variabilis* sequences form a distinct clade, showing their uniqueness (Figure 2 and Figure 3). Cysteine dioxygenase is a key enzyme in the synthesis of taurine, an important compound, product of cysteine metabolism, that is used in functional foods, as well as in pharmaceutical and cosmetic industries. Several patents are granted to the production of taurine by fermentation methods with transgenic microorganisms. Carboxypeptidases are generally applied either in research or in the pharmaceutical industries, but also found some application in food industries, as exemplified by the use of the carboxylase A in baking industry [74].

## 3. Materials and Methods

### 3.1. Biological Sample

Specimens of *Protopalythoa variabilis* (Duerden, 1898) were collected in coastal reefs of Porto de Galinhas, Pernambuco, Brazil (8°30′20″ S, 35°00′34″ W) during low tide. A voucher specimen (GPA 181) was identified by us and was kept at the cnidarian collection of the Anthozoan Research Group (GPA) at the Academic Center of Vitória, Federal University of Pernambuco (Brazil). This species has been mentioned by some authors as *Palythoa variabilis*, in reason of a proposed synonymy for the genera *Palythoa* and *Protopalythoa* [75]. However, the issue of distinctive genera is not completely solved yet (see, for example, [76]), despite new molecular phylogenetic approach, based on the universal target-enrichment baits, has been recently developed to help resolve long-standing controversial relationships in the class Anthozoa [77]. Thus, until an extensive revision of the group is not definitively resolved with morphological and molecular data precisely combined, with inclusion of species of both genera, the binomial nomenclature *Protopalythoa variabilis* is used herein, as for decades.

### 3.2. RNA Library Construction and Origin of Zoantharian RNA Sequences

The RNA isolation, library preparation and the transcriptome assembly of the *P. variabilis* holobiont were performed as described in one of our previous articles [24]. RNA sequencing was performed using the Illumina HiSeq 2500 platform. Reads were cleaned up before the de novo transcriptome assembly using the Trinity method for transcriptome reconstruction [78]. This Transcriptome Shotgun Assembly (TSA) project was deposited in DDBJ/EMBL/GenBank under the accession GCVI00000000, associated with the BioProject PRJNA279783 and biosample SAMN03450566. The statistics of the RNA sequencing and contig assembling are summarized in the Supplementary Table S1.

### 3.3. Assessment of the Biodiversity Composition of the P. variabilis Holobiont

*P. variabilis* transcripts with a high similarity to 16S rRNA, COI, and rbcL sequences were identified using BLASTn with an E-value limit of 1E-40. The closest related species were characterized by homology search of these transcripts against the NCBI nr database. Only the first hits in concordance with the selected gene markers aforementioned were retained for species composition identification in the holobiont assemblage.

### 3.4. Sequence Annotations for Enzyme Precursors in the P. variabilis Holobiont

The unigenes from the *P. variabilis* transcriptome were investigated for structural enzyme homology using BLASTx (BLAST+ suite, version 2.5.0) [79] with a fixed E-value of 1E-5 against four public protein databases: the NCBI non-redundant (nr) protein database (accessed from October to November 2016), the Clusters of Orthologous Groups (COG) database, version 2003–2014, the UniProtKB/Swiss-Prot database, downloaded in October 2016), and the EuKaryotic Orthologous Groups (KOG) database, version 2003. Basic statistics of the sequence annotations are described in Supplementary Figures S1 and S2. Species information of the selected annotations was extracted from the BLAST output files to discern the taxonomic distribution.

### 3.5. Gene Ontology, Enzyme Codes and KEGG Pathway Assignments

The Blast2GO software, version 4.0.2 [80], was used for the subsequent steps under default parameters to carry out the InterProScan protein domain analysis, followed by the Gene Ontology (GO) annotation. The annotations were then subjected to a generic GO-slim reduction, prior to the mapping of the Enzyme Commission codes (EC) and KEGG pathways. The GO annotations chart was plotted using WEGO [81], while further KEGG pathways information was retrieved through the KEGG BRITE hierarchies site [82].

### 3.6. Sequence Alignment and Phylogenetic Inference

Multiple sequence alignments of predicted enzyme sequences were performed using MUSCLE v3.8.31 [83] or Kalign v2.9.0b2 [84], depending on whether the enzyme domain/motif was a single structural unit or multiple-domain repeats, respectively. The phylogenetic tree was inferred using MEGA 7.0.26 [85] based on the LG+G+I model, with a bootstrap test of 500 replicates and edited using FigTree v 1.4.3. The amino acid sequence identities and similarities were determined using Jalview 2.9.0b2 [86].

### 3.7. Prediction of Enzymes with Two or More Activities

An alternative annotation strategy was used to identify potential enzymes with two or more enzymatic activities. To favor the multiple domains (hits) discovery, we first performed a BLASTx search against the UniProtKB/Swiss-Prot database, keeping an E-value of 1E-5 with the additional “culling_limit” option set to “1”. The selected set was reduced to only sequences with at least two non-overlapping hits on the same frame. During a second round of selection, only sequences with predicted protein products containing regions found by BLAST were kept. Finally, an InterProScan protein domain analysis [87] was performed to map each sequence to its GO annotation and its associated EC codes and KEGG pathways using the external EC2GO [88] and KEGG2GO mapping databases [89,90]. Only sequences with two or more ECs were kept, and data on the enzyme’s substrate(s) and product(s) were retrieved from KEGG Enzyme [82].

### 3.8. Analysis of Predicted Enzyme with Partial EC Number

Results of the annotation generated during the Blast2GO analysis were used to extract a list of the transcripts with a partial EC number. For each of these sequences, the corresponding protein sequence is predicted and subsequently submitted to InterProScan 5 via RESTful service 80 to validate the presence of a catalytic domain corresponding to the partial EC. These steps were automatized using a python script, depending on the external EC2GO mapping database [88]. To perform the observed distance analysis between the predicted *P. variabilis* and the known enzyme sequences, the available collections of sequences corresponding to each EC of interest were retrieved from the BRENDA website (www.brenda-enzymes.org, release 2018.1) [91] and sequences with a length shorter or higher to two population standard deviation were removed. The multiple sequence alignment was done using Kalign v2.04 77, and the analysis was computed with MEGA 7.0.26 78 using the Neighbor-Joining method and the p-distance method, removing missing ambiguous positions for each sequence pair, and with a bootstrap test of 500 replicates. The cladograms were edited in the Interactive Tree Of Life website [92].

## 4. Conclusions

Marine invertebrates with associated microbiota form complex holobiont assemblages, which are attractive sources of biologically active organic compounds and (poly-)peptides, including enzymes. Marine enzymes have a high potential to be applied in green organic synthesis and in pharmaceutical and industrial biotechnology. The search for improved biocatalysts can be carried out using different strategies, such as screening a huge number of environmental samples, pursuing enzyme engineering, mining genomic and proteomic data, or a combination of more than one approach. The process of data mining transcriptomes has some advantages over genomic analysis; the most obvious advantage is that only enzymes that are expressed in a given environmental context are retrieved, including enzymes not completely characterized and with unknow enzyme-substrate specificity. This is particularly advantageous in the case of the marine assemblages of microbionts that form species-specific holobionts, from which the purification of enzymes with a high yield may be a concern. Thus, once identified, the cloning and the recombinant production of desirable marine biocatalysts can be structure-guided and based on the nature of expressed transcripts.

According to data reported in this work, the zoantharian *P. variabilis* expresses a variety of putative enzymes that could potentially be converted into biotechnologically useful biocatalysts and biopharmaceuticals. This holo-transcriptomic data demonstrates that a single holobiont assemblage comprises a unique repository of relevant biotechnological enzymes. Finally, the integrative analyses of this holo-transcriptome point to a valuable marine resource for the discovery of improved enzymes with applications in green chemistry, industrial and pharmaceutical biotechnology.

## Figures and Tables

**Table 1:** List of predicted multi-functional enzymes from the *P. variabilis* holo-transcriptome with dual catalytic activities.

Unigene	ECs	Activities	Substrate	Product
CL12403.Contig1	1.5.1.20	methylenetetrahydrofolate reductase (NAD(P)H)	5-methyltetrahydrofolate	5,10-methylenetetrahydrofolate
	2.1.2.1	glycine hydroxymethyltransferase	5,10-methylenetetrahydrofolate	tetrahydrofolate
CL12403.Contig2	1.5.1.20	methylenetetrahydrofolate reductase (NAD(P)H)	5-methyltetrahydrofolate	5,10-methylenetetrahydrofolate
	2	transferase	?	?
CL2444.Contig1	4.2.1.11	phosphopyruvate hydratase	2-phospho-d-glycerate	phosphoenolpyruvate
	5.3.1.1	triose-phosphate isomerase	d-glyceraldehyde 3-phosphate	glycerone phosphate
Unigene12818	1.3.99.1	succinate dehydrogenase	succinate	fumarate
	1.8.4	oxidoreductase, acting on a sulfur group of donors	?	?
	5.3.4.1	protein disulfide isomerase	-S-S- bonds rearrangement	none
Unigene14615	2.4.2.30	NAD ADP-ribosyltransferase	NAD+	nicotinamide
	2.4.2.31	NAD(P)+protein-arginine ADP-ribosyltransferase	NAD+ & protein-l-arginine	nicotinamide & protein-omega-*N*-(ADP-d-ribosyl)-l-arginine
Unigene28009	2.5.1.21	farnesyl-diphosphate farnesyltransferase	(2E,6E)-farnesyl diphosphate	squalene
	1.14	oxidoreductase	?	?
Unigene32504	2.1.1	*O*-methyltransferase	?	?
	2.6.1.42	branched-chain-amino-acid transaminase	L-leucine	4-methyl-2-oxopentanoate
Unigene33780	2.7.7.9	UTP:glucose-1-phosphate uridylyltransferase	alpha-d-glucose 1-phosphate	UDP-glucose
	5.3.1.1	triose-phosphate isomerase	d-glyceraldehyde 3-phosphate	glycerone phosphate
Unigene34807	3.4.21	serine-type endopeptidase	?	?
	3.4.24	metalloendopeptidase	?	?
Unigene38918	2.7.2.3	phosphoglycerate kinase	3-phospho-d-glycerate	3-phospho-d-glyceroyl phosphate
	4.2.1.11	phosphopyruvate hydratase	2-phospho-d-glycerate	phosphoenolpyruvate
Unigene52468	2.1.1	methyltransferase	?	?
	2.5.1	transferase, transferring alkyl or aryl groups	?	?
Unigene9562	2.1.2.11	3-methyl-2-oxobutanoate hydroxymethyltransferase	5,10-methylenetetrahydrofolate & 3-methyl-2-oxobutanoate	tetrahydrofolate & 2-dehydropantoate
	6.3.2.1	pantoate-beta-alanine ligase	(R)-pantoate	(R)-pantothenate
Unigene9804	2.5.1.21	farnesyl-diphosphate farnesyltransferase	(2E,6E)-farnesyl diphosphate	squalene
	5.3.3.2	isopentenyl-diphosphate delta-isomerase	isopentenyl diphosphate	dimethylallyl diphosphate

**Table 2:** List of predicted enzymes with incomplete Enzyme Commission numbers.

Class	Sub-Subclass EC Number	Sub-Subclass Principal Enzyme Type(s)	Sub-Subclass Known Enzyme Entries	*P. variabilis* Number Sequences
*Oxidoreductases*	1.13.11	dioxygenase	80	26
	1.14.11	dioxygenase, hydroxylase, demethylase	56	9
	1.14.12	dioxygenase	23	1
	1.14.13	monooxygenase, hydroxylase	235	1
	1.14.16	monooxygenase	7	3
	1.14.17	monooxygenase	3	13
	1.14.19	desaturase	51	5
*Transferases*	2.7.10	protein-tyrosine kinase	2	72
	2.7.11	protein-serine/threonine kinase	33	100
*Hydrolases*	3.1.13	exoribonuclease	5	3
	3.1.21	endodeoxyribonuclease	9	1
	3.4.11	aminopeptidase	23	25
	3.4.13	dipeptidase	12	7
	3.4.15	peptidyl-dipeptidase	4	1
	3.4.16	serine-type carboxypeptidase	4	10
	3.4.17	metallocarboxypeptidase	20	25
	3.4.19	omega peptidase	12	9
	3.4.21	serine endopeptidase	100	136
	3.4.22	cysteine endopeptidase	58	20
	3.4.23	aspartic endopeptidase	41	70
	3.4.24	metalloendopeptidase	83	120
	3.4.25	threonine endopeptidase	2	34
*Lyases*	4.1.99	carbon-carbon lyases	16	3

## Supplementary Materials

The following are available online at http://www.mdpi.com/1660-3397/16/6/207/s1. Figure S1: Summary of the unigenes mapped to the public databases. Figure S2: Characteristics of the sequence homology search results. Figure S3: GO and KEGG pathways assignments. Figure S4: Maximum Likelihood (ML) phylogenetic tree of the predicted *P. variabilis* beta-*N*-acetylhexosaminidase (3.2.1.52) and their closest homologous sequences. Figure S5: Prediction of enzymes with two activities closely positioned in given metabolic pathway. Table S1: Species from the BLAST search of the barcode sequences from the *P. variabilis* holobiont transcriptome against the NCBI nr database. Table S2: KEGG pathways mapping summary. Table S3: List of predicted enzymes in *P. variabilis*. Table S4: List of enzymatic activities with relevance in treatment of rare diseases and other pharmaceutical fine chemicals predicted in *Protopalythoa variabilis* holo-transcriptome. Table S5: List of enzymatic activities with relevance in colorant, aromas, flavor, fragrance, cosmetic and hygienic industries predicted in *Protopalythoa variabilis* holo-transcriptome. Table S6: List of enzymatic activities with relevance in agrochemical, and food and feed industries predicted in *Protopalythoa variabilis* holo-transcriptome. Table S7: List of enzymatic activities with relevance in bioconversion and biopolymer synthesis predicted in *Protopalythoa variabilis* holo-transcriptome. Table S8: List of enzymatic activities with relevance in other industries predicted in *Protopalythoa variabilis* holo-transcriptome. Table S9: List of enzymatic activities with relevance in molecular biology and analytical applications predicted in *Protopalythoa variabilis* holo-transcriptome. Table S10: RNA-sequencing and assembling statistics. File S1: KEGG pathways.
