# Supplementary material for: The Holo-Transcriptome of the Zoantharian Protopalythoa variabilis (Cnidaria: Anthozoa): A Plentiful Source of Enzymes for Potential Application in Green Chemistry, Industrial and Pharmaceutical Biotechnology
Source: Mar Drugs. 2018 Jun 13;16(6):207. doi: 10.3390/md16060207 (PMC6025448; doi:10.3390/md16060207)
Supplement: Supplementary file 1 [file marinedrugs-16-00207-s001.zip › Supplementary Figures and Tables/Supplementary Figures 1-5.docx]

**Figure S1. Summary of the unigenes mapped to the public databases.**

A) Annotation hits of the poly-A transcripts from *P. variabilis* against the four protein databases used in this study. B) Venn diagram, constructed with Venny 2.0 [Oliveros, 2007], showing the unigenes cross-annotation between the databases.

Oliveros, J.C. 2007. Venny. An interactive tool for comparing lists with Venn's diagrams. (http://bioinfogp.cnb.csic.es/tools/venny/index.html)


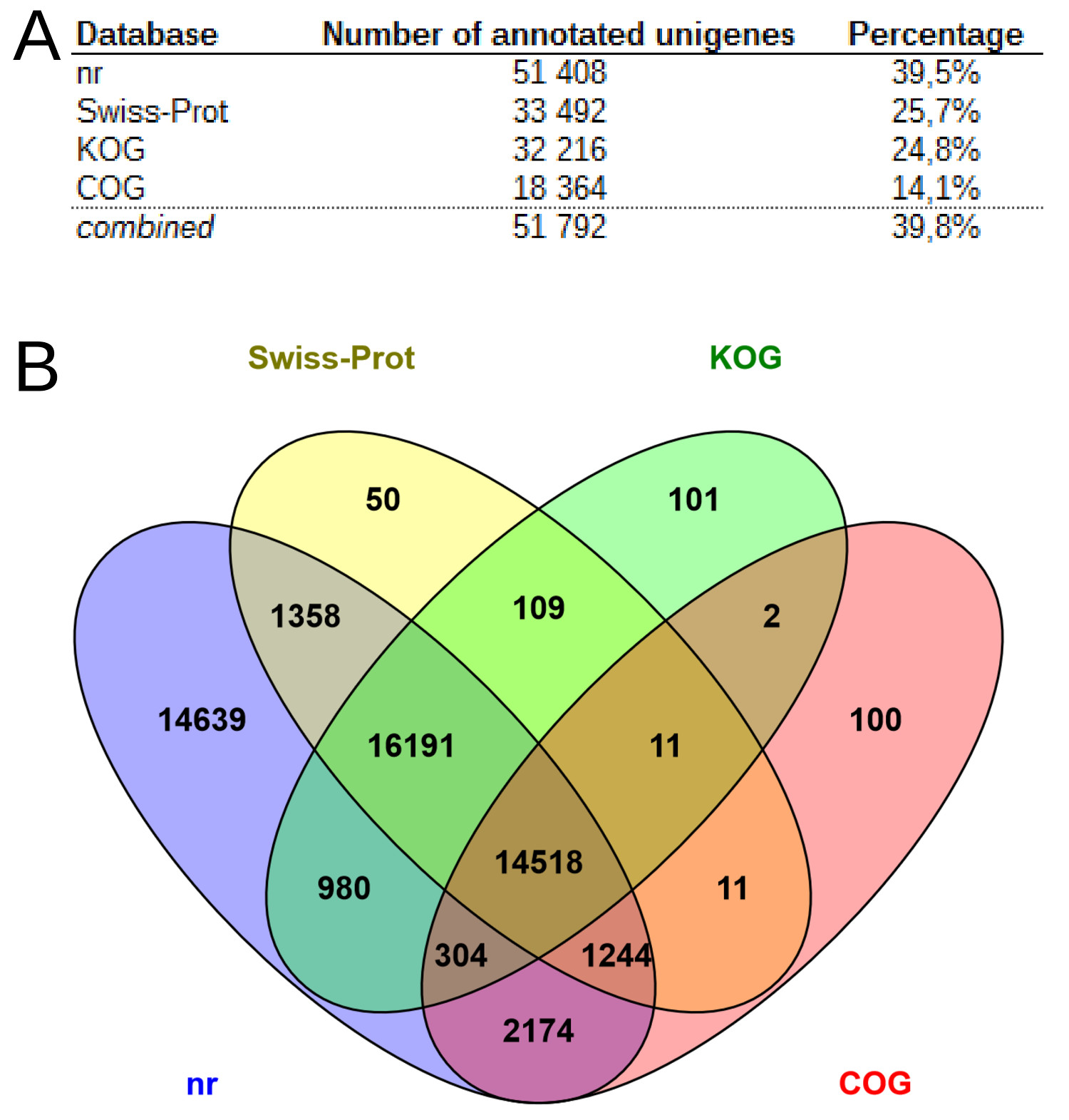


**Figure S2. Characteristic of the sequence homology search results.**

A) Annotation distribution based on the size length of the unigenes. B) BLASTx E-value distribution among mapped unigenes. C) BLASTx percent identity distribution among mapped unigenes.


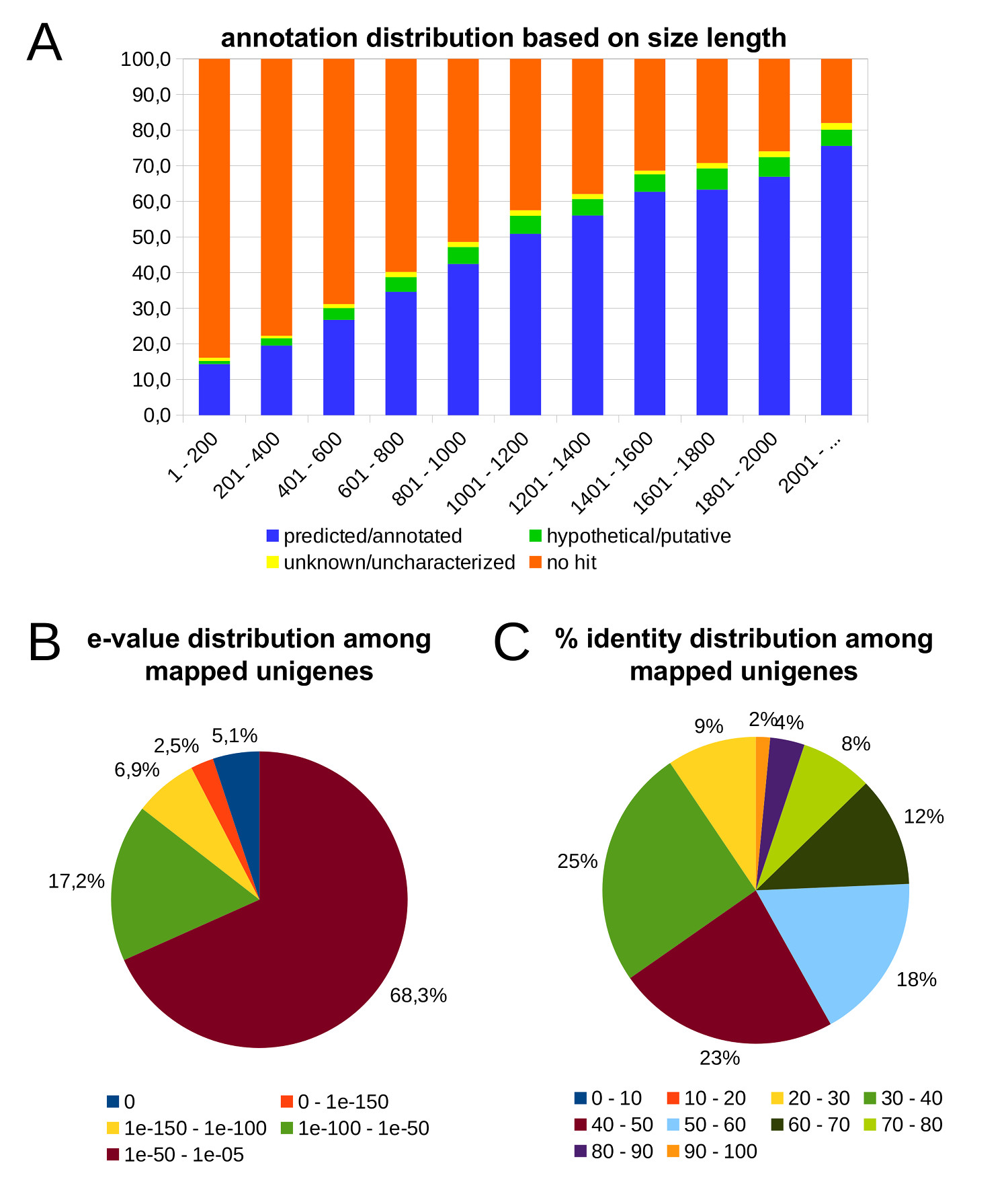


**Figure S3. GO and KEGG pathways assignment.**

A) Gene Ontology (GO) classification of the assembled *Protopalythoa variabilis* unigenes, categorized into the Cellular Component, Molecular Function, and Biological Process categories. B) Classification of the pathway assignment based on Metabolism, Organismal Systems, Genetic Information Processing, and Environmental Information Processing KEGG categories.


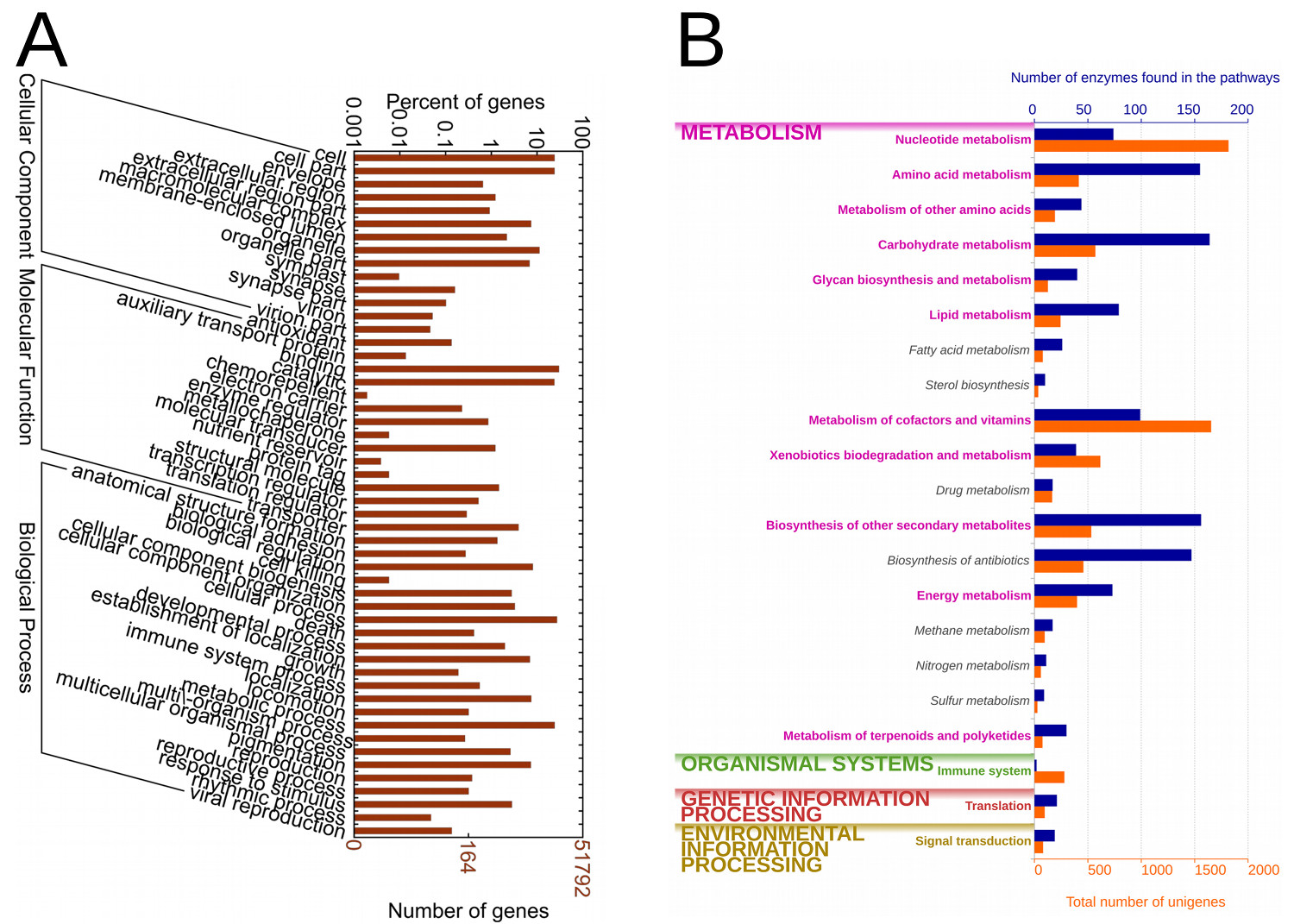


**Figure S4. Maximum Likelihood (ML) phylogenetic tree of the predicted *P. variabilis* beta-N-acetylhexosaminidase (3.2.1.52) and their closest homologous sequences.** A, multi-aligned sequences were retrieved from the UniProt database and were from archaea and bacteria (yellow), protist, algae, fungi and ichthyosporea (green), and metazoa (blue). Alignment of the catalytic domain of all sequences was performed as described in the materials and methods section. All positions with less than 95% site coverage were eliminated. Branches with less than 25% bootstrap replicates were collapsed. B, a close up of the catalytic region. The conserved catalytic glutamic acid is marked.


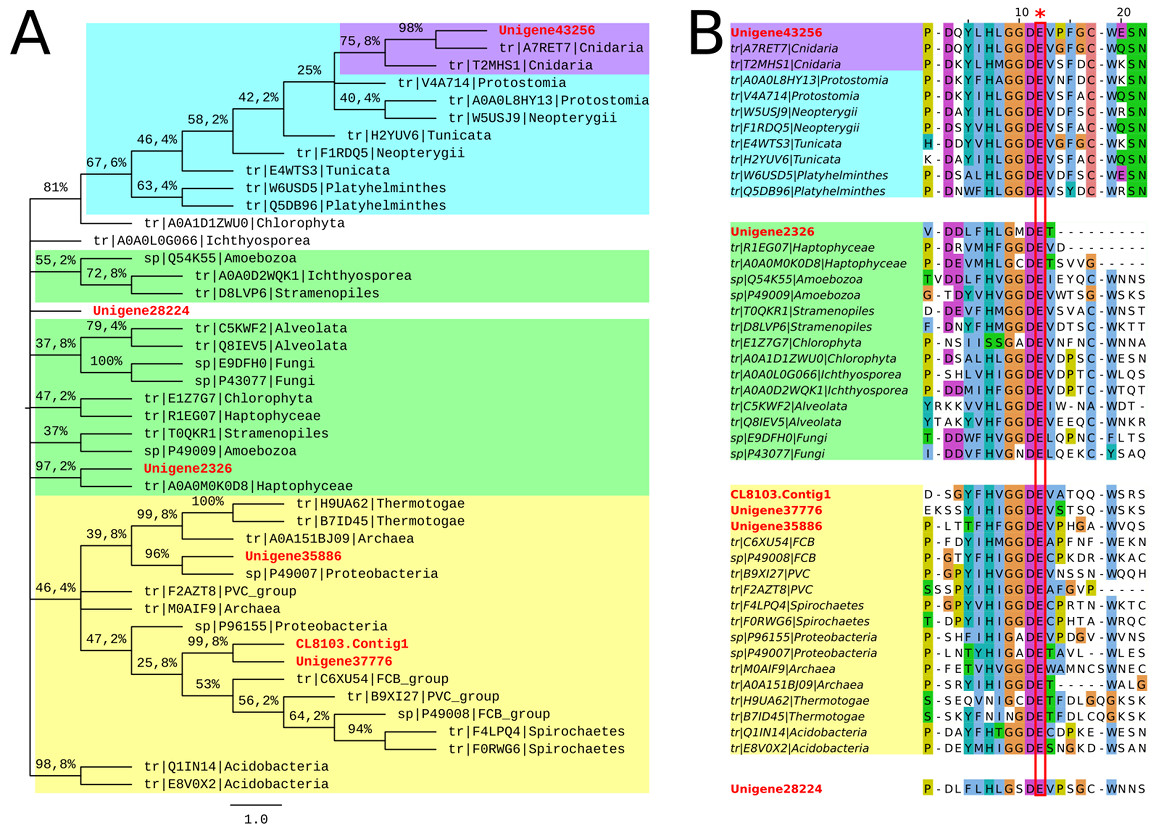


**Figure S5. Prediction of enzymes with two activities closely positioned in a metabolic pathway.**


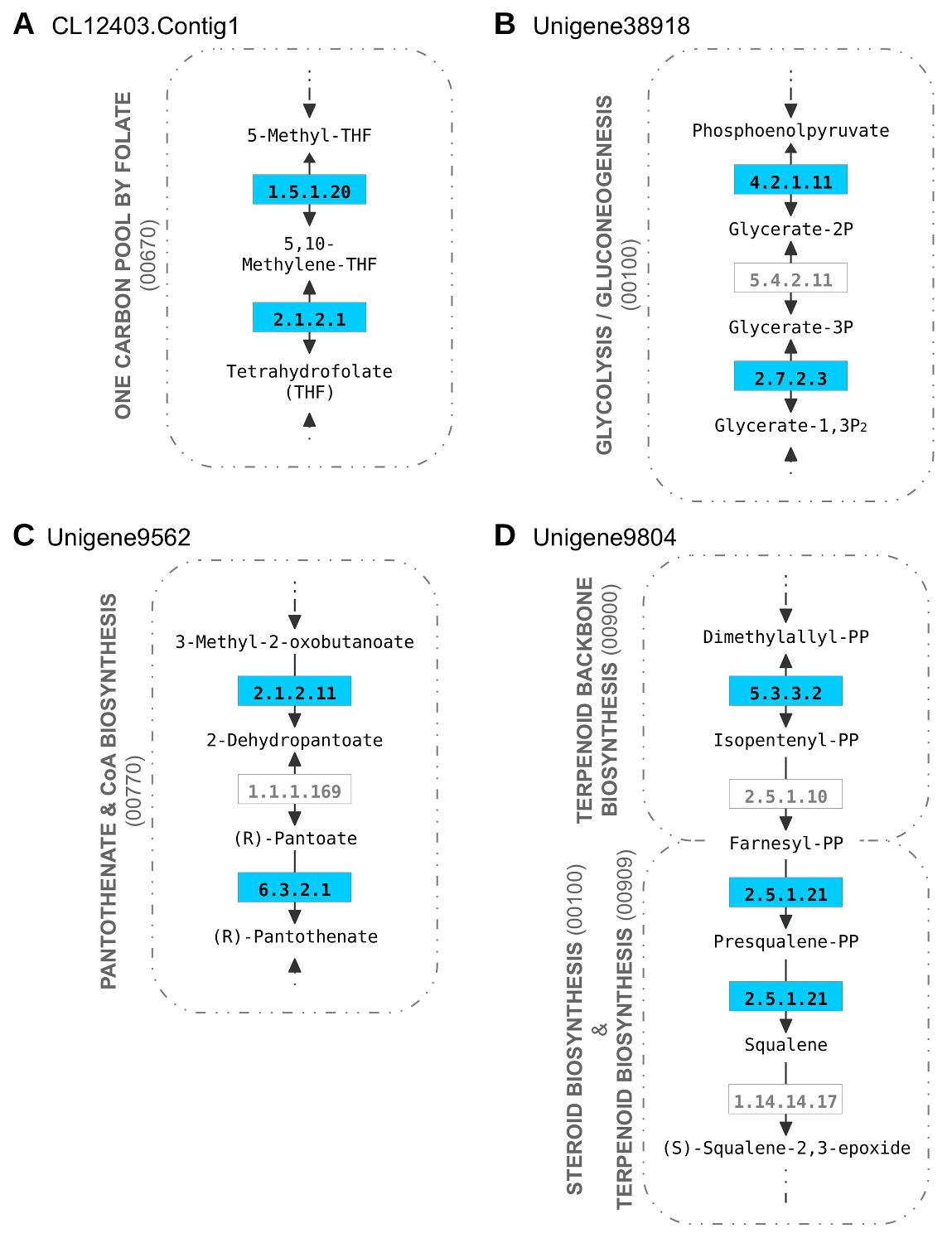


**A.** *one carbon pool by folate* – the product from the assembled transcript CL12403.contig1 had an oxidoreductase (EC:1.5.1.20) and a transferase (EC:2.1.2.1) activities predicted. Both enzymatic reactions were found to occur successively on derivatives of the cofactor tetrahydrofolate (THF) in the “One carbon pool by folate” pathway. Interestingly, these two activities were already covered by other predicted independent single-activity enzymes (unigene16194 and unigene60589 for EC:2.1.2.1, and unigene42790 and unigene31941 for EC:1.5.1.20) (Supplementary Table 1 and Supplementary File 1 map00670).

**B.** *glycolysis / gluconeogenesis* – the activities of lyase (EC:4.2.1.11) and transferase (EC:2.7.2.3) could be predicted from the unigene38918, and could be mapped to the “Glycolysis / gluconeogenesis” KEGG pathway . Both target phosphate derivative of glycerate, although they are separated by another enzymatic reaction.

**C.** *pantothenate & CoA biosynthesis* – unigne9562’s product have both a transferase (EC:2.1.2.11) and a ligase (EC:6.3.2.1) activities mapped to the “Pantothenate and CoA biosynthesis” pathway. As for unigene38918, both enzymatic reactions were not successive but separated by another one. The intermediary activity (EC:1.1.1.169) was also predicted in another single-activity enzyme (Supplementary Table 1 and Supplementary File 1 map00770).

**D.** *steroid / terpenoid biosynthesis* – the enzyme from unigene9804 has a first isomerase activity (EC:5.3.3.2) mapped to the “Terpenoid backbone biosynthesis” pathway. However, while its second activity was mapped to the first step in the steroid / terpenoid biosynthesis, it could also have been suggested as the similar last activity of the Terpenoid backbone biosynthesis, successively to the identified isomerase activity. Particularly because 1) both enzymes activities belongs to the class transferase, transfering alkyl or aryl groups other than methyl groups (EC:2.5.1), and 2) there is a lack of available marine sequences in the Swiss-Prot database for both EC:2.5.1.10 and EC:2.5.1.21 enzymes with the closest one being of a green algae for EC:2.5.1.21. However, closer analysis by sequence alignment seems to reject this possibility and indeed currently classify the second activity of unigene9804 as EC:2.5.2.21.
