# Supplementary material for: The Holo-Transcriptome of the Zoantharian Protopalythoa variabilis (Cnidaria: Anthozoa): A Plentiful Source of Enzymes for Potential Application in Green Chemistry, Industrial and Pharmaceutical Biotechnology
Source: Mar Drugs. 2018 Jun 13;16(6):207. doi: 10.3390/md16060207 (PMC6025448; doi:10.3390/md16060207)
Supplement: Supplementary file 1 [file marinedrugs-16-00207-s001.zip › Supplementary Figures and Tables/Supplementary File 1 _ KEGG pathways/Legend.docx]

**Supplementary File 1. KEGG pathways maps.** The maps were directly generated by the Kyoto Encyclopedia of Genes and Genomes (KEGG) through Blast2GO. Notation and color codes used in the diagrams are as defined in the KEGG documentation http://www.genome.jp/kegg/document/help_pathway.html and http://www.genome.jp/kegg/kegg1c.html.
