# Supplementary material for: The Holo-Transcriptome of the Zoantharian Protopalythoa variabilis (Cnidaria: Anthozoa): A Plentiful Source of Enzymes for Potential Application in Green Chemistry, Industrial and Pharmaceutical Biotechnology
Source: Mar Drugs. 2018 Jun 13;16(6):207. doi: 10.3390/md16060207 (PMC6025448; doi:10.3390/md16060207)
Supplement: Supplementary file 1 [file marinedrugs-16-00207-s001.zip › Supplementary Figures and Tables/Supplementary Table 04 - treatment of rare diseases and other pharmaceutical fine chemicals.docx]

**Supplementary Table 4. List of enzymatic activities with relevance in treatment of rare diseases and other pharmaceutical fine chemicals predicted in *Protopalythoa variabilis* holo-transcriptome.**

| **enzyme name** | **EC number** | **Indication** |
| --- | --- | --- |
| ***> Lysosomal Storage Disease (LSD)*** |  |  |
| alpha-glucosaminide acetyltransferase | 2.3.1.78 | MPS III (Sanfilippo’s syndrome) type C |
| UDP-N-acetylglucosamine | 2.7.8.17 | mucolipidosis (ML) |
| sphingomyelinase | 3.1.4.12 | Niemann-Pick disease |
| N-acetylgalactosamine-4-sulfatase | 3.1.6.12 | MPS VI (Maroteaux-Lamy syndrome) |
| iduronate-2-sulfatase | 3.1.6.13 | MPS II (Hunter’s syndrome) |
| N-acetylglucosamine-6-sulfatase | 3.1.6.14 | MPS III (Sanfilippo’s syndrome) type D, MPS IV (Morquio’s syndrome) type A |
| alpha-glucosidase | 3.2.1.3 | Pompe disease |
| neuraminidase | 3.2.1.18 | sialidosis |
| alpha-galactosidase | 3.2.1.22 | Fabry disease |
| beta-galactosidase | 3.2.1.23 | MPS IV (Morquio’s syndrome) type B |
| beta-glucuronidase | 3.2.1.31 | MPS VII (Sly’s syndrome) |
| beta-glucocerebrosidase | 3.2.1.45 | Gaucher's disease |
| beta-galactosidase | 3.2.1.46 | Krabbe disease |
| beta-N-acetylhexosaminidase | 3.2.1.52 | Sandhoff disease, Tay–Sachs disease |
| ***> cancer*** |  |  |
| L-asparaginase | 3.5.1.1 | acute lymphoblastic leukemia (ALL) |
| arginine deiminase | 3.5.3.15 | invasive malignant melanoma, hepatocellular carcinoma (HCC) |
| ***> other rare diseases*** |  |  |
| urate oxidase | 1.7.3.3 | hyperuricemia |
| deoxyribonuclease I | 3.1.21.1 | cystic fibrosis |
| adenosine deaminase | 3.5.4.4 | SCID |
| ***> transfusion & transplantation*** |  |  |
| superoxide dismutase (SOD) | 1.15.1.1 | organ tissue transplantation |
| transglutaminase | 2.3.2.13 | PEGylation of protein drug |
| beta-glucosidase | 3.2.1.21 | universal blood |
| alpha-galactosidase | 3.2.1.22 | universal blood |
| beta-galactosidase | 3.2.1.23 | universal blood |

MPS: mucopolysaccharidosis disorders ; SCID: severe combined immunodeficiency
