# Supplementary material for: The Holo-Transcriptome of the Zoantharian Protopalythoa variabilis (Cnidaria: Anthozoa): A Plentiful Source of Enzymes for Potential Application in Green Chemistry, Industrial and Pharmaceutical Biotechnology
Source: Mar Drugs. 2018 Jun 13;16(6):207. doi: 10.3390/md16060207 (PMC6025448; doi:10.3390/md16060207)
Supplement: Supplementary file 1 [file marinedrugs-16-00207-s001.zip › Supplementary Figures and Tables/Supplementary Table 05 - colorant, aromas, flavor, fragrance, cosmetic and hygienic industries.docx]

**Supplementary Table 5. List of enzymatic activities with relevance in colorant, aromas, flavor, fragrance, cosmetic and hygienic industries predicted in *Protopalythoa variabilis* holo-transcriptome.**

| **enzyme name** | **EC number** | **Usage** |
| --- | --- | --- |
| dehydrogenases | 1.1.1.- | monoterpene modification |
| peroxidases | 1.11.1.- | production of flavor compounds, skin protection in cosmetic products |
| catalase | 1.11.1.6 | skin protection in cosmetic products |
| peroxidase | 1.11.1.7 | production of flavor compounds |
| lipoxygenases | 1.13.11.- | monoterpene modification, production of flavor compounds |
| dioxygenases | 1.14.12.- | monoterpene modification |
| monooxygenases | 1.14.13.- | monoterpene modification |
| superoxide dismutase (SOD) | 1.15.1.1 | skin protection in cosmetic products |
| dehydrogenases | 1.2.1.- | monoterpene modification |
| amine oxidases | 1.4.3.- | production of aldehyde compounds (production of vanillin) |
| O-methyltransferase (FaOMT) | 2.1.1.- | production of flavor compounds |
| 4-hydroxybenzoate polyprenyltransferase | 2.5.1.39 | production of terpenoid flavor compounds |
| esterase | 3.1.1.1 | production of ester flavor compounds for the cosmtic industry |
| lipase | 3.1.1.3 | production of ester flavor compounds for the cosmtic industry |
| glycosidases | 3.2.1.- | liberate the aromas from the non-volatile conjugates |
| alpha-amylase | 3.2.1.1 | dental hygiene |
| polygalacturonase | 3.2.1.15 | extraction of flavor compounds, extraction of colorant compounds |
| beta-glucosidase | 3.2.1.21 | extraction of flavor compounds |
| 1,4-alpha-glucosidase | 3.2.1.3 | dental hygiene, extraction of colorant compounds |
| cellulase | 3.2.1.4 | extraction of flavor compounds |
| 4-hydroxybenzaldehyde synthases | 3.7.1.- | production of amino acid-derived flavor compounds, monoterpene modification |
| methylketone synthases | 4.1.1.- | monoterpene modification, production of aroma compounds production |
| fructose-bisphosphate aldolase | 4.1.2.13 | production of aldehyde compounds (production of strawberry aroma) |
| hydroperoxide lyases | 4.2.1.- | production of flavor compounds |
