# Supplementary material for: The Holo-Transcriptome of the Zoantharian Protopalythoa variabilis (Cnidaria: Anthozoa): A Plentiful Source of Enzymes for Potential Application in Green Chemistry, Industrial and Pharmaceutical Biotechnology
Source: Mar Drugs. 2018 Jun 13;16(6):207. doi: 10.3390/md16060207 (PMC6025448; doi:10.3390/md16060207)
Supplement: Supplementary file 1 [file marinedrugs-16-00207-s001.zip › Supplementary Figures and Tables/Supplementary Table 06 - agrochemical, and food and feed industries.docx]

**Supplementary Table 6. List of enzymatic activities with relevance in agrochemical, and food and feed industries predicted in *Protopalythoa variabilis* holo-transcriptome.**

| **enzyme name** | **EC number** | **Usage** |
| --- | --- | --- |
| ***> agrochemical*** |  |  |
| esterase | 3.1.1.1 | herbicide synthesis |
| lipase | 3.1.1.3 | herbicide synthesis |
| haloalkane dehalogenase | 3.8.1.5 | herbicide synthesis |
| ***> food & feed*** |  |  |
| catalase | 1.11.1.6 | beverages, starch processing, eggs |
| peroxidase | 1.11.1.7 | starch processing |
| gamma-glutamyltransferase | 2.3.2.13 | bakery, milk and cheese, meat and fish |
| 1,4-alpha-glucan branching enzyme | 2.4.1.18 | starch processing |
| lipase | 3.1.1.3 | bakery, milk and cheese, meat and fish, starch processing, vegetable fermentation |
| phospholipase A1 | 3.1.1.32 | milk and cheese |
| phospholipase A2 | 3.1.1.4 | bakery, starch processing, eggs |
| lysophospholipase | 3.1.1.5 | beverages, starch processing |
| hydroxyacylglutathione hydrolase | 3.1.2.6 | bakery |
| ribonuclease P | 3.1.26.5 | yeast extract production |
| alpha-amylase | 3.2.1.1 | beverages, starch processing, bakery |
| chitinase | 3.2.1.14 | single cell proteins production for animal and aquaculture feed |
| polygalacturonase | 3.2.1.15 | beverages clarification |
| lysozyme | 3.2.1.17 | preservation |
| beta-amylase | 3.2.1.2 | beverages, starch processing |
| beta-glucosidase | 3.2.1.21 | sugar production, ethanol production |
| alpha-galactosidase | 3.2.1.22 | sugar production |
| beta-galactosidase | 3.2.1.23 | milk processing |
| 1,4-alpha-glucosidase | 3.2.1.3 | beverages, starch processing, bakery |
| cellulase | 3.2.1.4 | beverages, starch processing, bakery |
| alpha-L-arabinofuranosidase | 3.2.1.55 | bakery |
| endo-1,3(4)-beta-glucanase | 3.2.1.6 | beverages |
| asparaginase | 3.5.1.1 | bakery, starch processing, fruits and vegetables |
| urease | 3.5.1.5 | feed industry |
| AMP deaminase | 3.5.4.6 | yeast extract production |
| xylose isomerase | 5.3.1.5 | sugar production |
| glucose-6-phosphate isomerase | 5.3.1.9 | sugar production |
