# Supplementary material for: The Holo-Transcriptome of the Zoantharian Protopalythoa variabilis (Cnidaria: Anthozoa): A Plentiful Source of Enzymes for Potential Application in Green Chemistry, Industrial and Pharmaceutical Biotechnology
Source: Mar Drugs. 2018 Jun 13;16(6):207. doi: 10.3390/md16060207 (PMC6025448; doi:10.3390/md16060207)
Supplement: Supplementary file 1 [file marinedrugs-16-00207-s001.zip › Supplementary Figures and Tables/Supplementary Table 07 - bioconversion and biopolymers.docx]

**Supplementary Table 7. List of enzymatic activities with relevance in bioconversion and biopolymer synthesis predicted in *Protopalythoa variabilis* holo-transcriptome.**

| **enzyme name** | **EC number** | **Usage** |
| --- | --- | --- |
| ***> bioblock and biopolymer production*** |  |  |
| 3-oxoacyl-ACP reductase | 1.1.1.100 | polyhydroxyalkanoate (PHA) precursor synthesis |
| alcohol dehydrogenase | 1.1.1.2 | synthesis of primary alcohols |
| peroxidase | 1.11.1.7 | phenol polymers synthesis, vinyl polymers synthesis |
| acetyl-CoA C-acyltransferase | 2.3.1.16 | polyhydroxyalkanoate (PHA) precursor synthesis |
| gamma-glutamyltransferase | 2.3.2.13 | crosslinking of proteins |
| glycosyltransferases | 2.4.1.- | various biopolymer synthesis |
| lipase | 3.1.1.3 | poly(lactic acid) (PLA) precursor synthesis, ring-opening polymerization (ROP) of lactones, polycondensation of hydroxycarboxylic acids |
| epoxide hydrolase | 3.3.2.9 | chiral synthesis |
| N-acyl-aliphatic-L-amino acid amidohydrolase | 3.5.1.14 | production of L-amino acids |
| enoyl-CoA hydratase | 4.2.1.17 | polyhydroxyalkanoate (PHA) precursor synthesis |
| nitrile hydratase | 4.2.1.84 | acrylamide synthesis (for polyacrylamide synthesis) |
| xylose isomerase | 5.3.1.5 | 5-hydroxymethylfurfural (HMF) synthesis (for various chemical compounds synthesis) |
| ***> biomass conversion*** |  |  |
| lipase | 3.1.1.3 | biolubricant production, biodiesel production, bioconversion of plant oils |
| alpha-amylase | 3.2.1.1 | bioethanol production |
| polygalacturonase | 3.2.1.15 | degradadation of lignocellulosic biomass |
| beta-glucosidase | 3.2.1.21 | cellulose processing, lignocellulose processing |
| alpha-galactosidase | 3.2.1.22 | hydrolysis of hemicelluloses, processing of lignocellulosic biomass |
| beta-galactosidase | 3.2.1.23 | hydrolysis of hemicelluloses |
| beta-mannosidase | 3.2.1.25 | hydrolysis of hemicelluloses, processing of lignocellulosic biomass |
| xylan 1,4-beta-xylosidase | 3.2.1.37 | hydrolysis of hemicelluloses, processing of lignocellulosic biomass |
| endo-1,3-beta-D-glucosidase | 3.2.1.39 | hydrolysis of hemicelluloses, processing of lignocellulosic biomass |
| cellulase | 3.2.1.4 | cellulose processing, lignocellulose processing |
| alpha-L-arabinofuranosidase | 3.2.1.55 | hydrolysis of hemicelluloses, processing of lignocellulosic biomass |
| endo-1,3(4)-beta-glucanase | 3.2.1.6 | hydrolysis of hemicelluloses, processing of lignocellulosic biomass |
