# Supplementary material for: The Holo-Transcriptome of the Zoantharian Protopalythoa variabilis (Cnidaria: Anthozoa): A Plentiful Source of Enzymes for Potential Application in Green Chemistry, Industrial and Pharmaceutical Biotechnology
Source: Mar Drugs. 2018 Jun 13;16(6):207. doi: 10.3390/md16060207 (PMC6025448; doi:10.3390/md16060207)
Supplement: Supplementary file 1 [file marinedrugs-16-00207-s001.zip › Supplementary Figures and Tables/Supplementary Table 08 - other industries.docx]

**Supplementary Table 8. List of enzymatic activities with relevance in other industries predicted in *Protopalythoa variabilis* holo-transcriptome.**

| **enzyme name** | **EC number** | **Usage** |
| --- | --- | --- |
| ***> cleaning*** |  |  |
| lipase | 3.1.1.3 | detergent additive for the removal of grease |
| alpha-amylase | 3.2.1.1 | laundry and dish detergent for carbohydrate removal |
| beta-mannosidase | 3.2.1.25 | mannan removal |
| cellulase | 3.2.1.4 | laundry detergent for color clarification |
| ***> textiles*** |  |  |
| catalase | 1.11.1.6 | cotton pre-treatment and finishing process |
| gamma-glutamyltransferase (transglutaminase) | 2.3.2.13 | treatment of wool and leather |
| lipase | 3.1.1.3 | leather degreasing, cotton fabric pre-treatment and finishing process, textiles processing (bio-stoning, bio-polishing, lubricants removal) |
| alpha-amylase | 3.2.1.1 | leather treatment (fiber splitting), textile desizing |
| polygalacturonase (pectinase) | 3.2.1.15 | cotton processing (bio-scouring, bio-bleaching), retting of textile fibres |
| 1,4-alpha-glucosidase | 3.2.1.3 | cotton bio-bleaching |
| cellulase | 3.2.1.4 | cotton finishing (bio-softening, bio-stoning and depilling), softening of jute |
| nitrile hydratase | 4.2.1.84 | surface modification of synthetic fibres |
| ***> pulp and paper*** |  |  |
| lipase | 3.1.1.3 | pitch control to reduce deposit involved in lower production level |
| alpha-amylase | 3.2.1.1 | deinking and drainage improvement |
| cellulase | 3.2.1.4 | deinking and drainage improvement |
| ***> waste treatment and bioremediation*** |  |  |
| glutathione gamma-glutamylcysteinyltransferase (phytochelatin) | 2.3.2.15 | heavy metal bioremediation |
| lipase | 3.1.1.3 | crude oil degradation |
| alpha-amylase | 3.2.1.1 | vegetables wastes treatment |
| 1,4-alpha-glucosidase | 3.2.1.3 | vegetables wastes treatment |
| epoxide hydrolase | 3.3.2.9 | organic compounds biodegradation |
| nitrile hydratase | 4.2.1.84 | nitriles biodegradation from wastes |
