# Supplementary material for: The Holo-Transcriptome of the Zoantharian Protopalythoa variabilis (Cnidaria: Anthozoa): A Plentiful Source of Enzymes for Potential Application in Green Chemistry, Industrial and Pharmaceutical Biotechnology
Source: Mar Drugs. 2018 Jun 13;16(6):207. doi: 10.3390/md16060207 (PMC6025448; doi:10.3390/md16060207)
Supplement: Supplementary file 1 [file marinedrugs-16-00207-s001.zip › Supplementary Figures and Tables/Supplementary Table 09 - molecular biology and analytical applications.docx]

**Supplementary Table 9. List of enzymatic activities with relevance in molecular biology and analytical applications predicted in *Protopalythoa variabilis* holo-transcriptome.**

| **enzyme name** | **EC number** | **Remark** |
| --- | --- | --- |
| ***> modifying enzymes*** |  |  |
| RNA-directed RNA polymerase | 2.7.7.48 |  |
| RNA-directed DNA polymerase | 2.7.7.49 |  |
| DNA-directed RNA polymerase | 2.7.7.6 |  |
| DNA-directed DNA polymerase | 2.7.7.7 |  |
| exodeoxyribonuclease | 3.1.11.4 |  |
| deoxyribonuclease I | 3.1.21.1 |  |
| deoxyribonuclease IV | 3.1.21.2 |  |
| type II site-specific deoxyribonuclease | 3.1.21.4 | i.e. restriction enzyme |
| deoxyribonuclease II | 3.1.22.1 |  |
| ribonuclease III | 3.1.26.3 |  |
| ribonuclease H | 3.1.26.4 |  |
| ribonuclease P | 3.1.26.5 |  |
| ribonuclease T2 | 3.1.27.1 |  |
| DNA ligase | 6.5.1.1 |  |
| RNA ligase | 6.5.1.3 |  |
| ***> enzymes for glycobiology*** |  |  |
| exo-alpha-sialidase | 3.2.1.18 |  |
| alpha-mannosidase | 3.2.1.24 |  |
| alpha-L-fucosidase | 3.2.1.51 |  |
| beta-N-acetylhexosaminidase | 3.2.1.52 |  |
| ***> enzymes for epigenetics*** |  |  |
| DNA (cytosine-5-)-methyltransferase | 2.1.1.37 | i.e. CpG/GpC methylase |
| ***> enzymes for analytical applications*** |  |  |
| peroxidase | 1.11.1.7 | hydrogen peroxide quantification |
| urate hydroxylase | 1.7.3.3 | uric acid quantification |
| glycerol kinase | 2.7.1.30 | triglyceride quantification |
| triacylglycerol lipase | 3.1.1.3 | triglyceride quantification |
| urease | 3.5.1.5 | urea quantification |
| ***> enzymes for tool production*** |  |  |
| glutathione gamma-glutamylcysteinyltransferase | 2.3.2.15 | nanocrystal (quantum dot) production used as fluorescent label |
