# Supplementary figures and images for: The Holo-Transcriptome of the Zoantharian Protopalythoa variabilis (Cnidaria: Anthozoa): A Plentiful Source of Enzymes for Potential Application in Green Chemistry, Industrial and Pharmaceutical Biotechnology (part 2 of 2)
Source: Mar Drugs. 2018 Jun 13;16(6):207. doi: 10.3390/md16060207 (PMC6025448; doi:10.3390/md16060207)

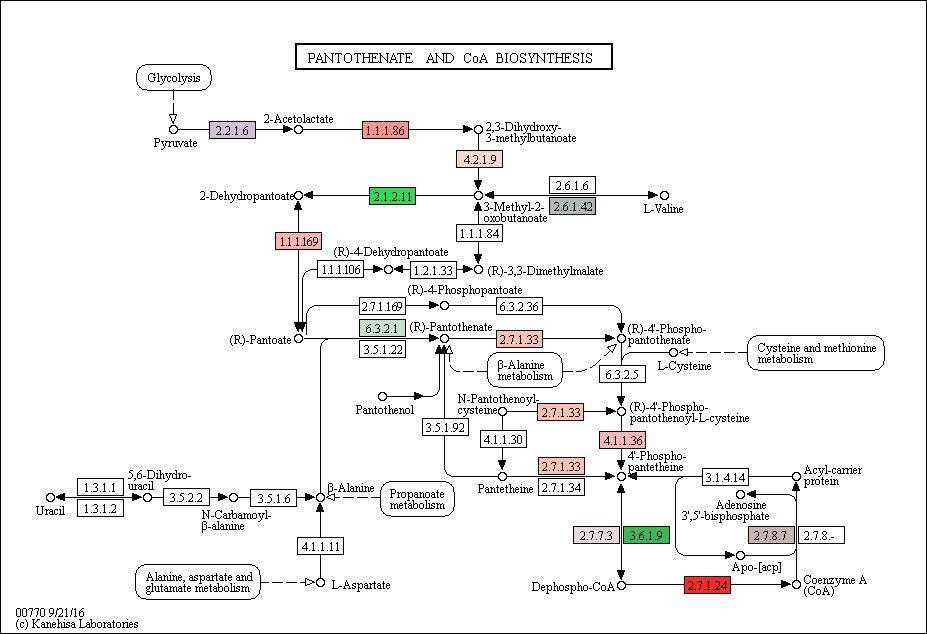

Supplement: Supplementary file 1 [file marinedrugs-16-00207-s001.zip › Supplementary Figures and Tables/Supplementary File 1 _ KEGG pathways/map00770 (Pantothenate and CoA biosynthesis) [13 enz found].png]

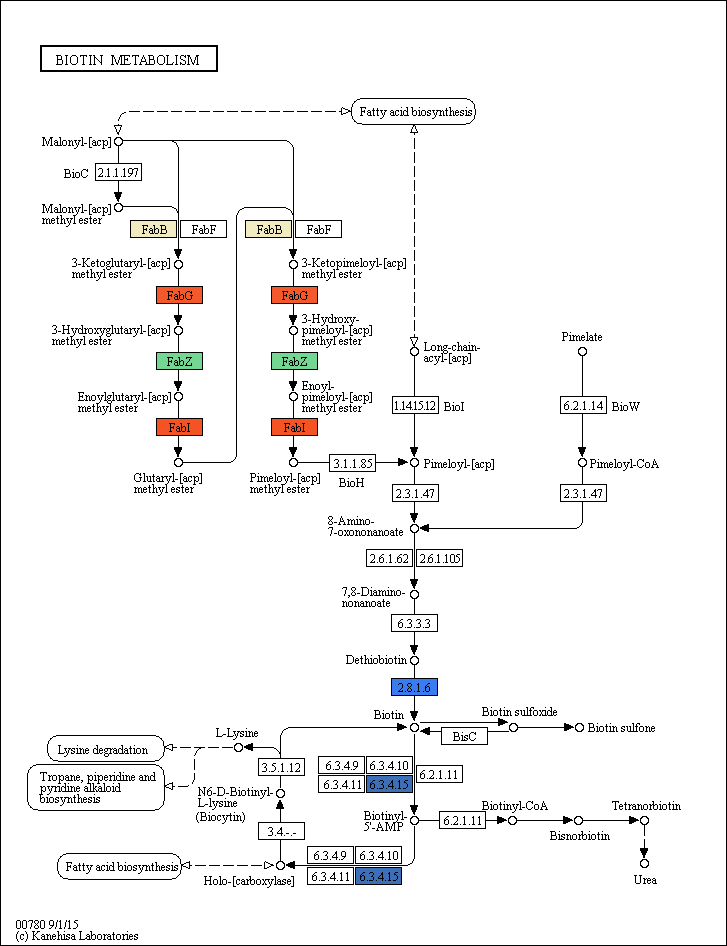

Supplement: Supplementary file 1 [file marinedrugs-16-00207-s001.zip › Supplementary Figures and Tables/Supplementary File 1 _ KEGG pathways/map00780 (Biotin metabolism) [6 enz found].png]

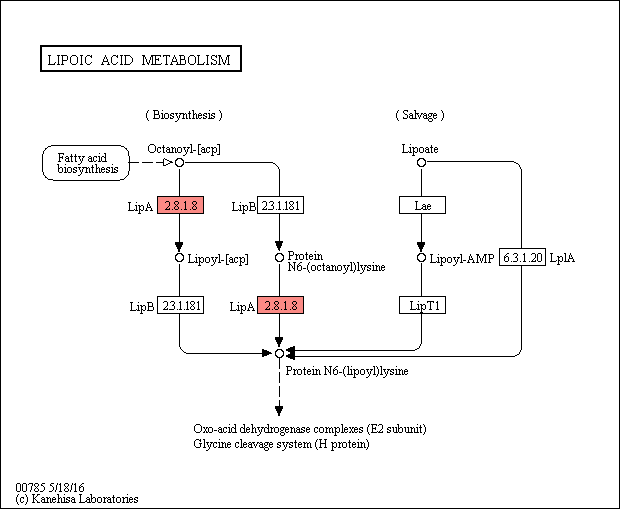

Supplement: Supplementary file 1 [file marinedrugs-16-00207-s001.zip › Supplementary Figures and Tables/Supplementary File 1 _ KEGG pathways/map00785 (Lipoic acid metabolism) [1 enz found].png]

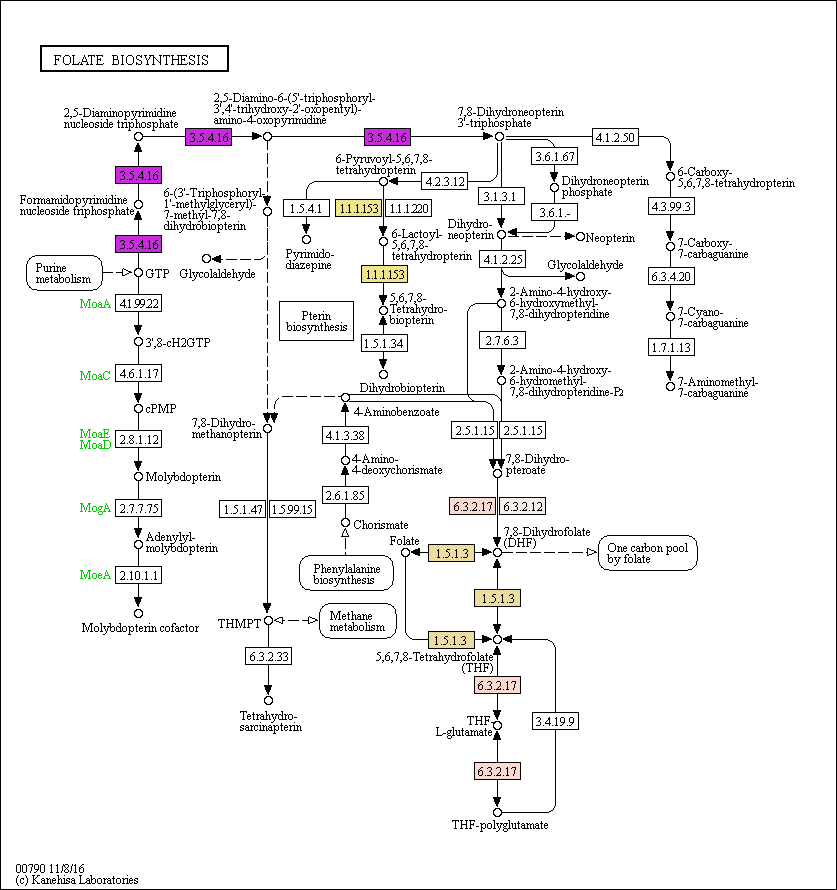

Supplement: Supplementary file 1 [file marinedrugs-16-00207-s001.zip › Supplementary Figures and Tables/Supplementary File 1 _ KEGG pathways/map00790 (Folate biosynthesis) [5 enz found].png]

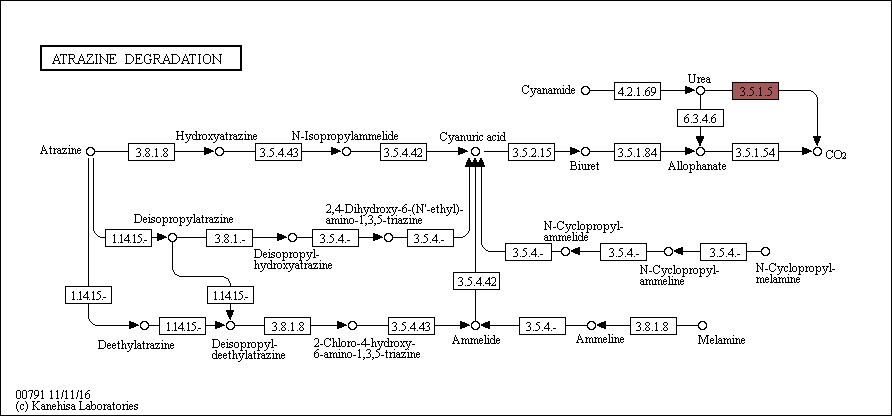

Supplement: Supplementary file 1 [file marinedrugs-16-00207-s001.zip › Supplementary Figures and Tables/Supplementary File 1 _ KEGG pathways/map00791 (Atrazine degradation) [1 enz found].png]

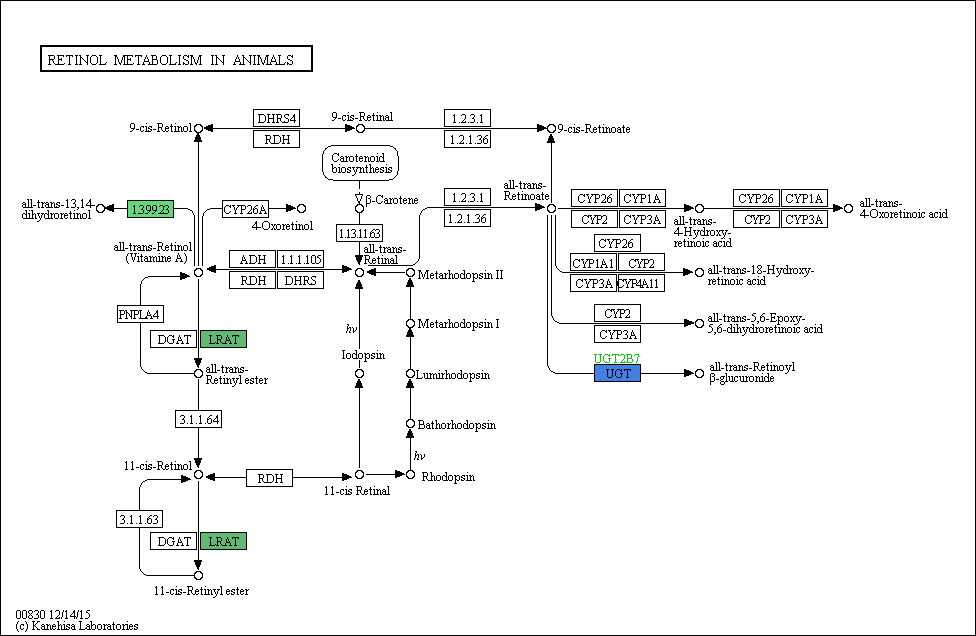

Supplement: Supplementary file 1 [file marinedrugs-16-00207-s001.zip › Supplementary Figures and Tables/Supplementary File 1 _ KEGG pathways/map00830 (Retinol metabolism in animals) [3 enz found].png]

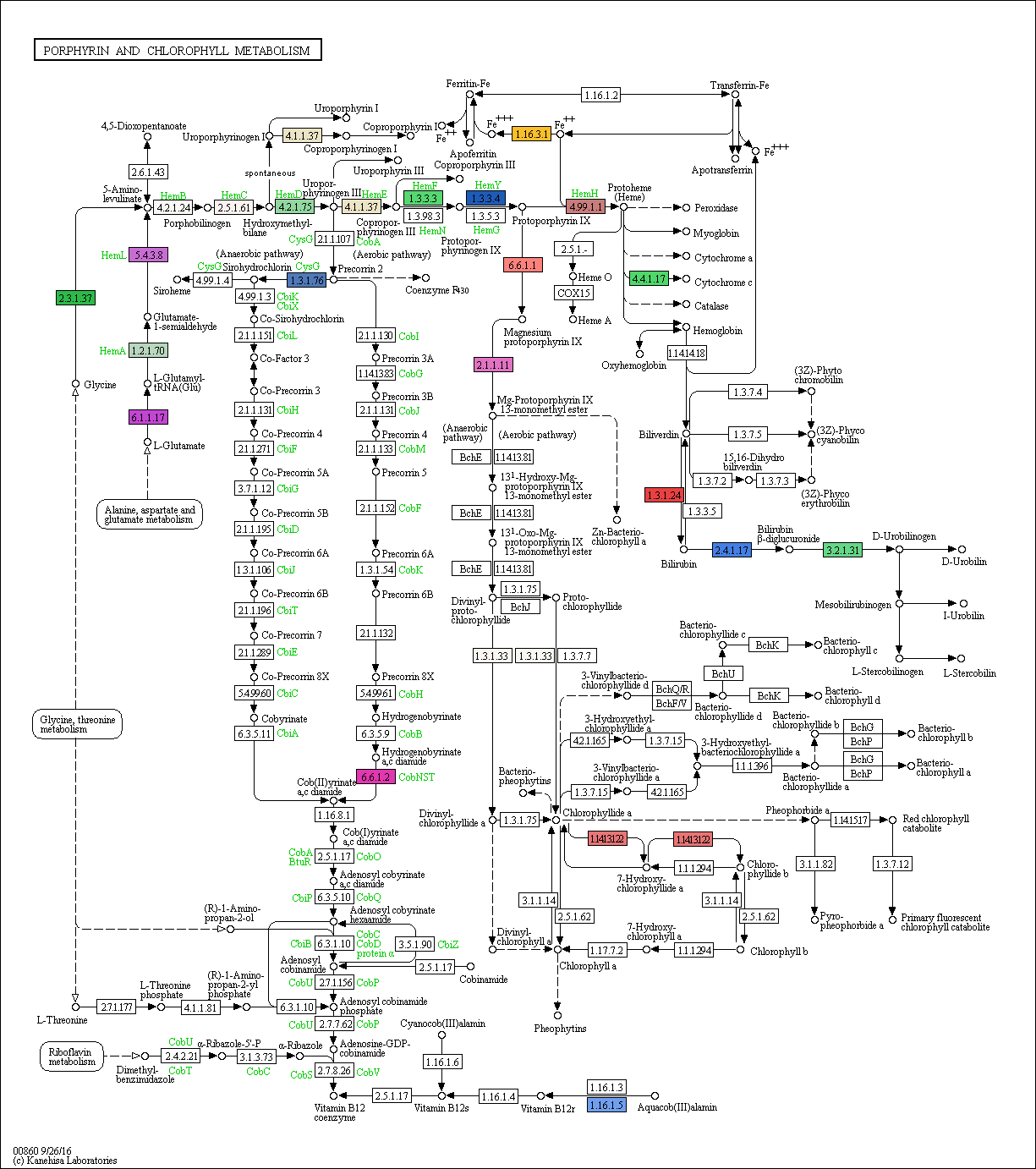

Supplement: Supplementary file 1 [file marinedrugs-16-00207-s001.zip › Supplementary Figures and Tables/Supplementary File 1 _ KEGG pathways/map00860 (Porphyrin and chlorophyll metabolism) [23 enz found].png]

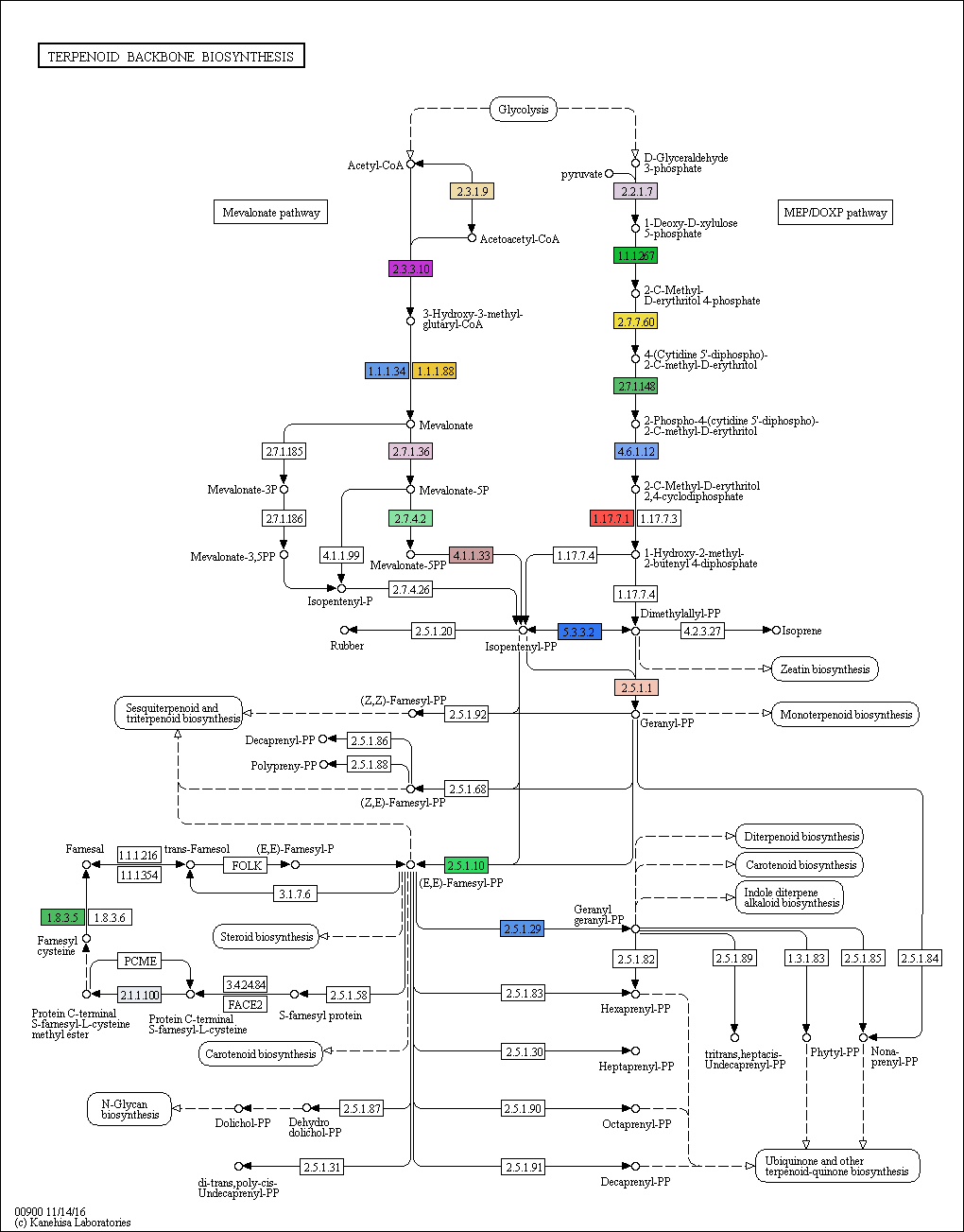

Supplement: Supplementary file 1 [file marinedrugs-16-00207-s001.zip › Supplementary Figures and Tables/Supplementary File 1 _ KEGG pathways/map00900 (Terpenoid backbone biosynthesis) [19 enz found].png]

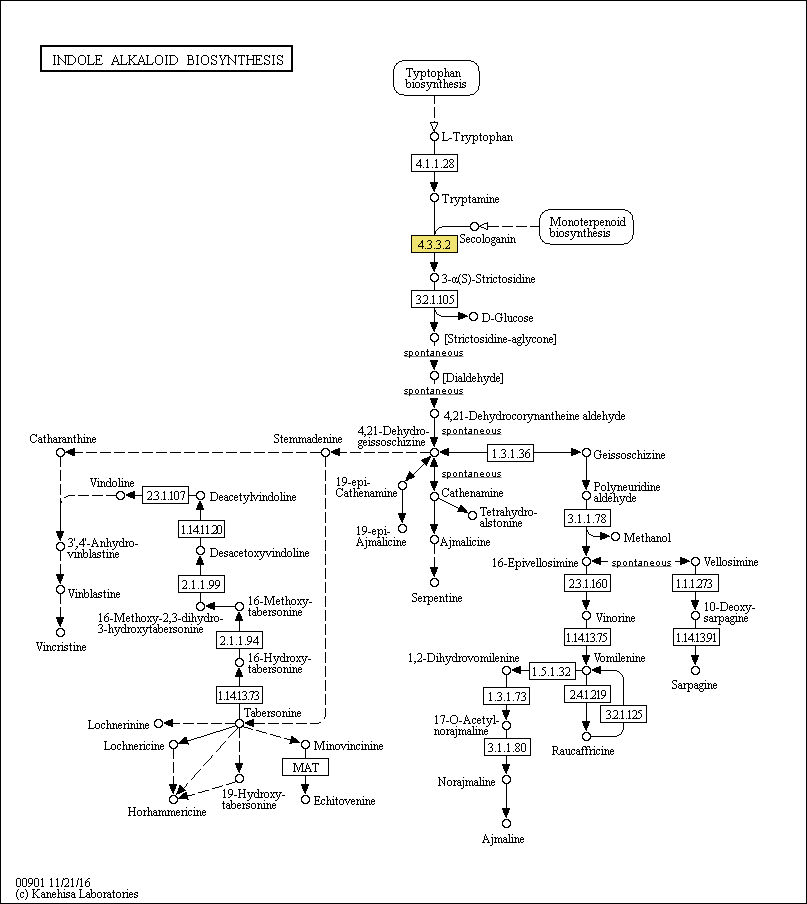

Supplement: Supplementary file 1 [file marinedrugs-16-00207-s001.zip › Supplementary Figures and Tables/Supplementary File 1 _ KEGG pathways/map00901 (Indole alkaloid biosynthesis) [1 enz found].png]

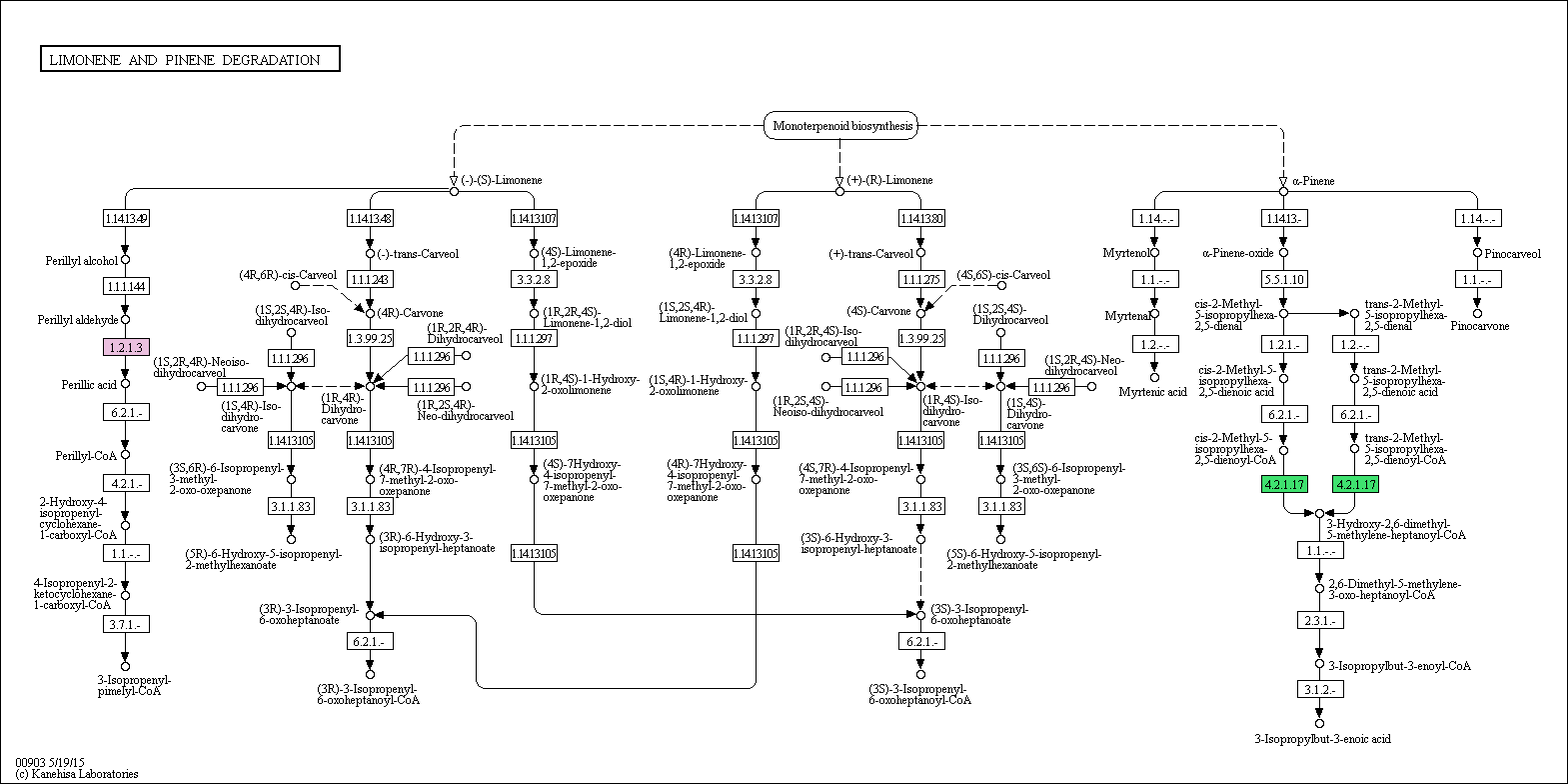

Supplement: Supplementary file 1 [file marinedrugs-16-00207-s001.zip › Supplementary Figures and Tables/Supplementary File 1 _ KEGG pathways/map00903 (Limonene and pinene degradation) [2 enz found].png]

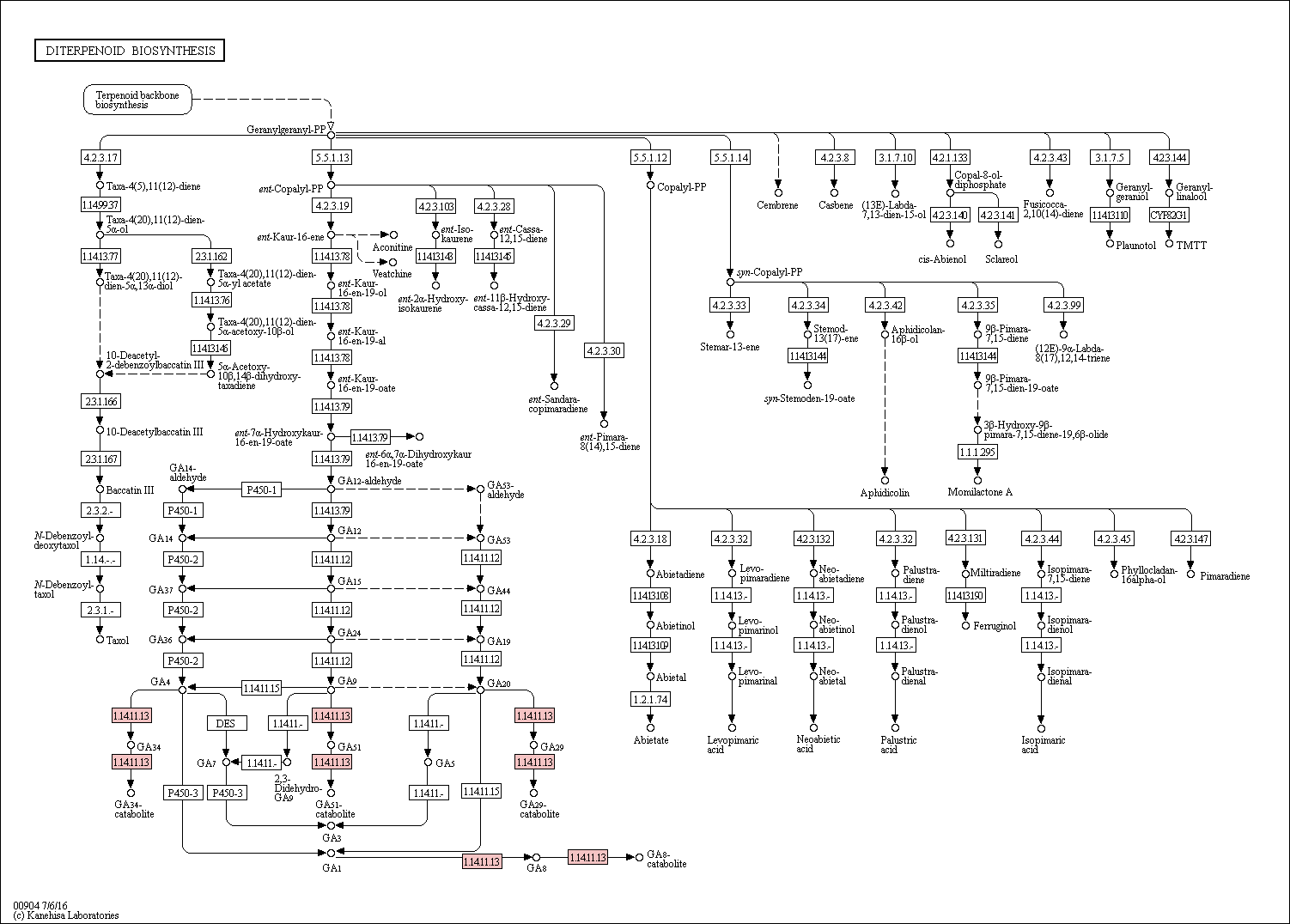

Supplement: Supplementary file 1 [file marinedrugs-16-00207-s001.zip › Supplementary Figures and Tables/Supplementary File 1 _ KEGG pathways/map00904 (Diterpenoid biosynthesis) [1 enz found].png]

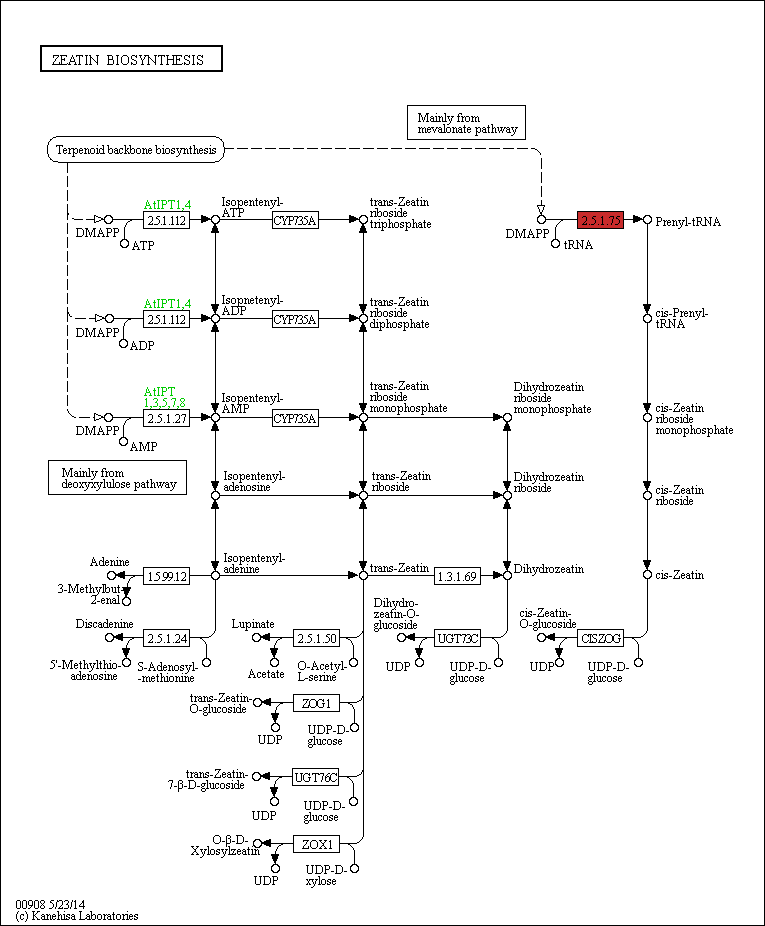

Supplement: Supplementary file 1 [file marinedrugs-16-00207-s001.zip › Supplementary Figures and Tables/Supplementary File 1 _ KEGG pathways/map00908 (Zeatin biosynthesis) [1 enz found].png]

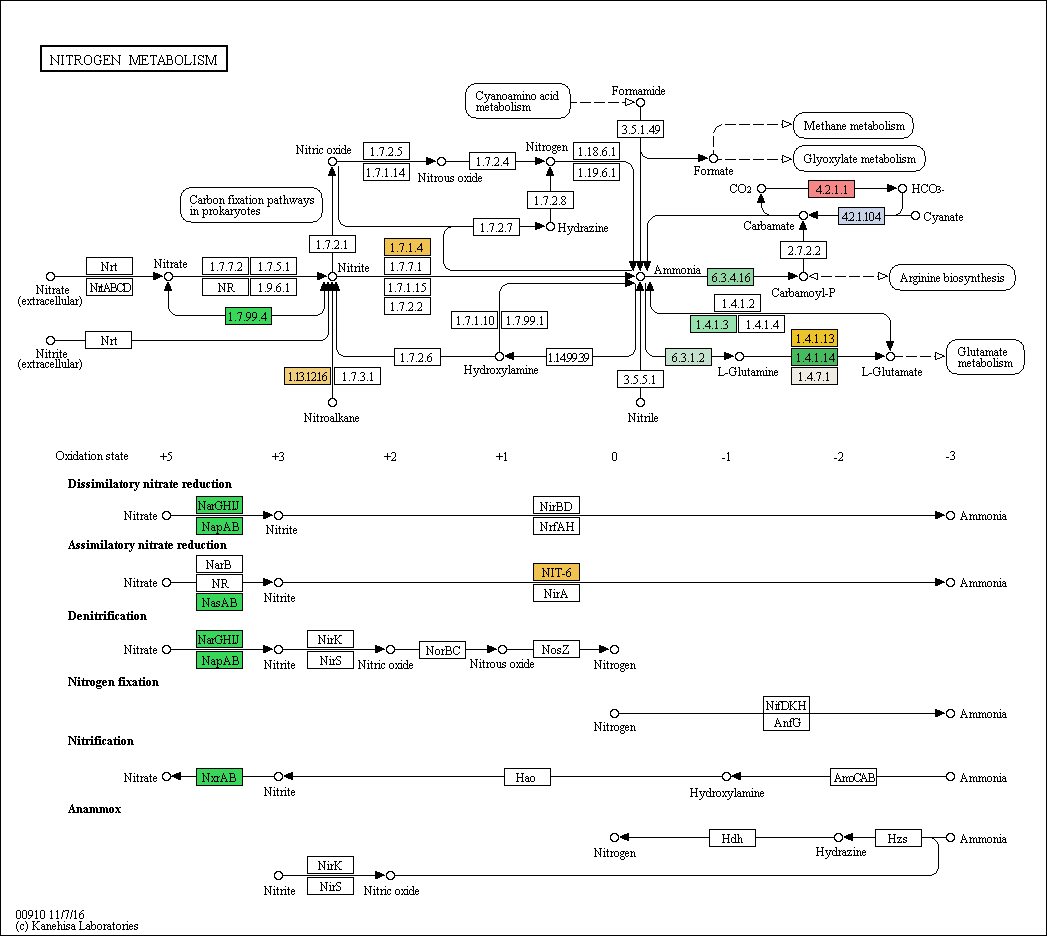

Supplement: Supplementary file 1 [file marinedrugs-16-00207-s001.zip › Supplementary Figures and Tables/Supplementary File 1 _ KEGG pathways/map00910 (Nitrogen metabolism) [11 enz found].png]

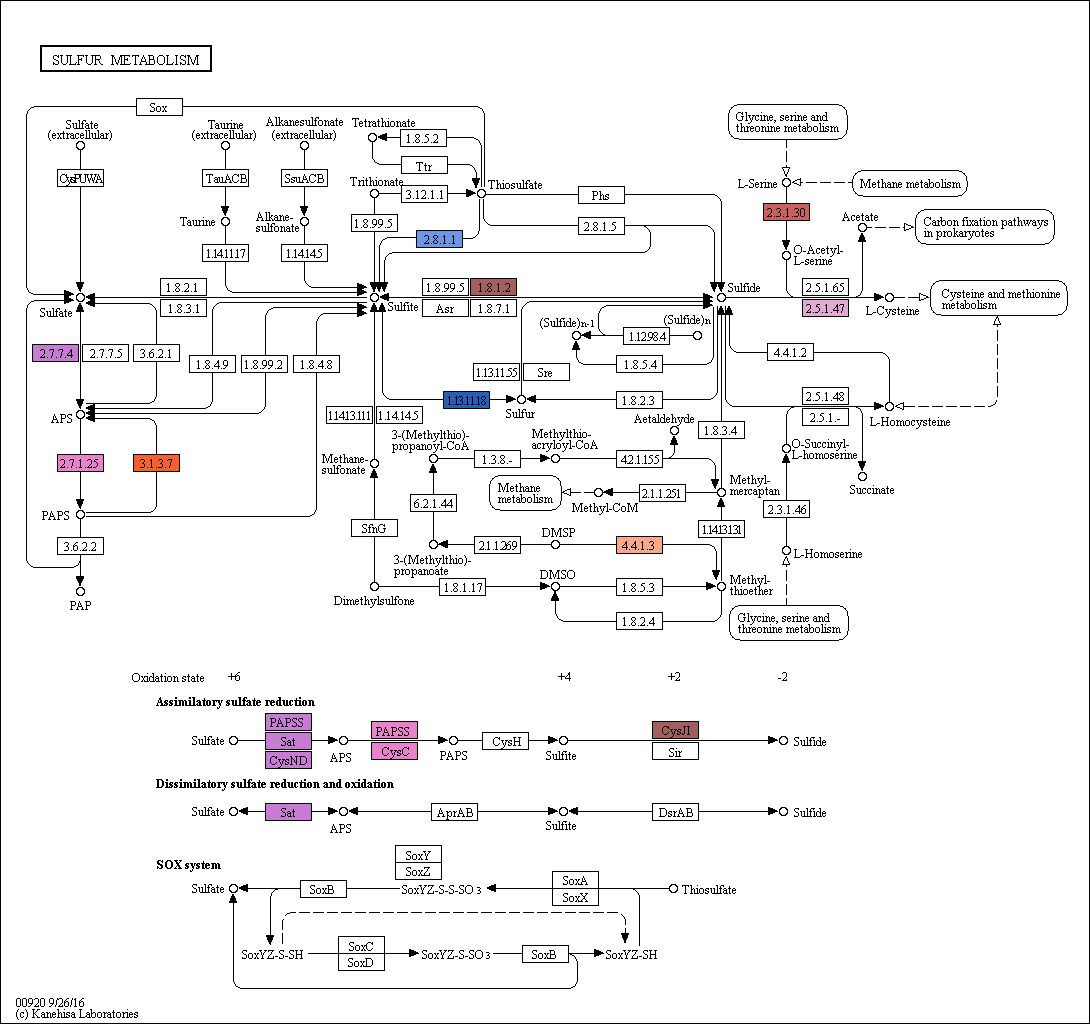

Supplement: Supplementary file 1 [file marinedrugs-16-00207-s001.zip › Supplementary Figures and Tables/Supplementary File 1 _ KEGG pathways/map00920 (Sulfur metabolism) [9 enz found].png]

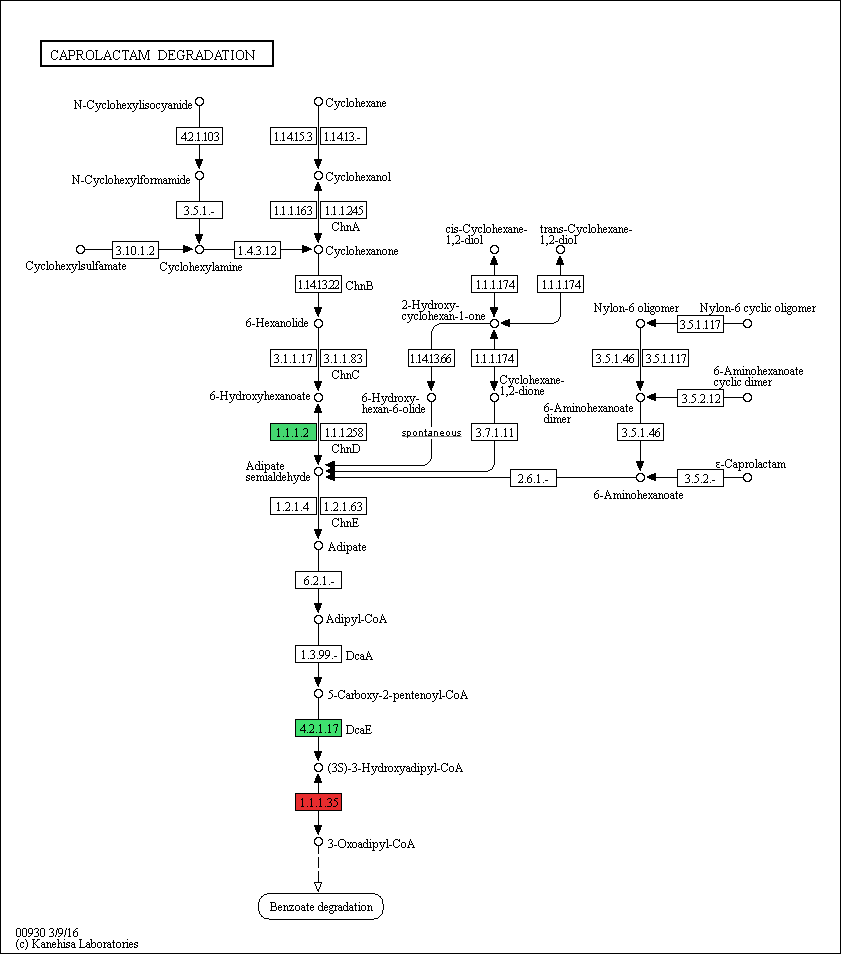

Supplement: Supplementary file 1 [file marinedrugs-16-00207-s001.zip › Supplementary Figures and Tables/Supplementary File 1 _ KEGG pathways/map00930 (Caprolactam degradation) [3 enz found].png]

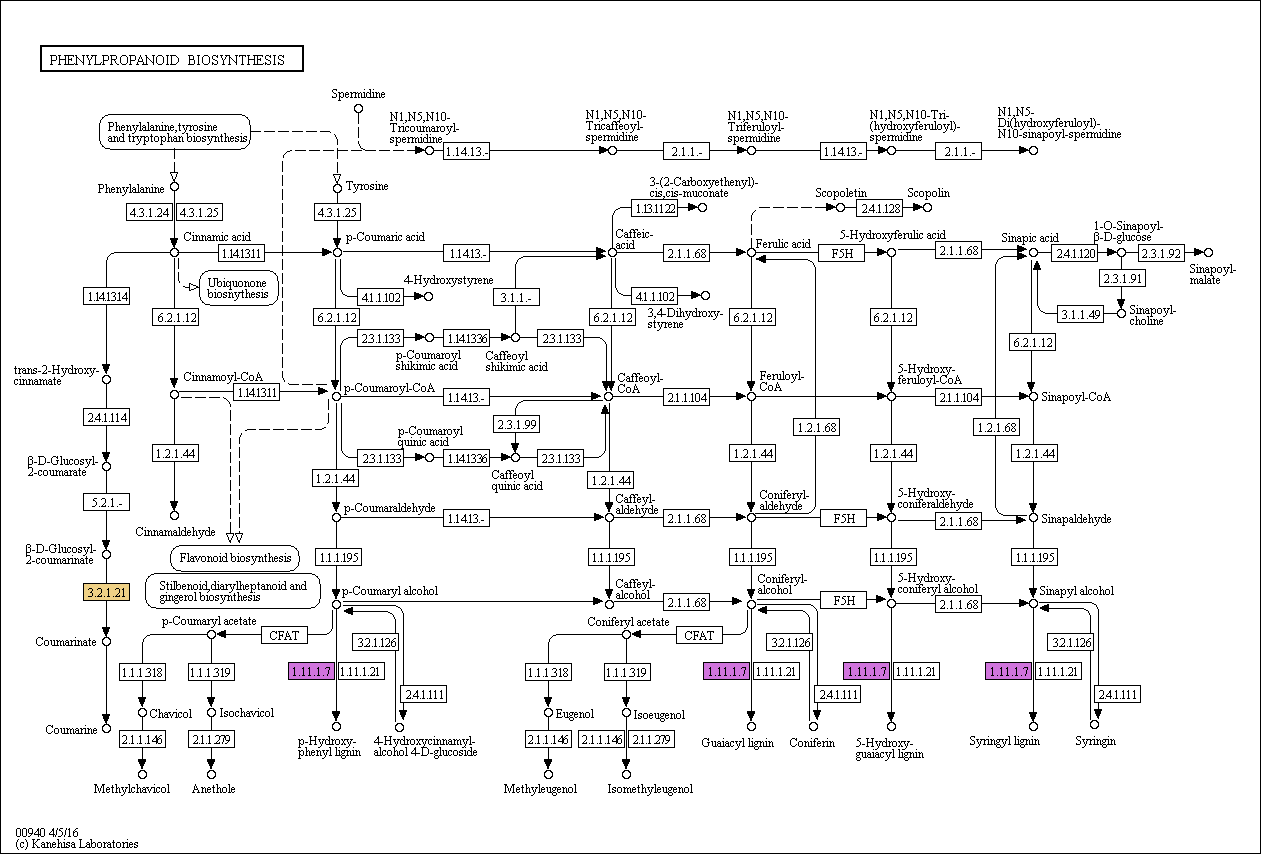

Supplement: Supplementary file 1 [file marinedrugs-16-00207-s001.zip › Supplementary Figures and Tables/Supplementary File 1 _ KEGG pathways/map00940 (Phenylpropanoid biosynthesis) [2 enz found].png]

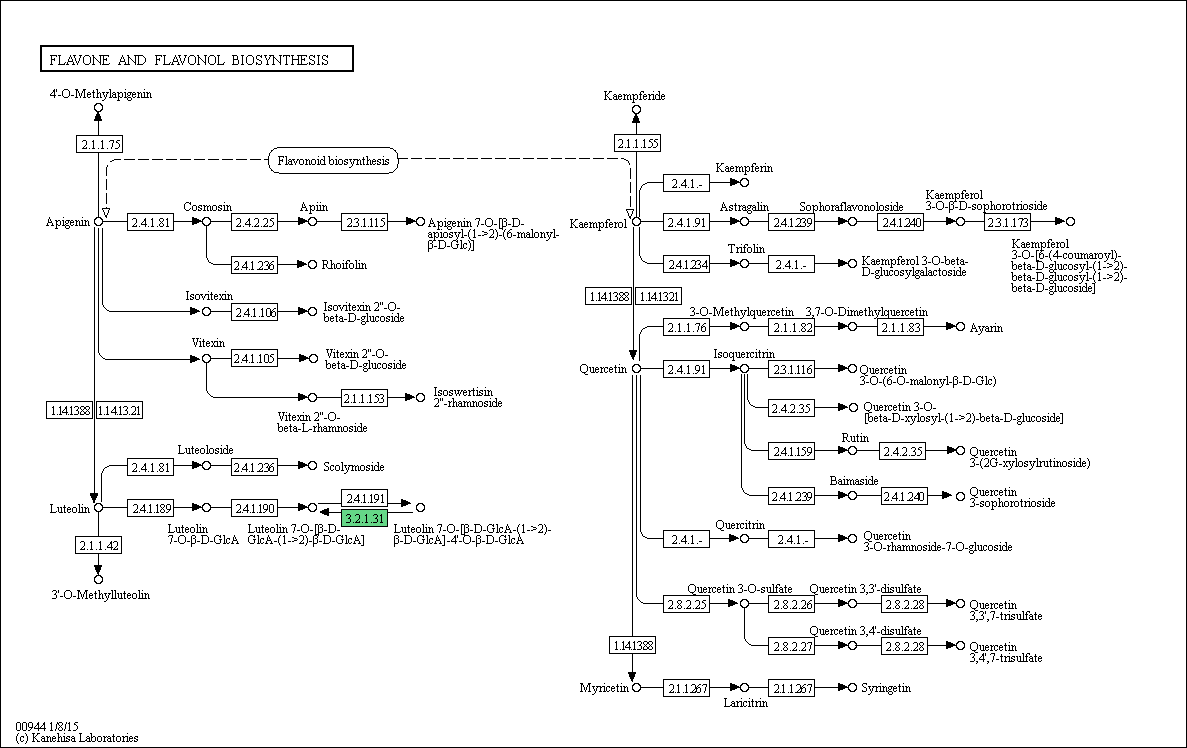

Supplement: Supplementary file 1 [file marinedrugs-16-00207-s001.zip › Supplementary Figures and Tables/Supplementary File 1 _ KEGG pathways/map00944 (Flavone and flavonol biosynthesis) [1 enz found].png]

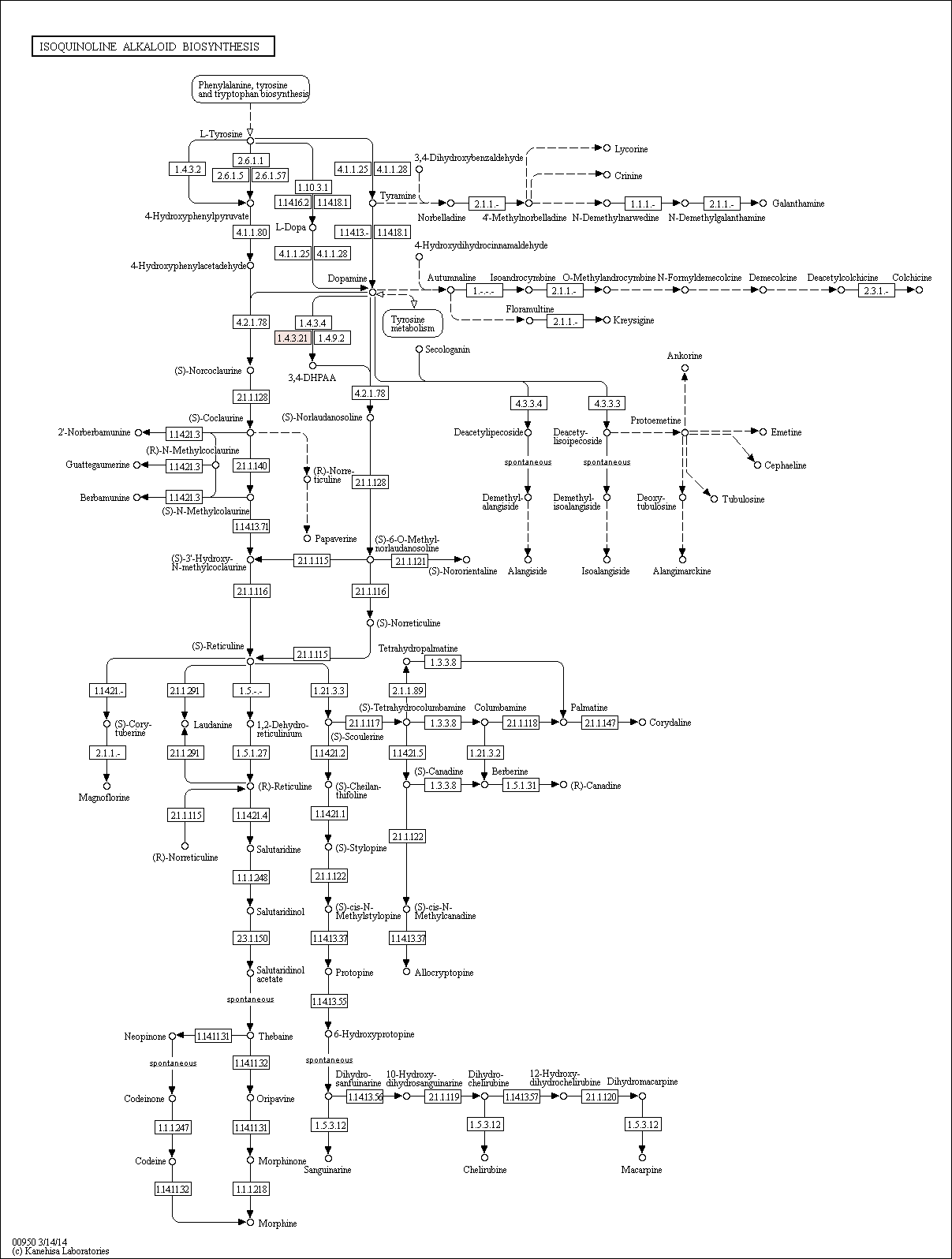

Supplement: Supplementary file 1 [file marinedrugs-16-00207-s001.zip › Supplementary Figures and Tables/Supplementary File 1 _ KEGG pathways/map00950 (Isoquinoline alkaloid biosynthesis) [1 enz found].png]

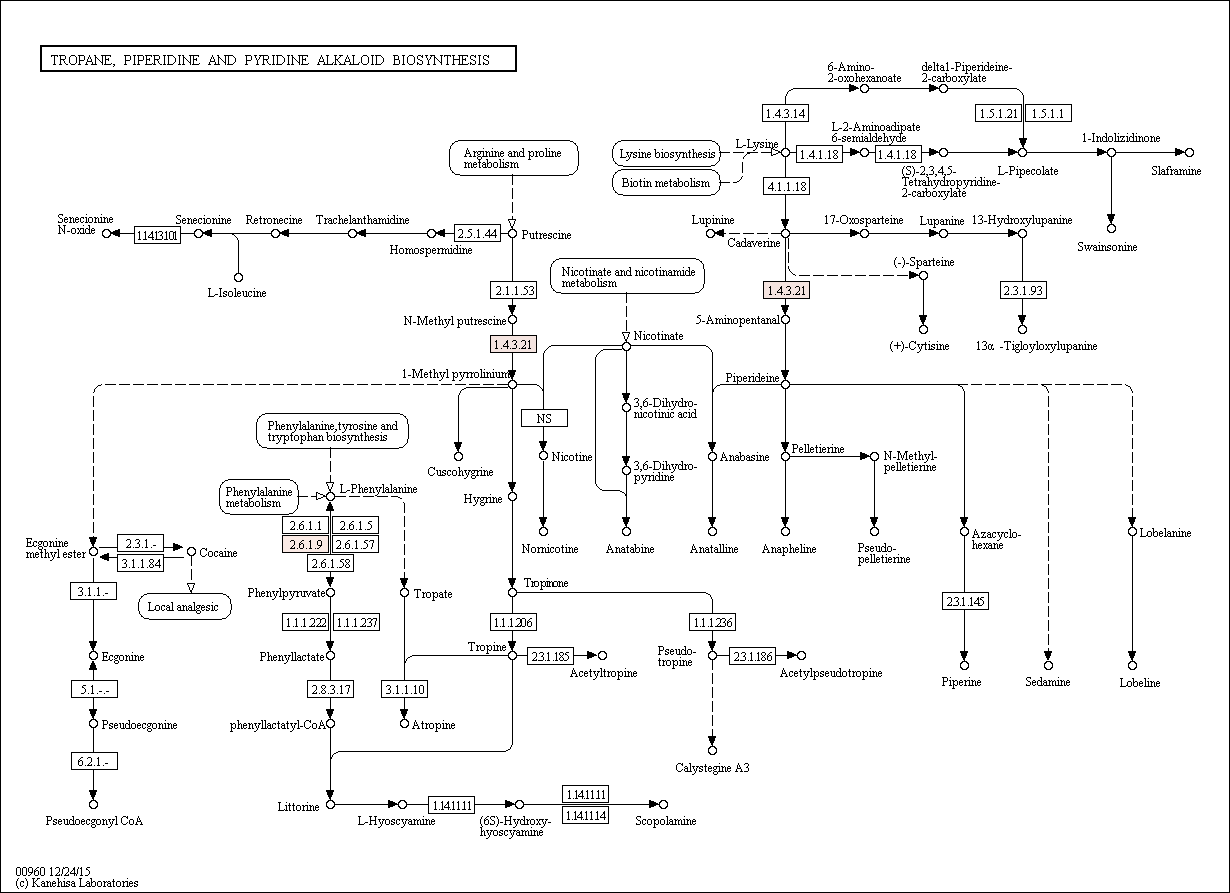

Supplement: Supplementary file 1 [file marinedrugs-16-00207-s001.zip › Supplementary Figures and Tables/Supplementary File 1 _ KEGG pathways/map00960 (Tropane, piperidine and pyridine alkaloid biosynthesis) [2 enz found].png]

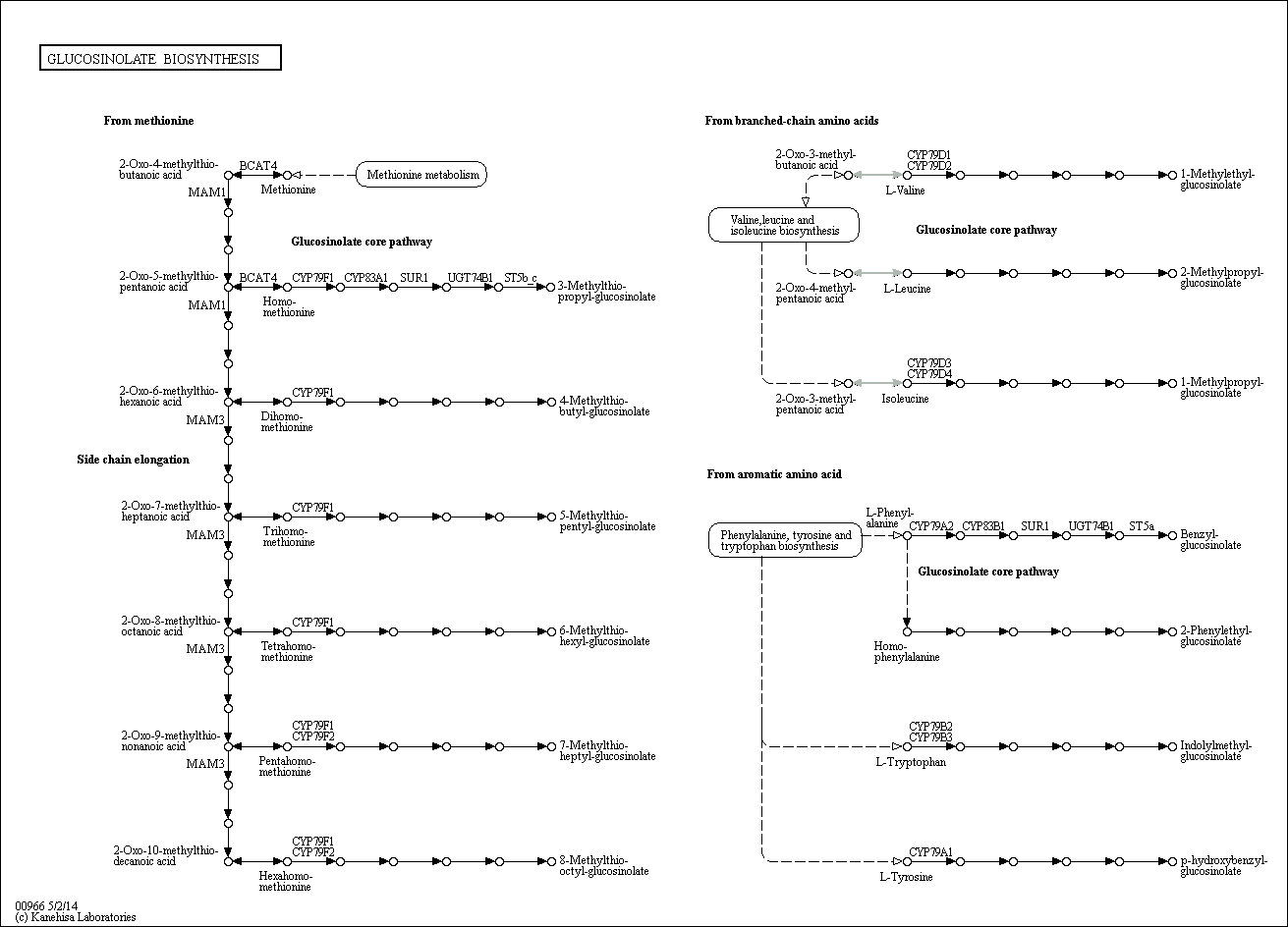

Supplement: Supplementary file 1 [file marinedrugs-16-00207-s001.zip › Supplementary Figures and Tables/Supplementary File 1 _ KEGG pathways/map00966 (Glucosinolate biosynthesis) [1 enz found].png]

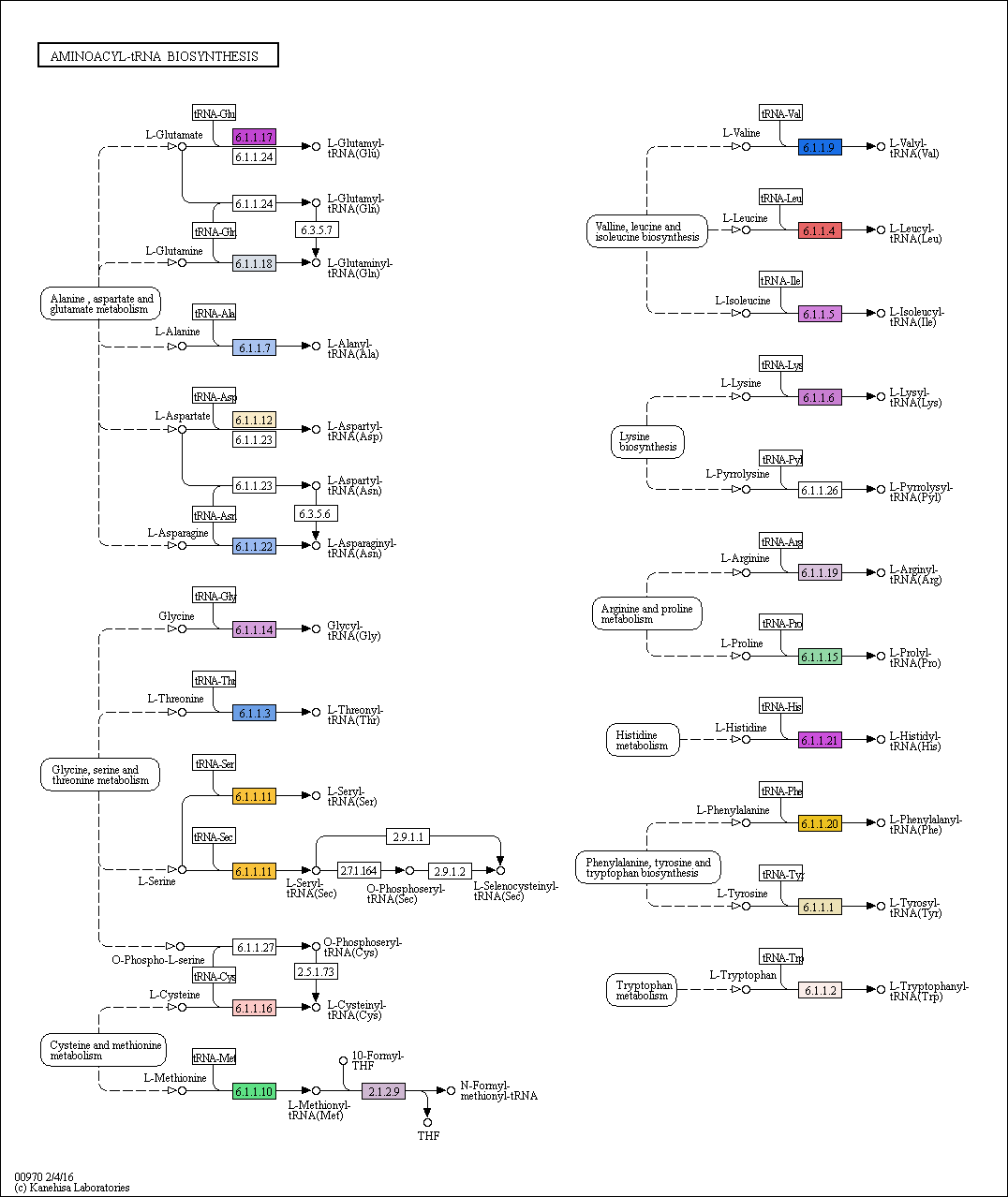

Supplement: Supplementary file 1 [file marinedrugs-16-00207-s001.zip › Supplementary Figures and Tables/Supplementary File 1 _ KEGG pathways/map00970 (Aminoacyl-tRNA biosynthesis) [21 enz found].png]

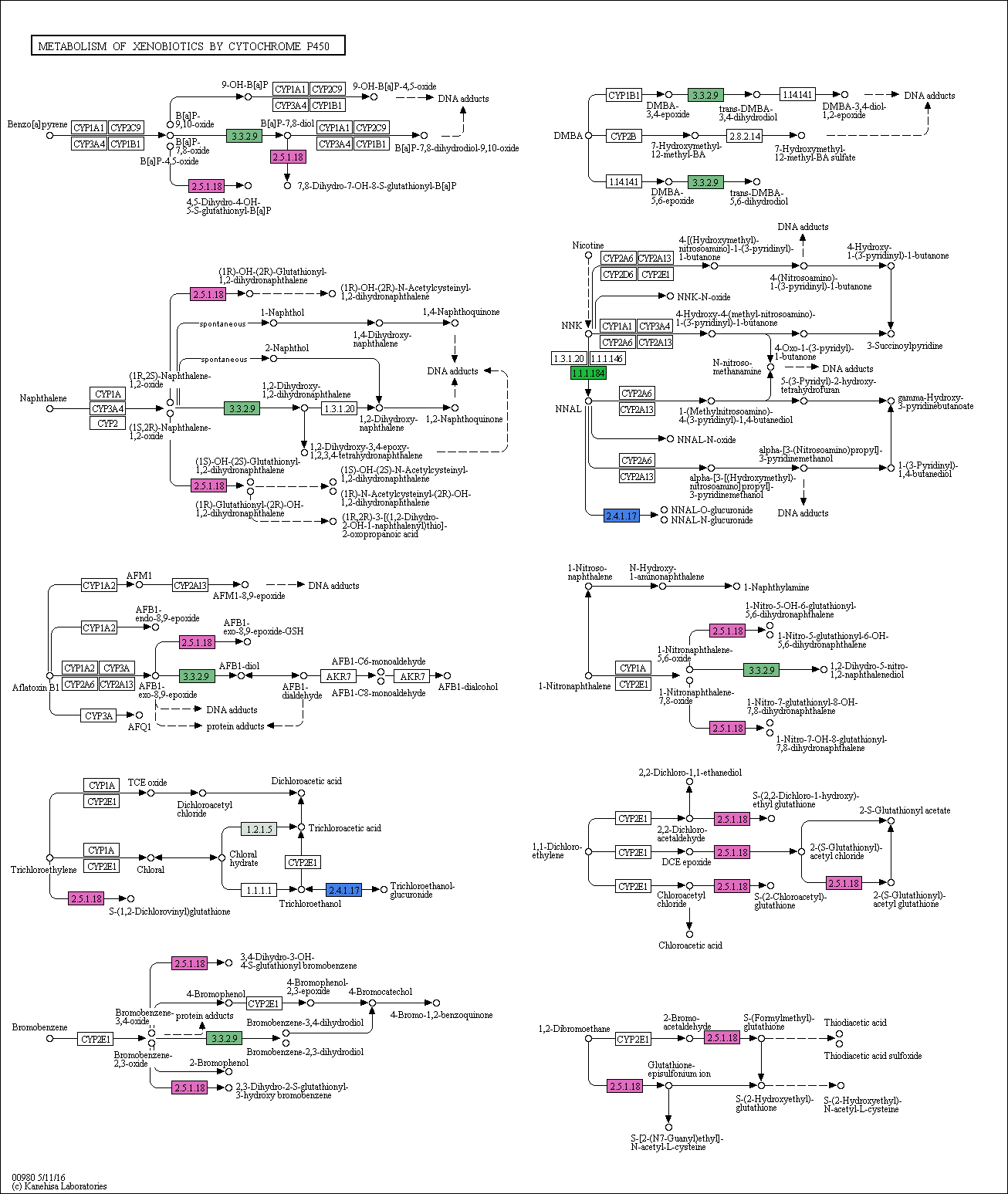

Supplement: Supplementary file 1 [file marinedrugs-16-00207-s001.zip › Supplementary Figures and Tables/Supplementary File 1 _ KEGG pathways/map00980 (Metabolism of xenobiotics by cytochrome P450) [5 enz found].png]

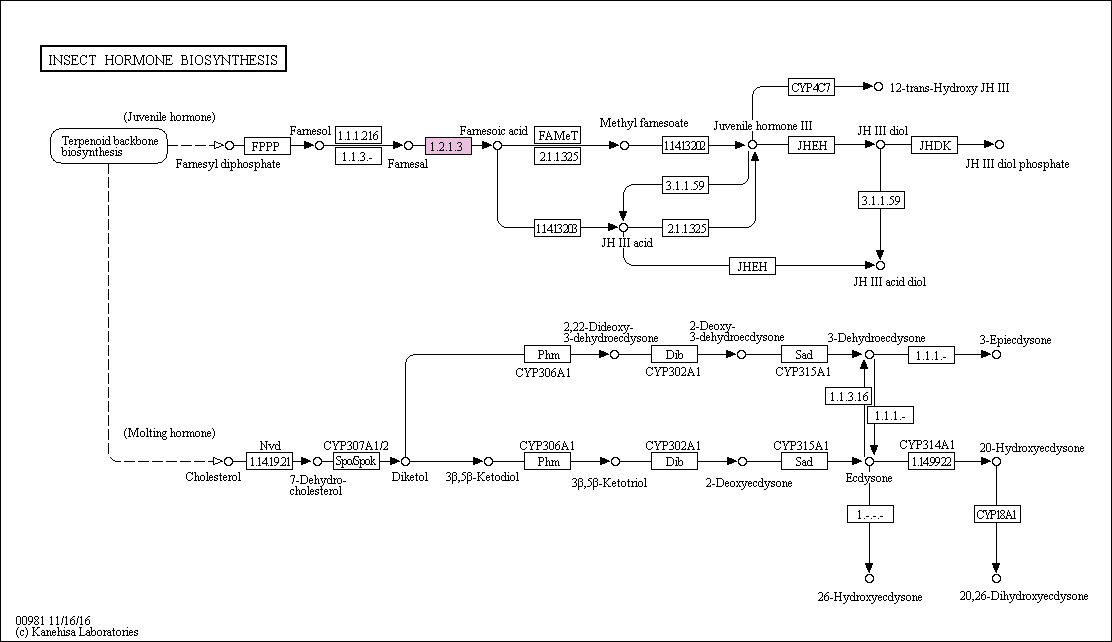

Supplement: Supplementary file 1 [file marinedrugs-16-00207-s001.zip › Supplementary Figures and Tables/Supplementary File 1 _ KEGG pathways/map00981 (Insect hormone biosynthesis) [1 enz found].png]

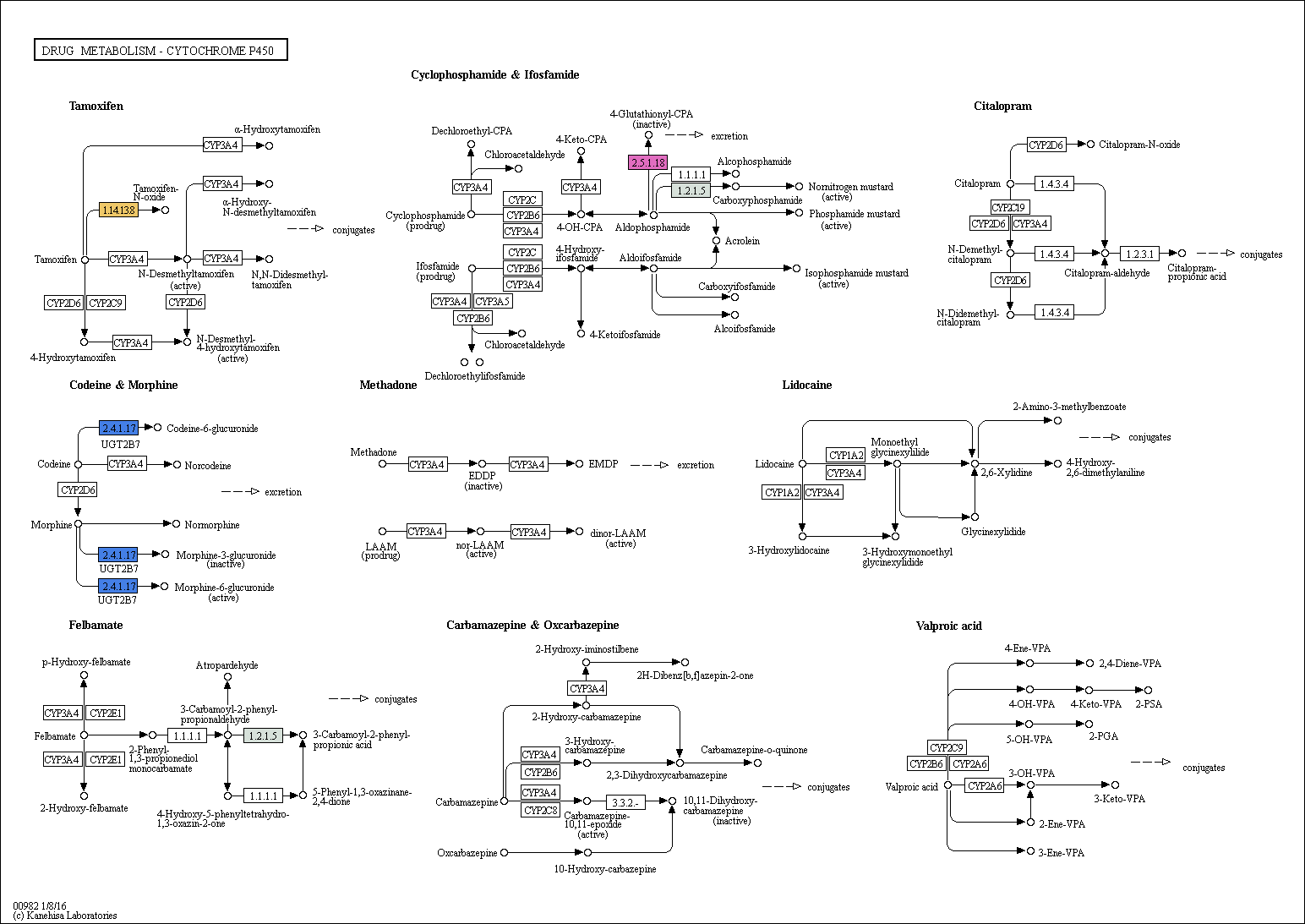

Supplement: Supplementary file 1 [file marinedrugs-16-00207-s001.zip › Supplementary Figures and Tables/Supplementary File 1 _ KEGG pathways/map00982 (Drug metabolism) [4 enz found].png]

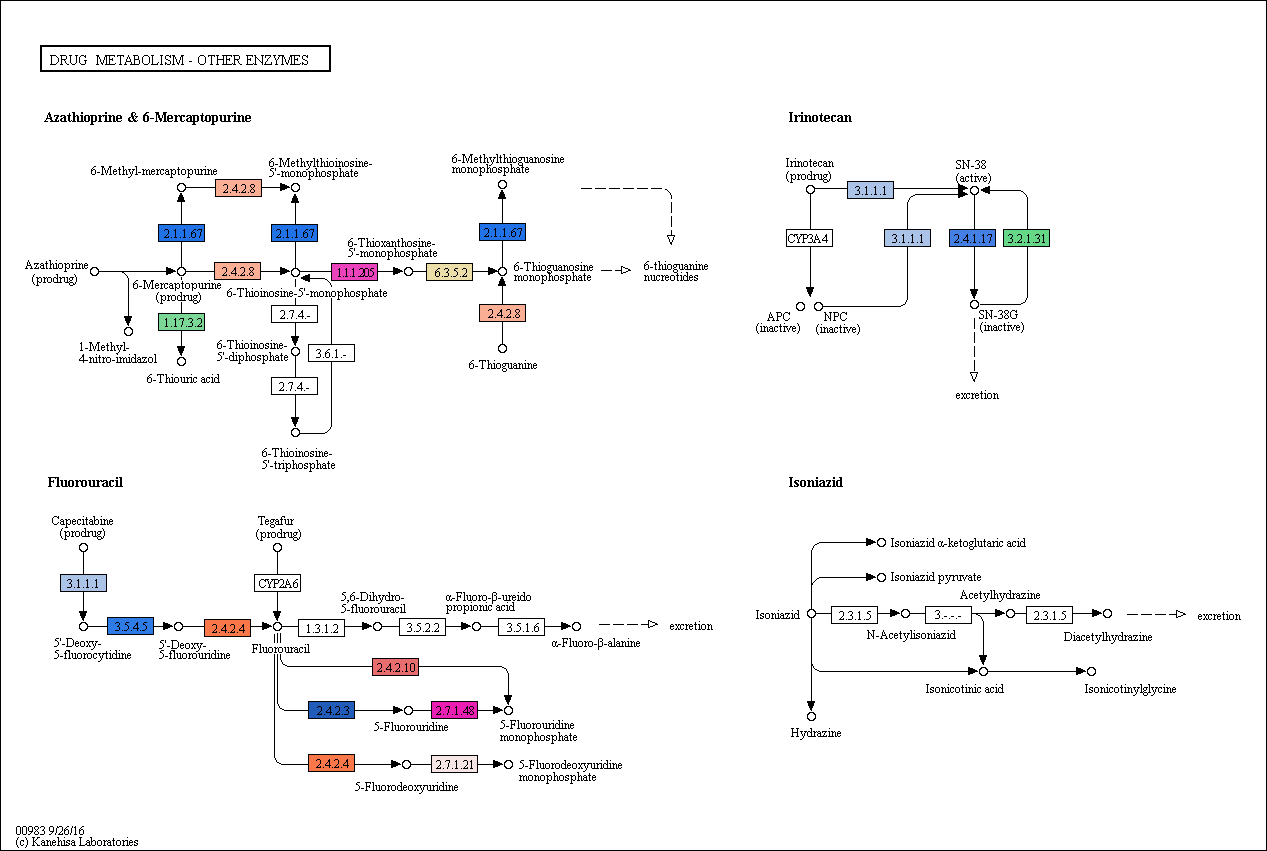

Supplement: Supplementary file 1 [file marinedrugs-16-00207-s001.zip › Supplementary Figures and Tables/Supplementary File 1 _ KEGG pathways/map00983 (Drug metabolism) [14 enz found].png]

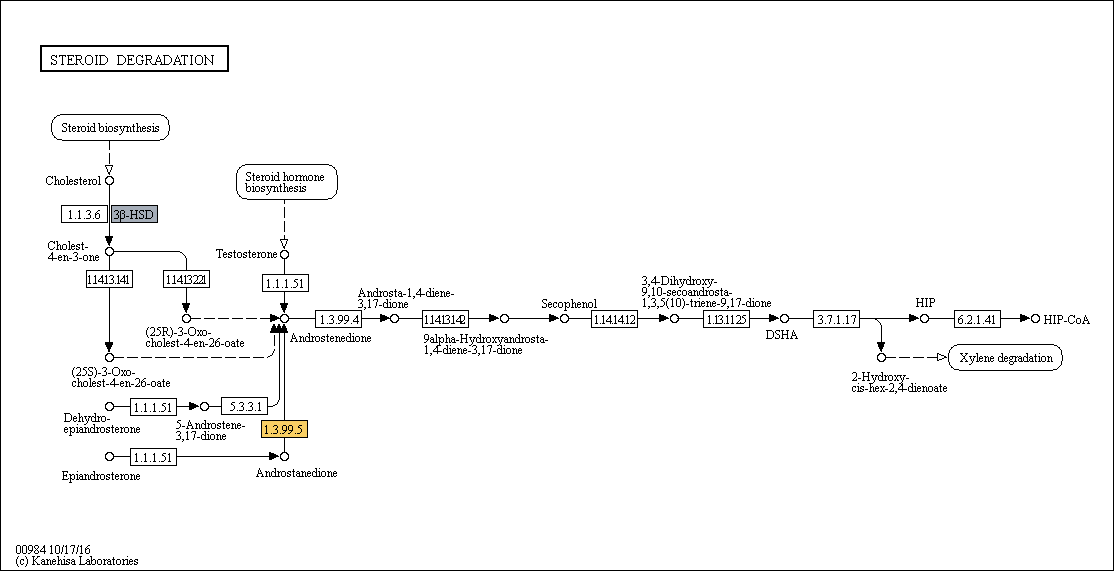

Supplement: Supplementary file 1 [file marinedrugs-16-00207-s001.zip › Supplementary Figures and Tables/Supplementary File 1 _ KEGG pathways/map00984 (Steroid degradation) [2 enz found].png]

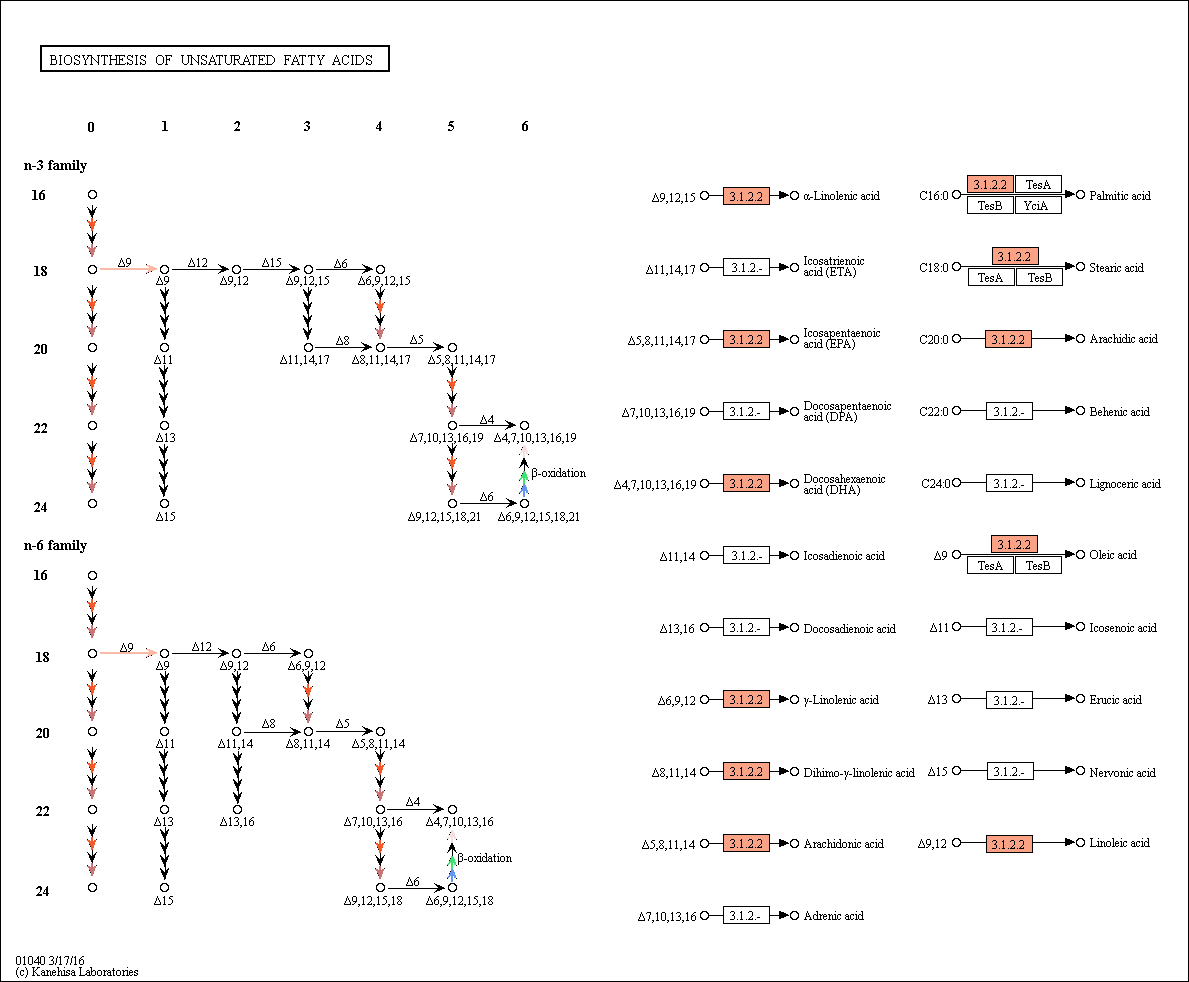

Supplement: Supplementary file 1 [file marinedrugs-16-00207-s001.zip › Supplementary Figures and Tables/Supplementary File 1 _ KEGG pathways/map01040 (Biosynthesis of unsaturated fatty acids) [7 enz found].png]

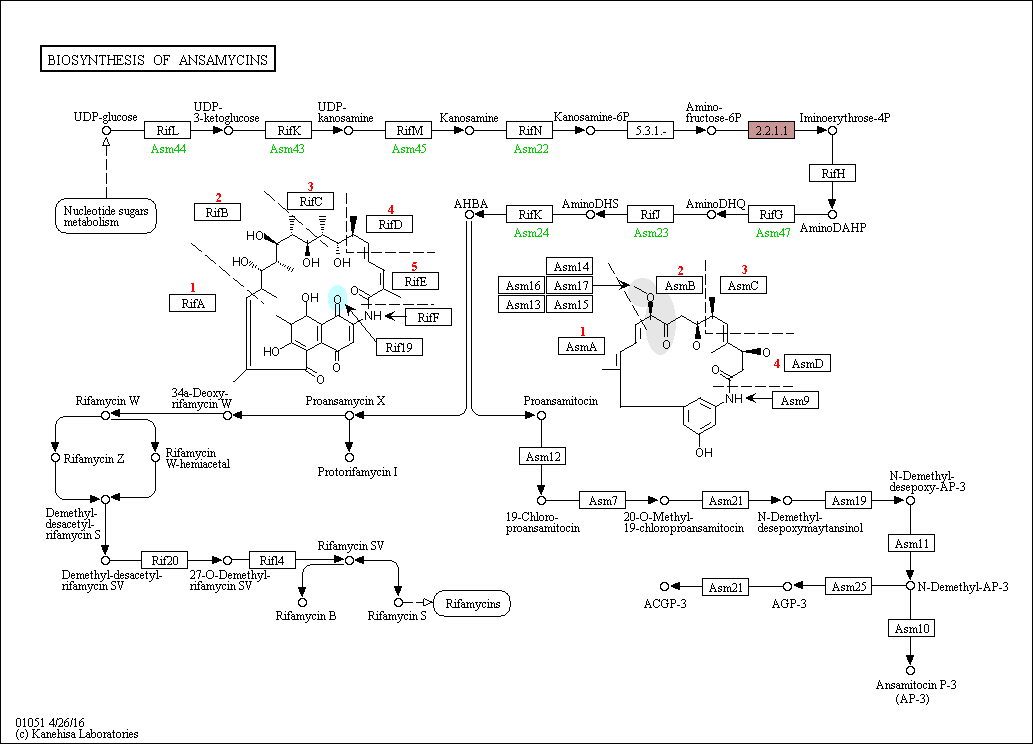

Supplement: Supplementary file 1 [file marinedrugs-16-00207-s001.zip › Supplementary Figures and Tables/Supplementary File 1 _ KEGG pathways/map01051 (Biosynthesis of ansamycins) [1 enz found].png]

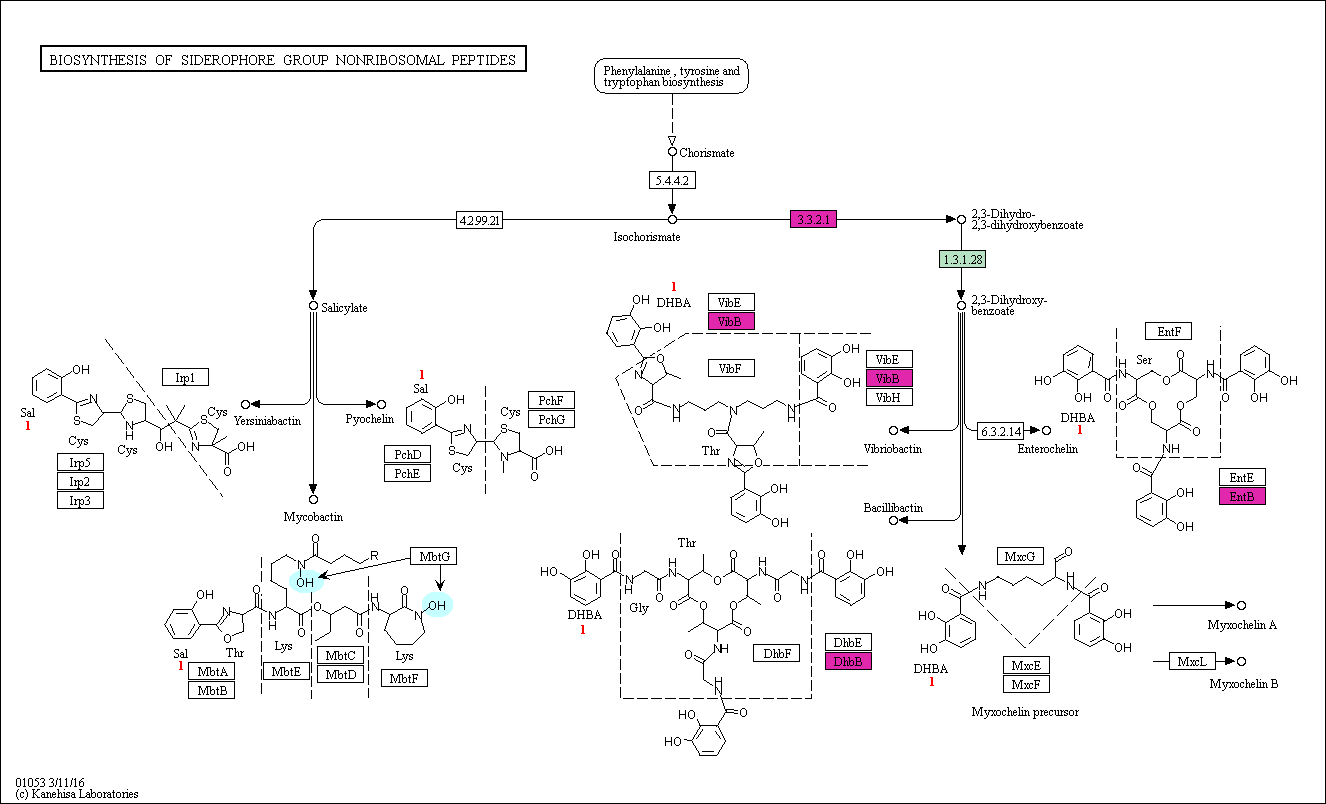

Supplement: Supplementary file 1 [file marinedrugs-16-00207-s001.zip › Supplementary Figures and Tables/Supplementary File 1 _ KEGG pathways/map01053 (Biosynthesis of siderophore group nonribosomal peptides) [2 enz found].png]

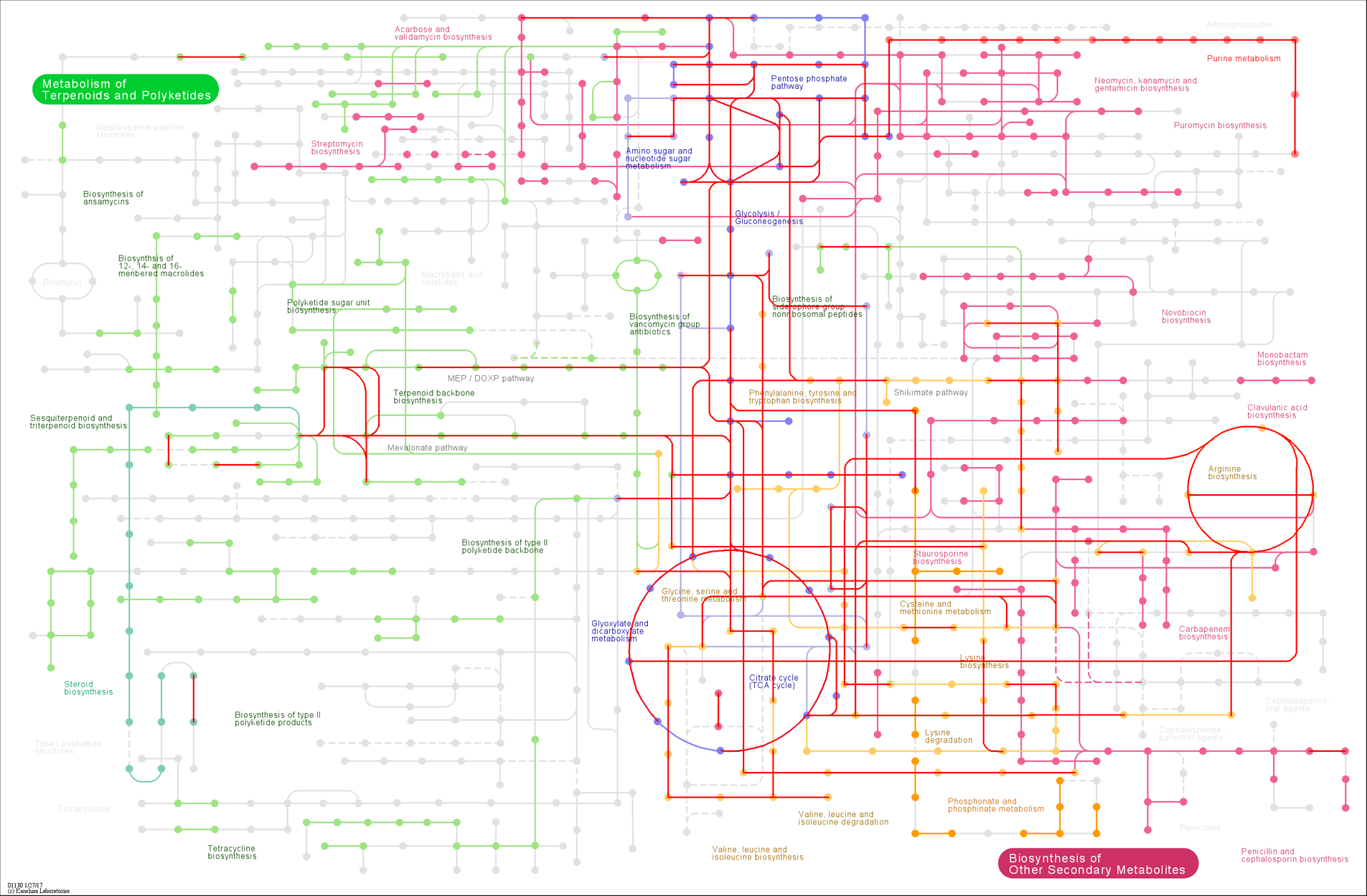

Supplement: Supplementary file 1 [file marinedrugs-16-00207-s001.zip › Supplementary Figures and Tables/Supplementary File 1 _ KEGG pathways/map01130 (Biosynthesis of antibiotics) [147 enz found].png]

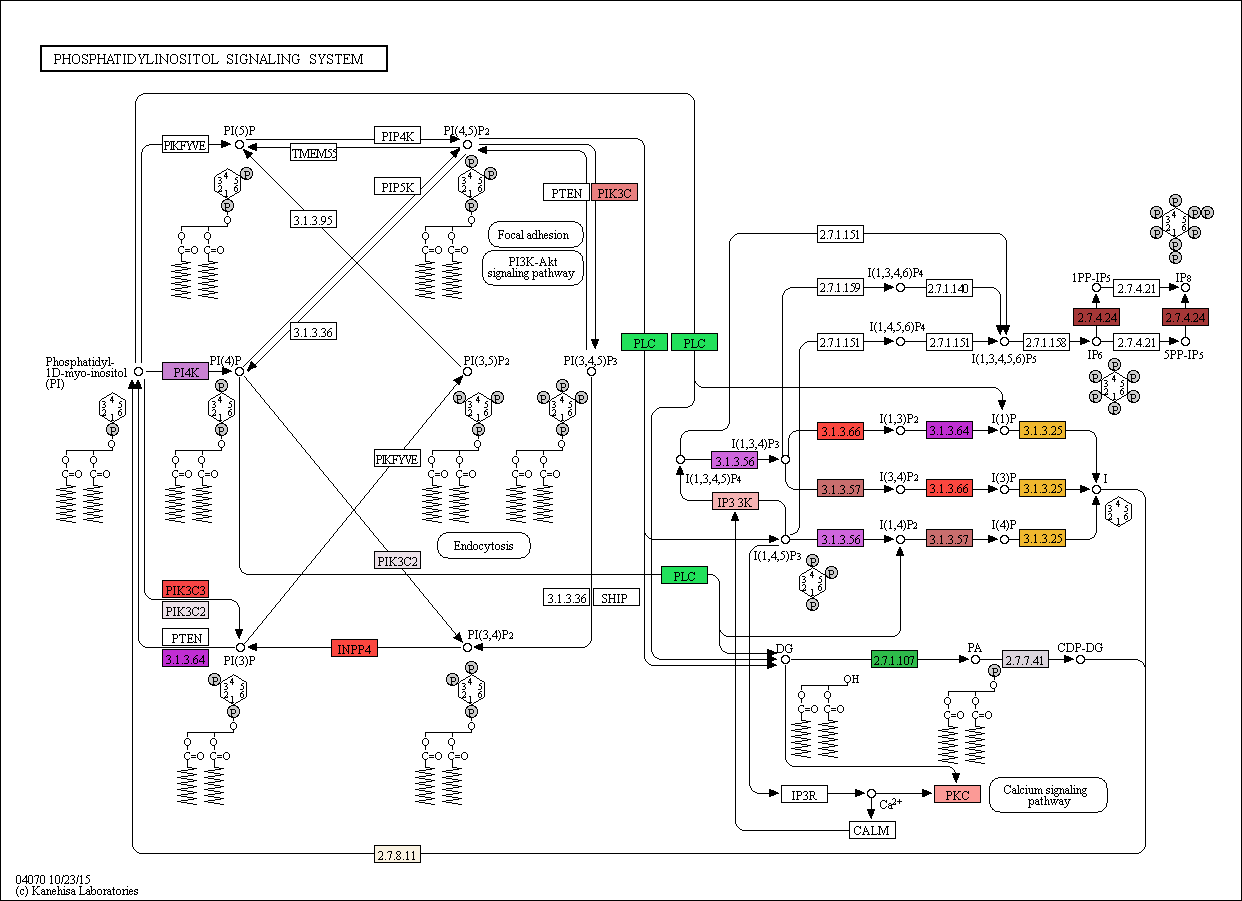

Supplement: Supplementary file 1 [file marinedrugs-16-00207-s001.zip › Supplementary Figures and Tables/Supplementary File 1 _ KEGG pathways/map04070 (Phosphatidylinositol signaling system) [17 enz found].png]

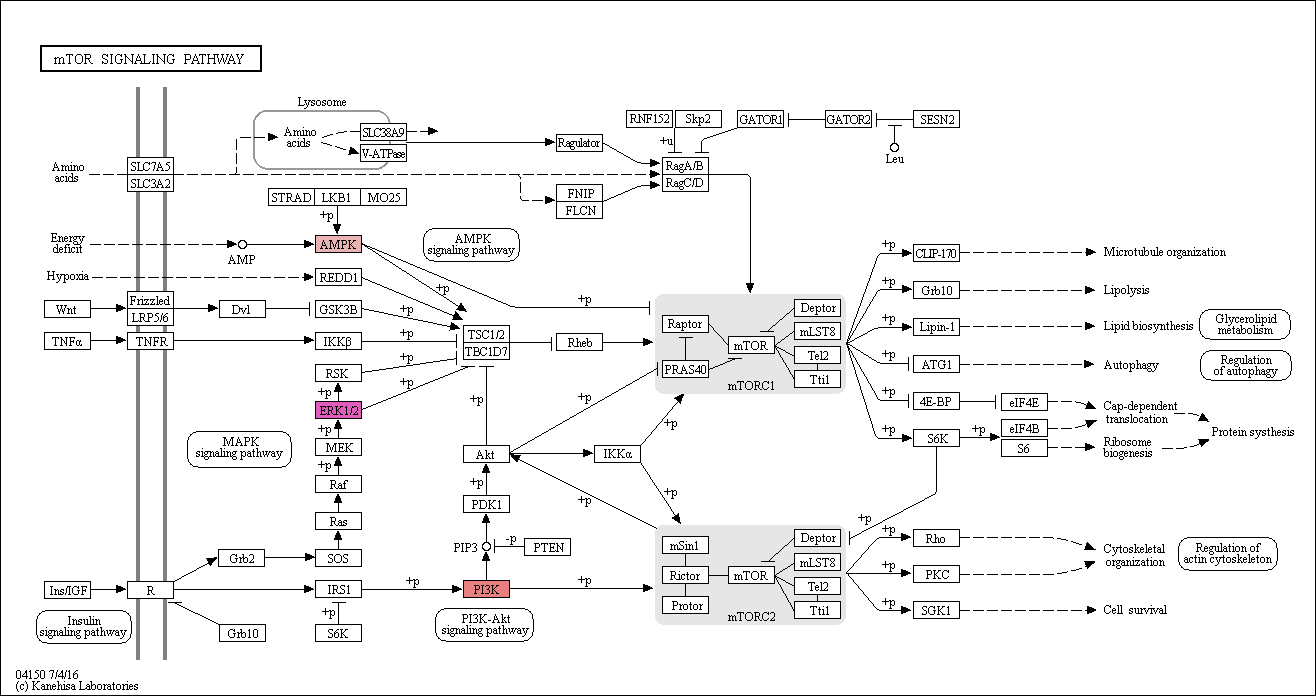

Supplement: Supplementary file 1 [file marinedrugs-16-00207-s001.zip › Supplementary Figures and Tables/Supplementary File 1 _ KEGG pathways/map04150 (mTOR signaling pathway) [3 enz found].png]

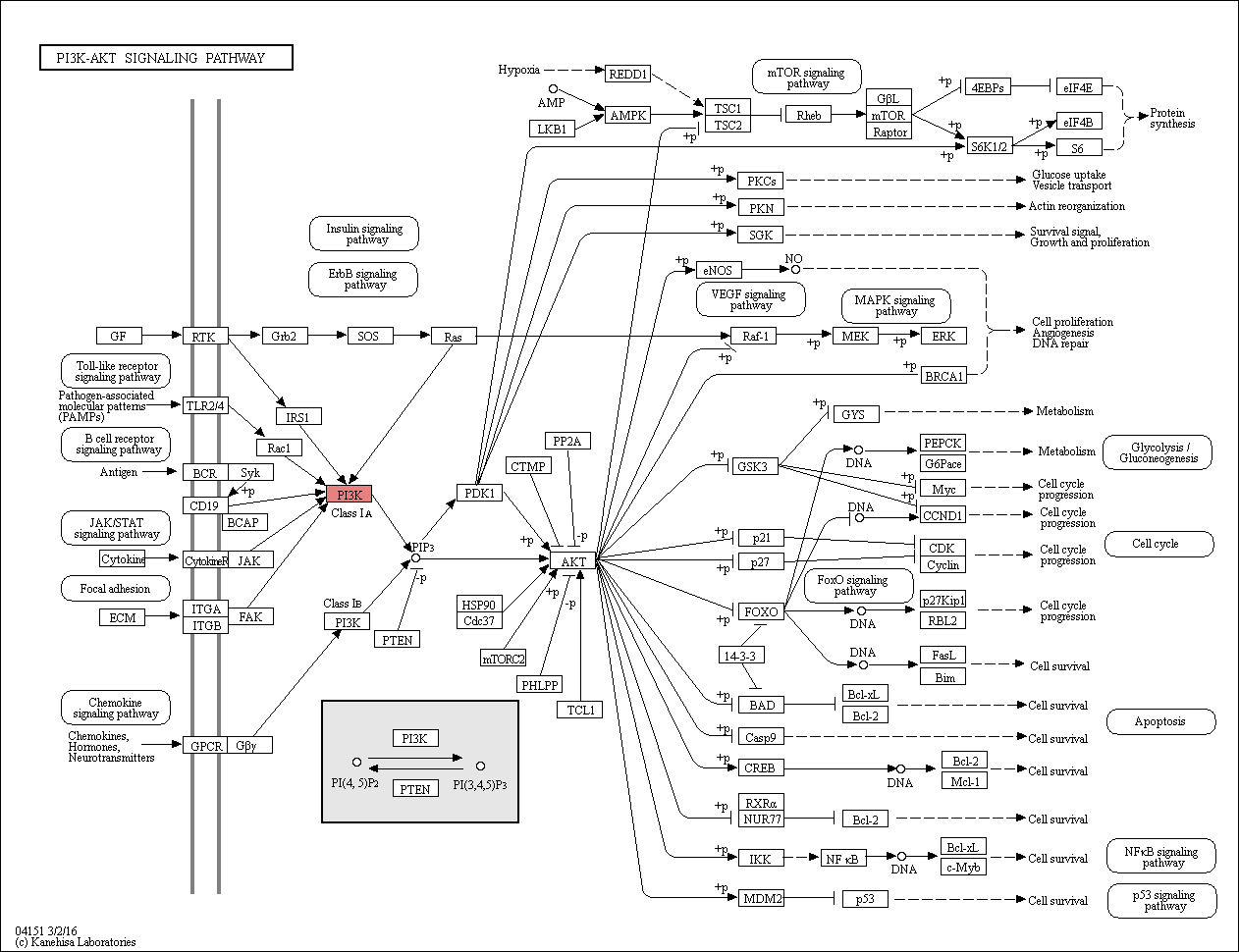

Supplement: Supplementary file 1 [file marinedrugs-16-00207-s001.zip › Supplementary Figures and Tables/Supplementary File 1 _ KEGG pathways/map04151 (PI3K-Akt signaling pathway) [1 enz found].png]

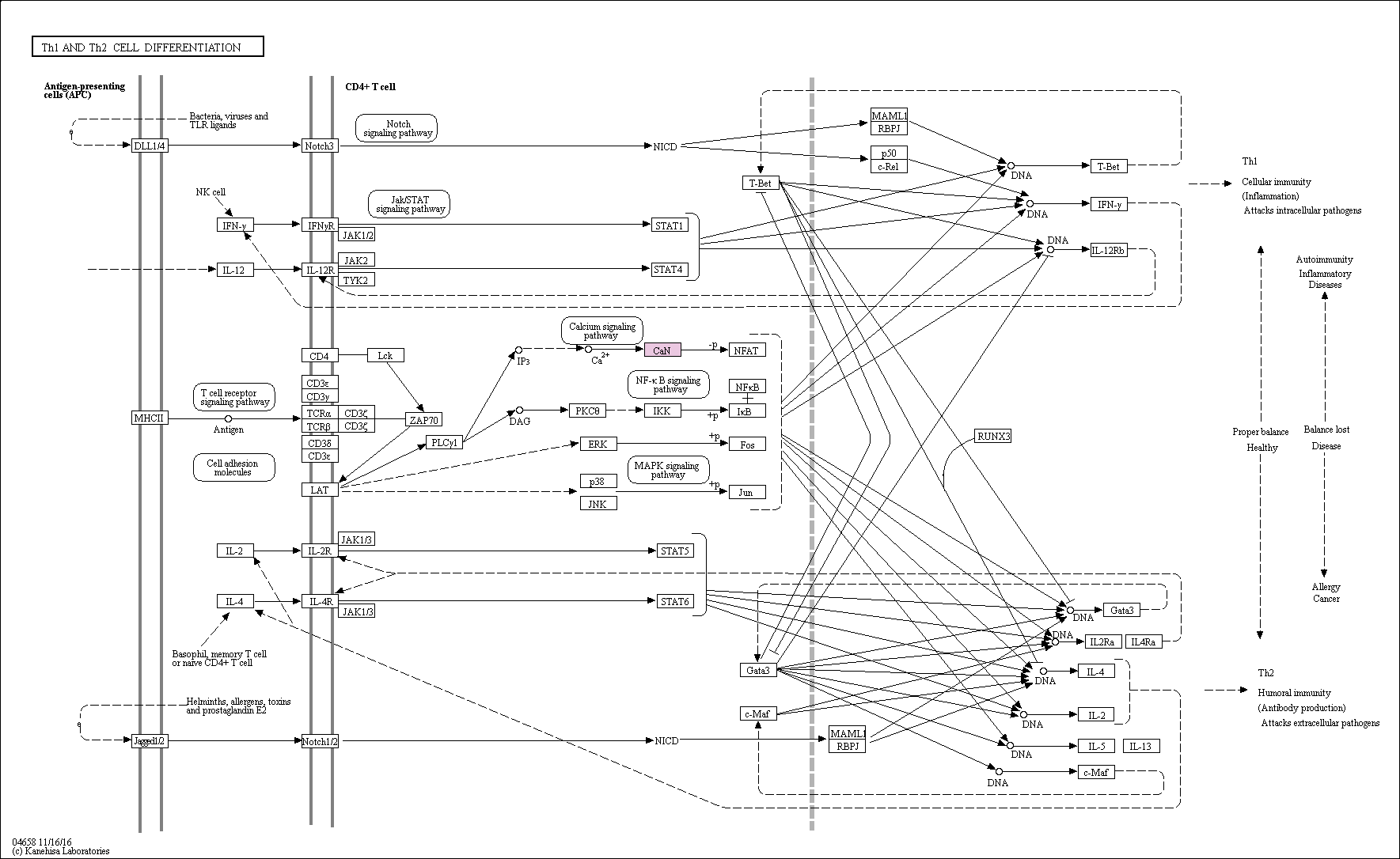

Supplement: Supplementary file 1 [file marinedrugs-16-00207-s001.zip › Supplementary Figures and Tables/Supplementary File 1 _ KEGG pathways/map04658 (Th1 and Th2 cell differentiation) [1 enz found].png]

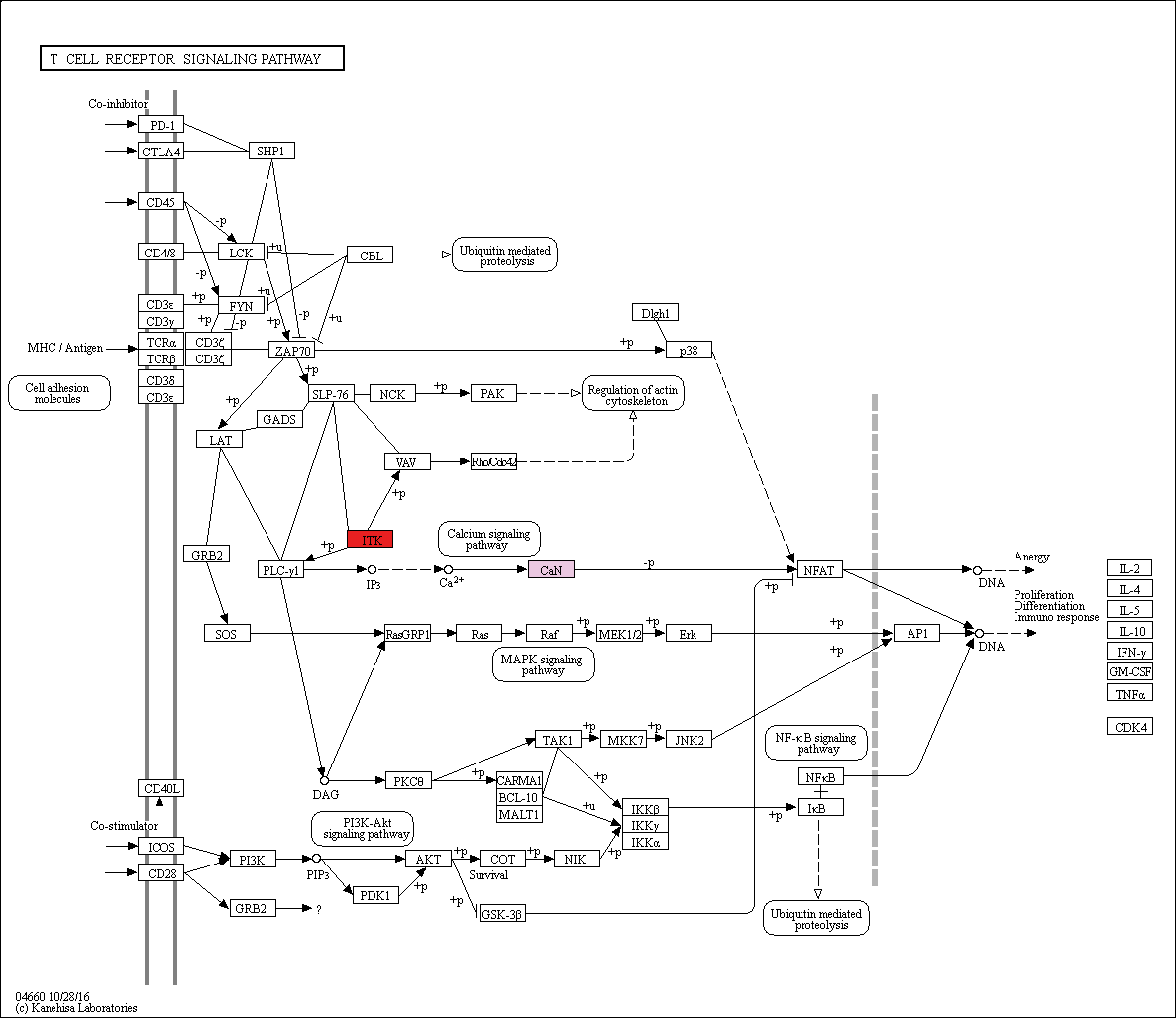

Supplement: Supplementary file 1 [file marinedrugs-16-00207-s001.zip › Supplementary Figures and Tables/Supplementary File 1 _ KEGG pathways/map04660 (T cell receptor signaling pathway) [2 enz found].png]
