# Supplementary figures and images for: The Holo-Transcriptome of the Zoantharian Protopalythoa variabilis (Cnidaria: Anthozoa): A Plentiful Source of Enzymes for Potential Application in Green Chemistry, Industrial and Pharmaceutical Biotechnology (part 1 of 2)
Source: Mar Drugs. 2018 Jun 13;16(6):207. doi: 10.3390/md16060207 (PMC6025448; doi:10.3390/md16060207)

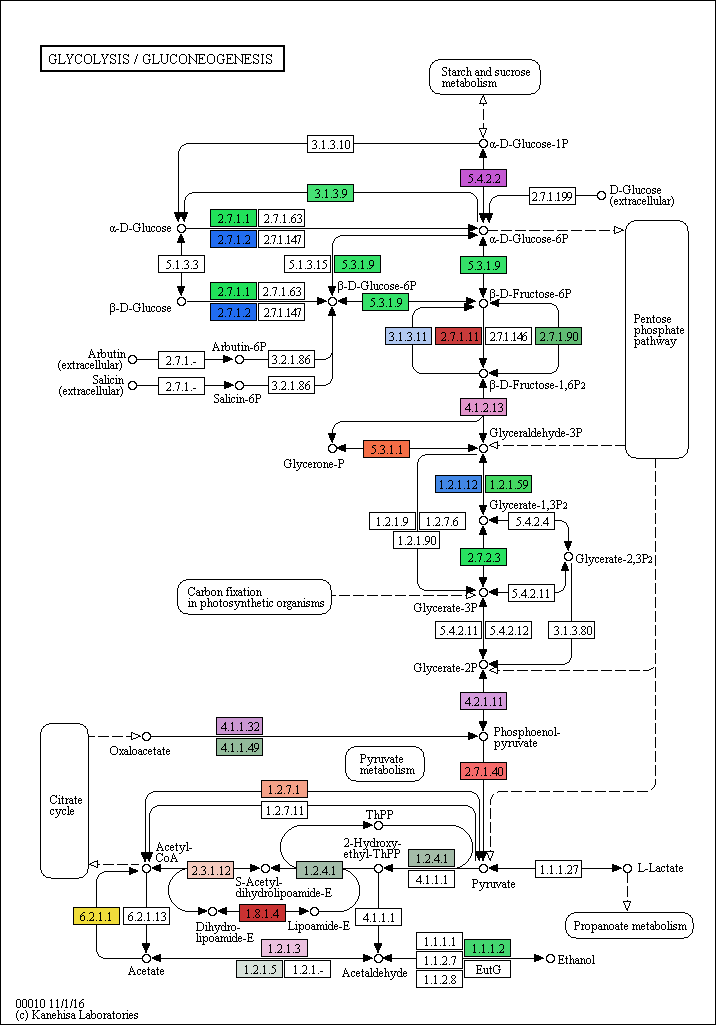

Supplement: Supplementary file 1 [file marinedrugs-16-00207-s001.zip › Supplementary Figures and Tables/Supplementary File 1 _ KEGG pathways/map00010 (Glycolysis-gluconeogenesis) [25 enz found].png]

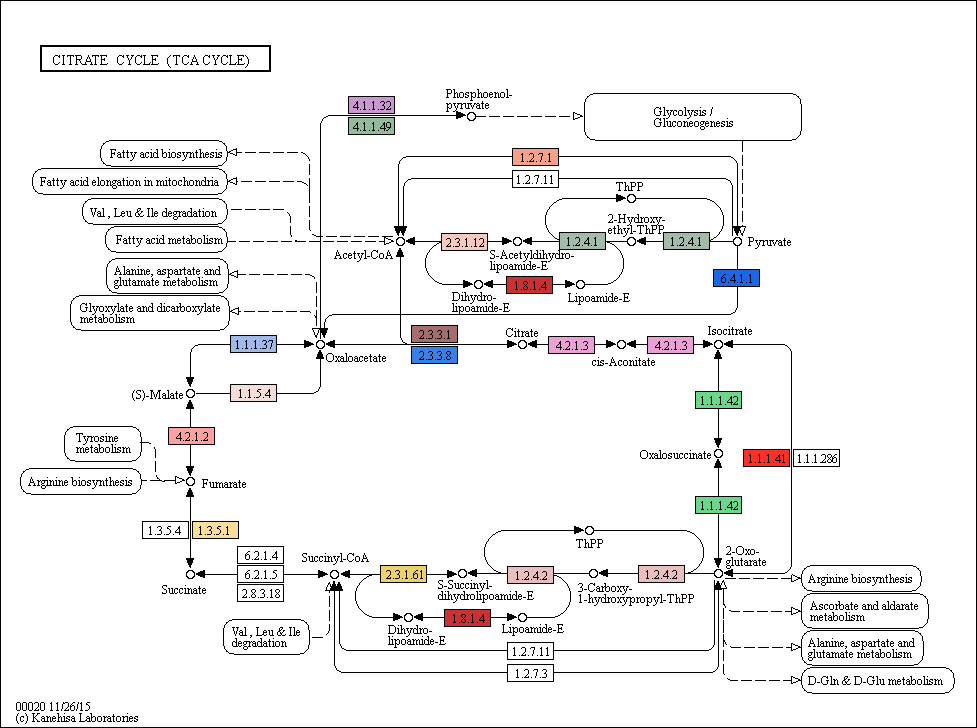

Supplement: Supplementary file 1 [file marinedrugs-16-00207-s001.zip › Supplementary Figures and Tables/Supplementary File 1 _ KEGG pathways/map00020 (Citrate cycle) [18 enz found].png]

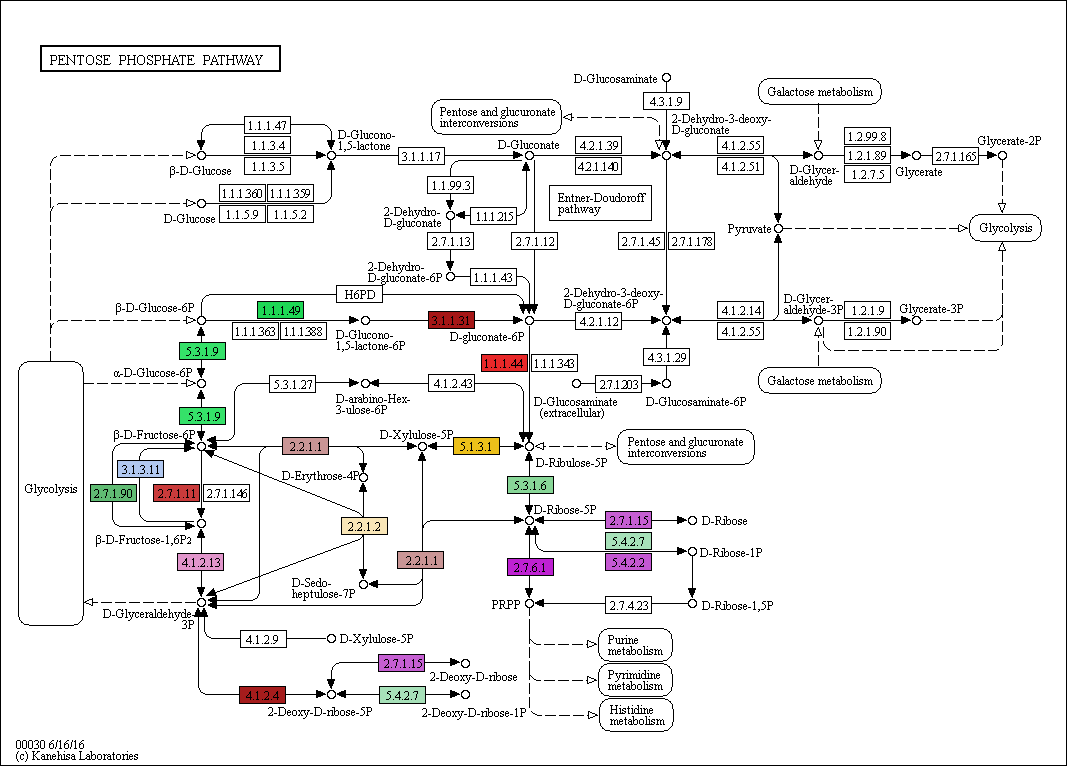

Supplement: Supplementary file 1 [file marinedrugs-16-00207-s001.zip › Supplementary Figures and Tables/Supplementary File 1 _ KEGG pathways/map00030 (Pentose phosphate) [17 enz found].png]

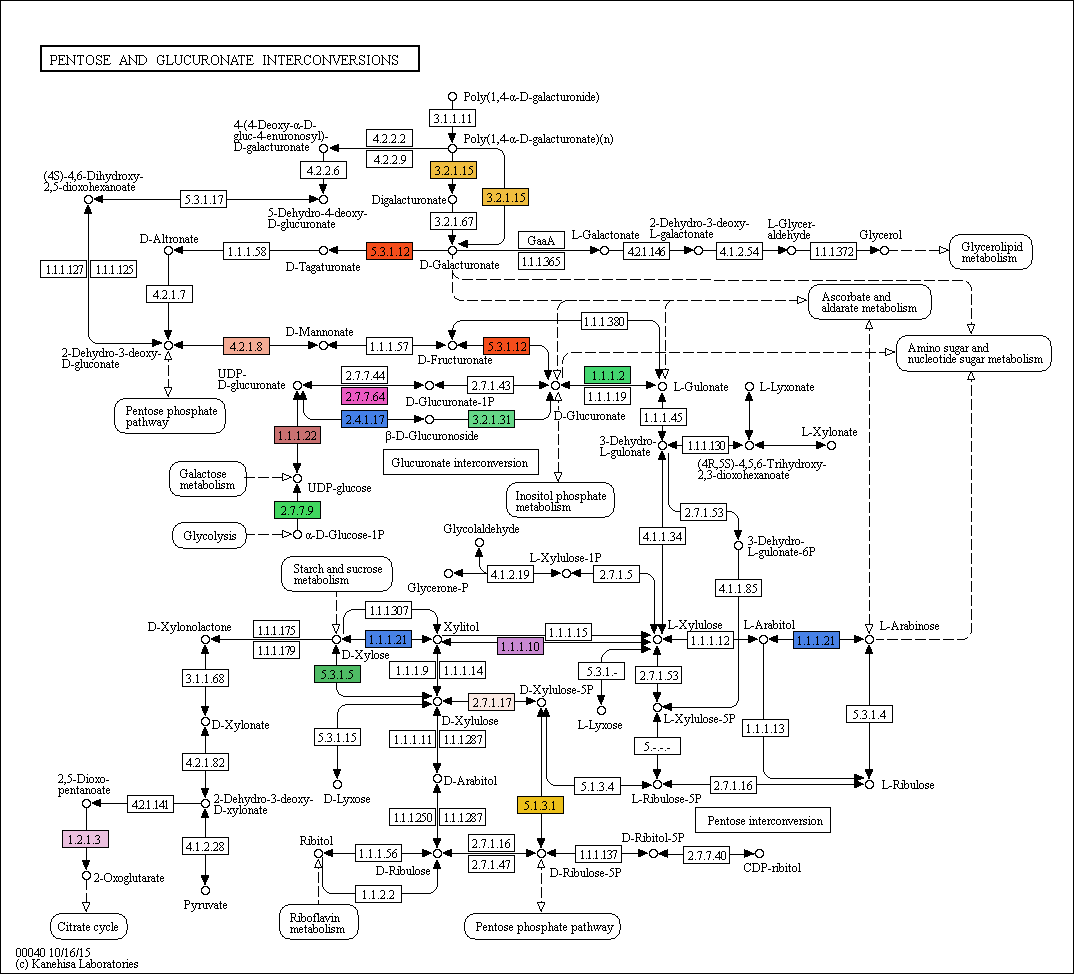

Supplement: Supplementary file 1 [file marinedrugs-16-00207-s001.zip › Supplementary Figures and Tables/Supplementary File 1 _ KEGG pathways/map00040 (Pentose and glucuronate interconversions) [15 enz found].png]

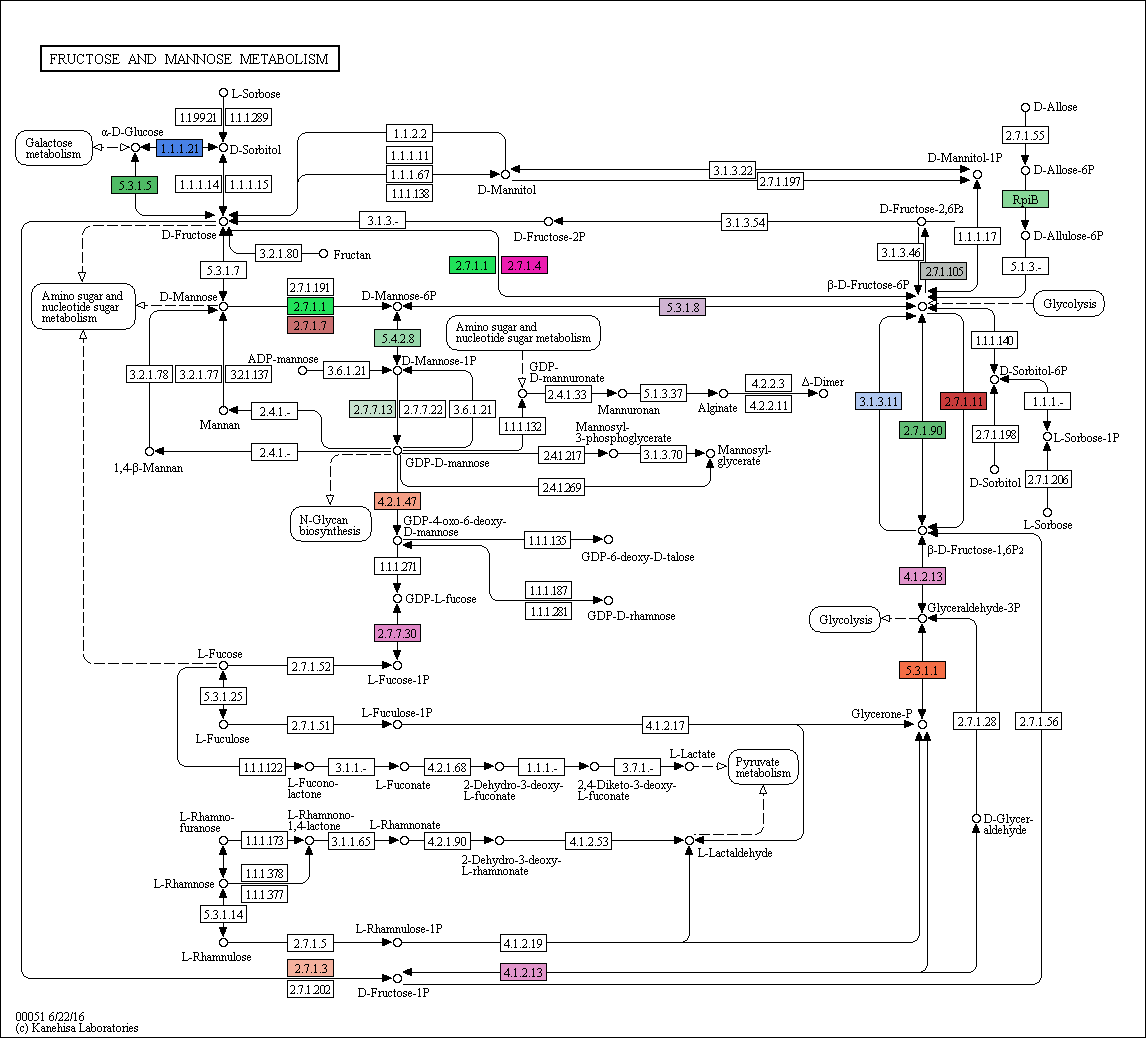

Supplement: Supplementary file 1 [file marinedrugs-16-00207-s001.zip › Supplementary Figures and Tables/Supplementary File 1 _ KEGG pathways/map00051 (Fructose and mannose metabolism) [18 enz found].png]

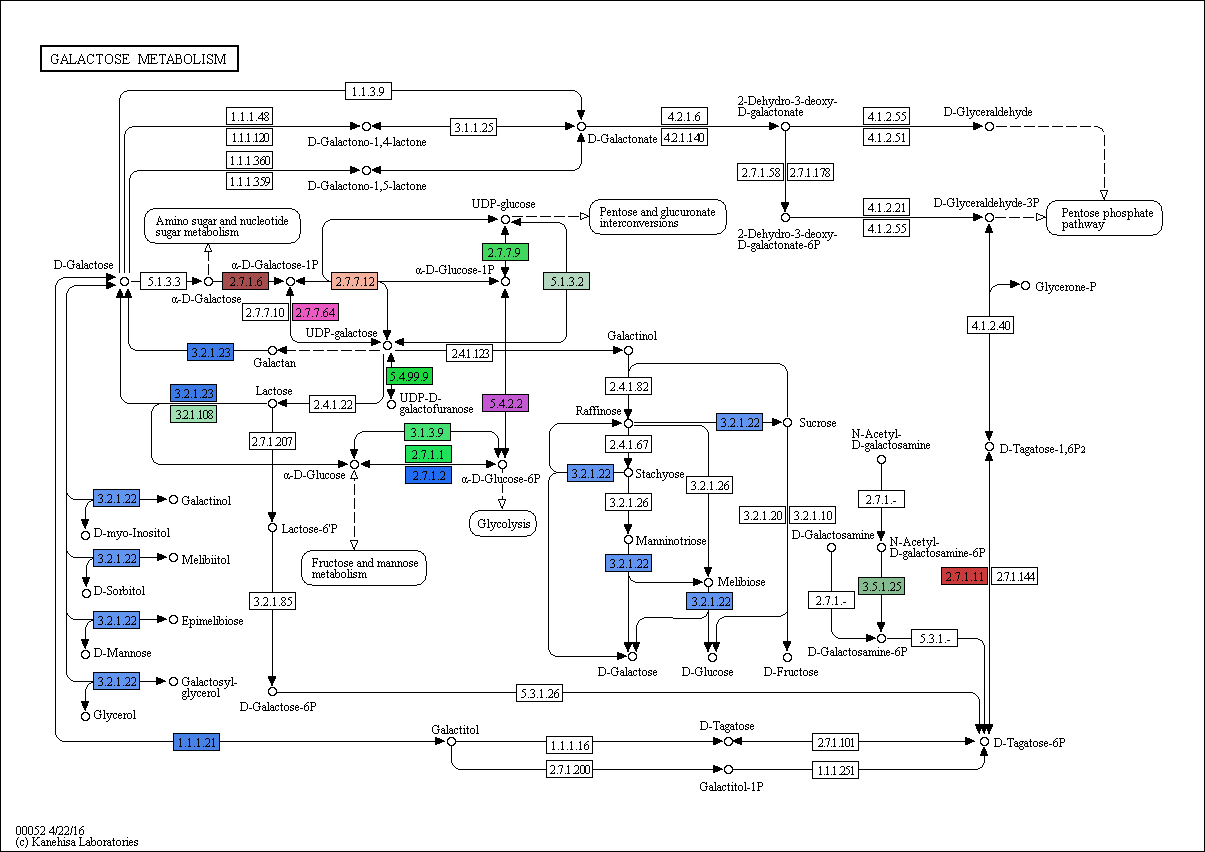

Supplement: Supplementary file 1 [file marinedrugs-16-00207-s001.zip › Supplementary Figures and Tables/Supplementary File 1 _ KEGG pathways/map00052 (Galactose metabolism) [16 enz found].png]

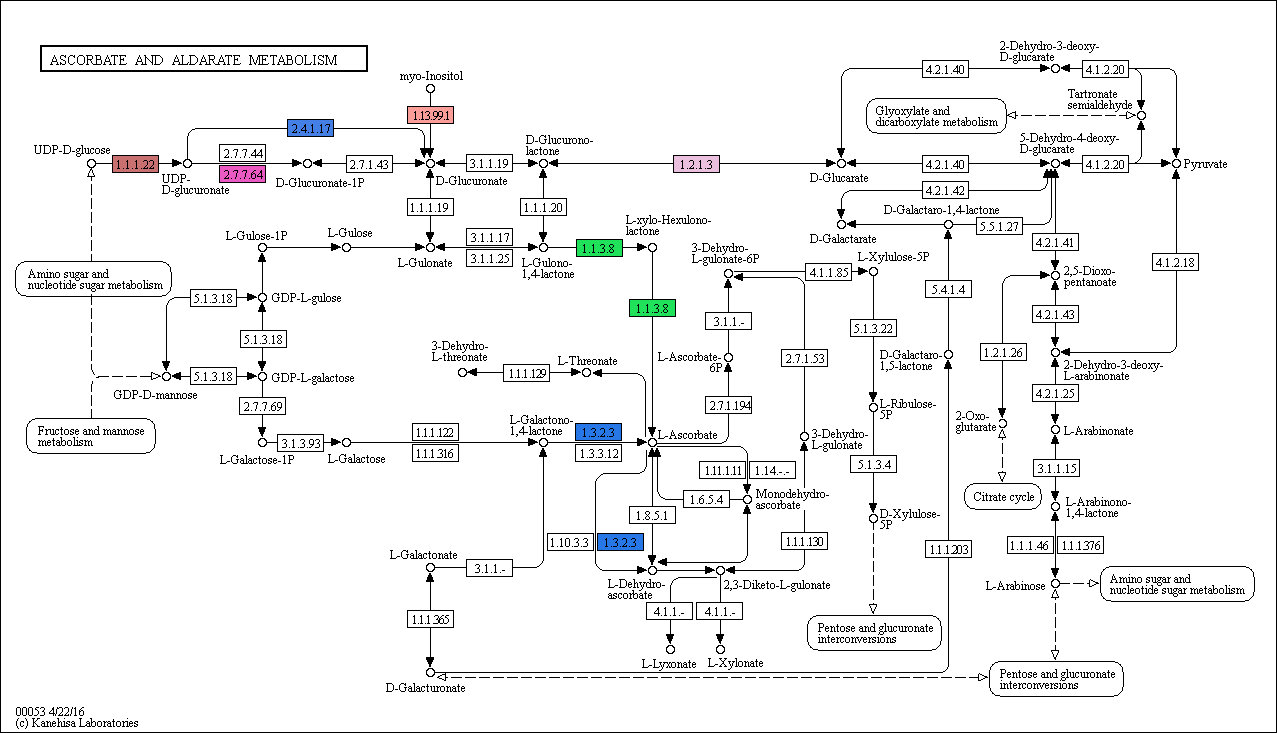

Supplement: Supplementary file 1 [file marinedrugs-16-00207-s001.zip › Supplementary Figures and Tables/Supplementary File 1 _ KEGG pathways/map00053 (Ascorbate and aldarate metabolsim) [7 enz found].png]

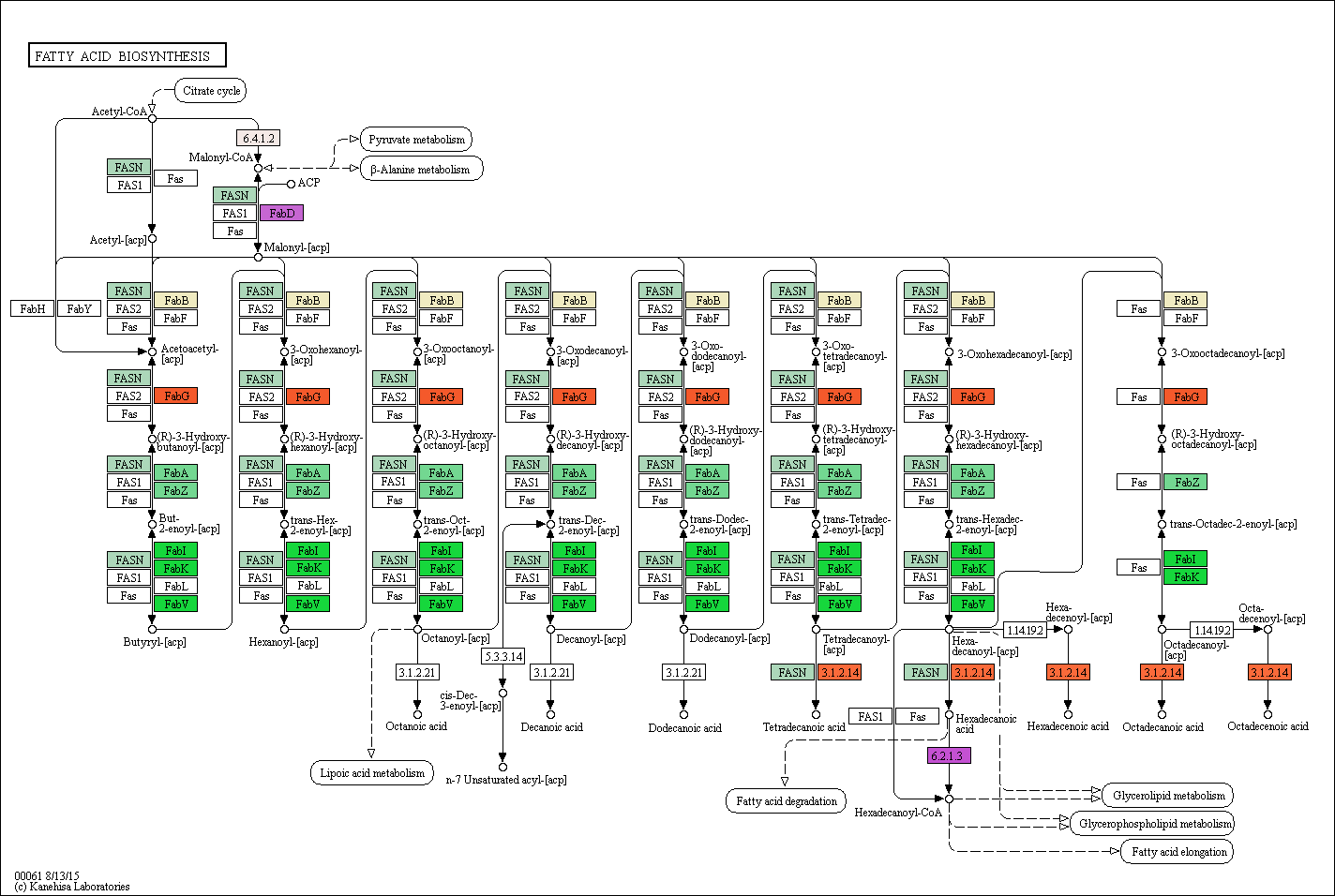

Supplement: Supplementary file 1 [file marinedrugs-16-00207-s001.zip › Supplementary Figures and Tables/Supplementary File 1 _ KEGG pathways/map00061 (Fatty acid biosynthesis) [10 enz found].png]

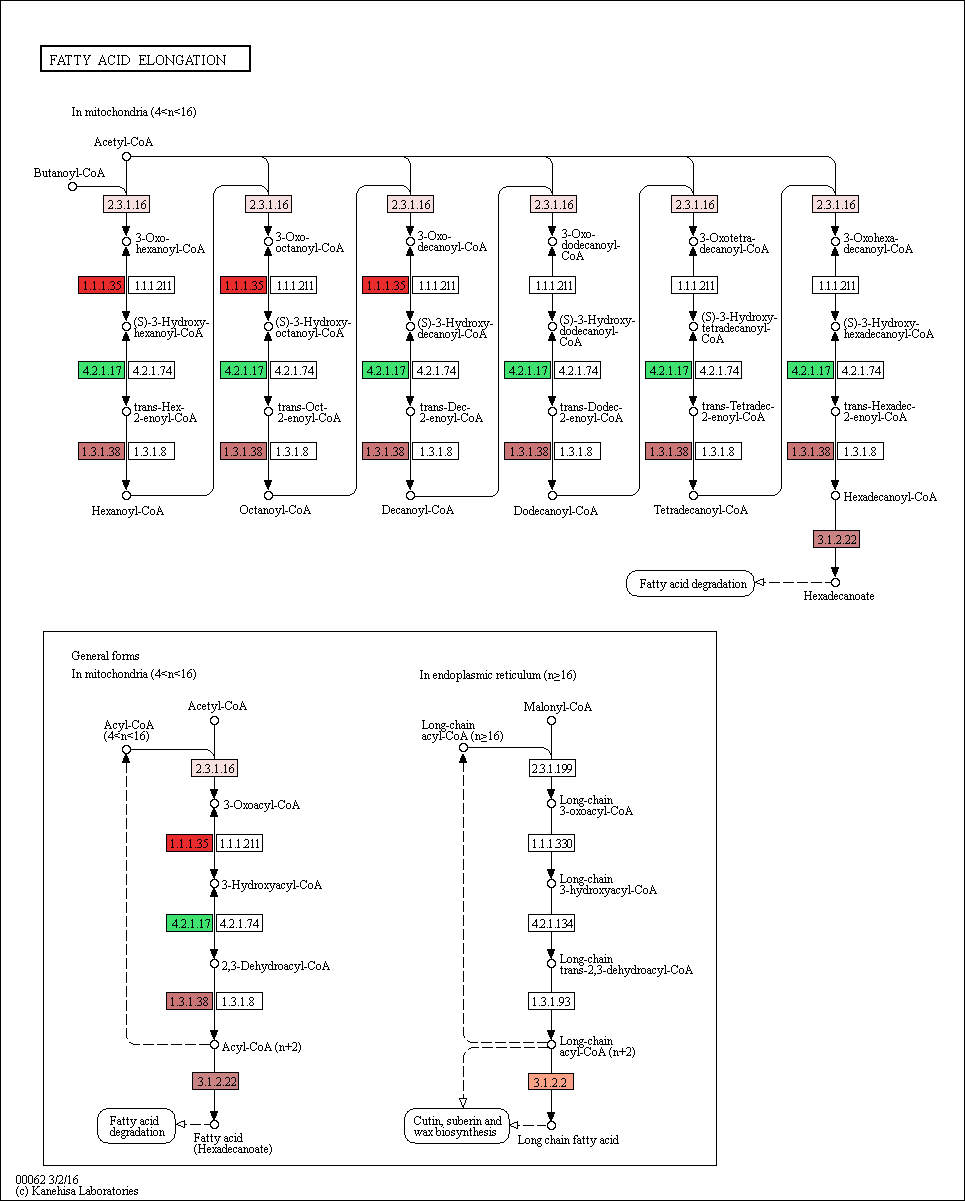

Supplement: Supplementary file 1 [file marinedrugs-16-00207-s001.zip › Supplementary Figures and Tables/Supplementary File 1 _ KEGG pathways/map00062 (Fatty acid elongation) [6 enz found].png]

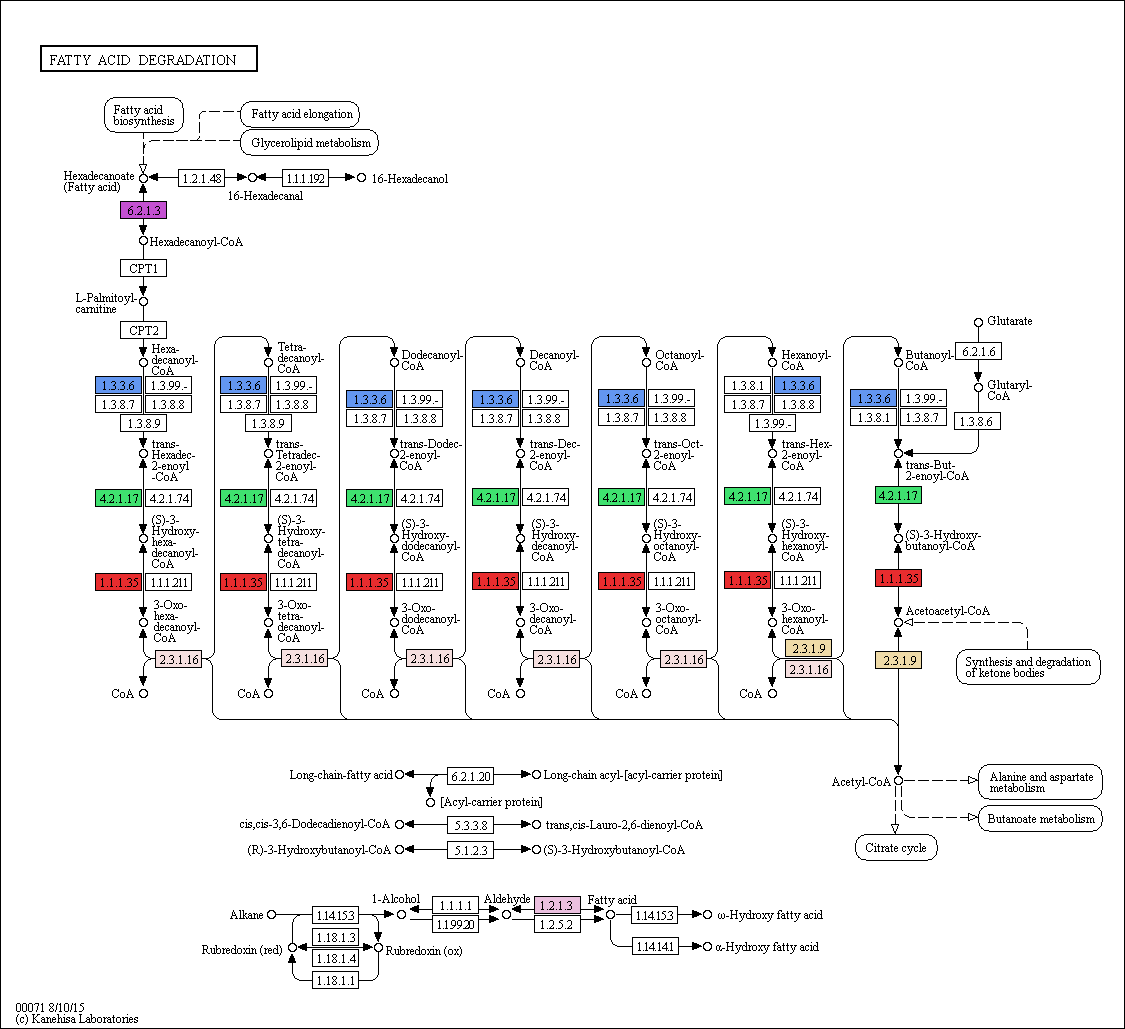

Supplement: Supplementary file 1 [file marinedrugs-16-00207-s001.zip › Supplementary Figures and Tables/Supplementary File 1 _ KEGG pathways/map00071 (Fatty acid degradation) [7 enz found].png]

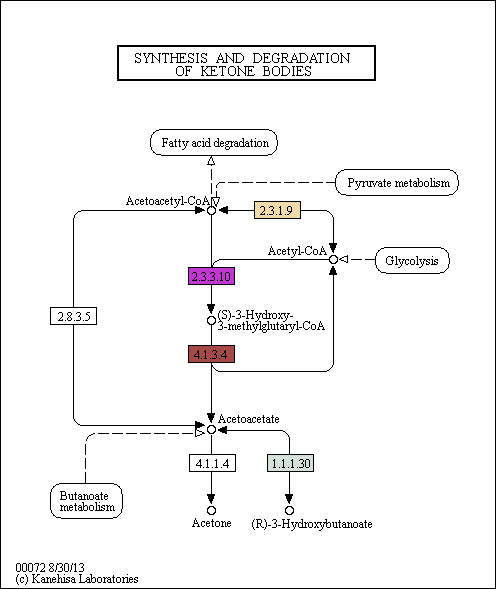

Supplement: Supplementary file 1 [file marinedrugs-16-00207-s001.zip › Supplementary Figures and Tables/Supplementary File 1 _ KEGG pathways/map00072 (Synthesis and degradation of ketone bodies) [4 enz found].png]

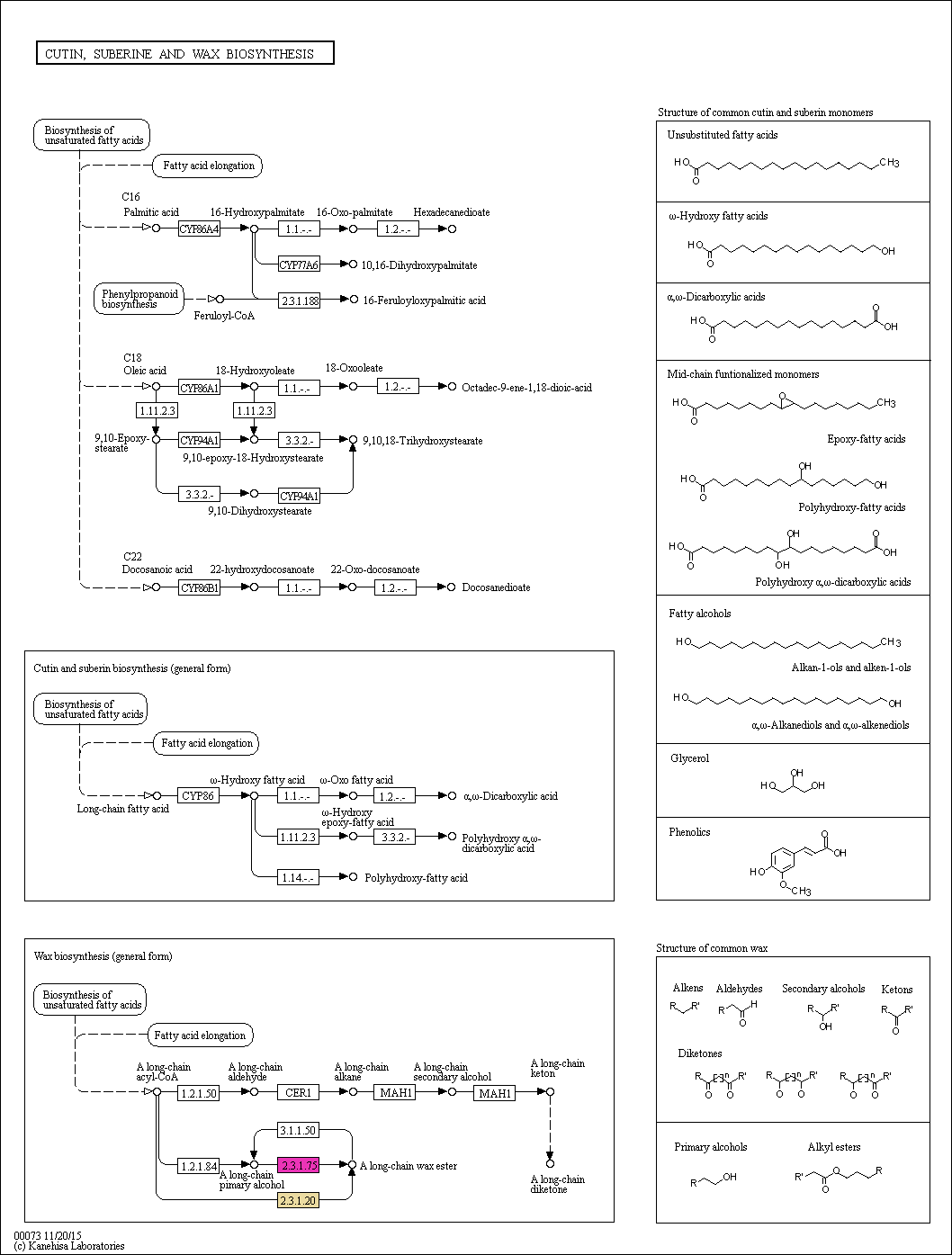

Supplement: Supplementary file 1 [file marinedrugs-16-00207-s001.zip › Supplementary Figures and Tables/Supplementary File 1 _ KEGG pathways/map00073 (Wax biosynthesis) [2 enz found].png]

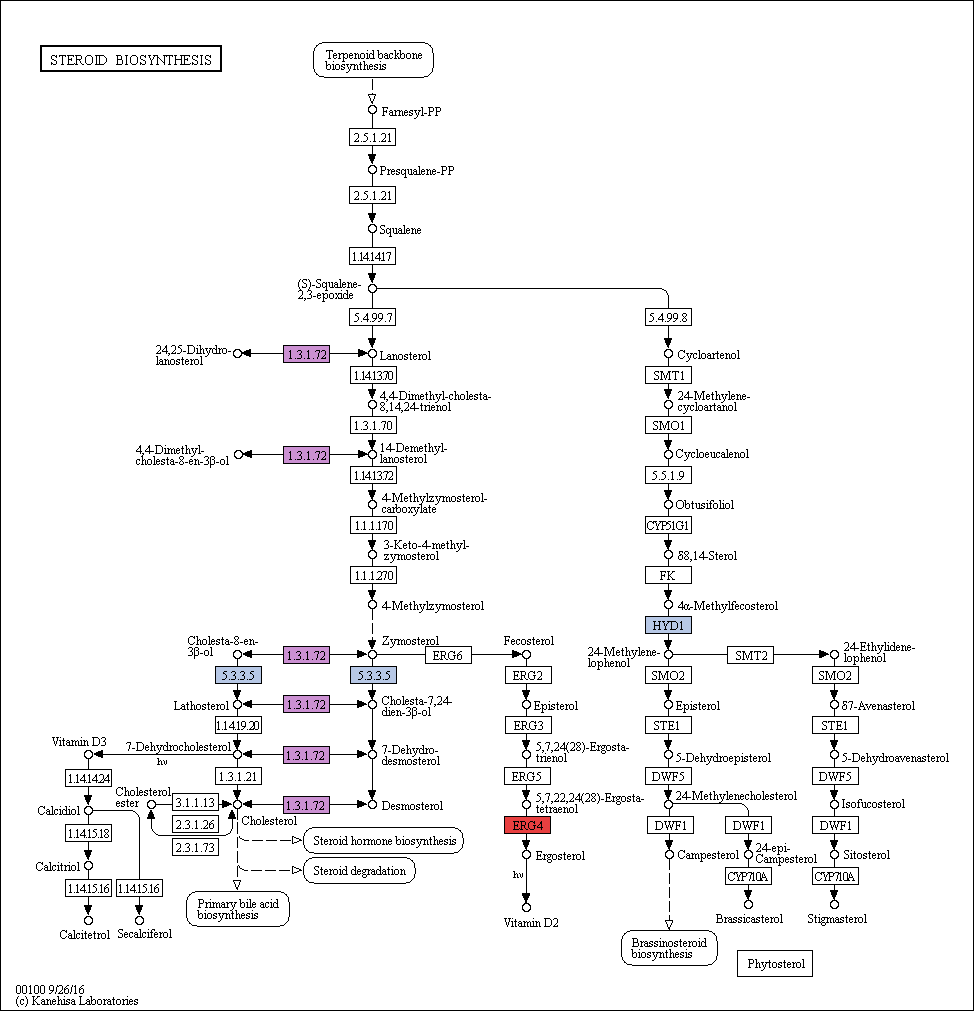

Supplement: Supplementary file 1 [file marinedrugs-16-00207-s001.zip › Supplementary Figures and Tables/Supplementary File 1 _ KEGG pathways/map00100 (Steroid biosynthesis) [3 enz found].png]

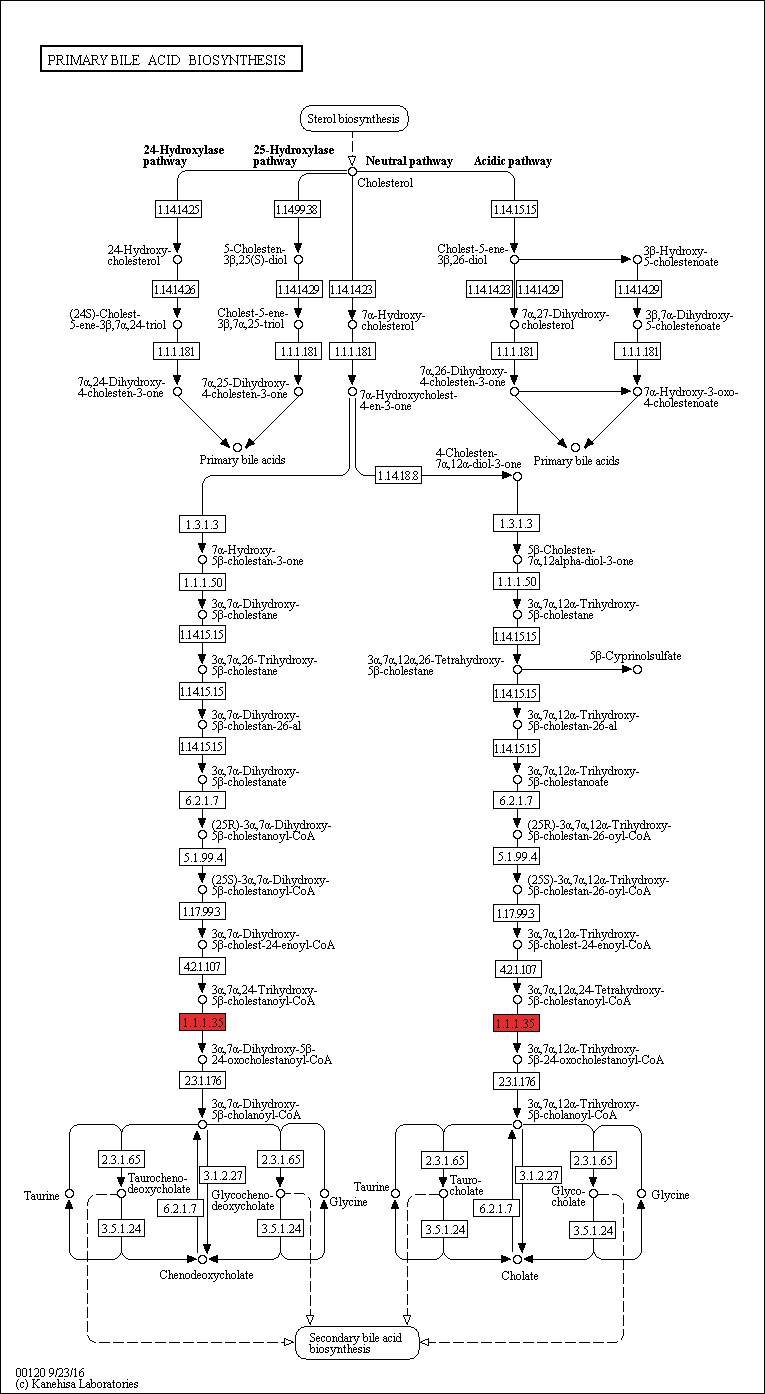

Supplement: Supplementary file 1 [file marinedrugs-16-00207-s001.zip › Supplementary Figures and Tables/Supplementary File 1 _ KEGG pathways/map00120 (Primary bile acid biosynthesis) [1 enz found].png]

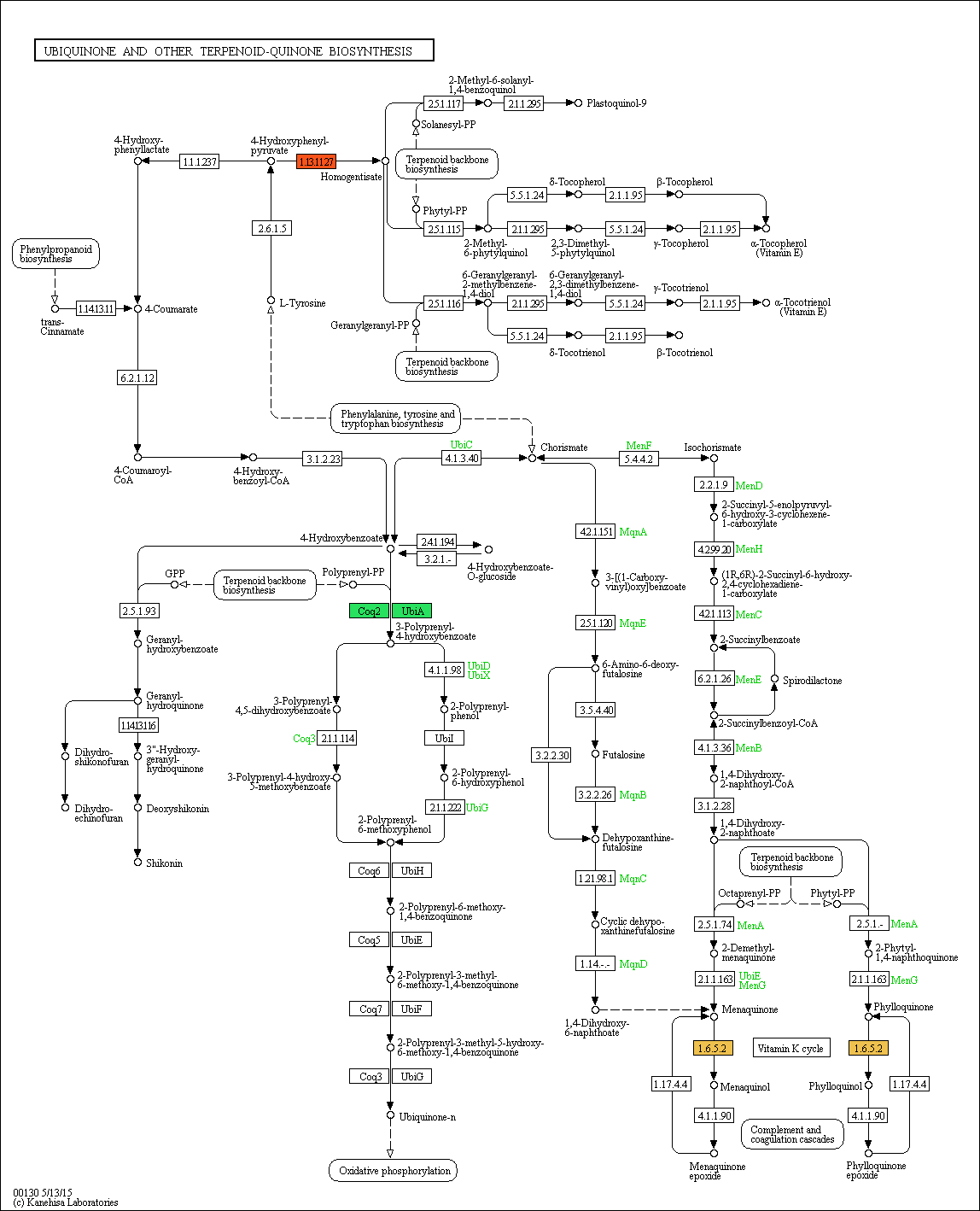

Supplement: Supplementary file 1 [file marinedrugs-16-00207-s001.zip › Supplementary Figures and Tables/Supplementary File 1 _ KEGG pathways/map00130 (Ubiquinone and other terpenoid-quinone biosynthesis) [3 enz found].png]

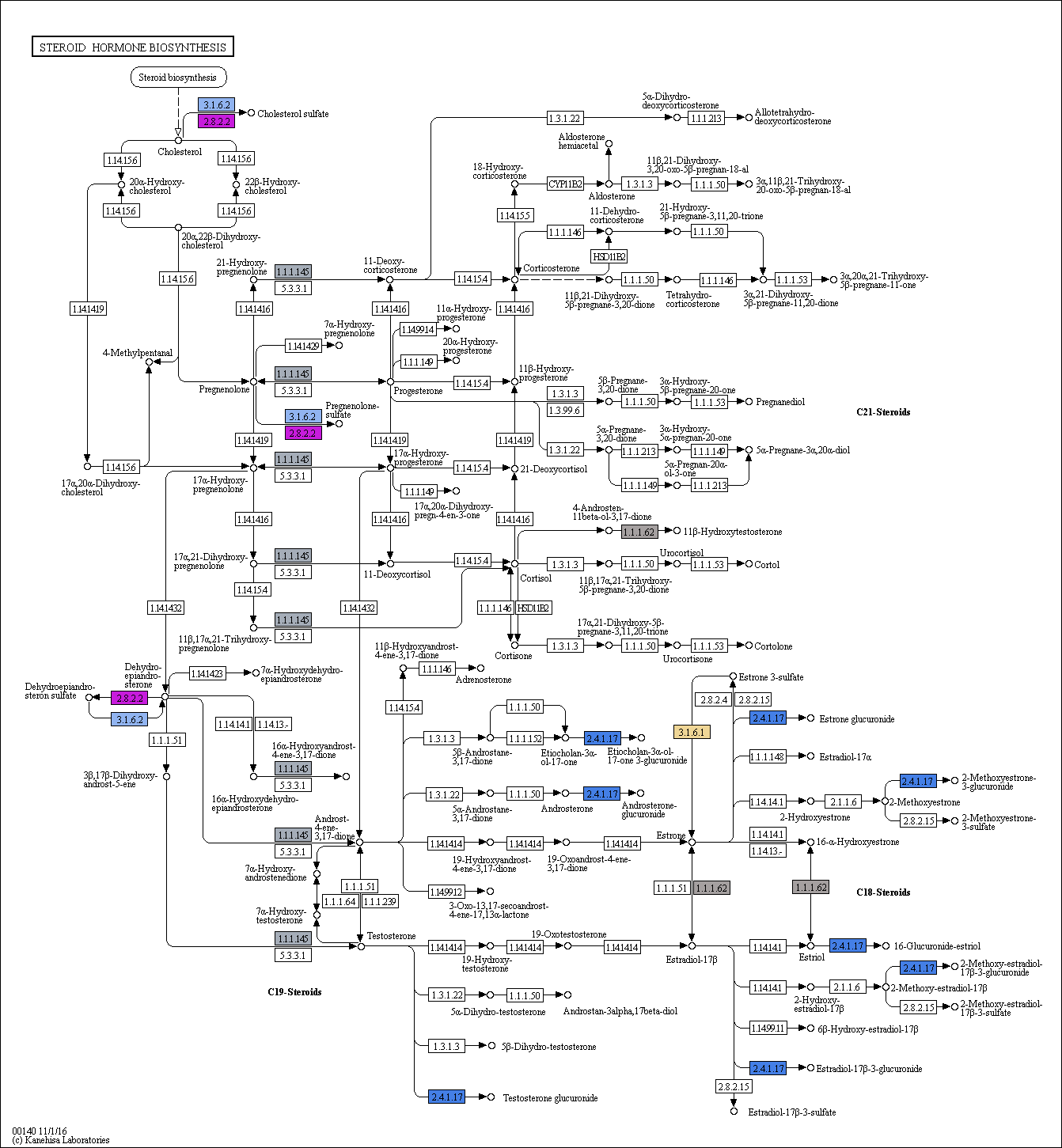

Supplement: Supplementary file 1 [file marinedrugs-16-00207-s001.zip › Supplementary Figures and Tables/Supplementary File 1 _ KEGG pathways/map00140 (Steroid hormone biosynthesis) [6 enz found].png]

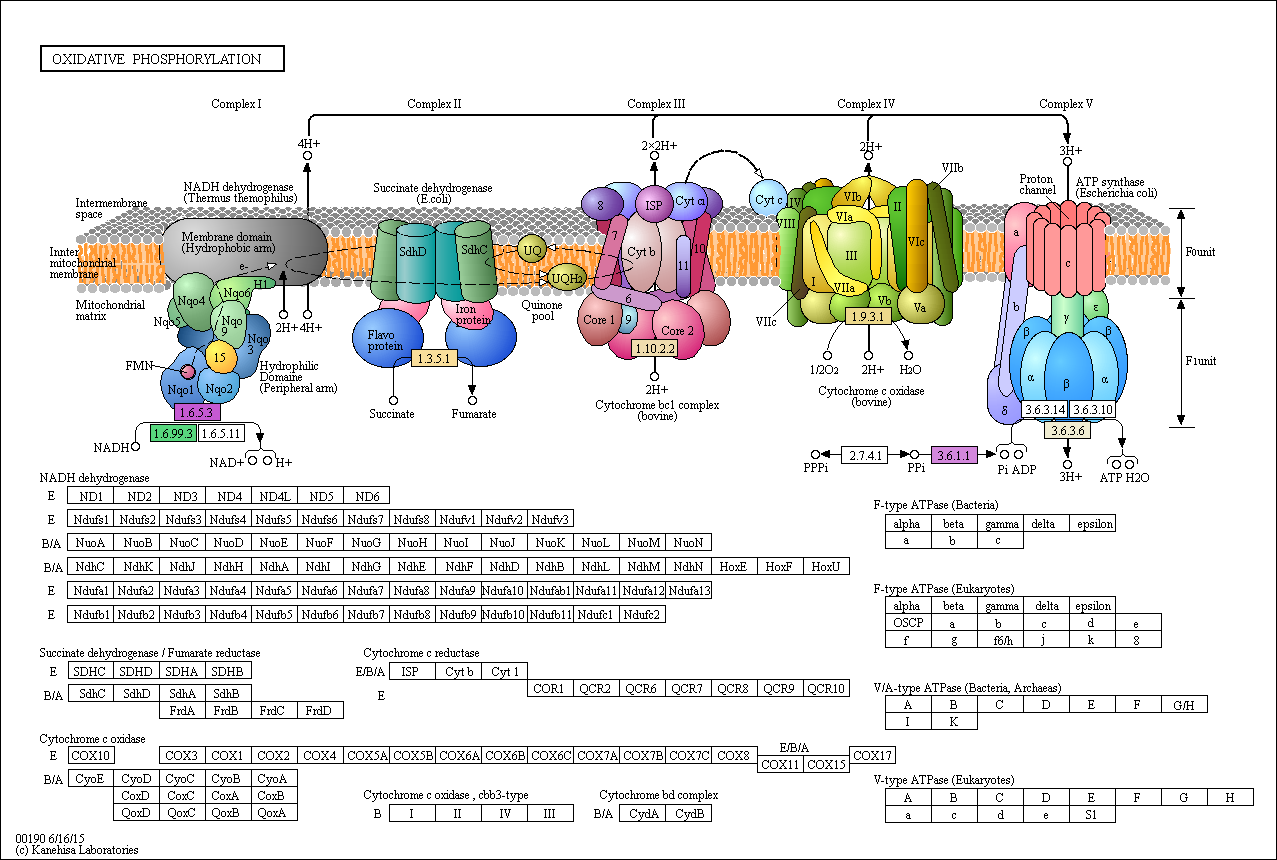

Supplement: Supplementary file 1 [file marinedrugs-16-00207-s001.zip › Supplementary Figures and Tables/Supplementary File 1 _ KEGG pathways/map00190 (Oxidative phosphorylation) [7 enz found].png]

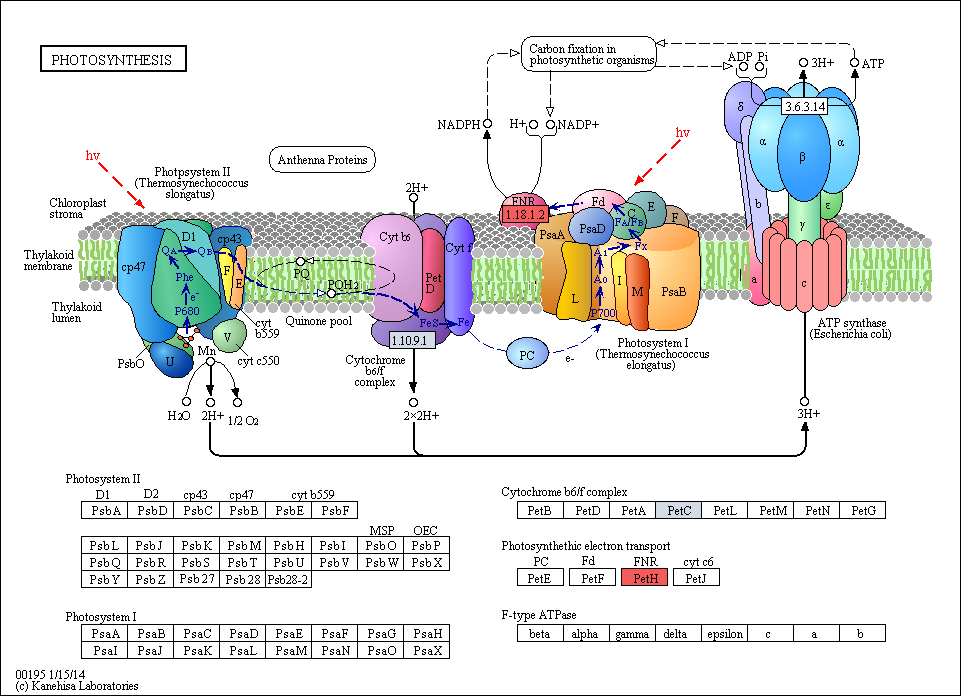

Supplement: Supplementary file 1 [file marinedrugs-16-00207-s001.zip › Supplementary Figures and Tables/Supplementary File 1 _ KEGG pathways/map00195 (Photosynthesis) [2 enz found].png]

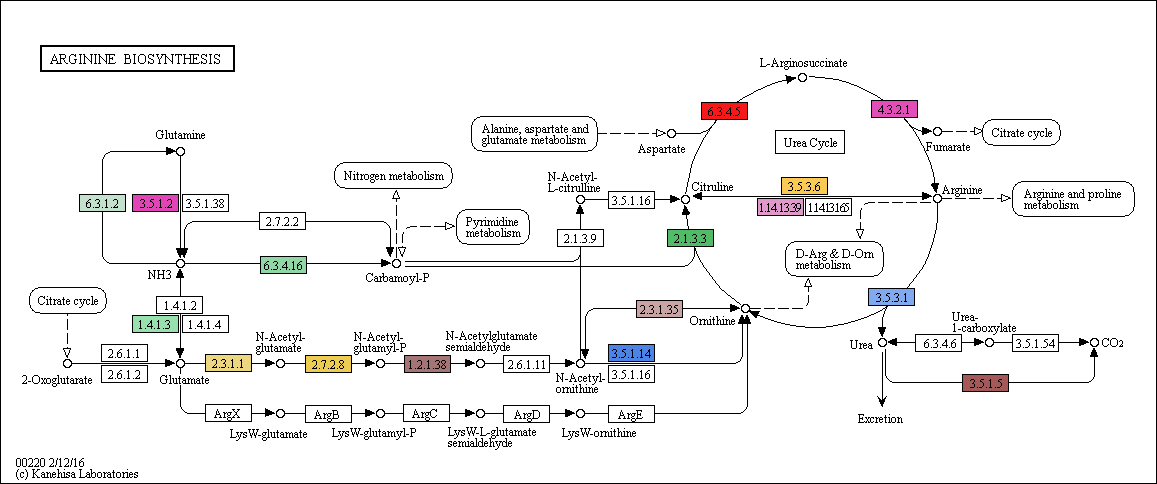

Supplement: Supplementary file 1 [file marinedrugs-16-00207-s001.zip › Supplementary Figures and Tables/Supplementary File 1 _ KEGG pathways/map00220 (Arginine biosynthesis) [16 enz found].png]

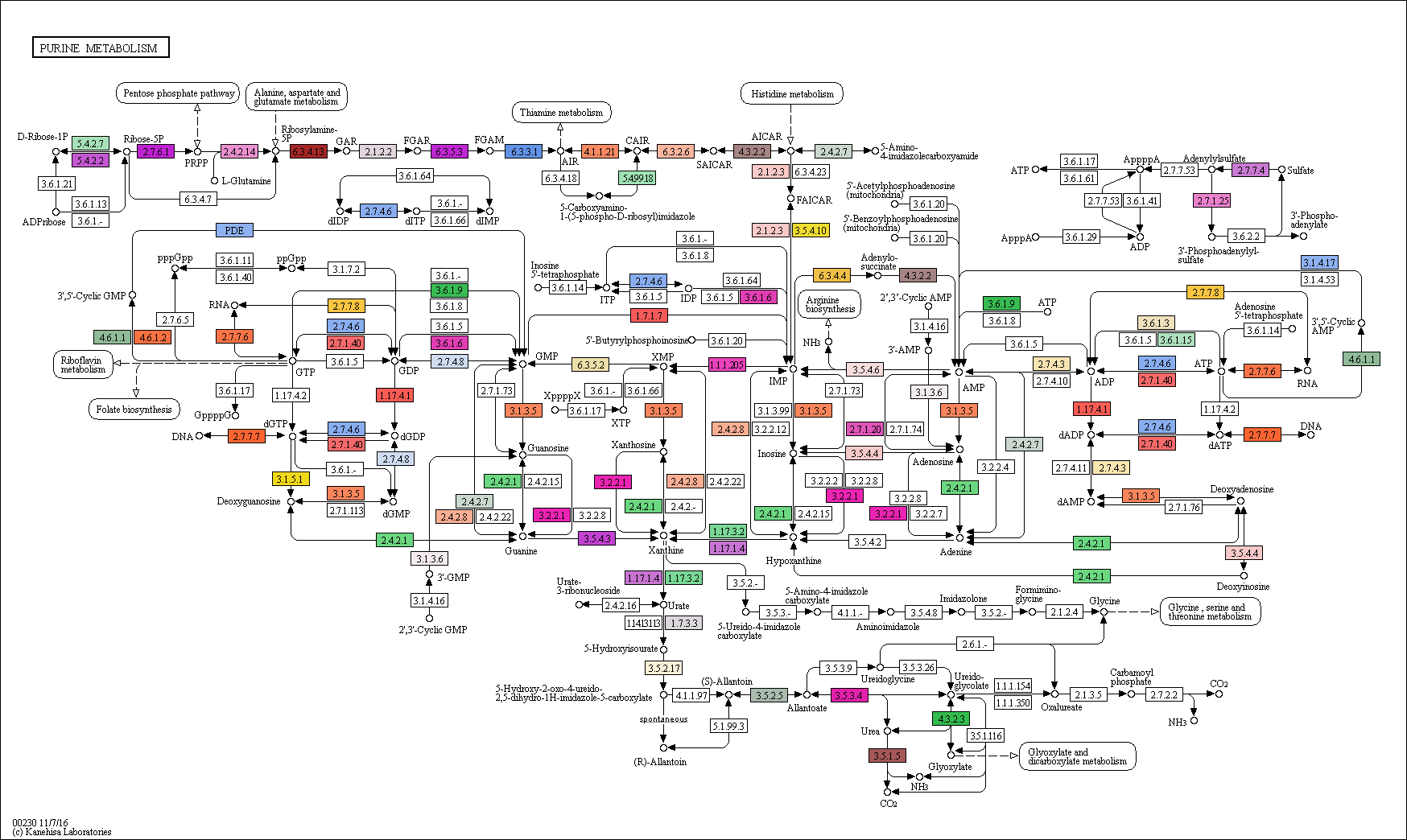

Supplement: Supplementary file 1 [file marinedrugs-16-00207-s001.zip › Supplementary Figures and Tables/Supplementary File 1 _ KEGG pathways/map00230 (Purine metabolism) [54 enz found].png]

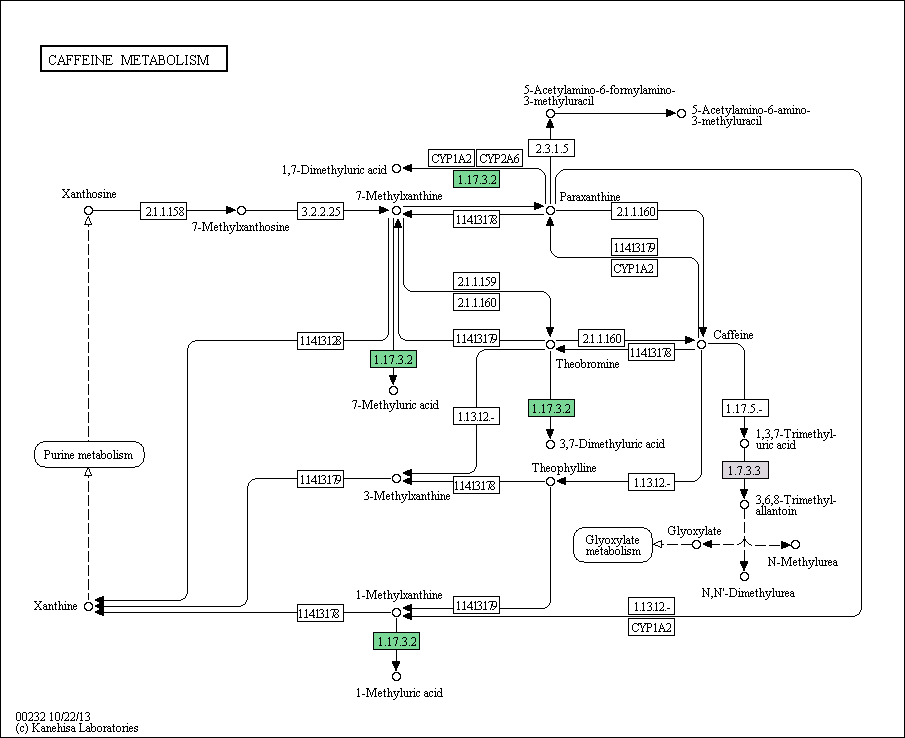

Supplement: Supplementary file 1 [file marinedrugs-16-00207-s001.zip › Supplementary Figures and Tables/Supplementary File 1 _ KEGG pathways/map00232 (Caffeine metabolism) [2 enz found].png]

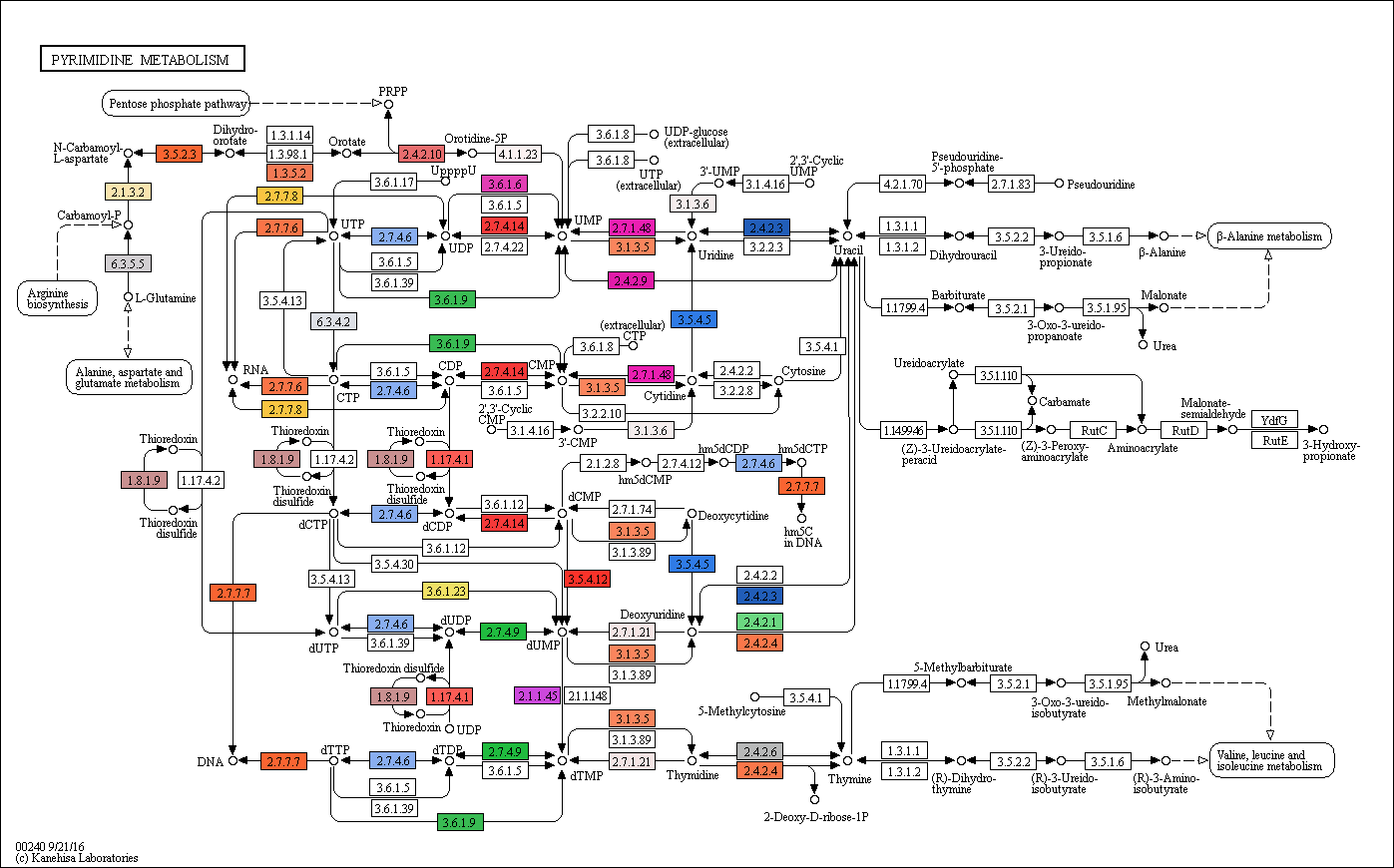

Supplement: Supplementary file 1 [file marinedrugs-16-00207-s001.zip › Supplementary Figures and Tables/Supplementary File 1 _ KEGG pathways/map00240 (Pyrimidine metabolism) [30 enz found].png]

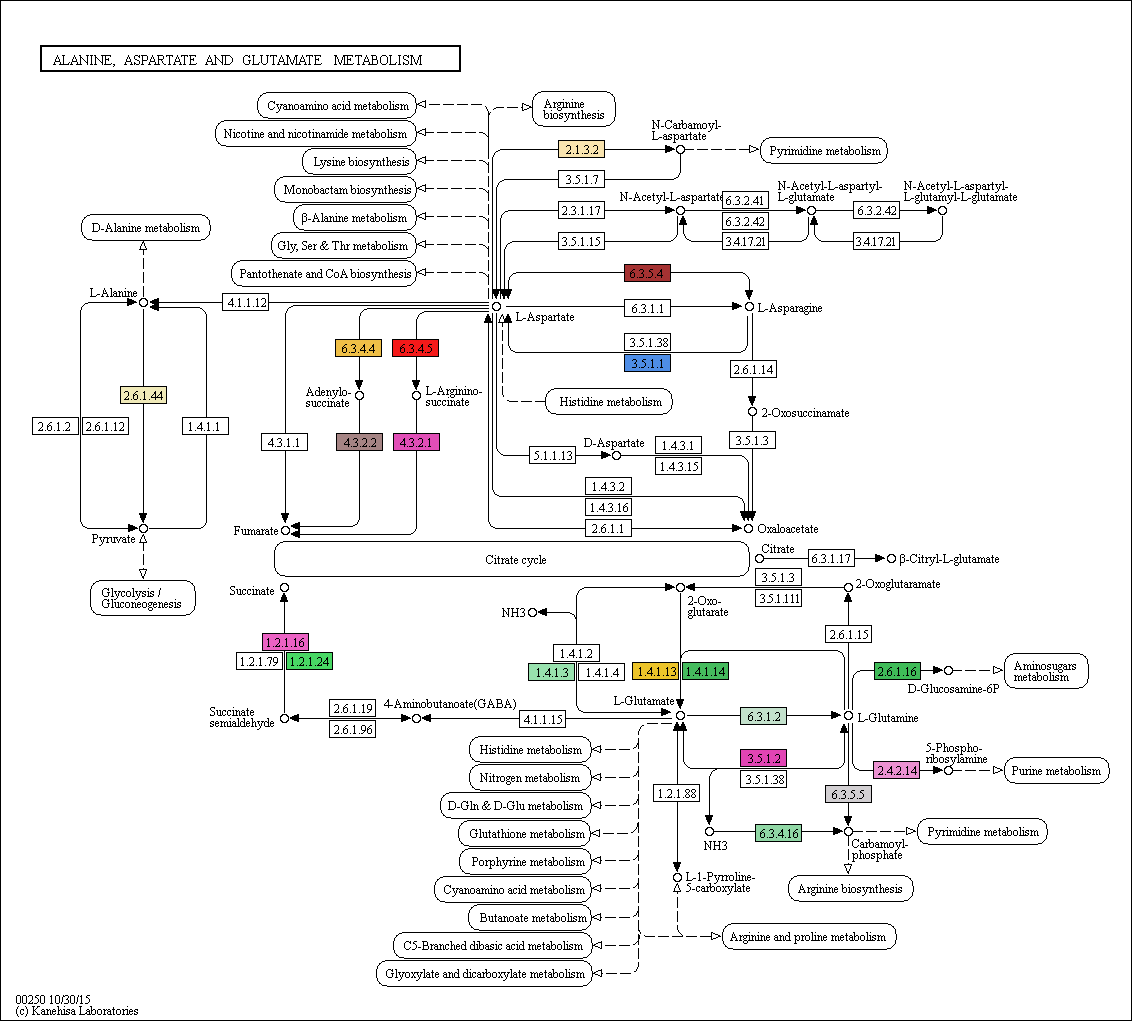

Supplement: Supplementary file 1 [file marinedrugs-16-00207-s001.zip › Supplementary Figures and Tables/Supplementary File 1 _ KEGG pathways/map00250 (Alanine, aspartate and glutamate metabolism) [19 enz found].png]

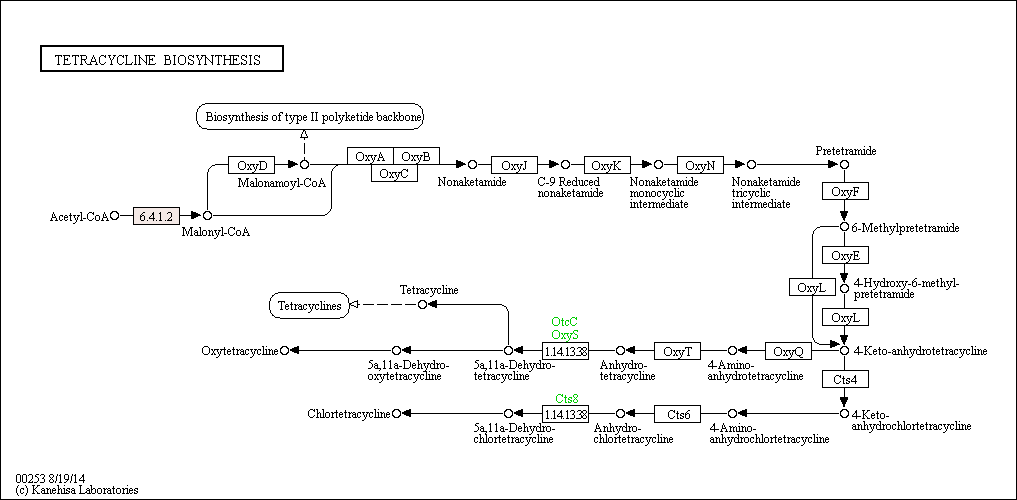

Supplement: Supplementary file 1 [file marinedrugs-16-00207-s001.zip › Supplementary Figures and Tables/Supplementary File 1 _ KEGG pathways/map00253 (Tetracycline biosynthesis) [1 enz found].png]

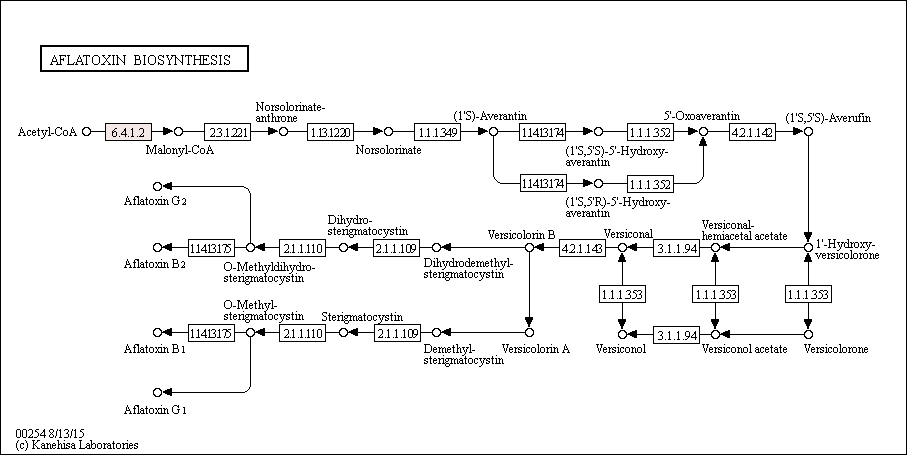

Supplement: Supplementary file 1 [file marinedrugs-16-00207-s001.zip › Supplementary Figures and Tables/Supplementary File 1 _ KEGG pathways/map00254 (Aflatoxin biosynthesis) [1 enz found].png]

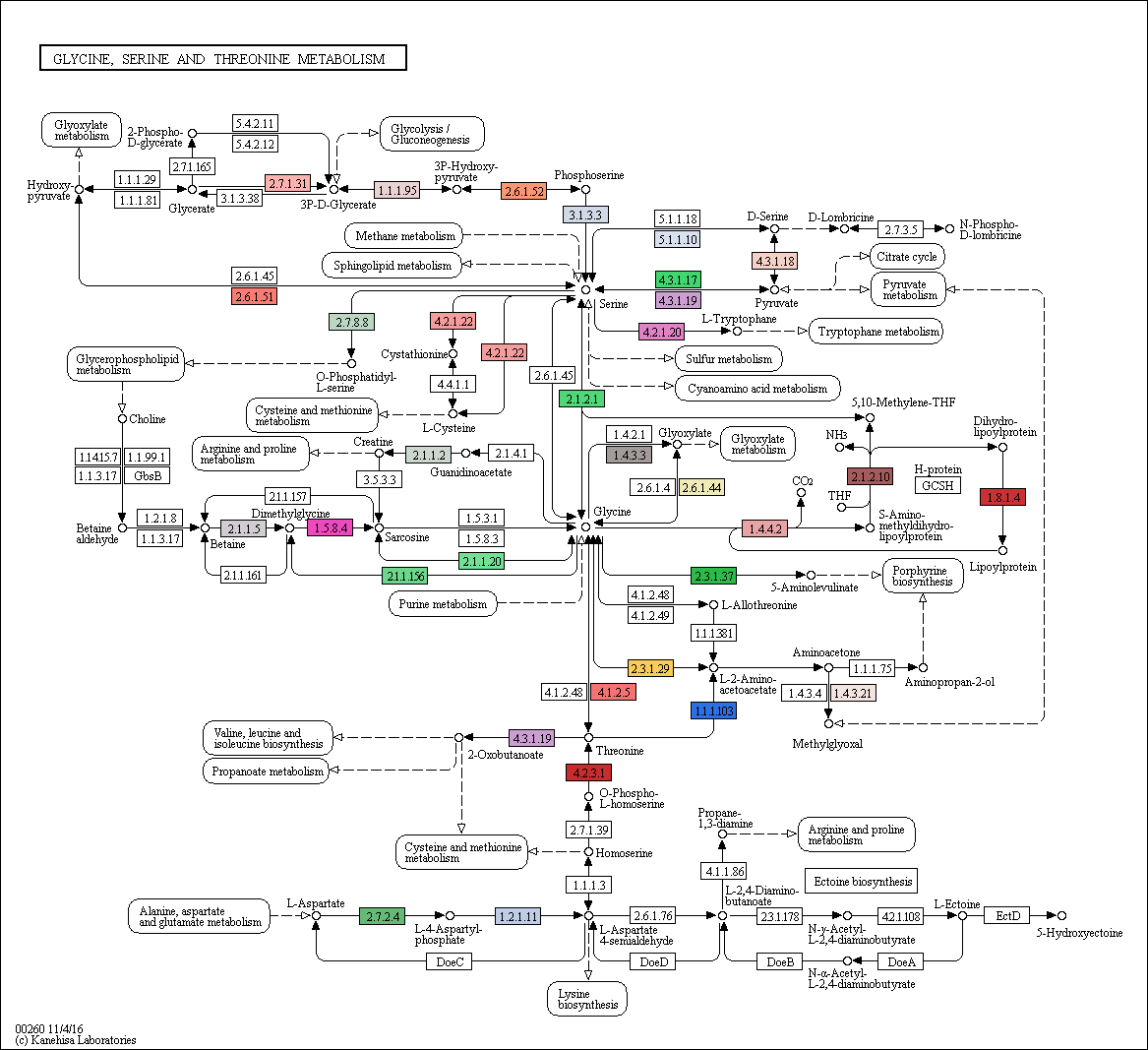

Supplement: Supplementary file 1 [file marinedrugs-16-00207-s001.zip › Supplementary Figures and Tables/Supplementary File 1 _ KEGG pathways/map00260 (Glycine, serine and threonine metabolism) [31 enz found].png]

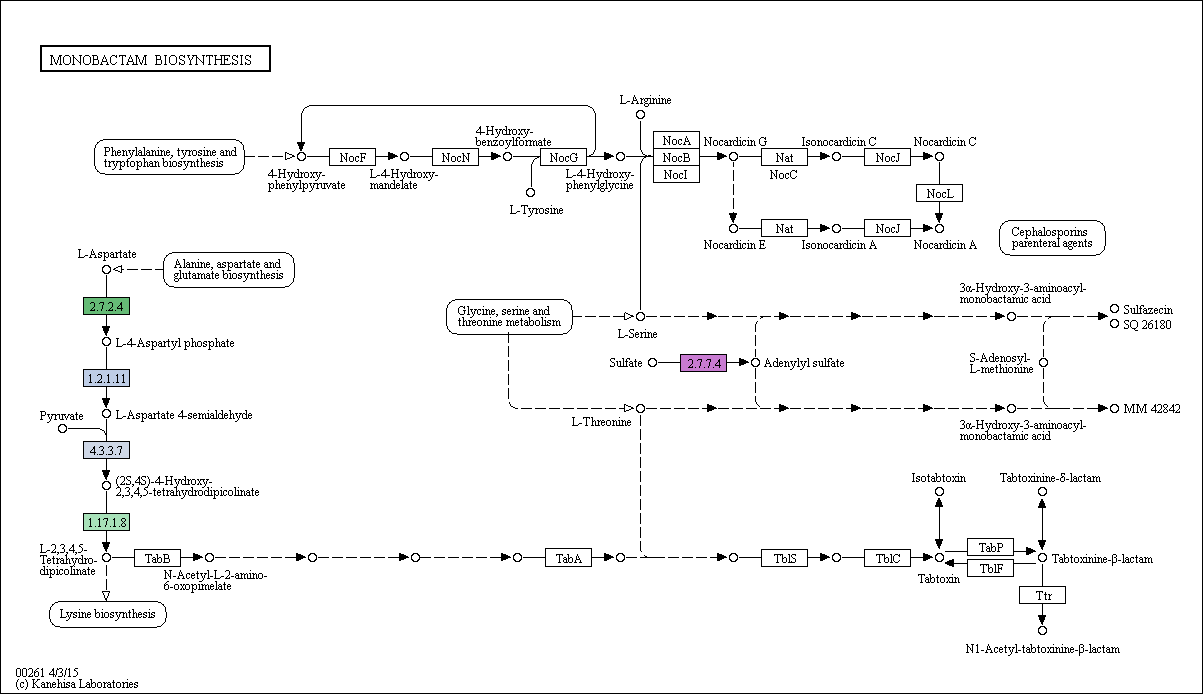

Supplement: Supplementary file 1 [file marinedrugs-16-00207-s001.zip › Supplementary Figures and Tables/Supplementary File 1 _ KEGG pathways/map00261 (Monobactam biosynthesis) [5 enz found].png]

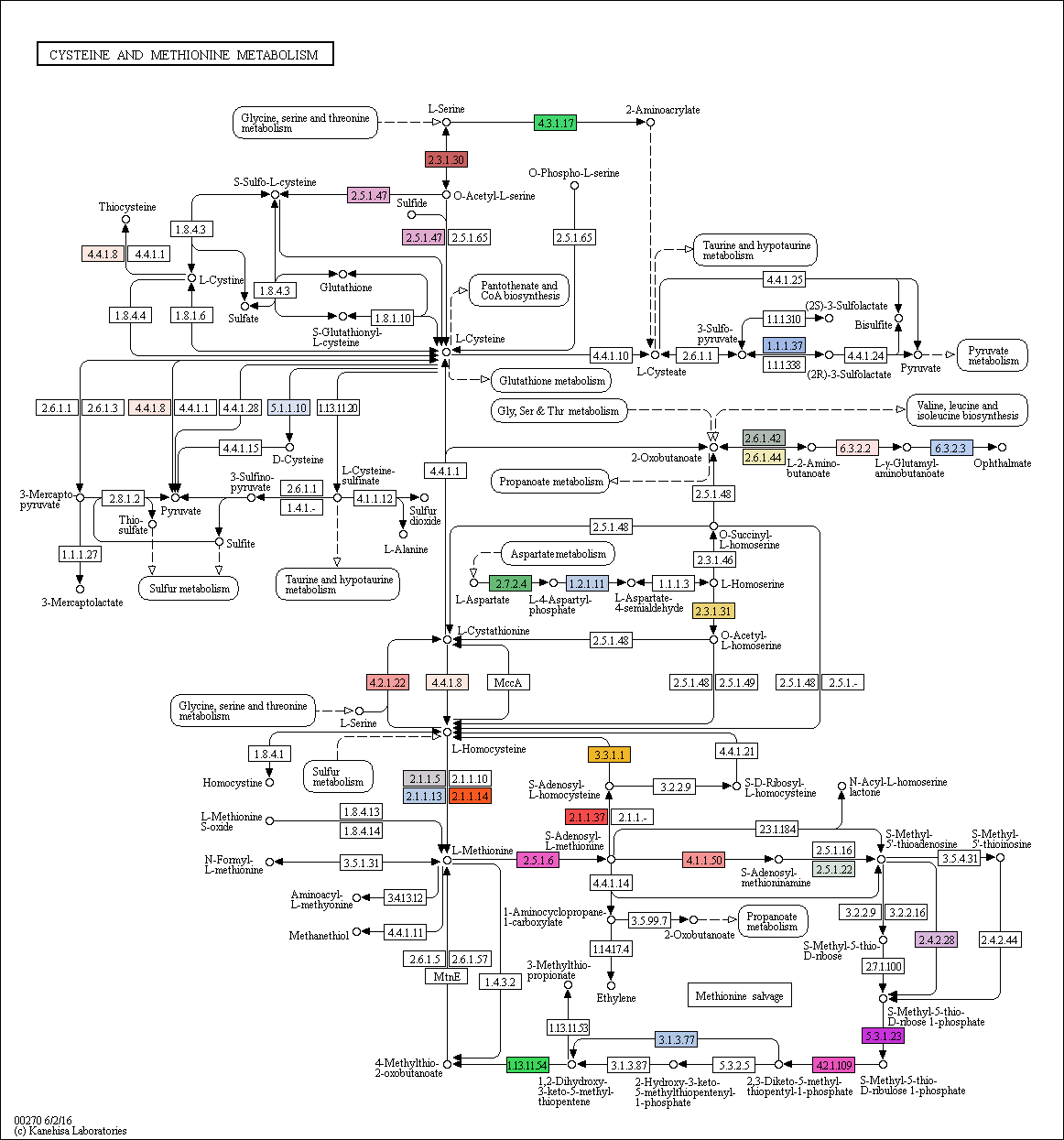

Supplement: Supplementary file 1 [file marinedrugs-16-00207-s001.zip › Supplementary Figures and Tables/Supplementary File 1 _ KEGG pathways/map00270 (Cysteine and methionine metabolsim) [27 enz found].png]

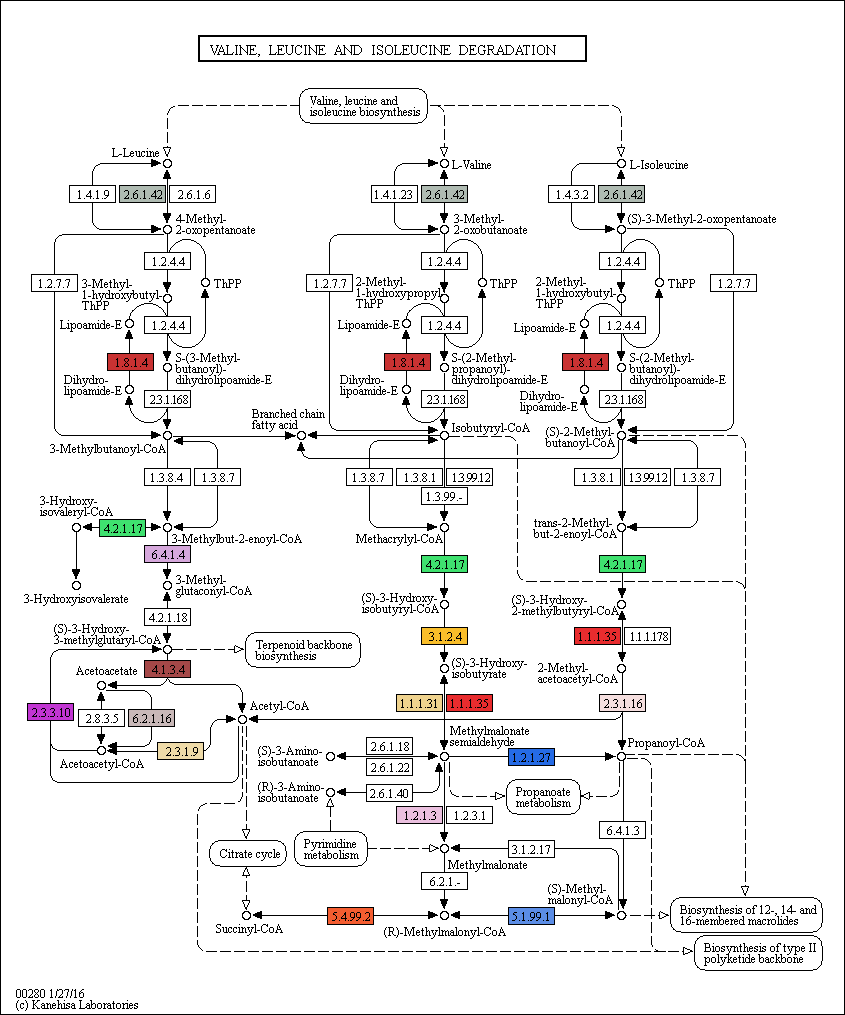

Supplement: Supplementary file 1 [file marinedrugs-16-00207-s001.zip › Supplementary Figures and Tables/Supplementary File 1 _ KEGG pathways/map00280 (Valine, leucine and isoleucine degradation) [16 enz found].png]

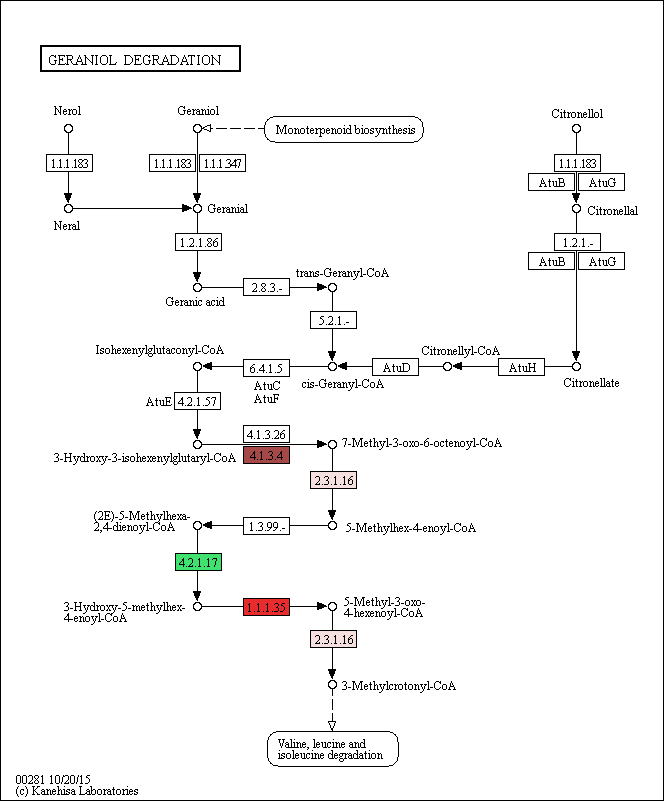

Supplement: Supplementary file 1 [file marinedrugs-16-00207-s001.zip › Supplementary Figures and Tables/Supplementary File 1 _ KEGG pathways/map00281 (geraniol degradation) [4 enz found].png]

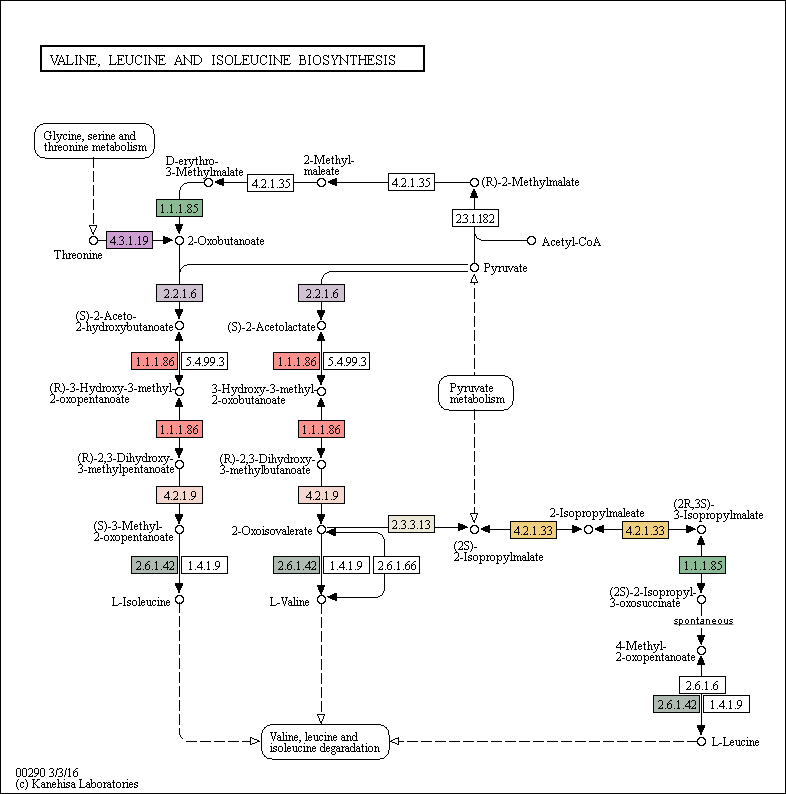

Supplement: Supplementary file 1 [file marinedrugs-16-00207-s001.zip › Supplementary Figures and Tables/Supplementary File 1 _ KEGG pathways/map00290 (Valine, leucine and isoleucine biosynthesis) [8 enz found].png]

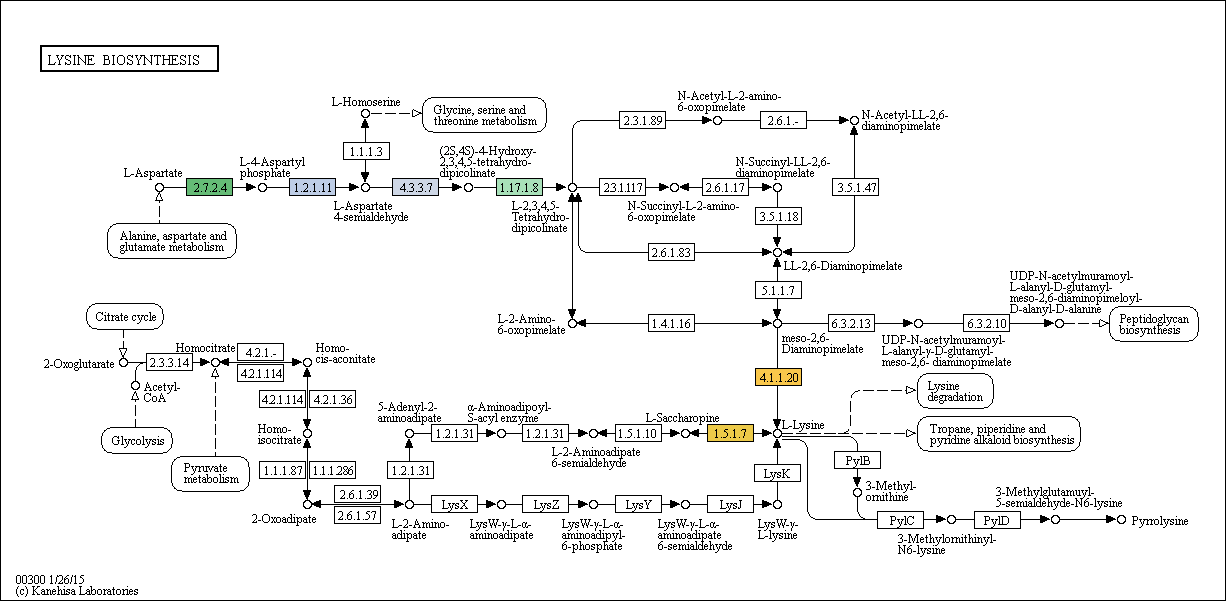

Supplement: Supplementary file 1 [file marinedrugs-16-00207-s001.zip › Supplementary Figures and Tables/Supplementary File 1 _ KEGG pathways/map00300 (Lysine biosynthesis) [6 enz found].png]

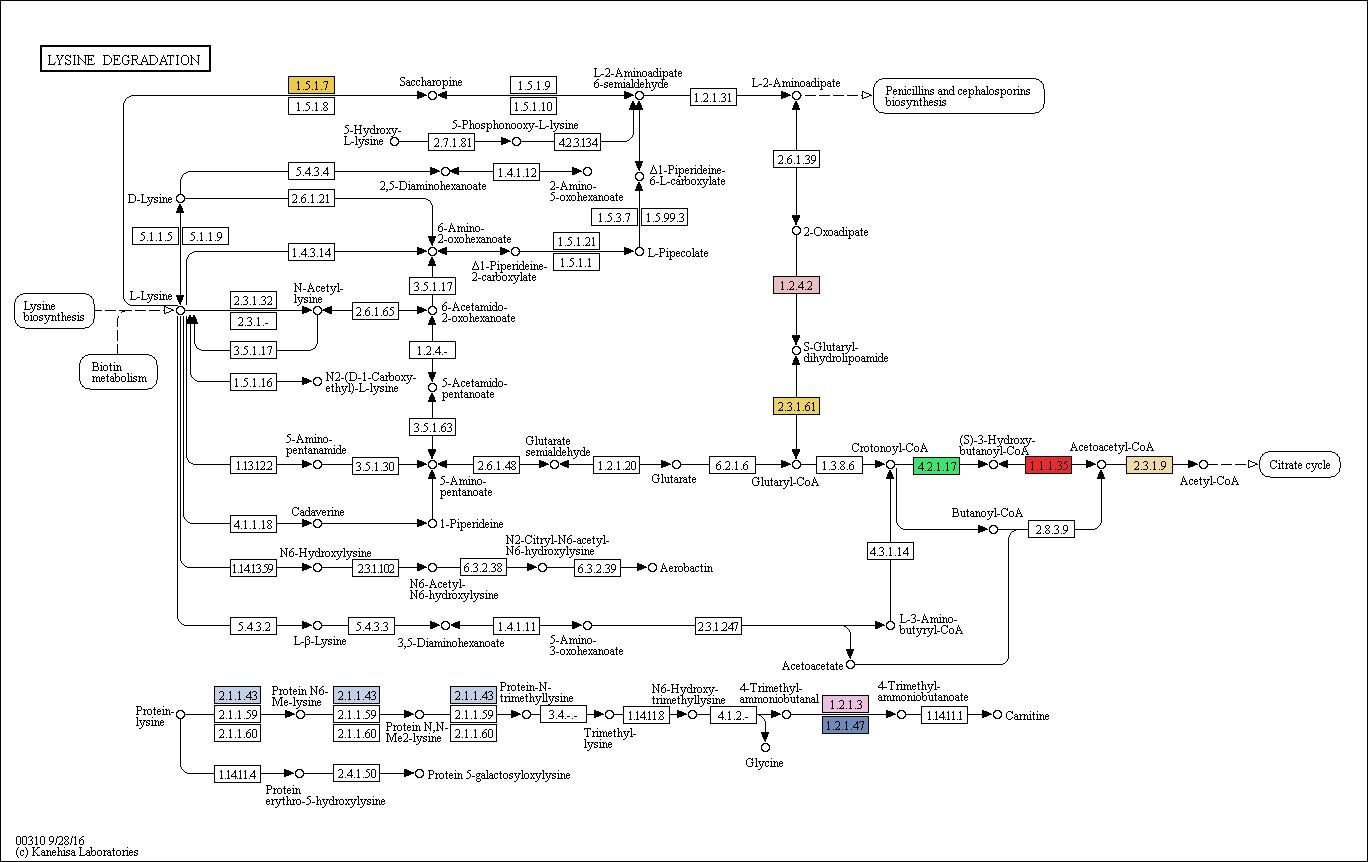

Supplement: Supplementary file 1 [file marinedrugs-16-00207-s001.zip › Supplementary Figures and Tables/Supplementary File 1 _ KEGG pathways/map00310 (Lysine degradation) [9 enz found].png]

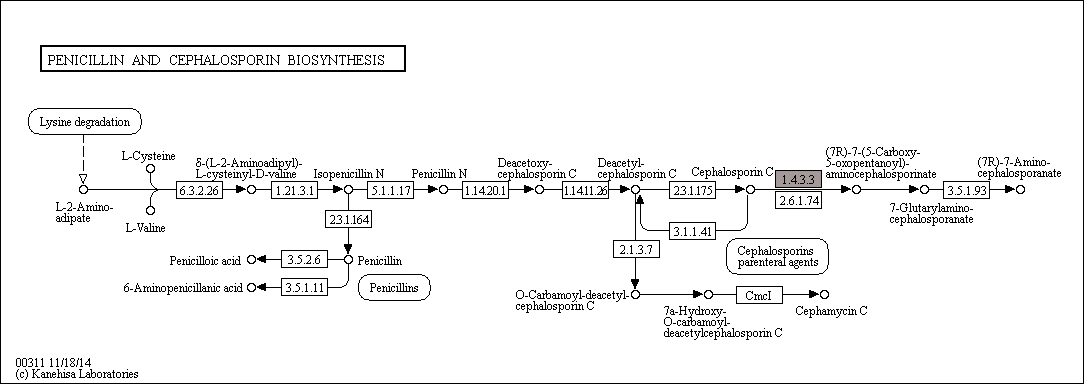

Supplement: Supplementary file 1 [file marinedrugs-16-00207-s001.zip › Supplementary Figures and Tables/Supplementary File 1 _ KEGG pathways/map00311 (Penicillin and cephalosporin biosynthesis) [1 enz found].png]

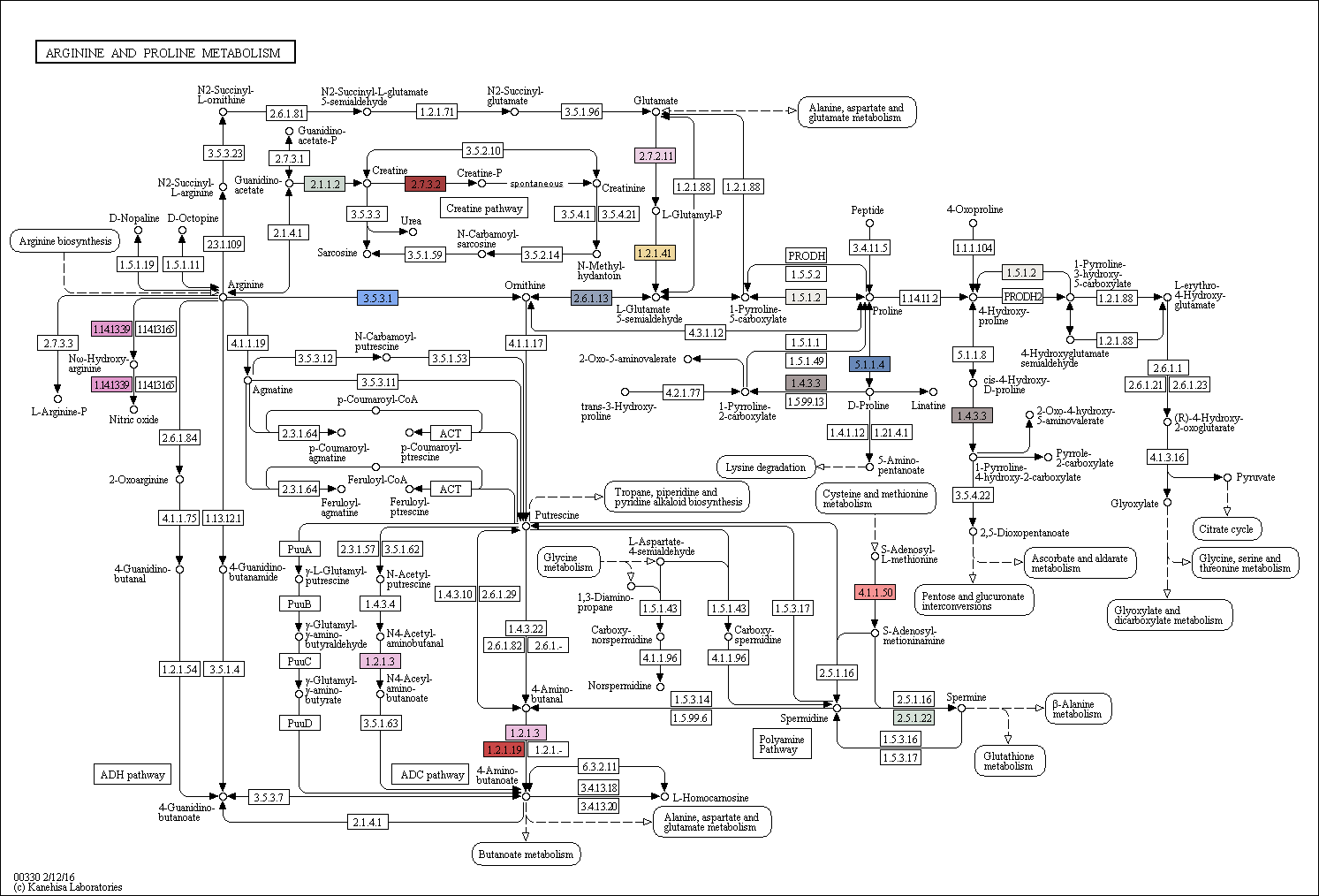

Supplement: Supplementary file 1 [file marinedrugs-16-00207-s001.zip › Supplementary Figures and Tables/Supplementary File 1 _ KEGG pathways/map00330 (Arginine and proline metabolism) [14 enz found].png]

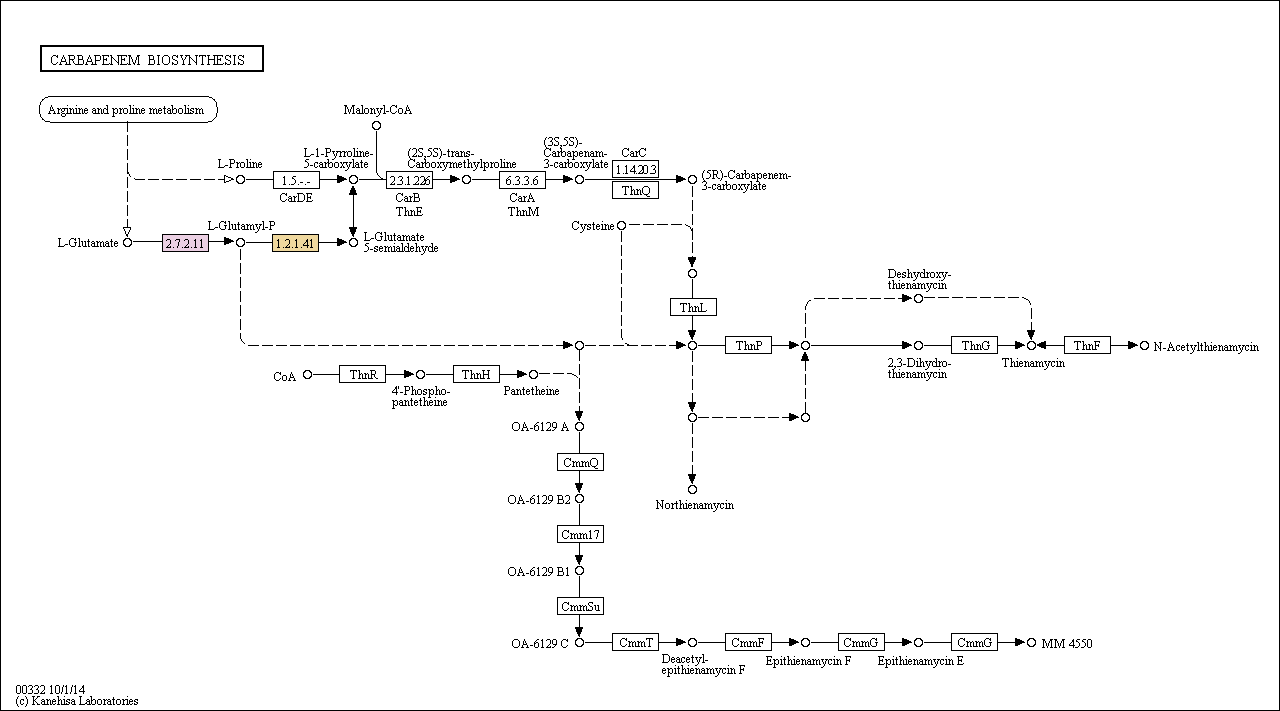

Supplement: Supplementary file 1 [file marinedrugs-16-00207-s001.zip › Supplementary Figures and Tables/Supplementary File 1 _ KEGG pathways/map00332 (Carbapenem biosynthesis) [2 enz found].png]

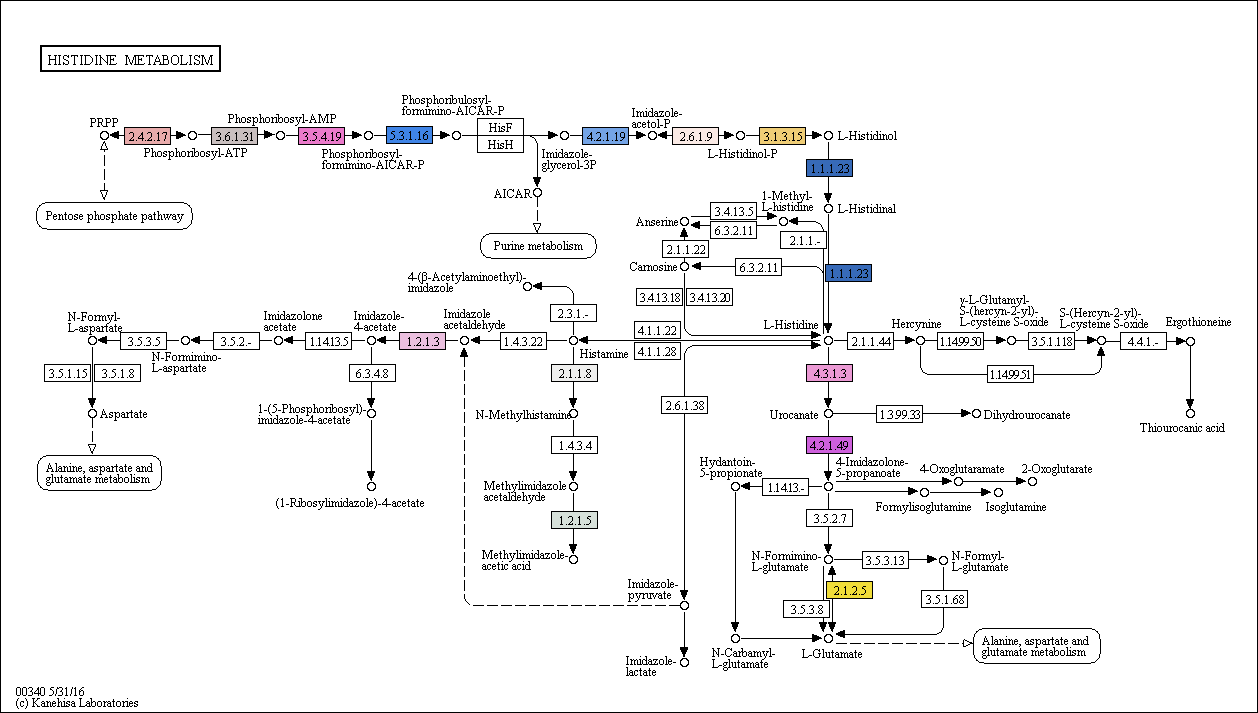

Supplement: Supplementary file 1 [file marinedrugs-16-00207-s001.zip › Supplementary Figures and Tables/Supplementary File 1 _ KEGG pathways/map00340 (Histidine metabolism) [14 enz found].png]

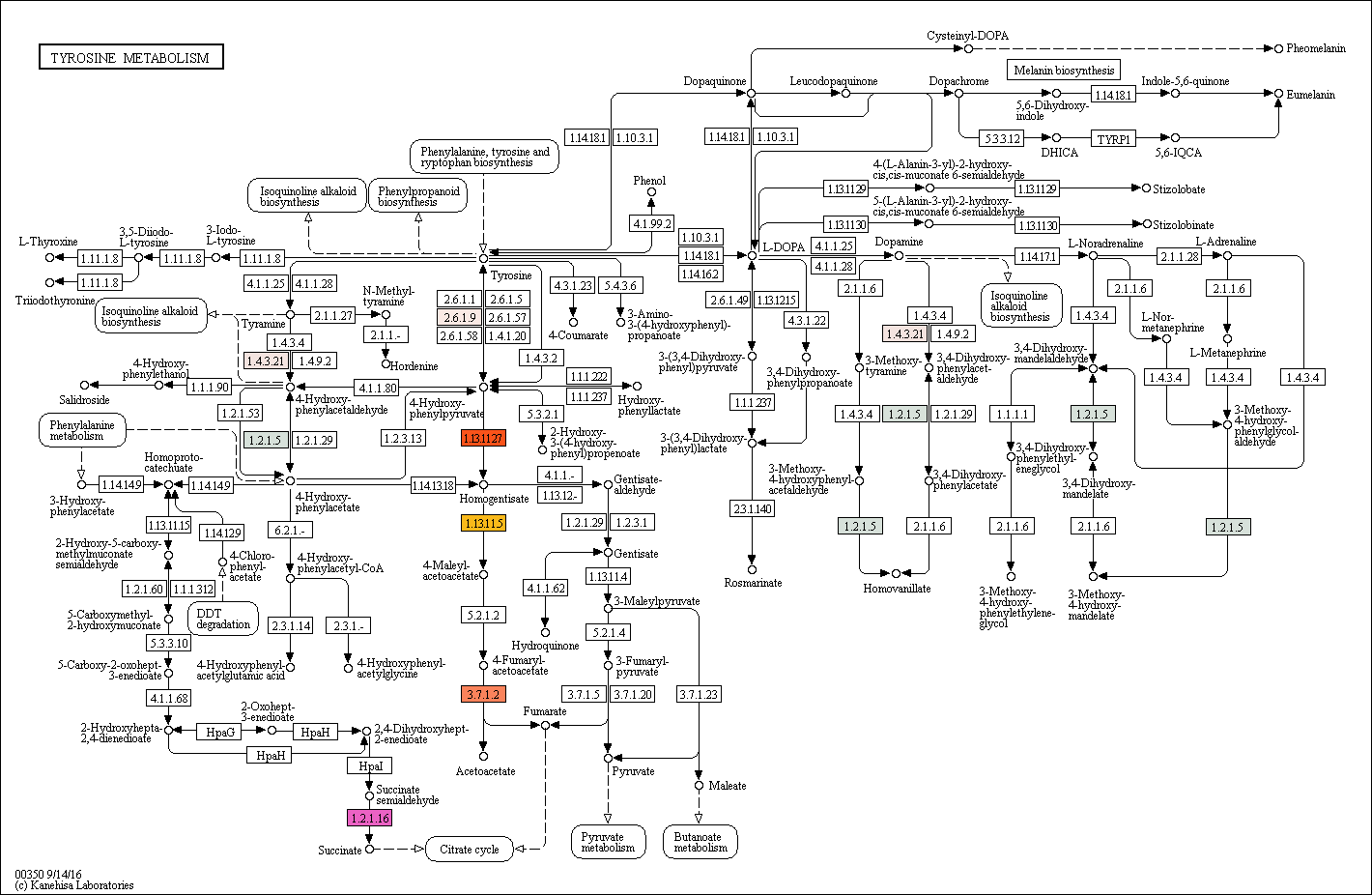

Supplement: Supplementary file 1 [file marinedrugs-16-00207-s001.zip › Supplementary Figures and Tables/Supplementary File 1 _ KEGG pathways/map00350 (Tyrosine metabolism) [7 enz found].png]

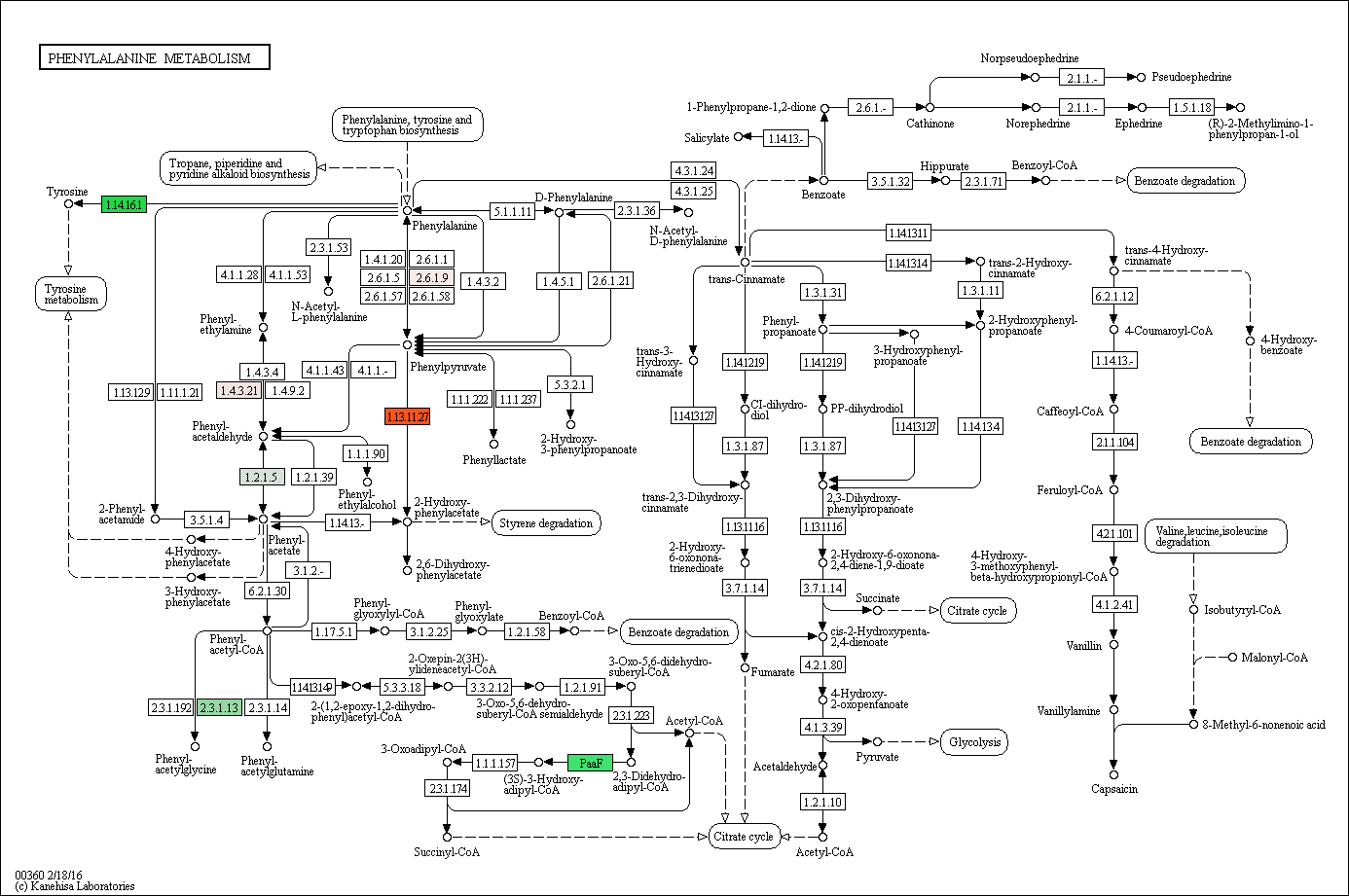

Supplement: Supplementary file 1 [file marinedrugs-16-00207-s001.zip › Supplementary Figures and Tables/Supplementary File 1 _ KEGG pathways/map00360 (Phenylalanine metabolism) [7 enz found].png]

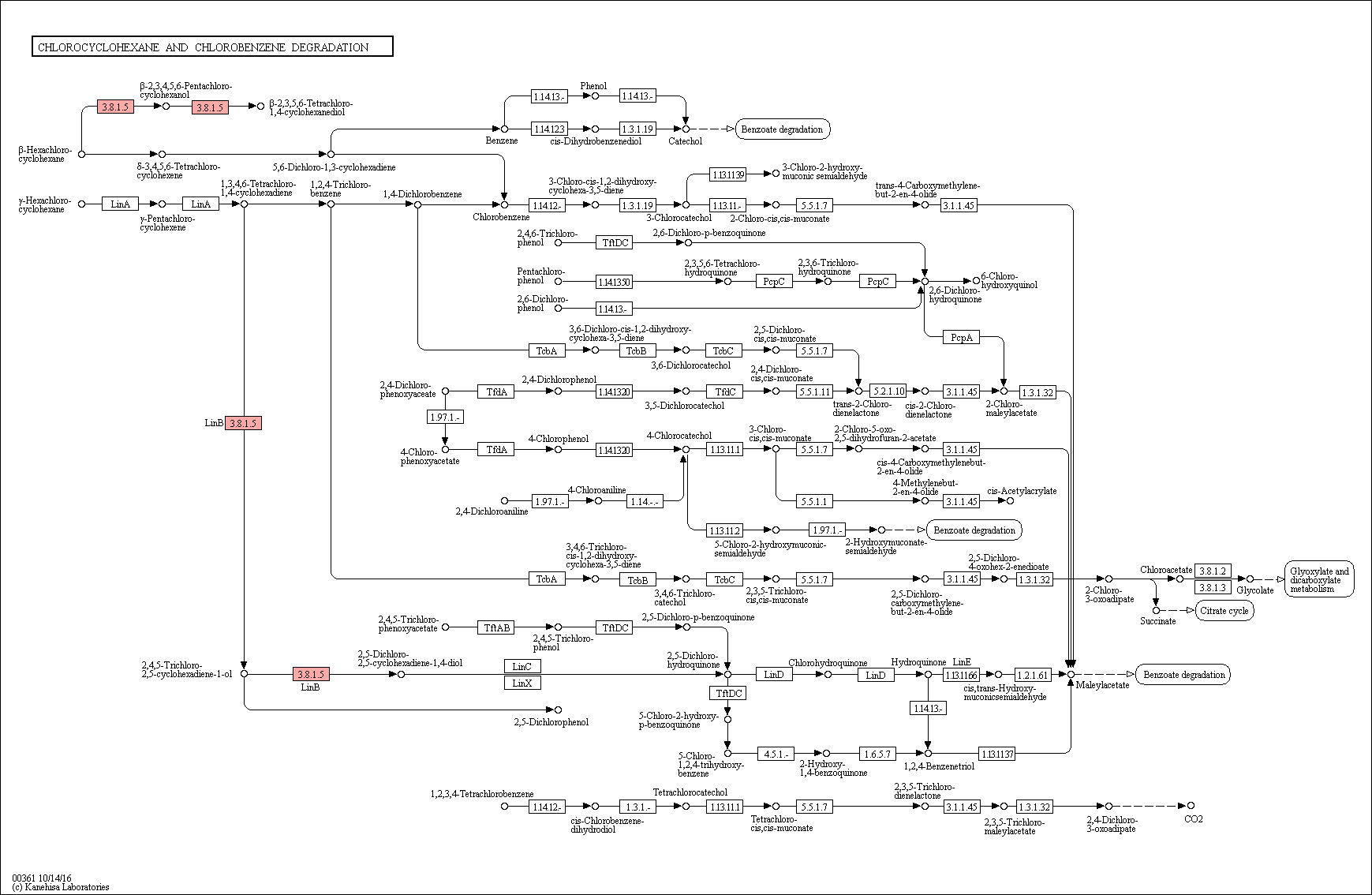

Supplement: Supplementary file 1 [file marinedrugs-16-00207-s001.zip › Supplementary Figures and Tables/Supplementary File 1 _ KEGG pathways/map00361 (Chlorocyclohexane and chlorobenzene degradation) [1 enz found].png]

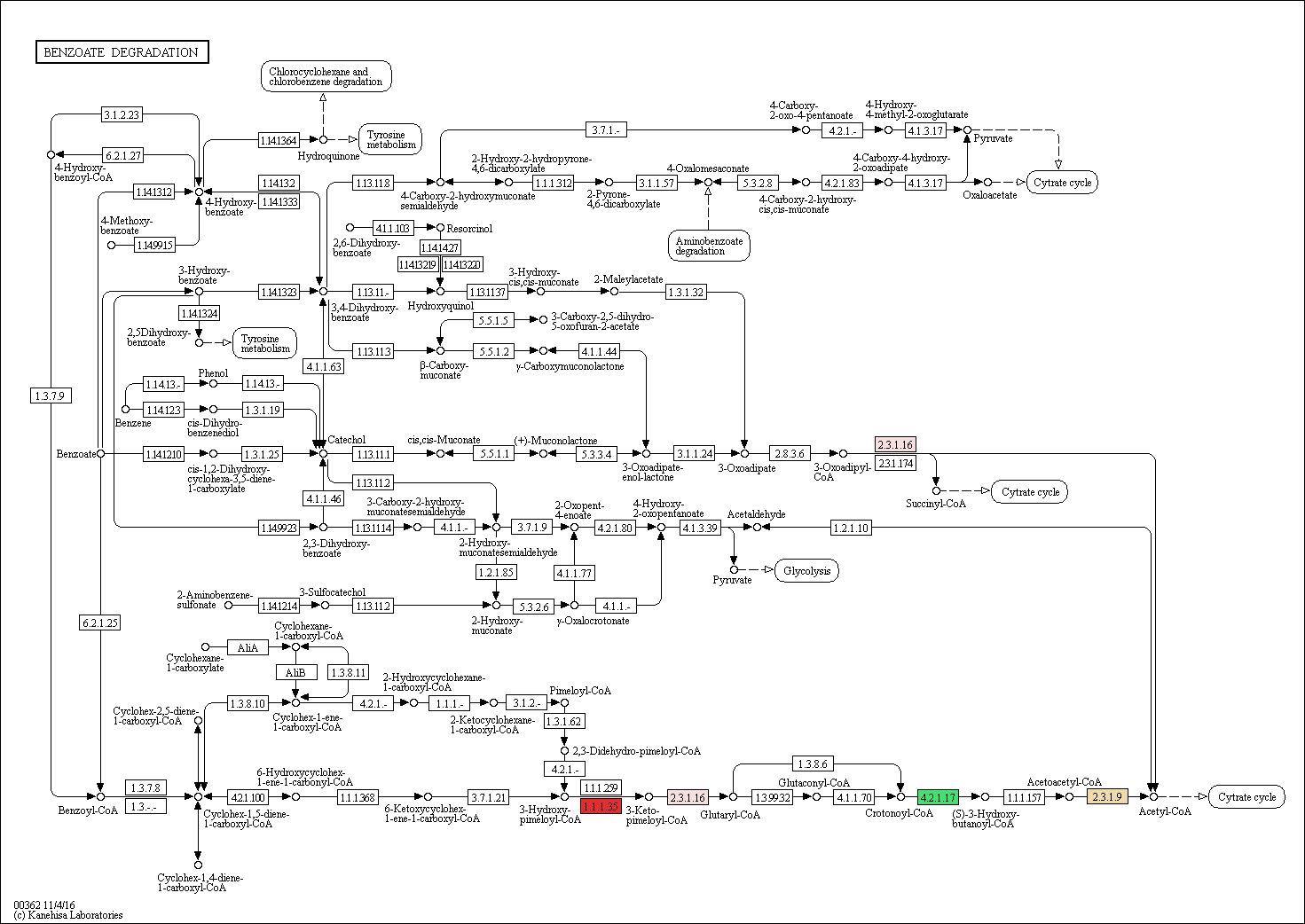

Supplement: Supplementary file 1 [file marinedrugs-16-00207-s001.zip › Supplementary Figures and Tables/Supplementary File 1 _ KEGG pathways/map00362 (Benzoate degradation) [4 enz found].png]

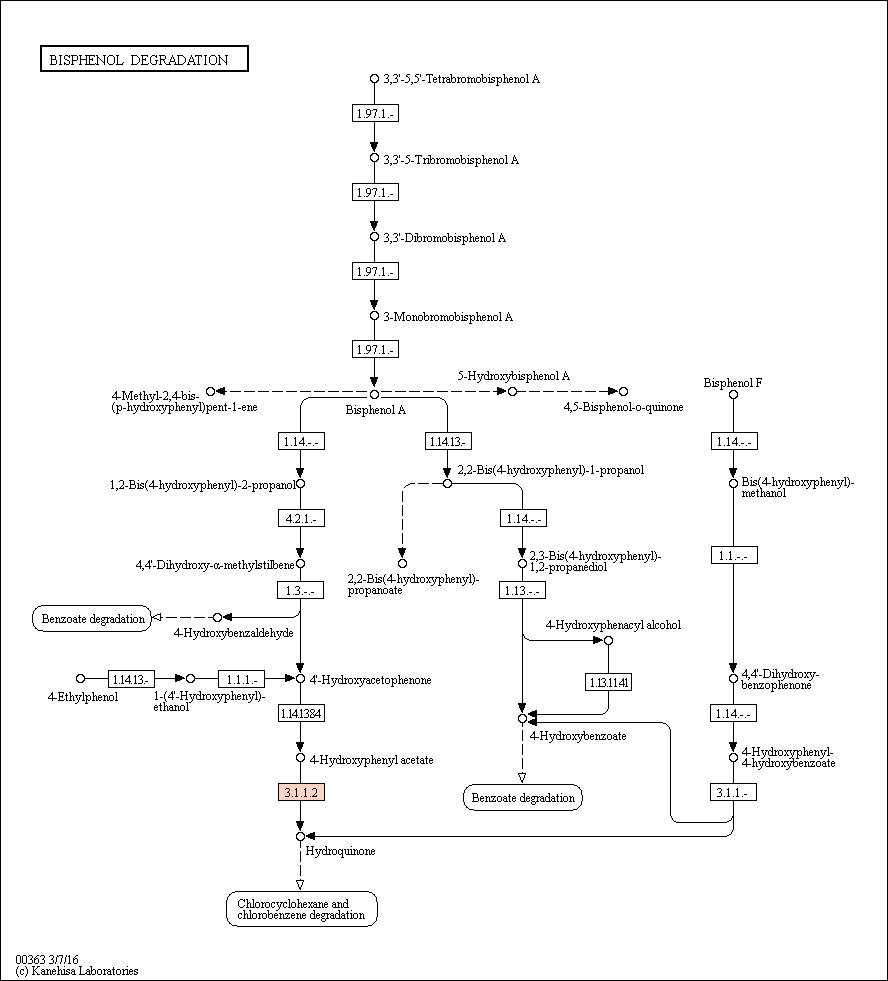

Supplement: Supplementary file 1 [file marinedrugs-16-00207-s001.zip › Supplementary Figures and Tables/Supplementary File 1 _ KEGG pathways/map00363 (Bisphenol degradation) [1 enz found].png]

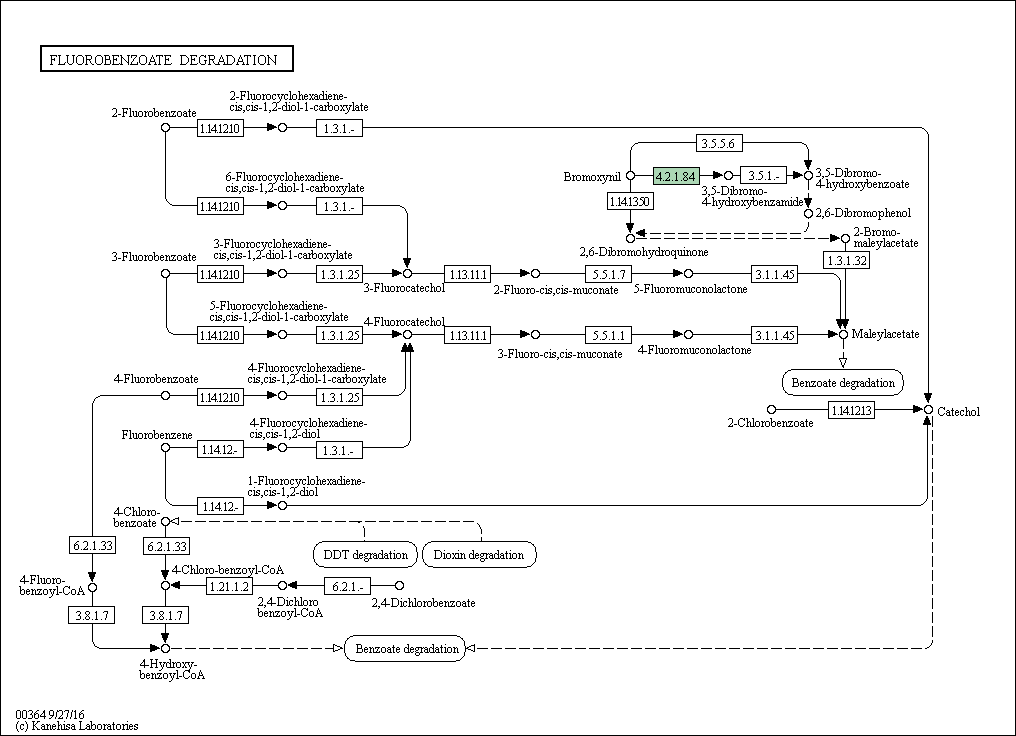

Supplement: Supplementary file 1 [file marinedrugs-16-00207-s001.zip › Supplementary Figures and Tables/Supplementary File 1 _ KEGG pathways/map00364 (Fluorobenzoate degradation) [1 enz found].png]

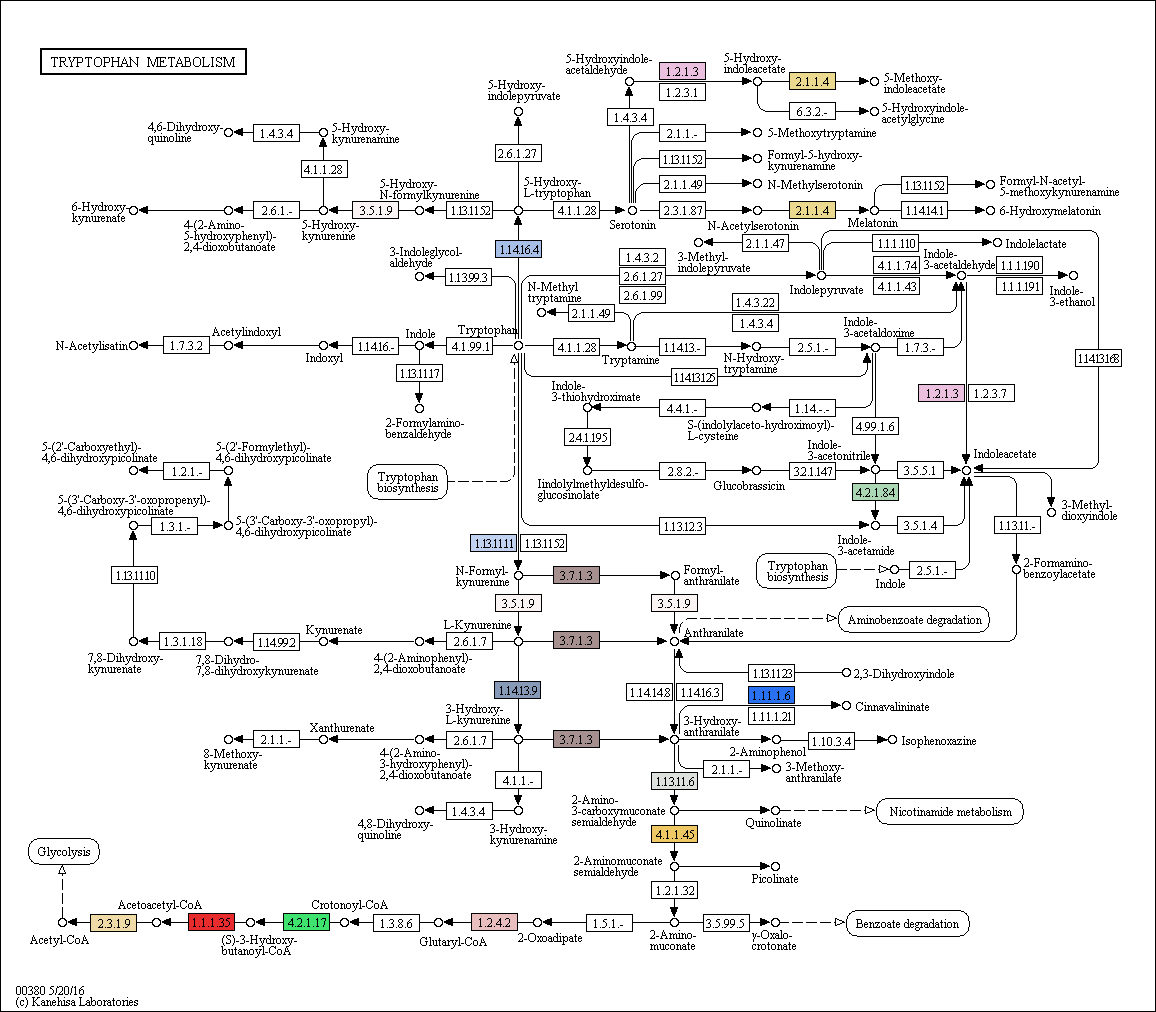

Supplement: Supplementary file 1 [file marinedrugs-16-00207-s001.zip › Supplementary Figures and Tables/Supplementary File 1 _ KEGG pathways/map00380 (Tryptophan metabolism) [15 enz found].png]

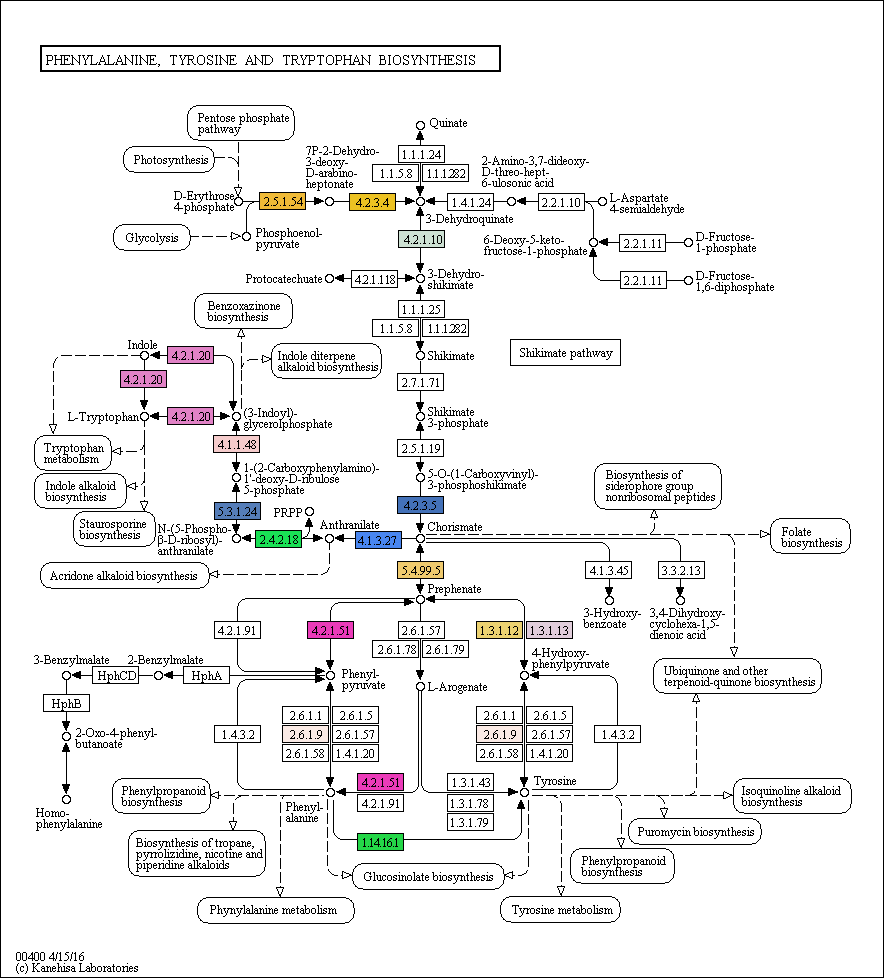

Supplement: Supplementary file 1 [file marinedrugs-16-00207-s001.zip › Supplementary Figures and Tables/Supplementary File 1 _ KEGG pathways/map00400 (Phenylalanine, tyrosin and tryptophan biosynthesis) [15 enz found].png]

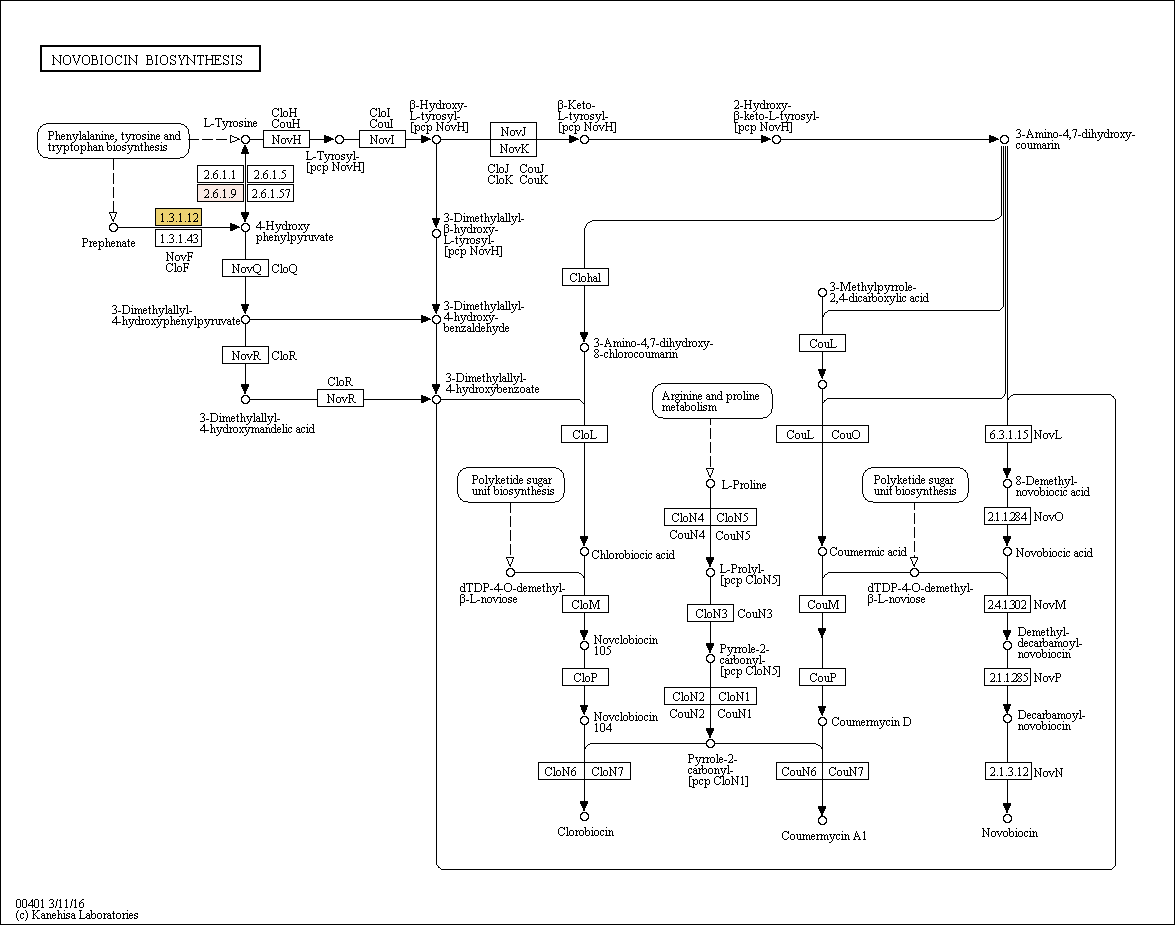

Supplement: Supplementary file 1 [file marinedrugs-16-00207-s001.zip › Supplementary Figures and Tables/Supplementary File 1 _ KEGG pathways/map00401 (Novobiocin biosynthesis) [2 enz found].png]

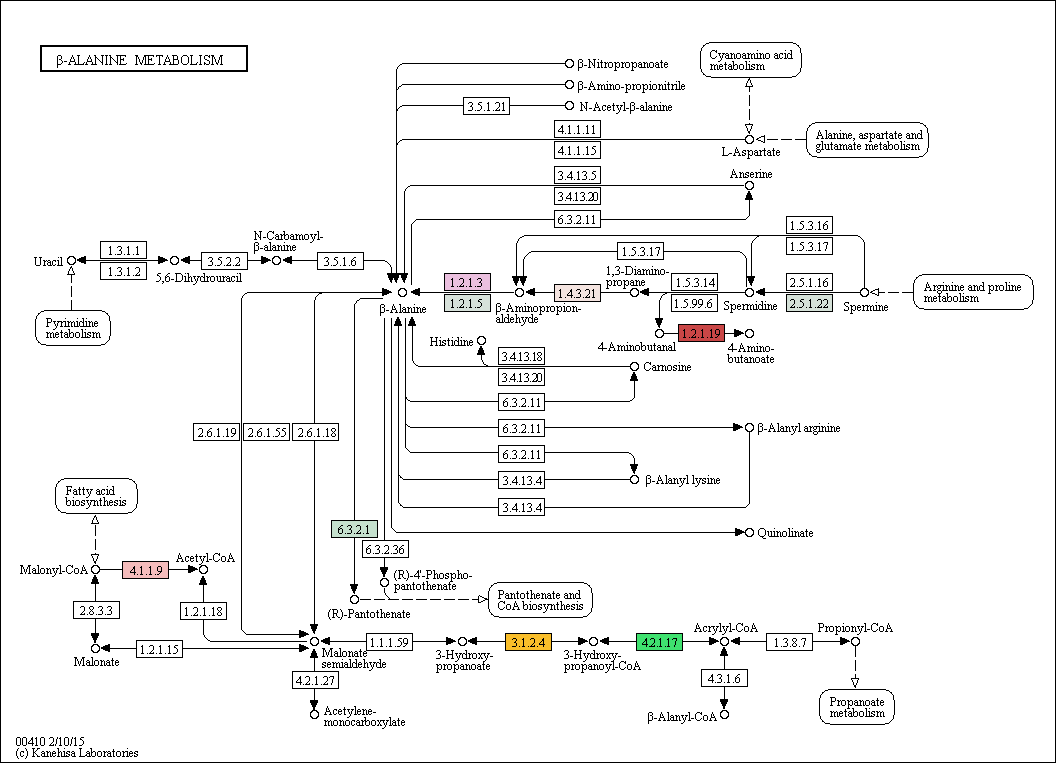

Supplement: Supplementary file 1 [file marinedrugs-16-00207-s001.zip › Supplementary Figures and Tables/Supplementary File 1 _ KEGG pathways/map00410 (Beta-alanine metabolism) [9 enz found].png]

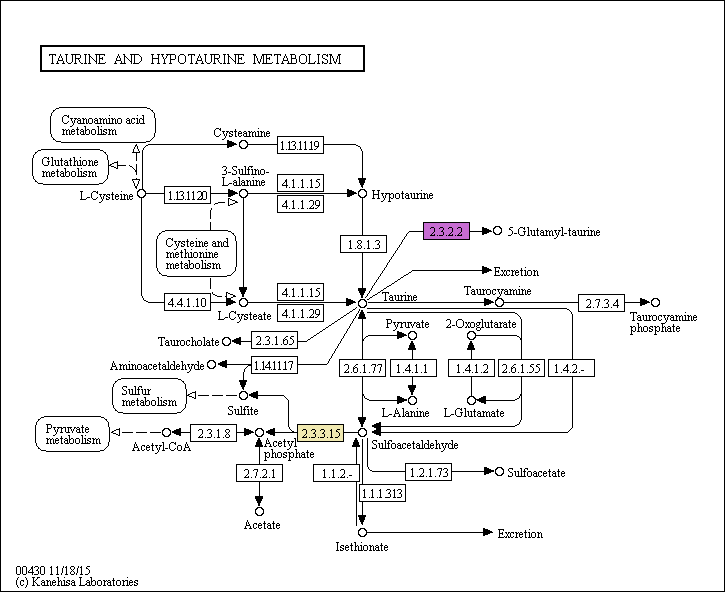

Supplement: Supplementary file 1 [file marinedrugs-16-00207-s001.zip › Supplementary Figures and Tables/Supplementary File 1 _ KEGG pathways/map00430 (Taurine and hypotaurine metabolism) [2 enz found].png]

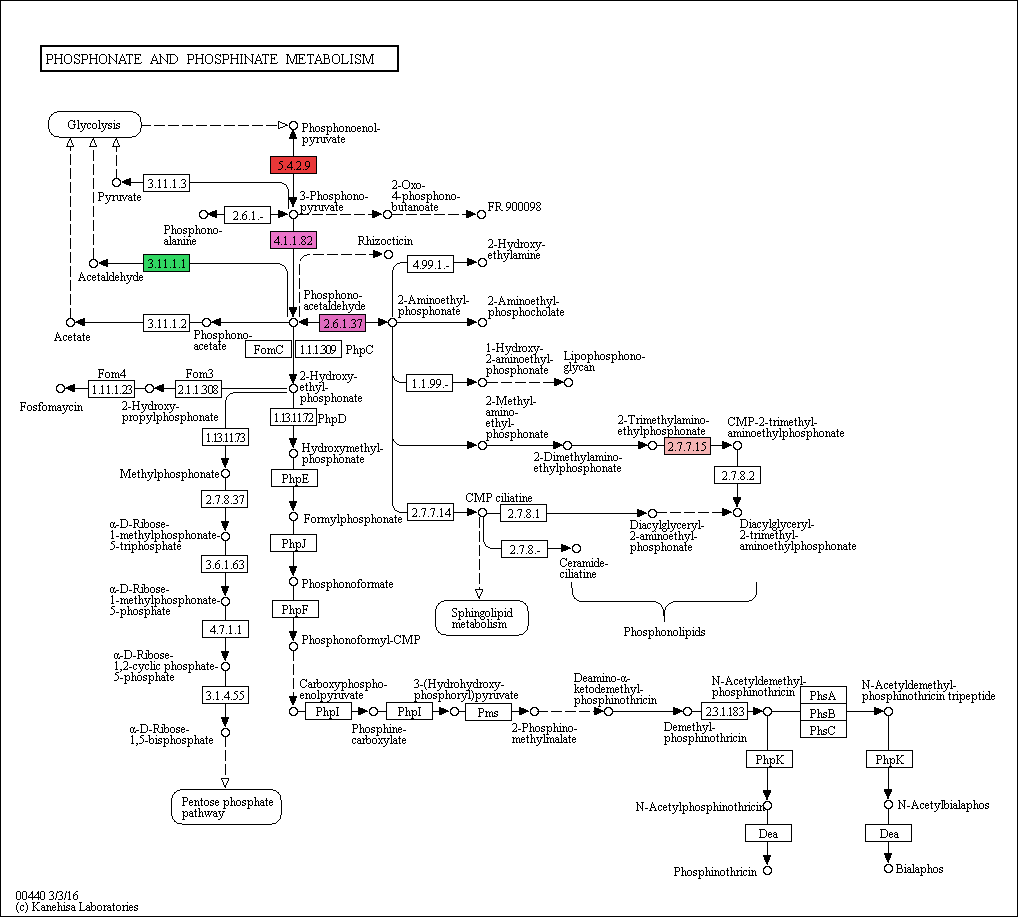

Supplement: Supplementary file 1 [file marinedrugs-16-00207-s001.zip › Supplementary Figures and Tables/Supplementary File 1 _ KEGG pathways/map00440 (Phosphonate and phosphinate metabolism) [5 enz found].png]

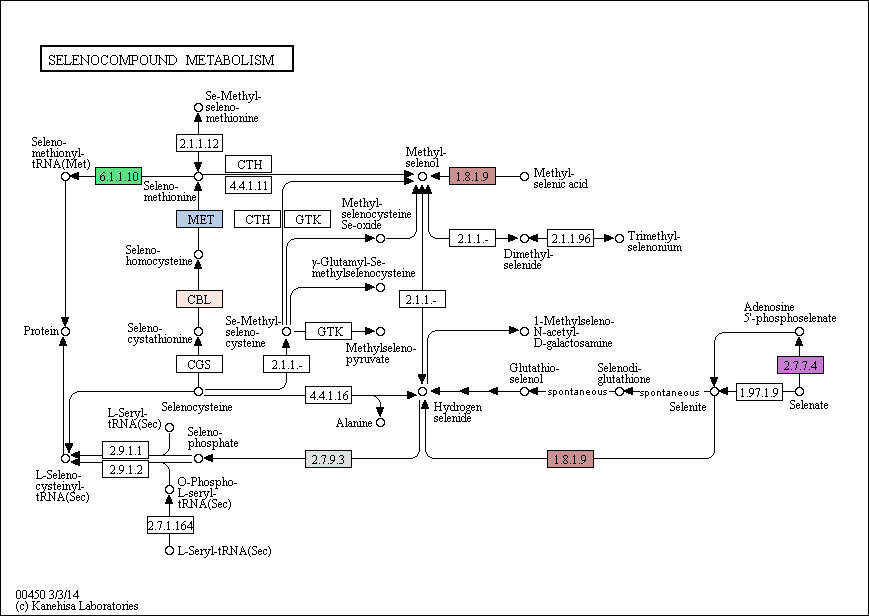

Supplement: Supplementary file 1 [file marinedrugs-16-00207-s001.zip › Supplementary Figures and Tables/Supplementary File 1 _ KEGG pathways/map00450 (Selenocompound metabolism) [7 enz found].png]

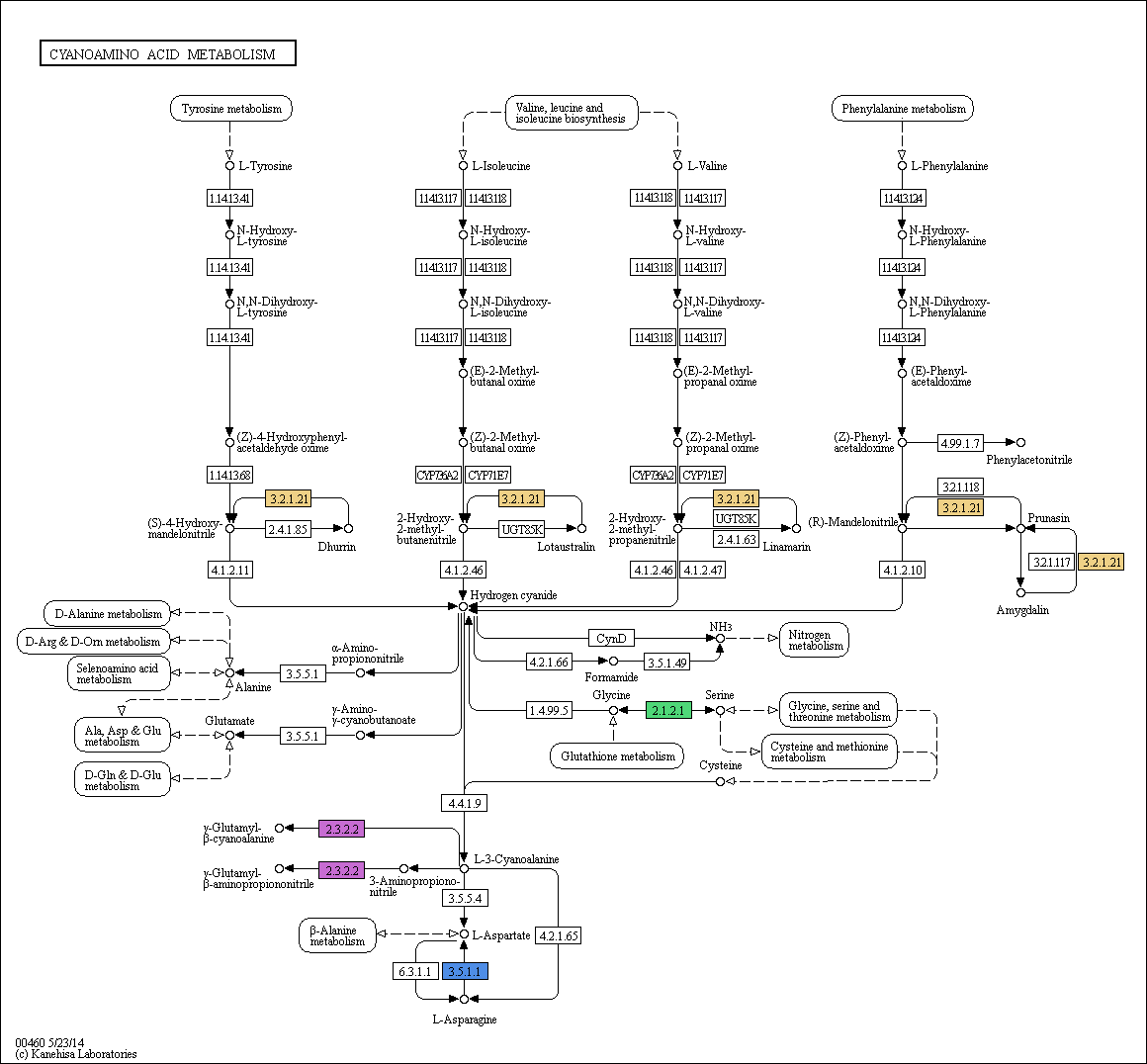

Supplement: Supplementary file 1 [file marinedrugs-16-00207-s001.zip › Supplementary Figures and Tables/Supplementary File 1 _ KEGG pathways/map00460 (Cyanoamino acid metabolism) [4 enz found].png]

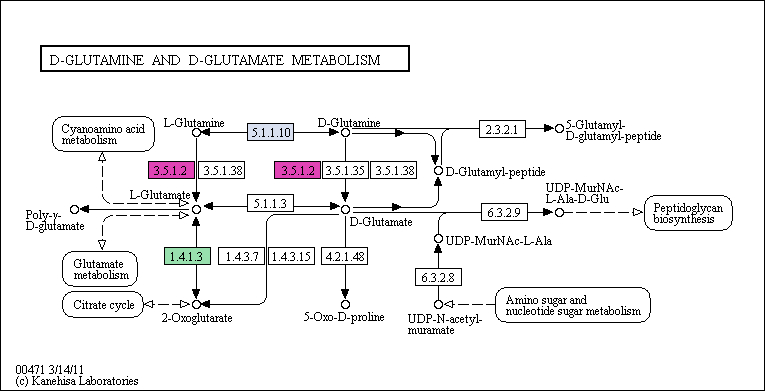

Supplement: Supplementary file 1 [file marinedrugs-16-00207-s001.zip › Supplementary Figures and Tables/Supplementary File 1 _ KEGG pathways/map00471 (D-glutamine and D-glutamate metabolism) [3 enz found].png]

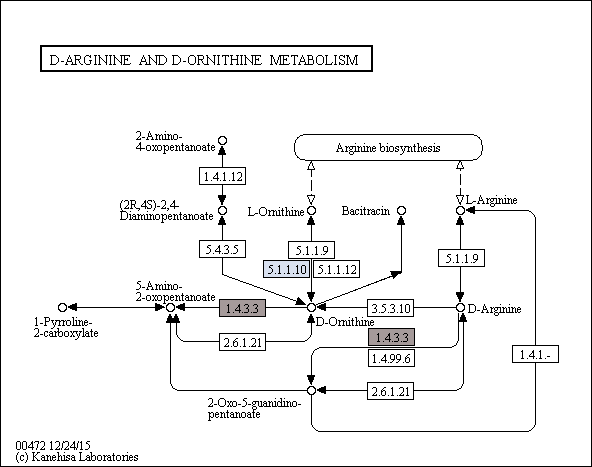

Supplement: Supplementary file 1 [file marinedrugs-16-00207-s001.zip › Supplementary Figures and Tables/Supplementary File 1 _ KEGG pathways/map00472 (D-arginine and D-ornithine metabolism) [2 enz found].png]

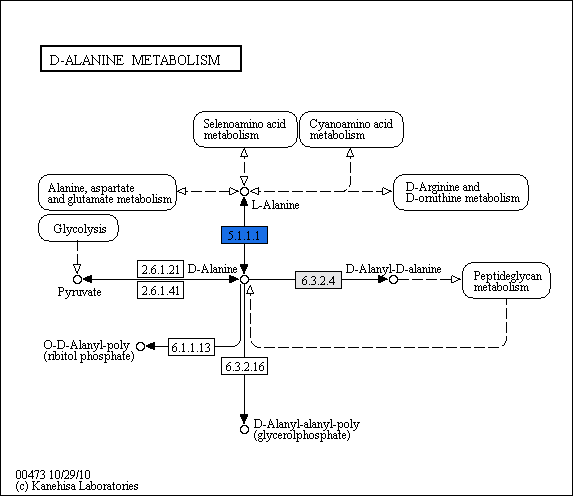

Supplement: Supplementary file 1 [file marinedrugs-16-00207-s001.zip › Supplementary Figures and Tables/Supplementary File 1 _ KEGG pathways/map00473 (D-alanine metabolism) [2 enz found].png]

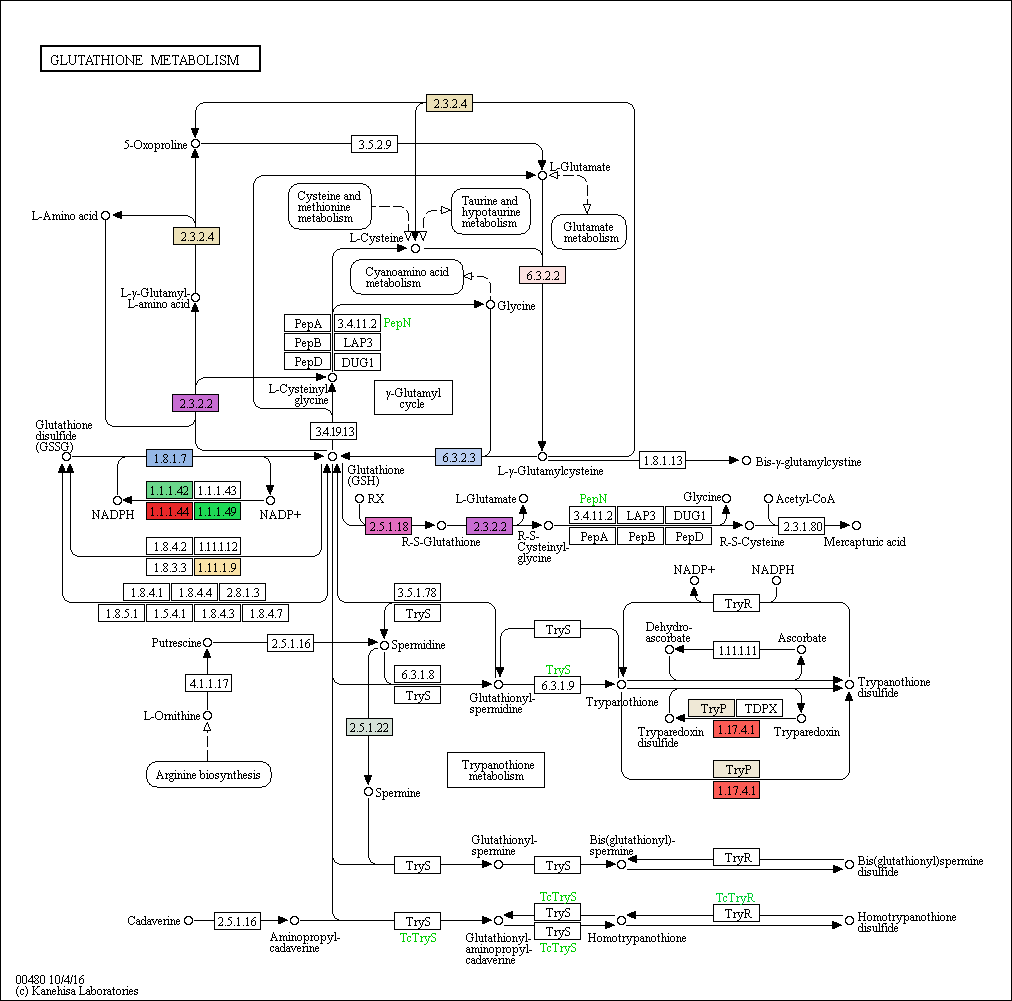

Supplement: Supplementary file 1 [file marinedrugs-16-00207-s001.zip › Supplementary Figures and Tables/Supplementary File 1 _ KEGG pathways/map00480 (Glutathione metabolism) [13 enz found].png]

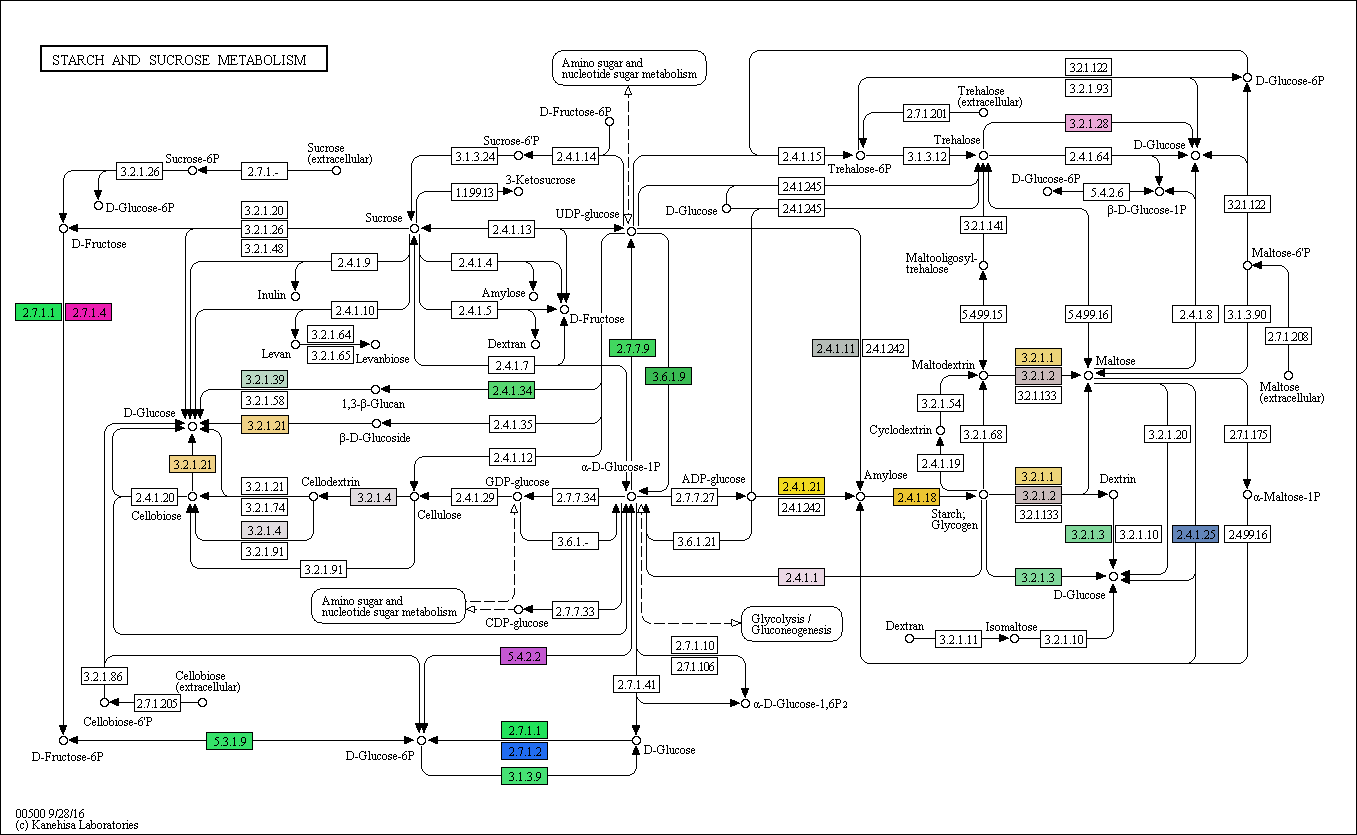

Supplement: Supplementary file 1 [file marinedrugs-16-00207-s001.zip › Supplementary Figures and Tables/Supplementary File 1 _ KEGG pathways/map00500 (Starch and sucrose metabolism) [21 enz found].png]

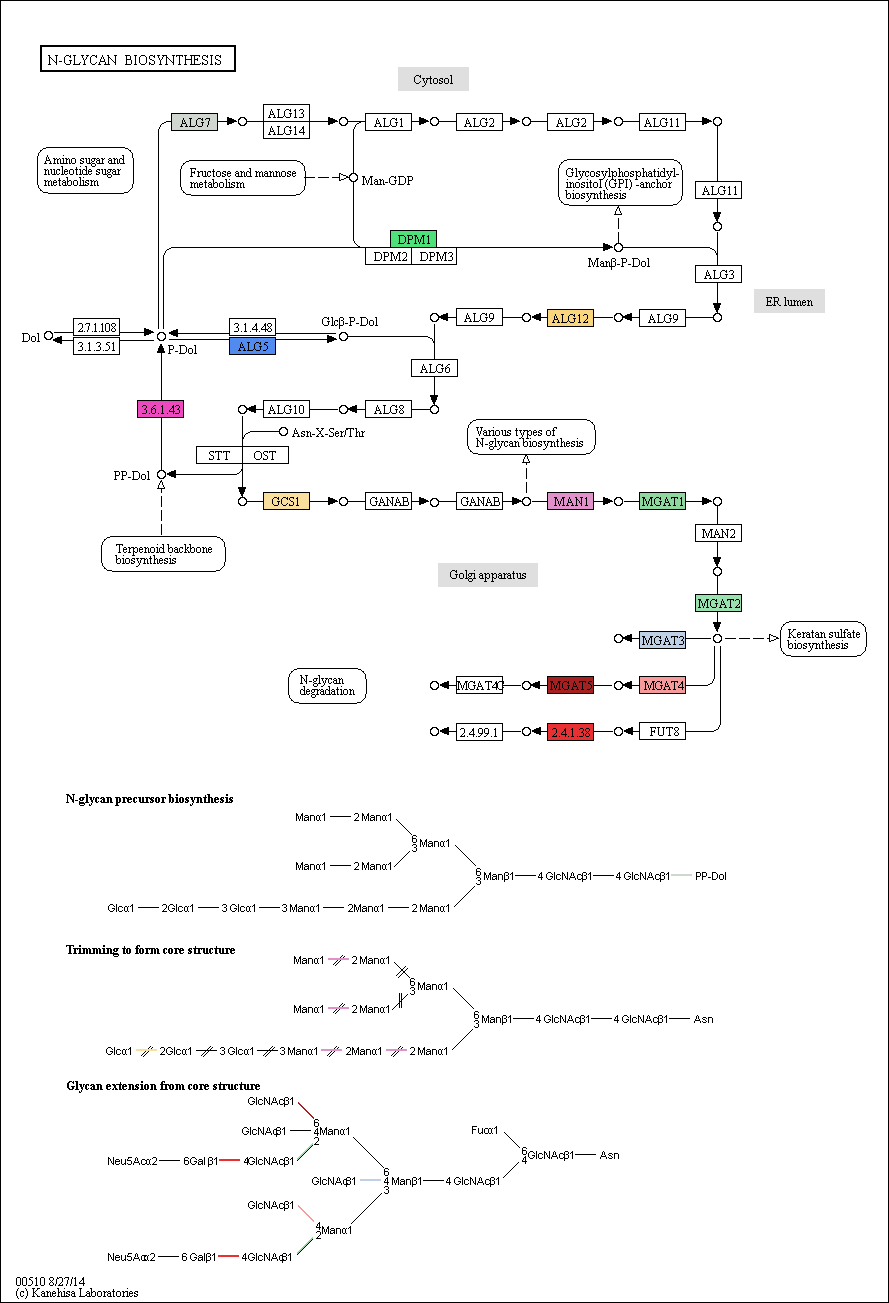

Supplement: Supplementary file 1 [file marinedrugs-16-00207-s001.zip › Supplementary Figures and Tables/Supplementary File 1 _ KEGG pathways/map00510 (N-glycan biosynthesis) [13 enz found].png]

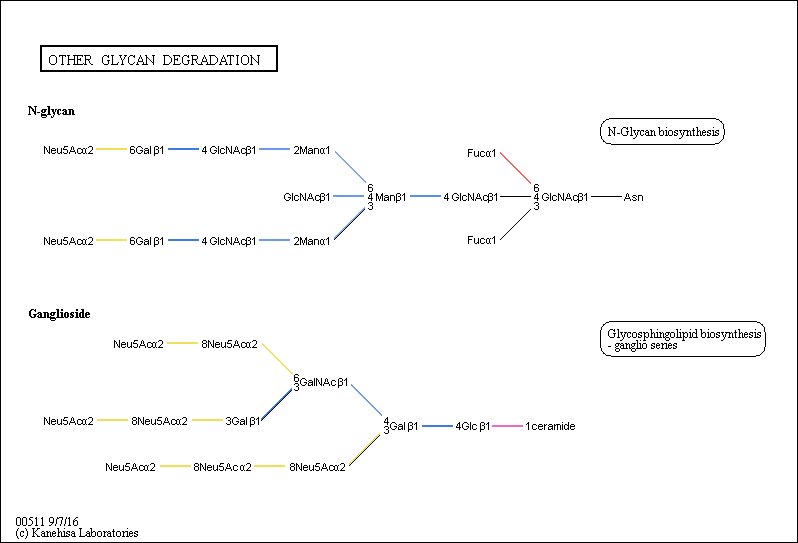

Supplement: Supplementary file 1 [file marinedrugs-16-00207-s001.zip › Supplementary Figures and Tables/Supplementary File 1 _ KEGG pathways/map00511 (Other glycan degradation) [7 enz found].png]

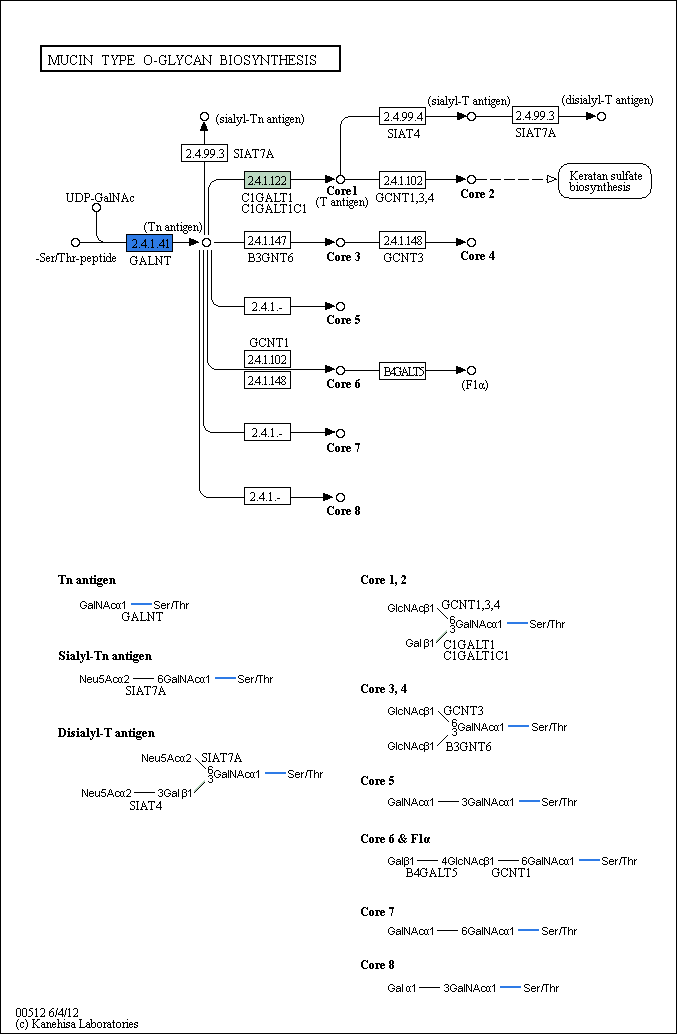

Supplement: Supplementary file 1 [file marinedrugs-16-00207-s001.zip › Supplementary Figures and Tables/Supplementary File 1 _ KEGG pathways/map00512 (Mucin type O-glycan biosynthesis) [2 enz found].png]

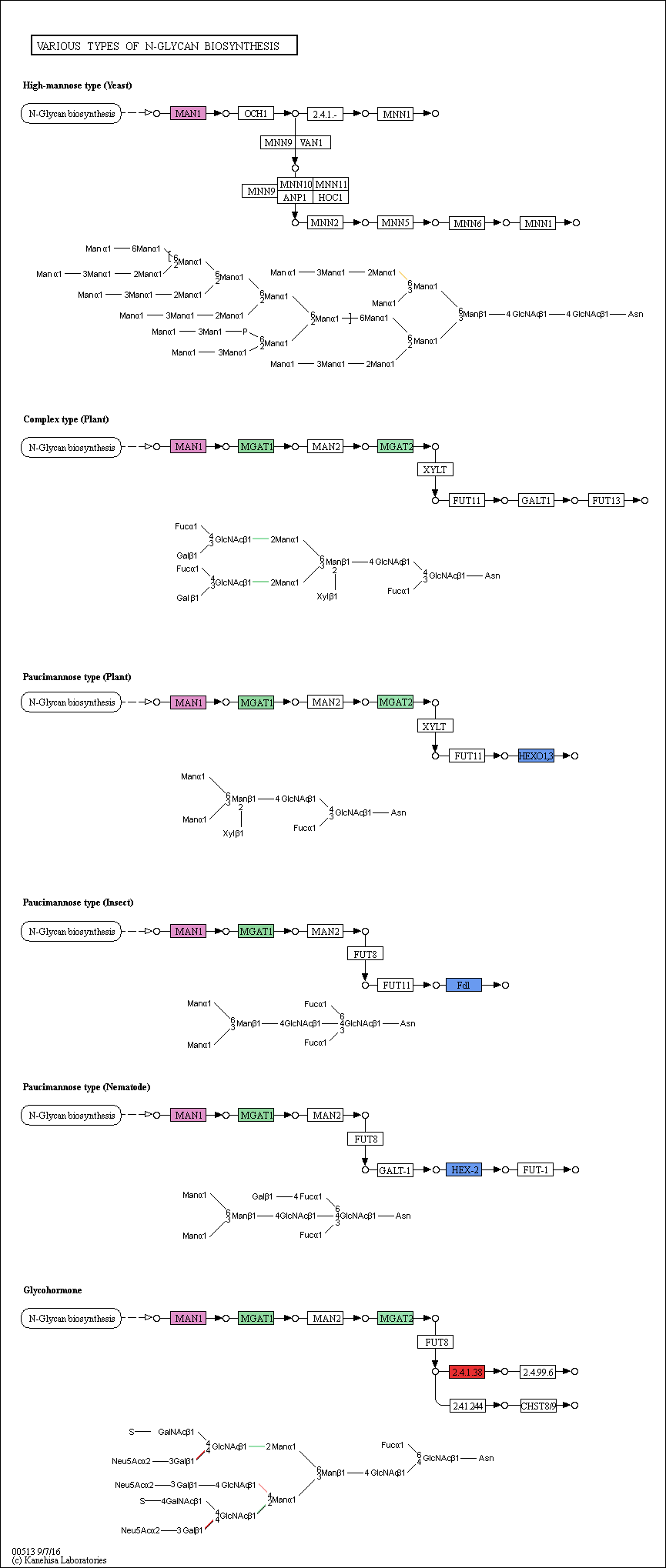

Supplement: Supplementary file 1 [file marinedrugs-16-00207-s001.zip › Supplementary Figures and Tables/Supplementary File 1 _ KEGG pathways/map00513 (Various type of N-glycan biosynthesis) [7 enz found].png]

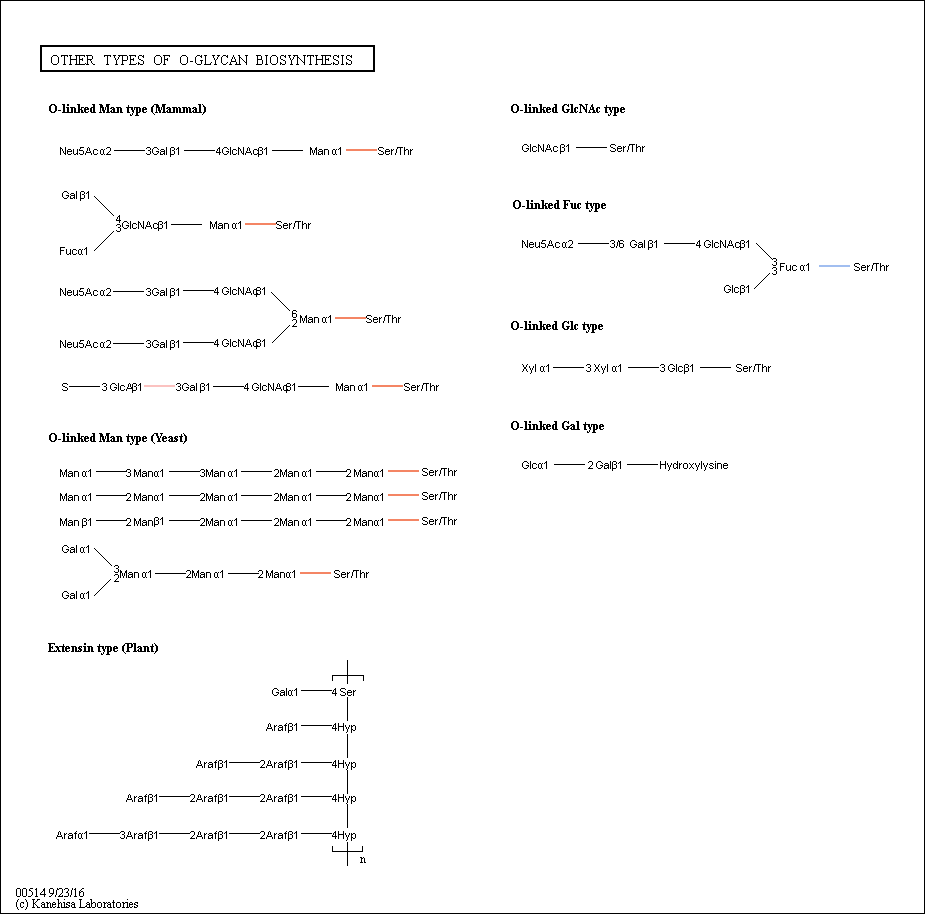

Supplement: Supplementary file 1 [file marinedrugs-16-00207-s001.zip › Supplementary Figures and Tables/Supplementary File 1 _ KEGG pathways/map00514 (Other types of O-glycan biosynthesis) [3 enz found].png]

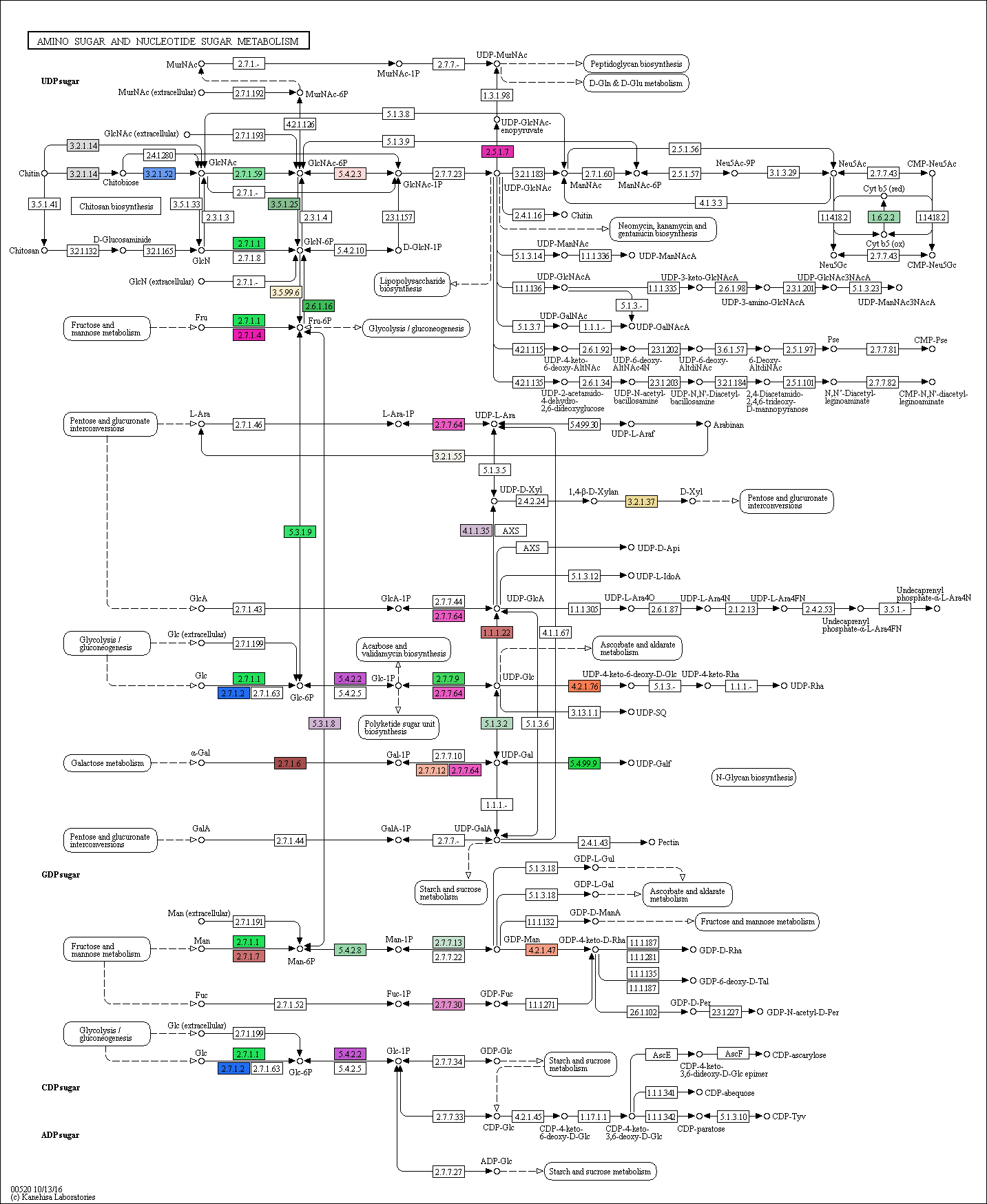

Supplement: Supplementary file 1 [file marinedrugs-16-00207-s001.zip › Supplementary Figures and Tables/Supplementary File 1 _ KEGG pathways/map00520 (Amino sugar and nucleotide sugar metabolism) [31 enz found].png]

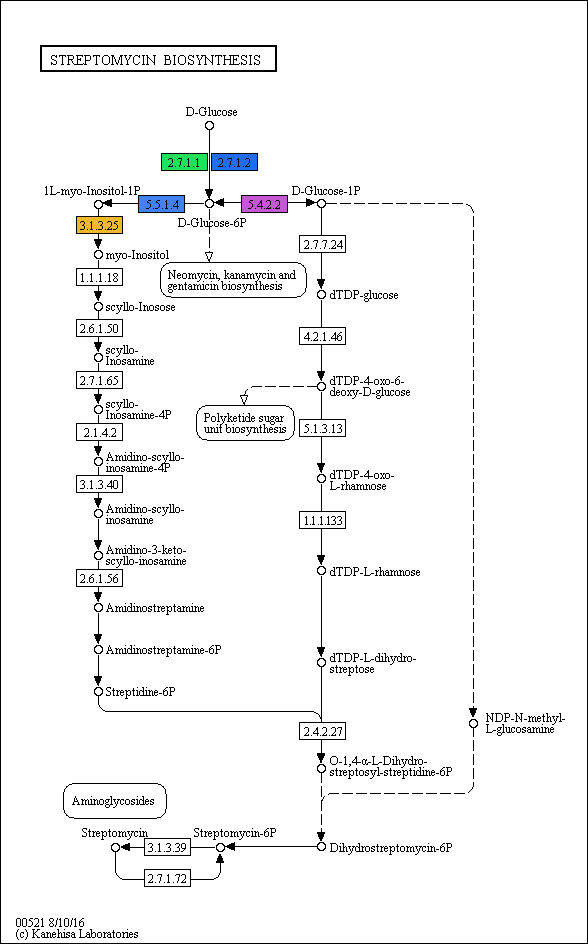

Supplement: Supplementary file 1 [file marinedrugs-16-00207-s001.zip › Supplementary Figures and Tables/Supplementary File 1 _ KEGG pathways/map00521 (Streptomycin biosynthesis) [5 enz found].png]

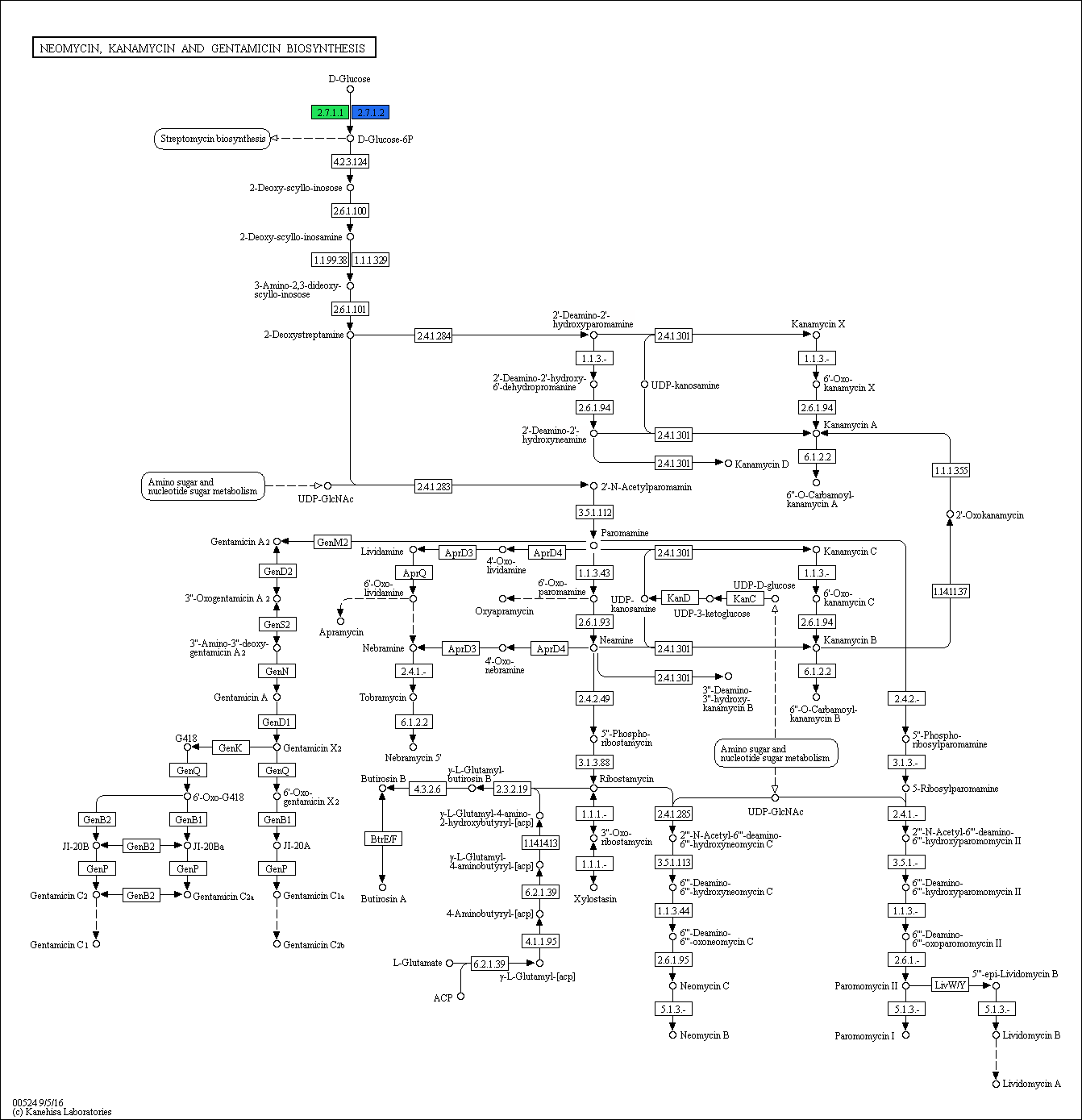

Supplement: Supplementary file 1 [file marinedrugs-16-00207-s001.zip › Supplementary Figures and Tables/Supplementary File 1 _ KEGG pathways/map00524 (Neomycin, kanamycin and gentamicin biosynthesis) [2 enz found].png]

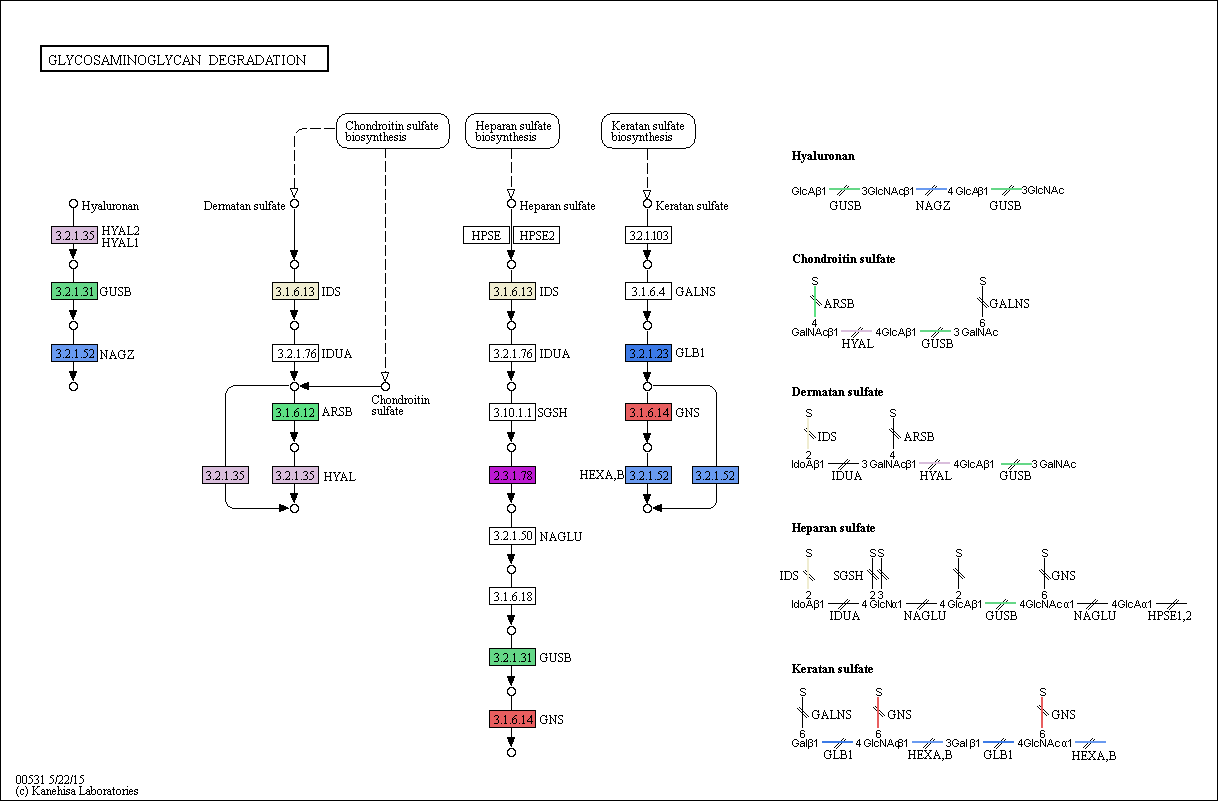

Supplement: Supplementary file 1 [file marinedrugs-16-00207-s001.zip › Supplementary Figures and Tables/Supplementary File 1 _ KEGG pathways/map00531 (Glycosaminoglycan degradation) [8 enz found].png]

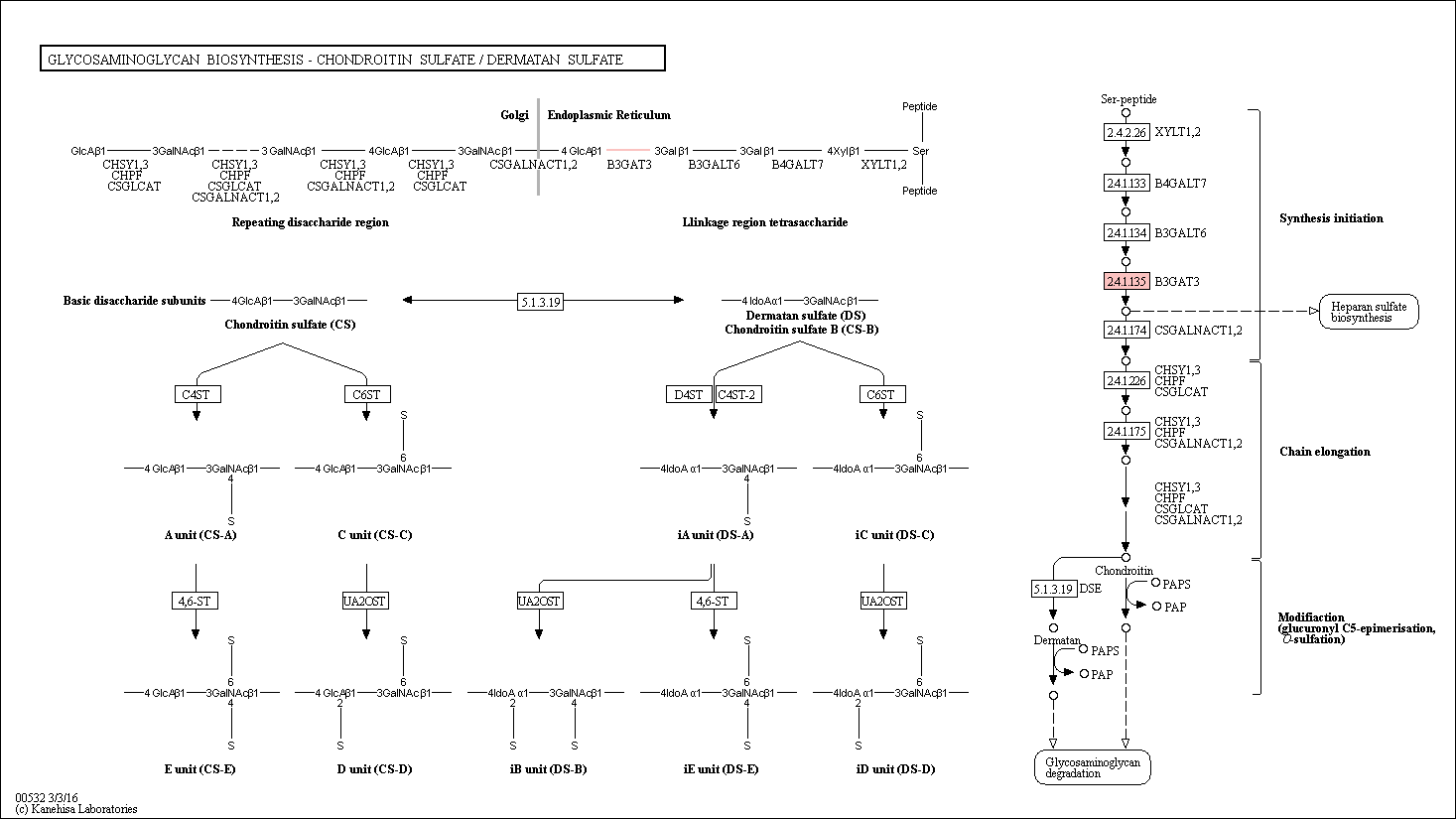

Supplement: Supplementary file 1 [file marinedrugs-16-00207-s001.zip › Supplementary Figures and Tables/Supplementary File 1 _ KEGG pathways/map00532 (Glycosaminaglycan biosynthesis) [1 enz found].png]

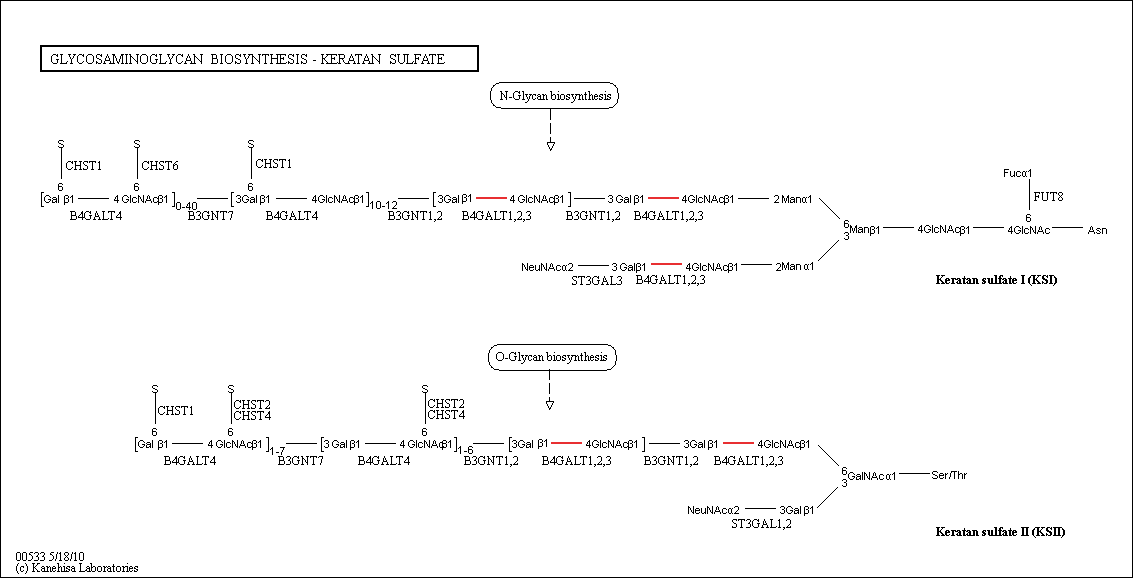

Supplement: Supplementary file 1 [file marinedrugs-16-00207-s001.zip › Supplementary Figures and Tables/Supplementary File 1 _ KEGG pathways/map00533 (Glycosaminoglycan biosynthesis) [1 enz found].png]

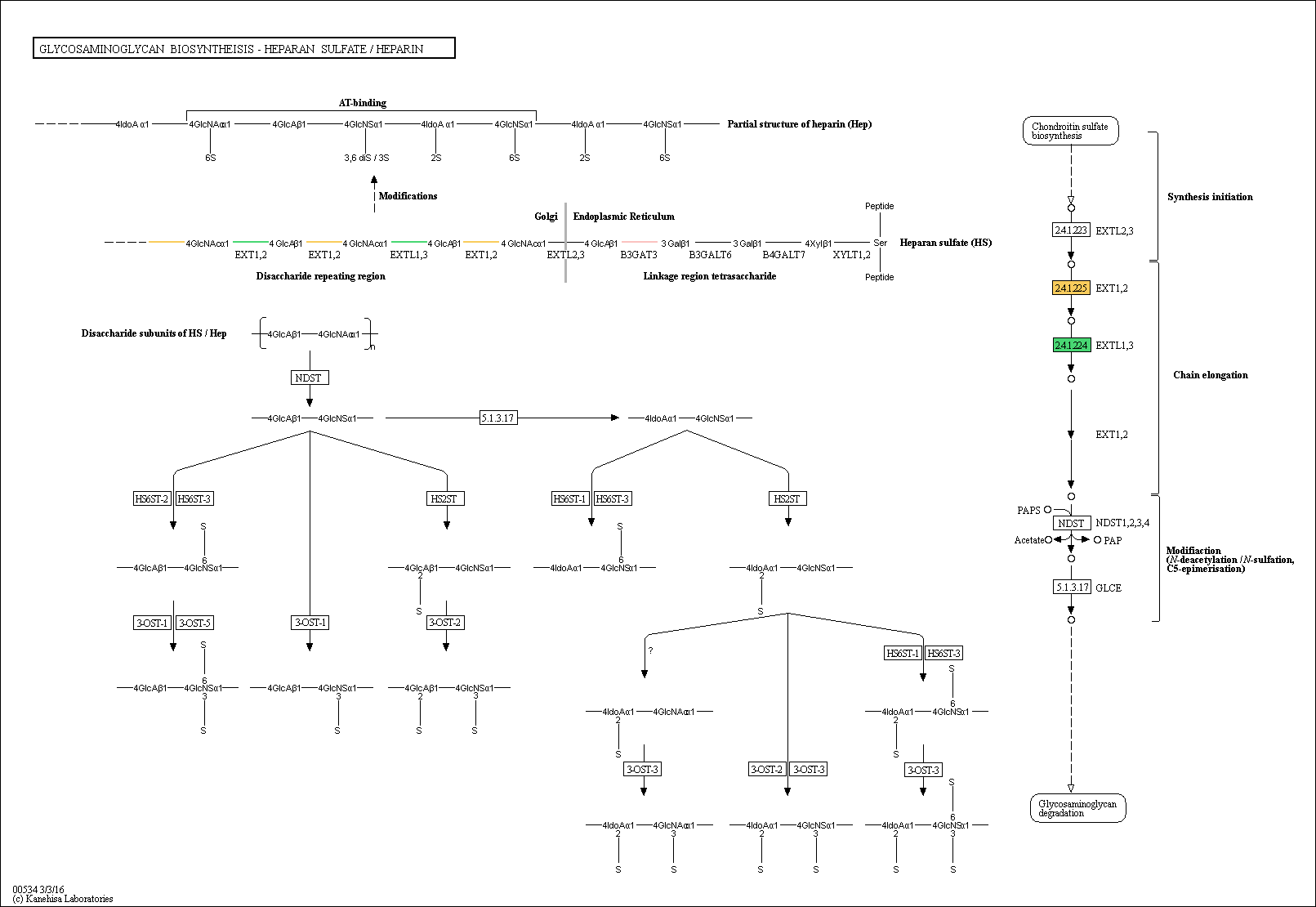

Supplement: Supplementary file 1 [file marinedrugs-16-00207-s001.zip › Supplementary Figures and Tables/Supplementary File 1 _ KEGG pathways/map00534 (Glycosaminoglycan biosynthesis) [1 enz found].png]

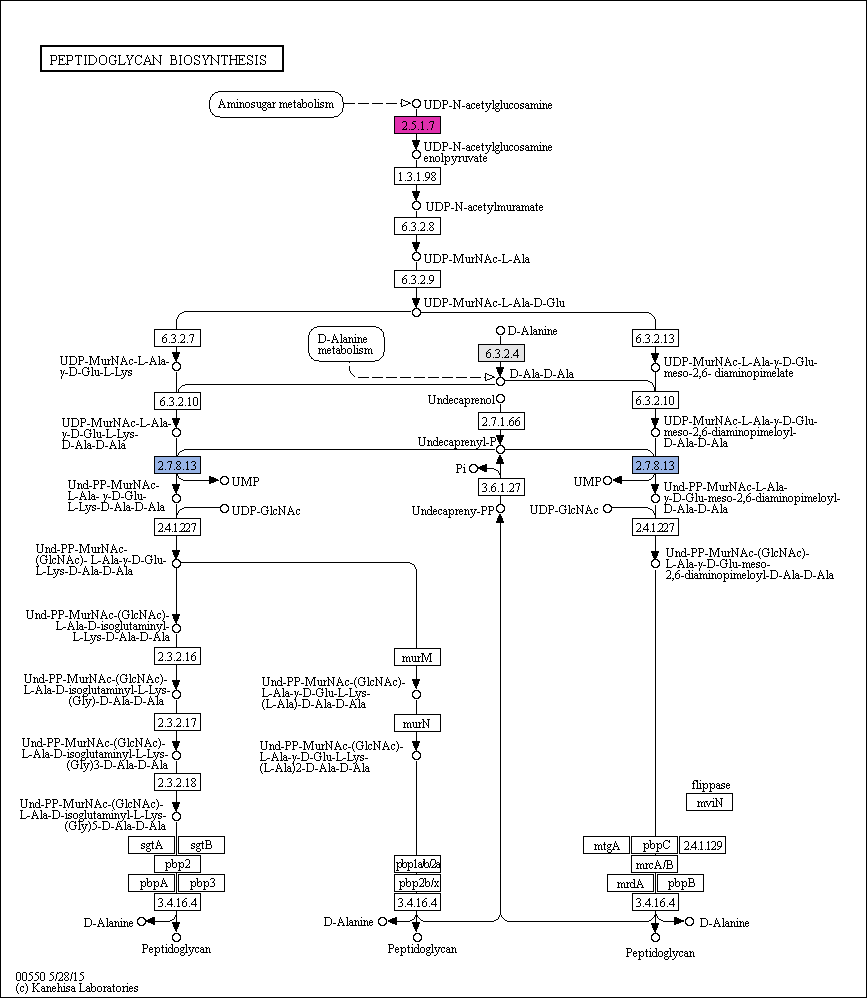

Supplement: Supplementary file 1 [file marinedrugs-16-00207-s001.zip › Supplementary Figures and Tables/Supplementary File 1 _ KEGG pathways/map00550 (Peptidoglycan biosynthesis) [3 enz found].png]

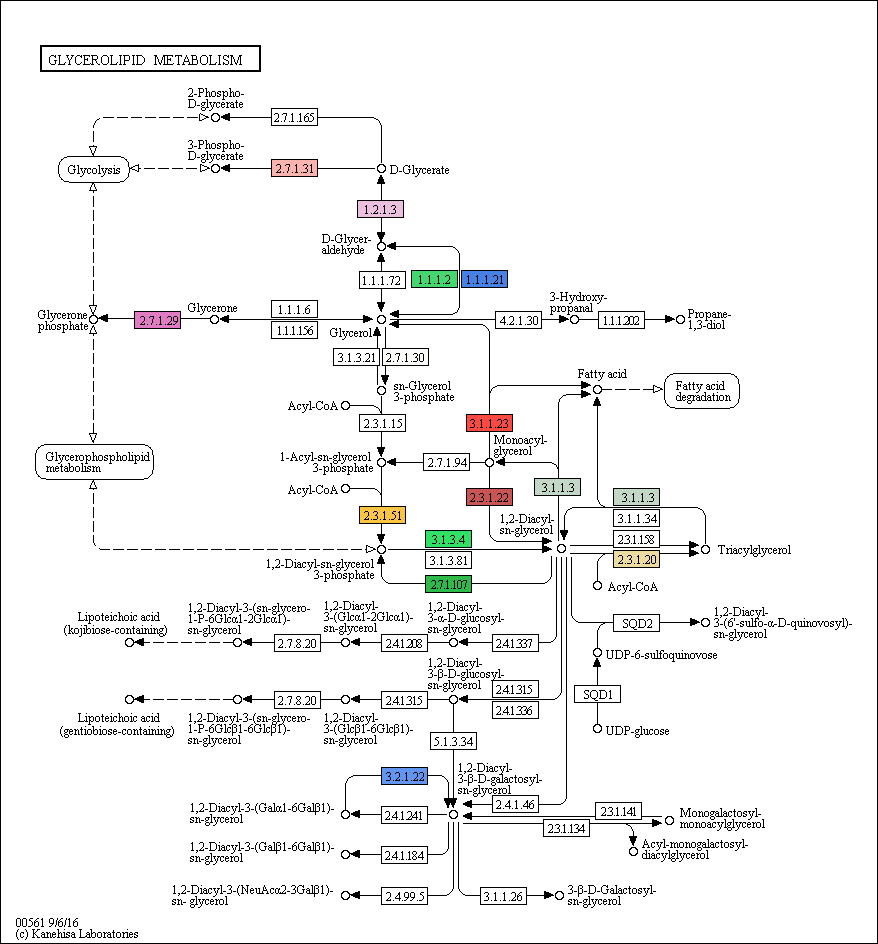

Supplement: Supplementary file 1 [file marinedrugs-16-00207-s001.zip › Supplementary Figures and Tables/Supplementary File 1 _ KEGG pathways/map00561 (Glycerolipid metabolism) [14 enz found].png]

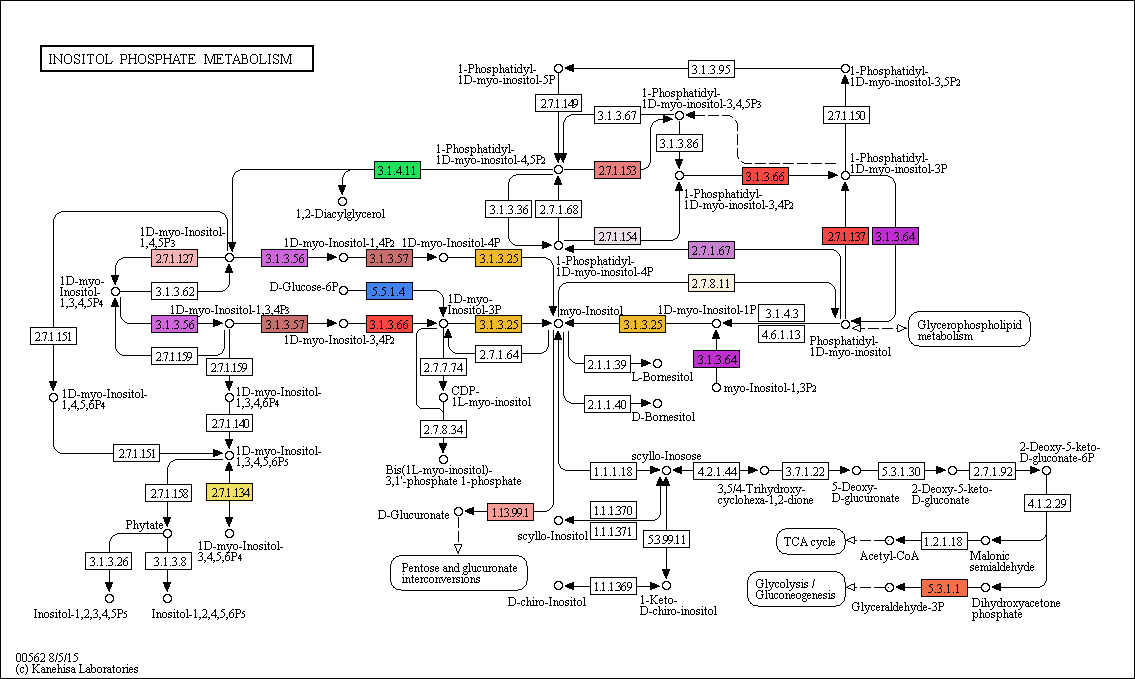

Supplement: Supplementary file 1 [file marinedrugs-16-00207-s001.zip › Supplementary Figures and Tables/Supplementary File 1 _ KEGG pathways/map00562 (Inositol phosphate metabolism) [17 enz found].png]

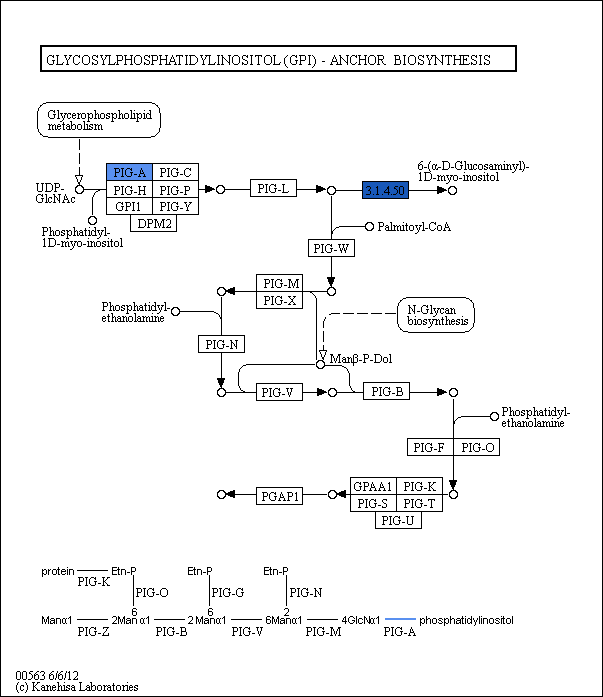

Supplement: Supplementary file 1 [file marinedrugs-16-00207-s001.zip › Supplementary Figures and Tables/Supplementary File 1 _ KEGG pathways/map00563 (GPI anchor biosynthesis) [2 enz found].png]

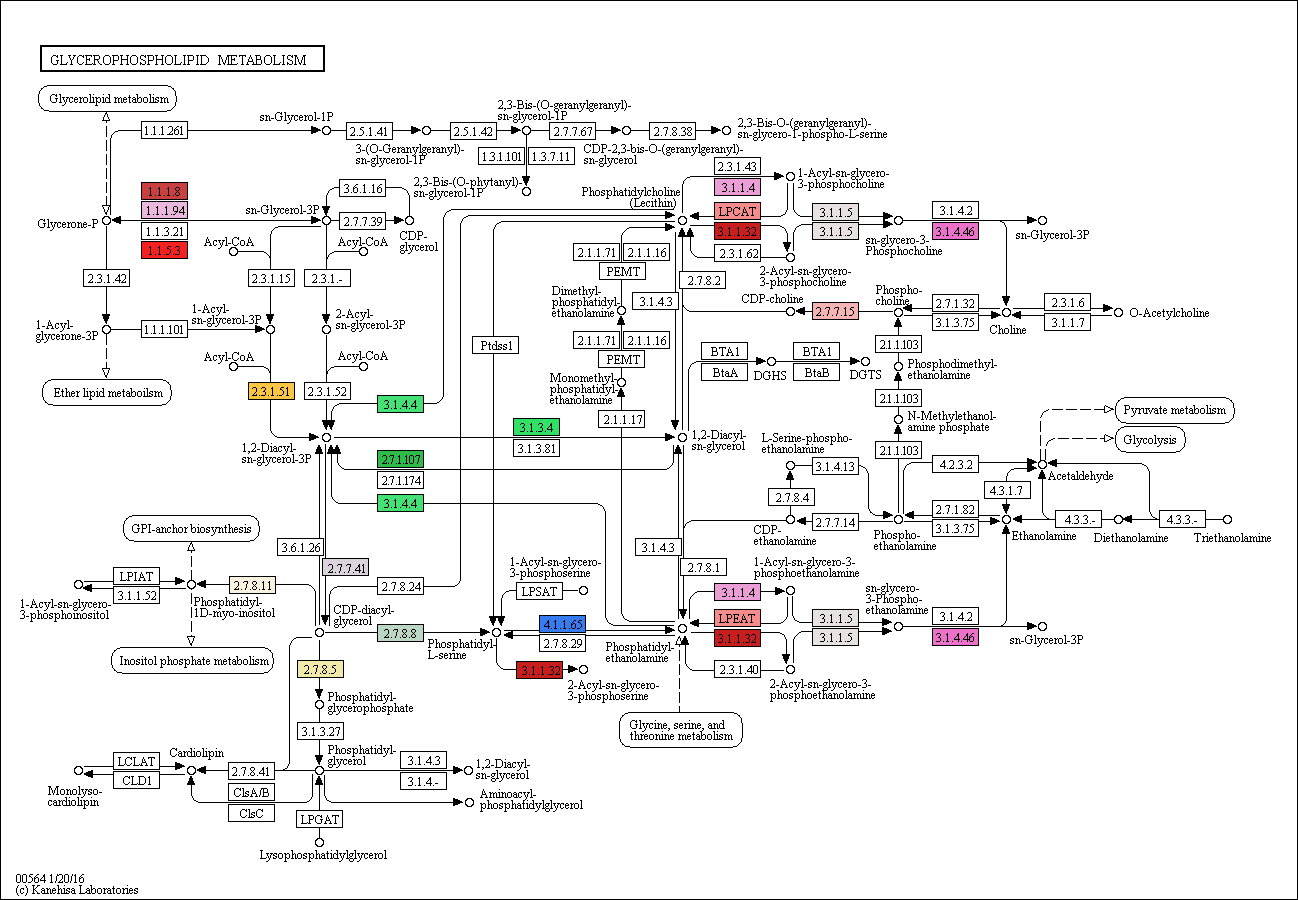

Supplement: Supplementary file 1 [file marinedrugs-16-00207-s001.zip › Supplementary Figures and Tables/Supplementary File 1 _ KEGG pathways/map00564 (Glycerophospholipid metabolism) [18 enz found].png]

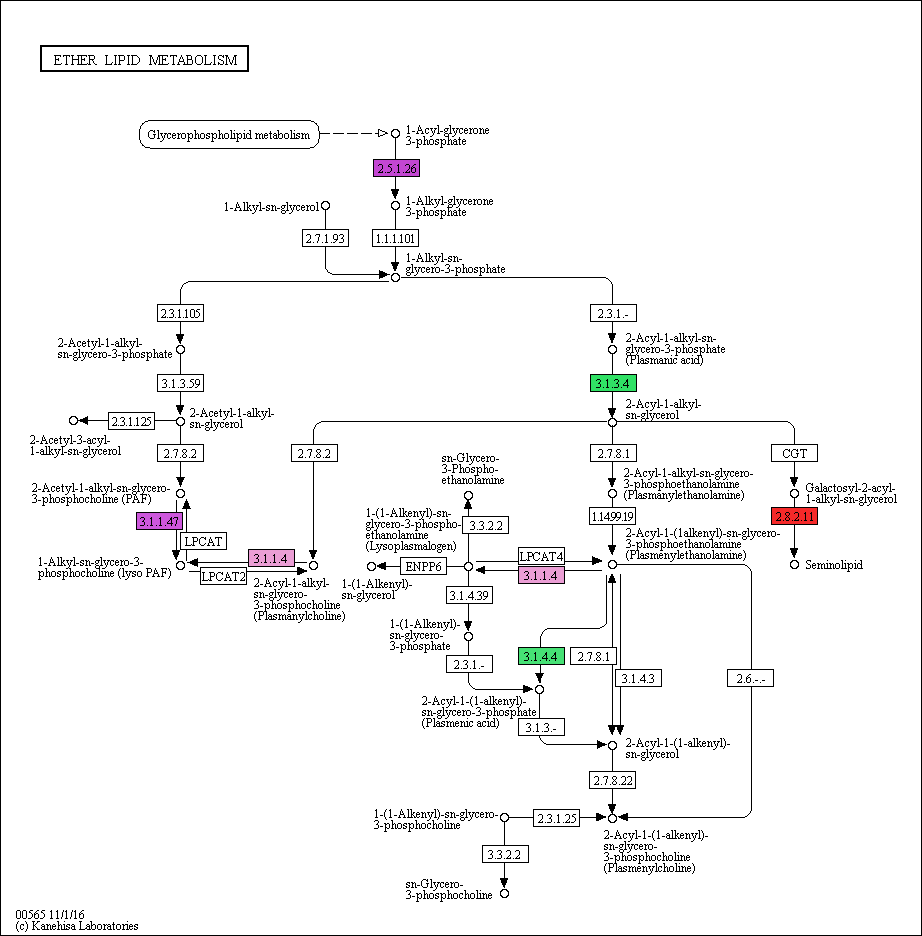

Supplement: Supplementary file 1 [file marinedrugs-16-00207-s001.zip › Supplementary Figures and Tables/Supplementary File 1 _ KEGG pathways/map00565 (Ether lipid metabolism) [6 enz found].png]

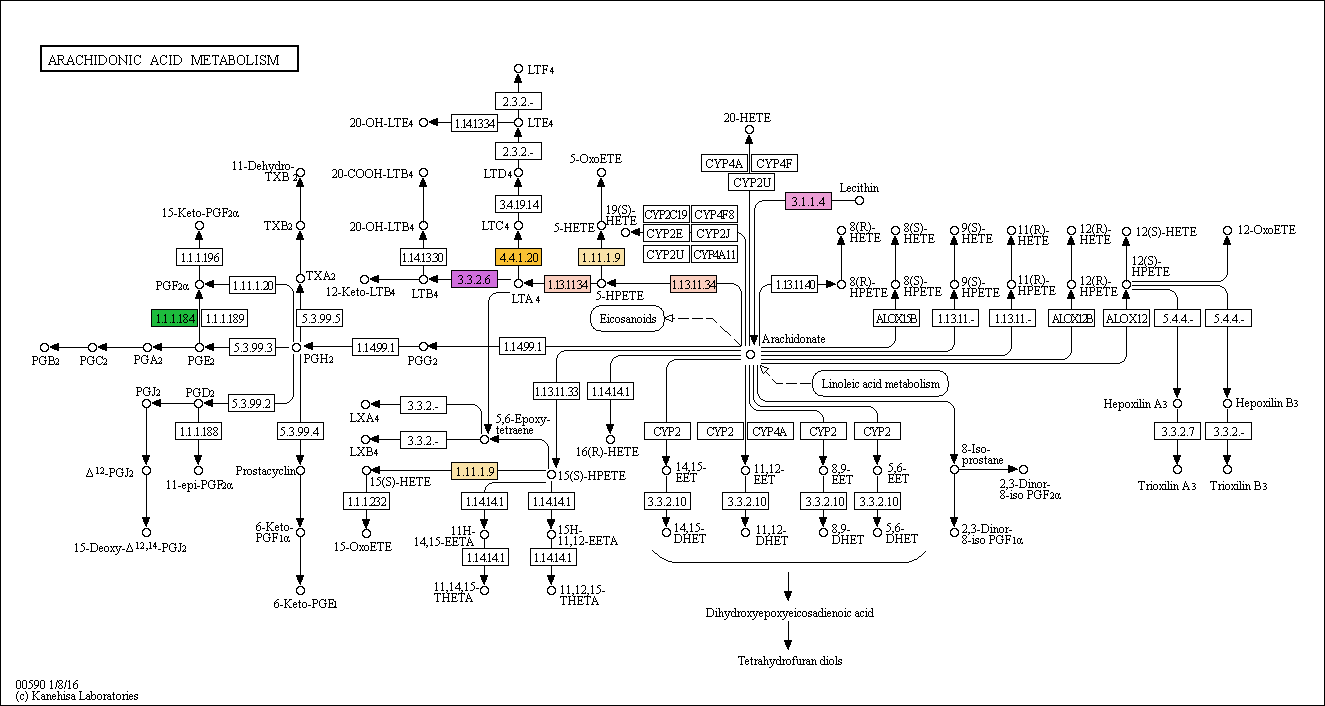

Supplement: Supplementary file 1 [file marinedrugs-16-00207-s001.zip › Supplementary Figures and Tables/Supplementary File 1 _ KEGG pathways/map00590 (Arachidonic acid metabolism) [6 enz found].png]

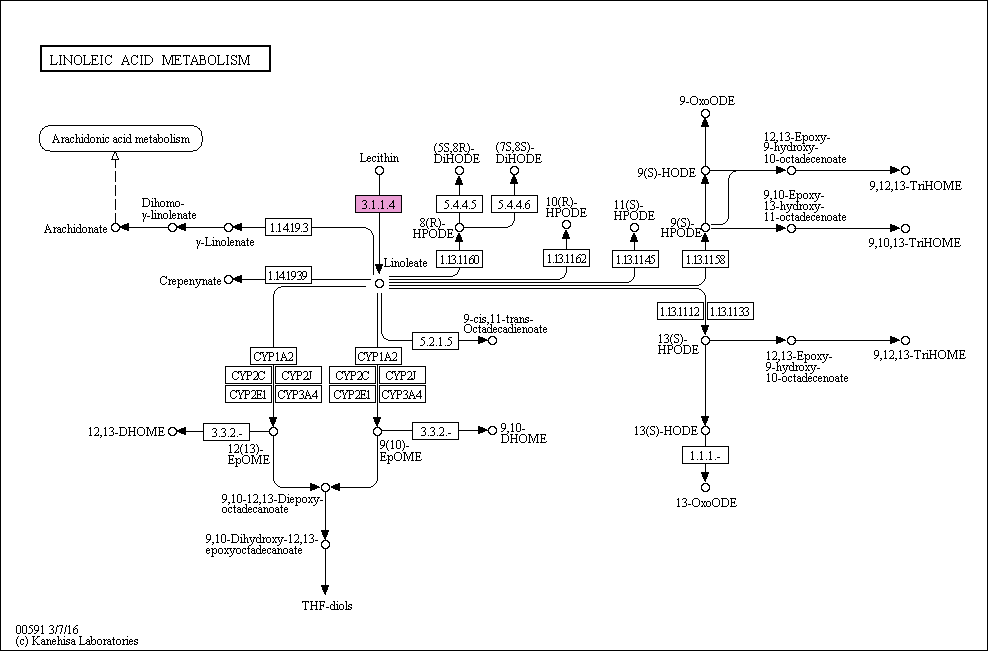

Supplement: Supplementary file 1 [file marinedrugs-16-00207-s001.zip › Supplementary Figures and Tables/Supplementary File 1 _ KEGG pathways/map00591 (Linoleic acid metabolism) [1 enz found].png]

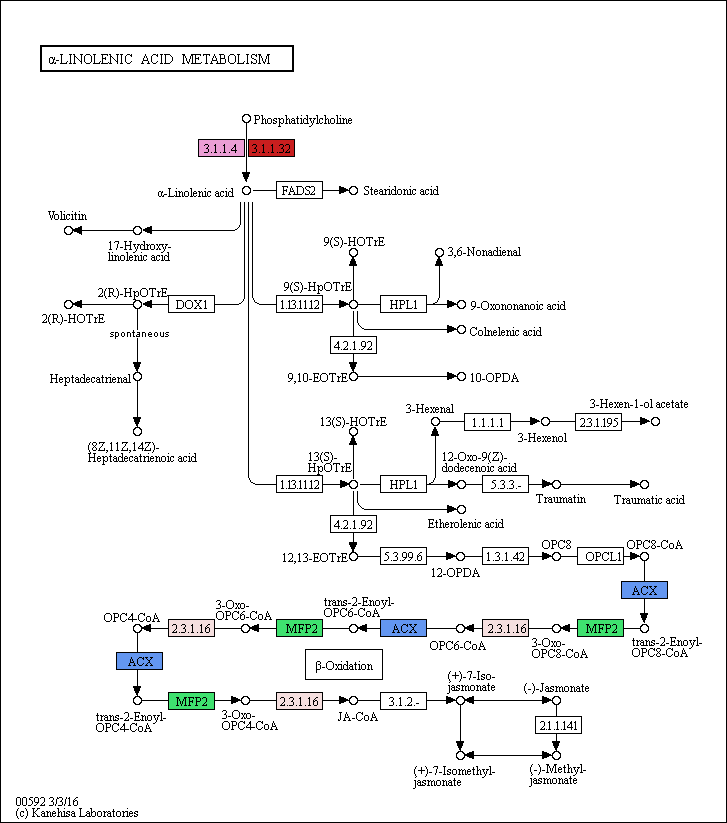

Supplement: Supplementary file 1 [file marinedrugs-16-00207-s001.zip › Supplementary Figures and Tables/Supplementary File 1 _ KEGG pathways/map00592 (Alpha-linolenic acid metabolism) [5 enz found].png]

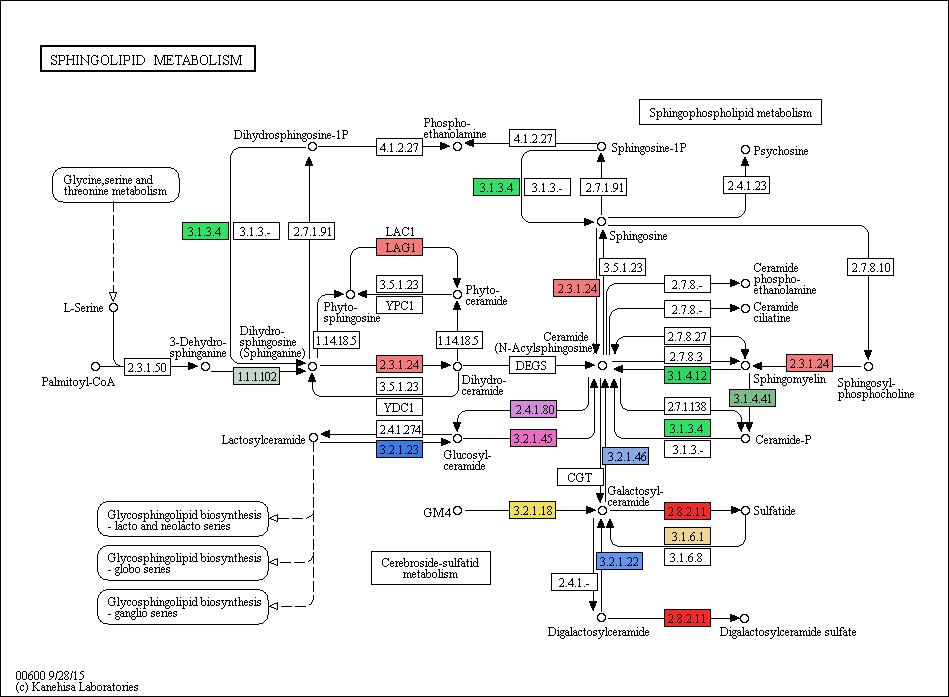

Supplement: Supplementary file 1 [file marinedrugs-16-00207-s001.zip › Supplementary Figures and Tables/Supplementary File 1 _ KEGG pathways/map00600 (Shingolipid metabolism) [13 enz found].png]

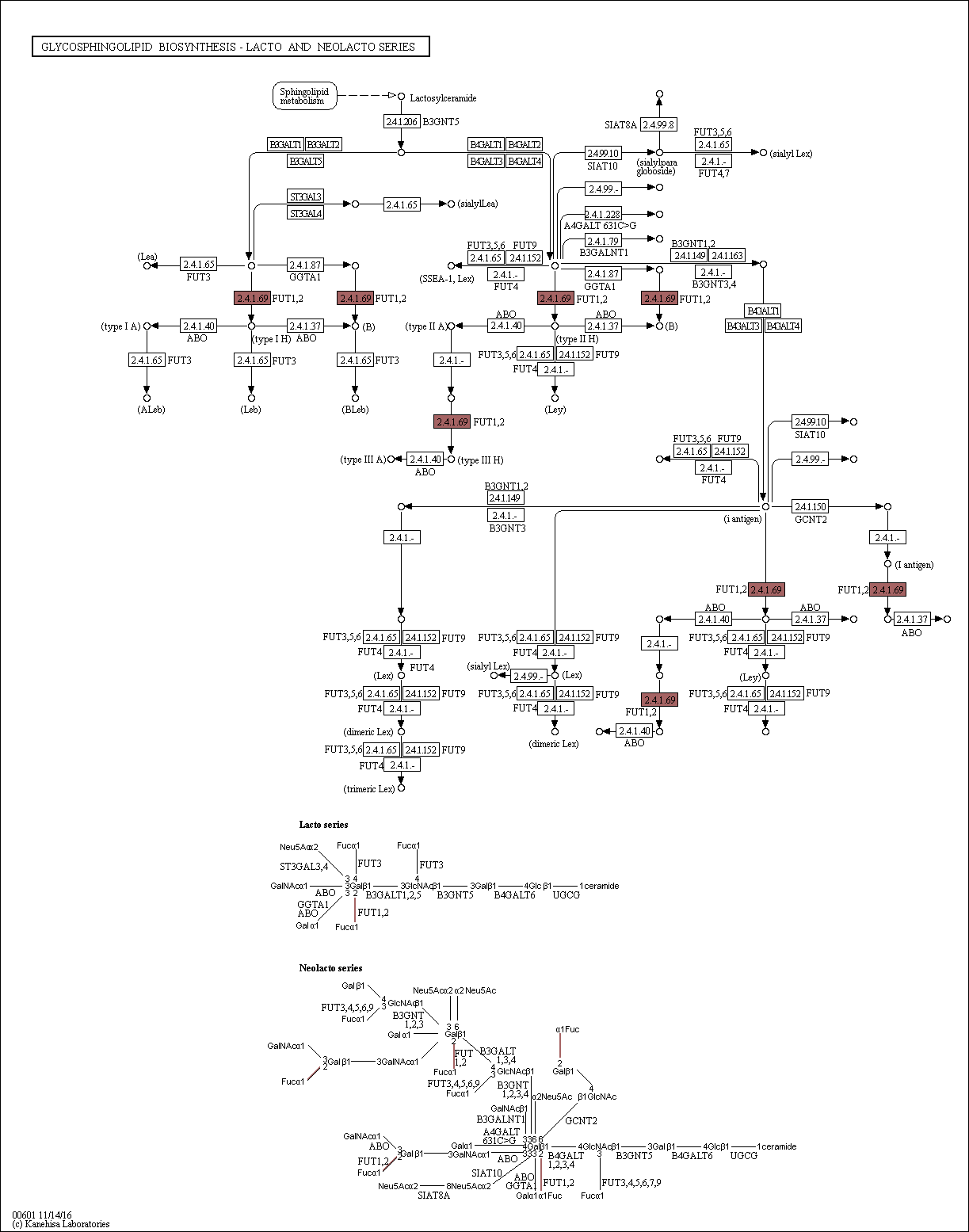

Supplement: Supplementary file 1 [file marinedrugs-16-00207-s001.zip › Supplementary Figures and Tables/Supplementary File 1 _ KEGG pathways/map00601 (Glycosphingolipid biosynthesis) [1 enz found].png]

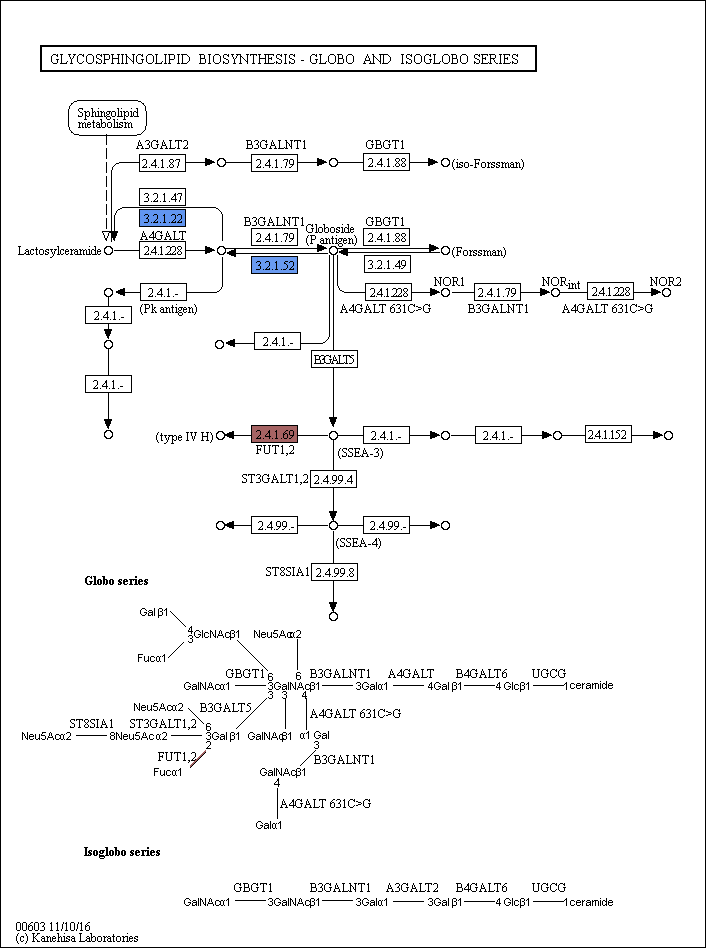

Supplement: Supplementary file 1 [file marinedrugs-16-00207-s001.zip › Supplementary Figures and Tables/Supplementary File 1 _ KEGG pathways/map00603 (Glycosphingolipid biosynthesis) [3 enz found].png]

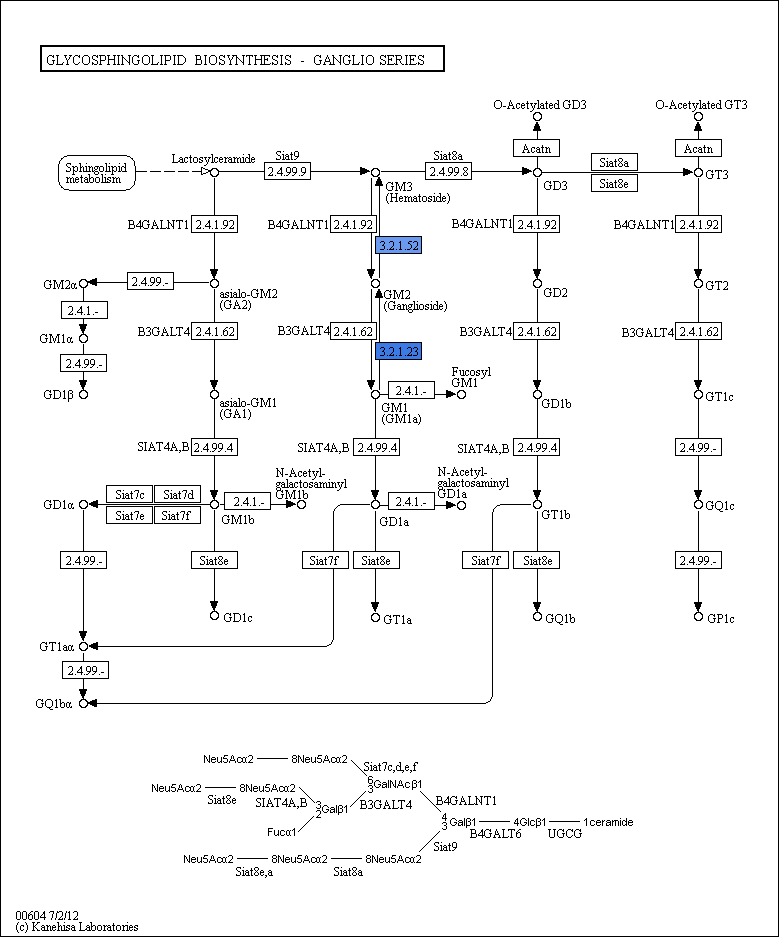

Supplement: Supplementary file 1 [file marinedrugs-16-00207-s001.zip › Supplementary Figures and Tables/Supplementary File 1 _ KEGG pathways/map00604 (Glycosphingolipid biosynthesis) [2 enz found].png]

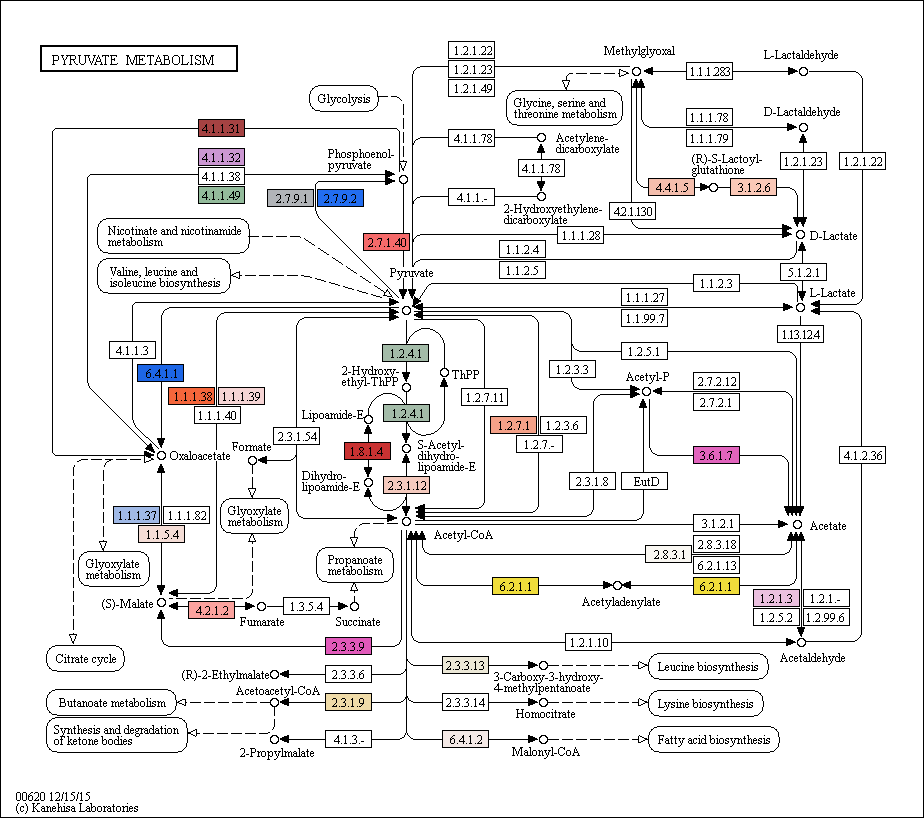

Supplement: Supplementary file 1 [file marinedrugs-16-00207-s001.zip › Supplementary Figures and Tables/Supplementary File 1 _ KEGG pathways/map00620 (Pyruvate metabolism) [26 enz found].png]

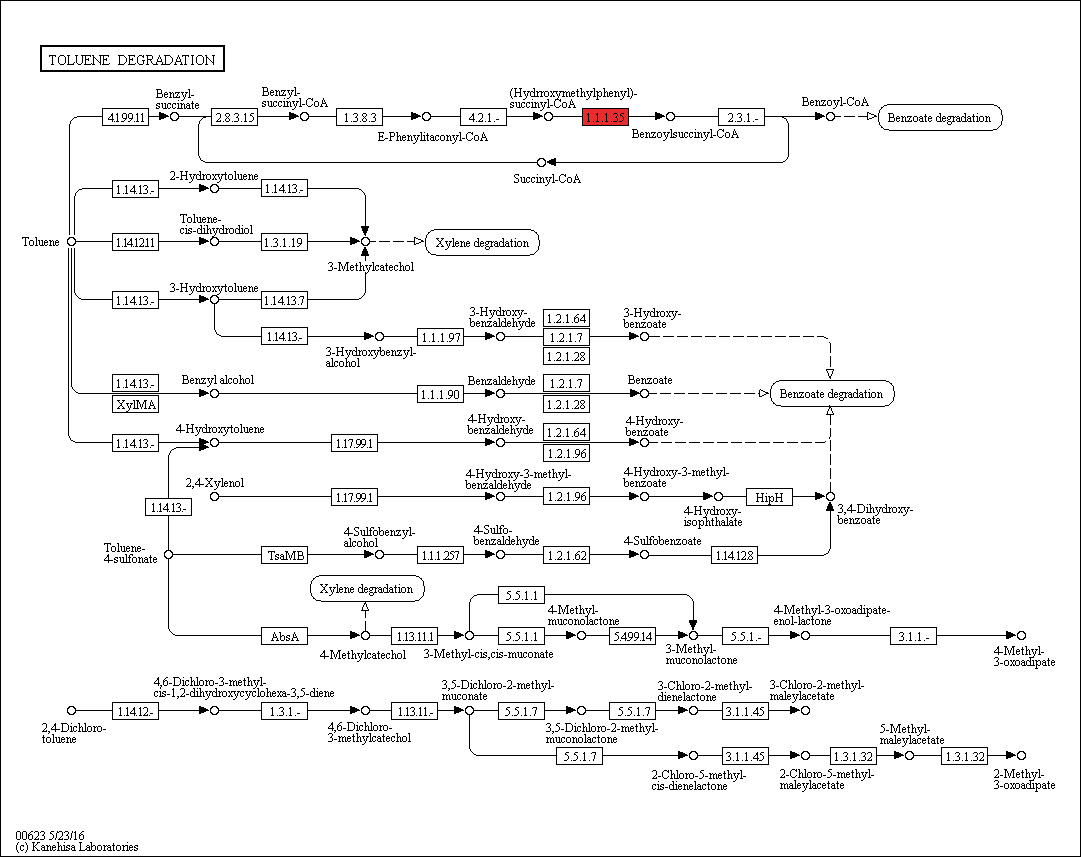

Supplement: Supplementary file 1 [file marinedrugs-16-00207-s001.zip › Supplementary Figures and Tables/Supplementary File 1 _ KEGG pathways/map00623 (Toluene degradation) [1 enz found].png]

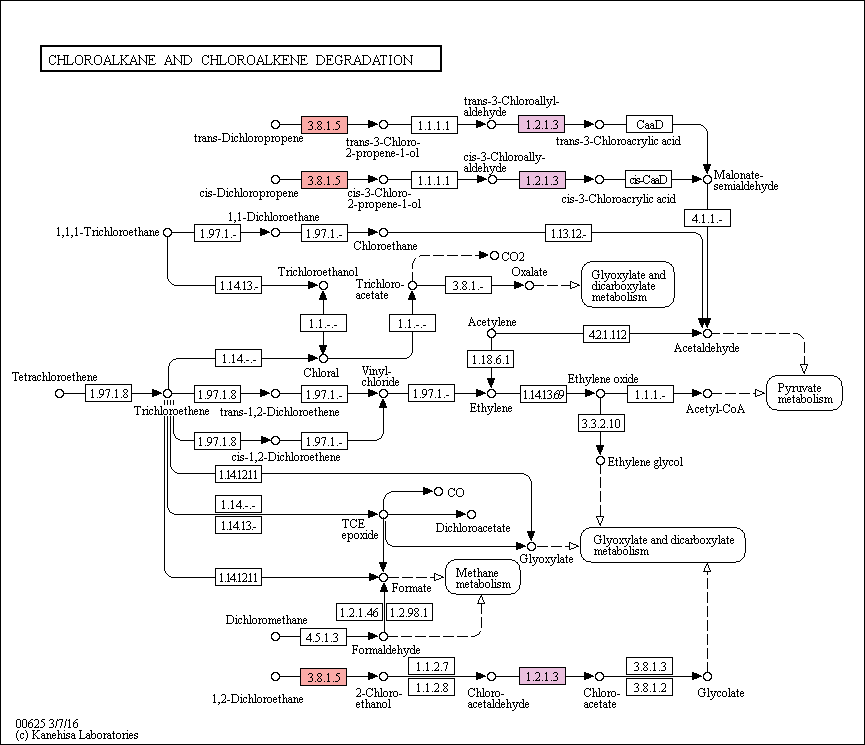

Supplement: Supplementary file 1 [file marinedrugs-16-00207-s001.zip › Supplementary Figures and Tables/Supplementary File 1 _ KEGG pathways/map00625 (Chloroalkane and chloroalkene degradation) [2 enz found].png]

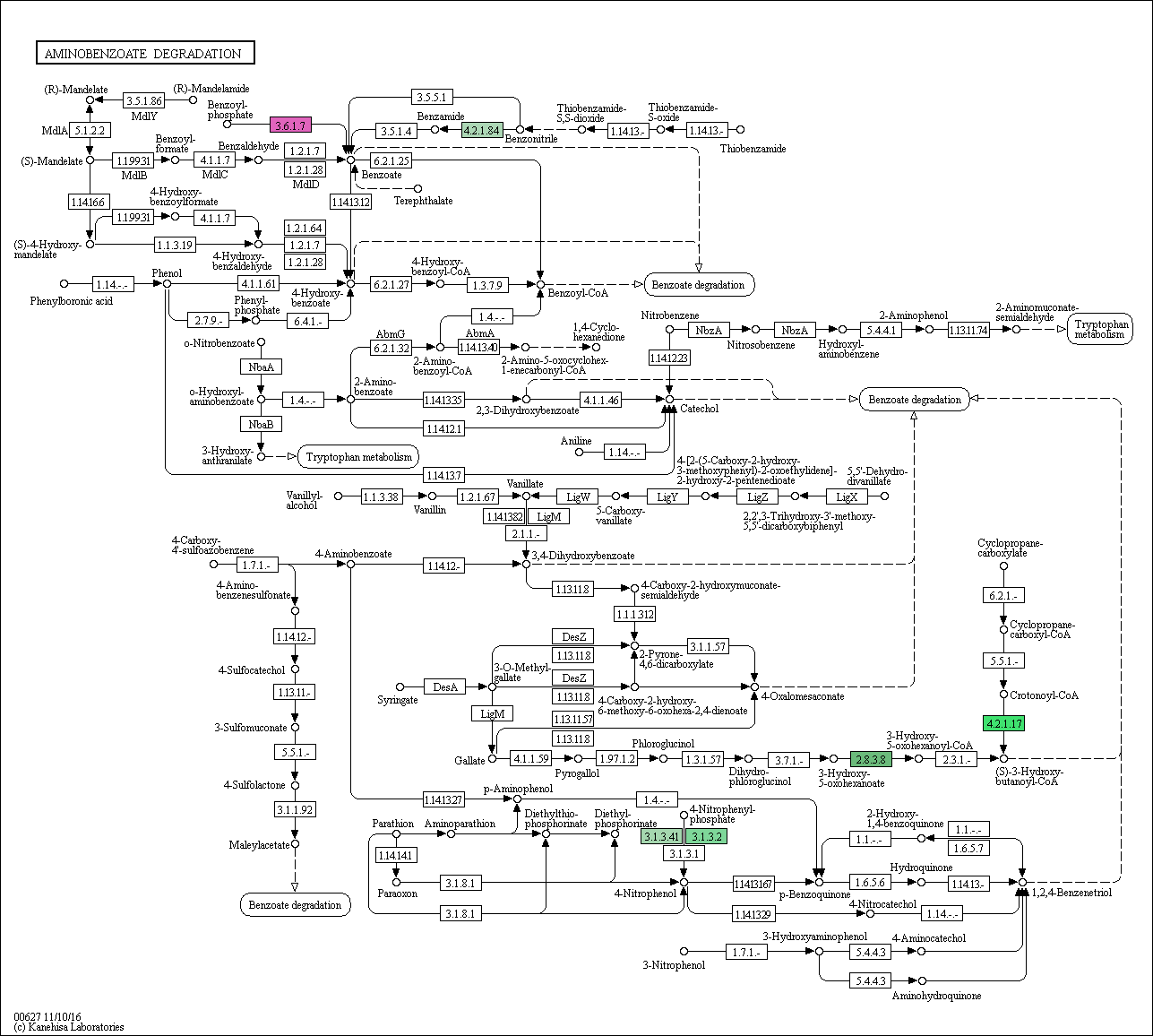

Supplement: Supplementary file 1 [file marinedrugs-16-00207-s001.zip › Supplementary Figures and Tables/Supplementary File 1 _ KEGG pathways/map00627 (Aminobenzoate degradation) [6 enz found].png]

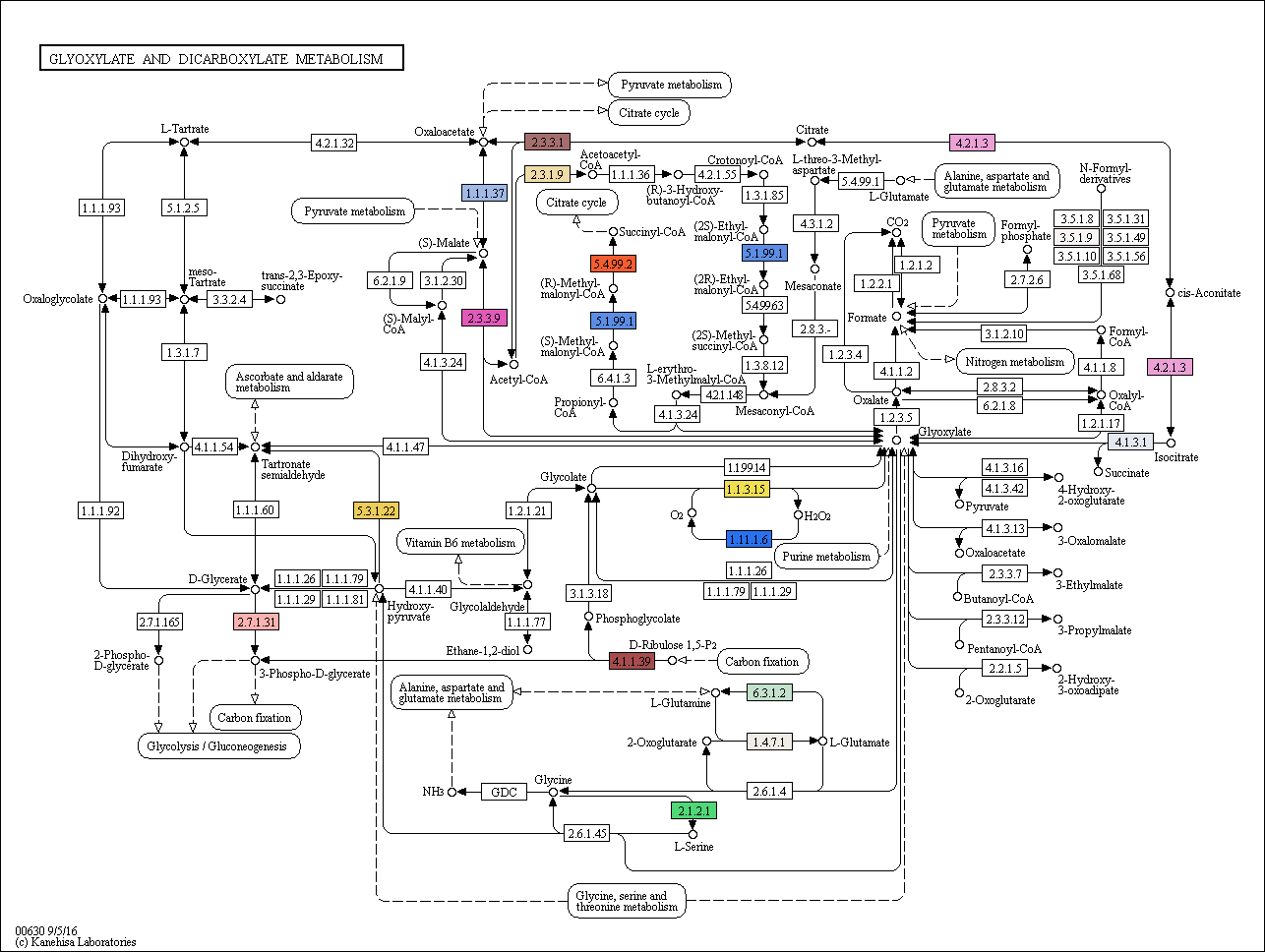

Supplement: Supplementary file 1 [file marinedrugs-16-00207-s001.zip › Supplementary Figures and Tables/Supplementary File 1 _ KEGG pathways/map00630 (Glyoxylate and dicarboxylate metabolism) [17 enz found].png]

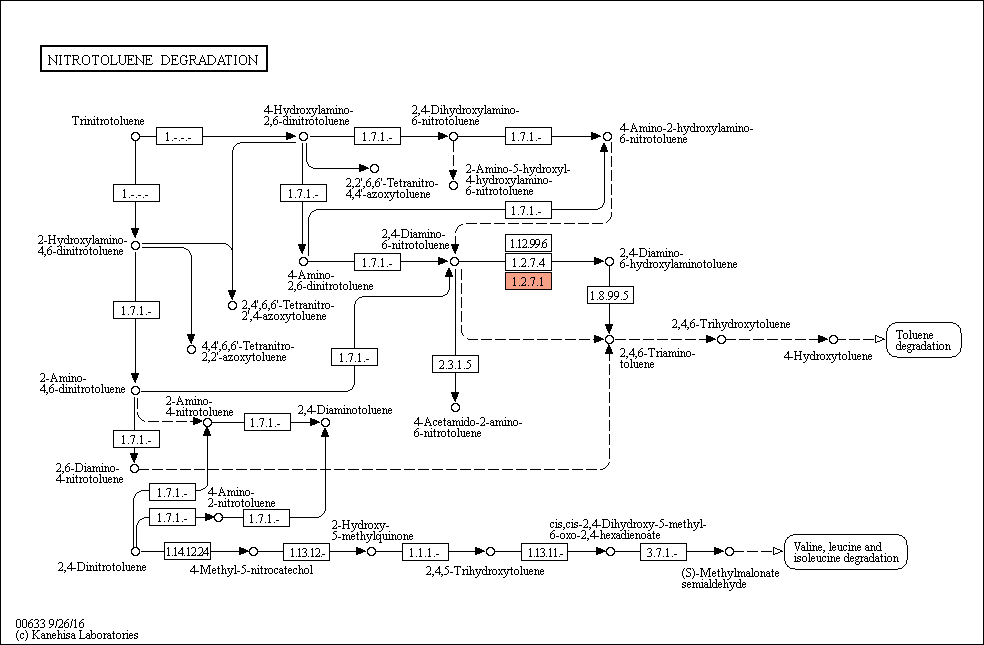

Supplement: Supplementary file 1 [file marinedrugs-16-00207-s001.zip › Supplementary Figures and Tables/Supplementary File 1 _ KEGG pathways/map00633 (Nitrotoluene degradation) [1 enz found].png]

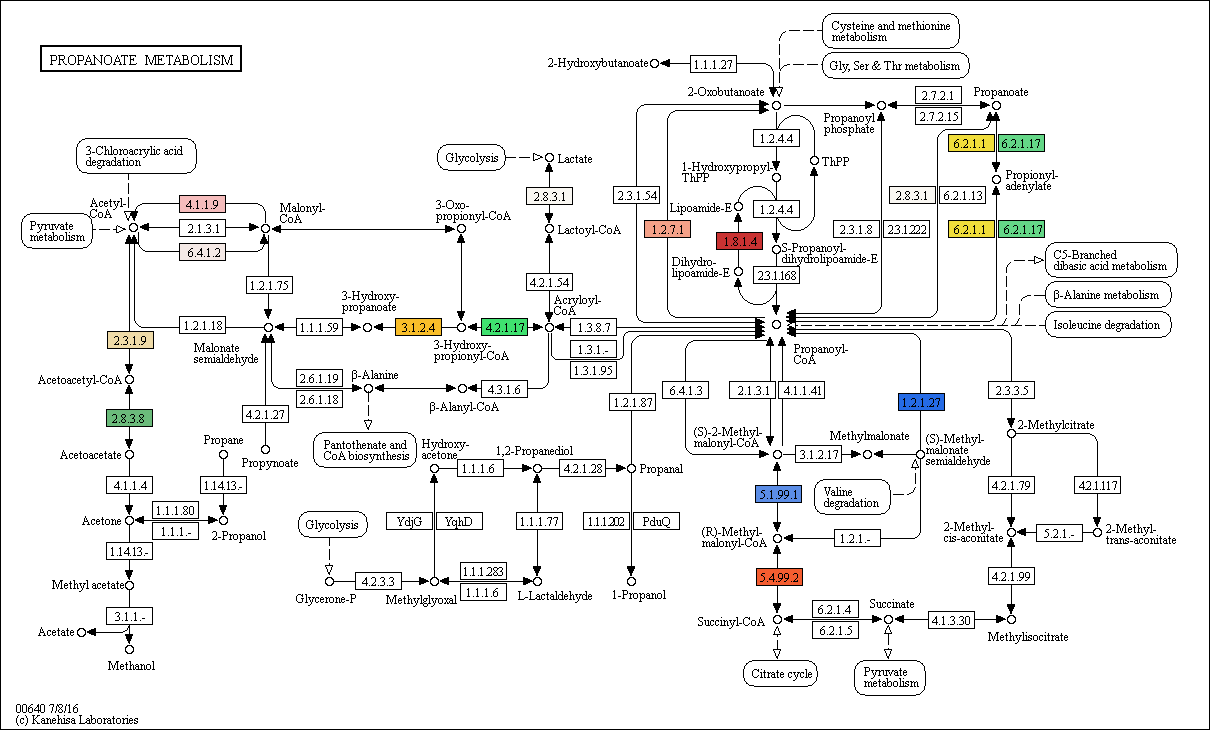

Supplement: Supplementary file 1 [file marinedrugs-16-00207-s001.zip › Supplementary Figures and Tables/Supplementary File 1 _ KEGG pathways/map00640 (Propanoate metabolism) [14 enz found].png]

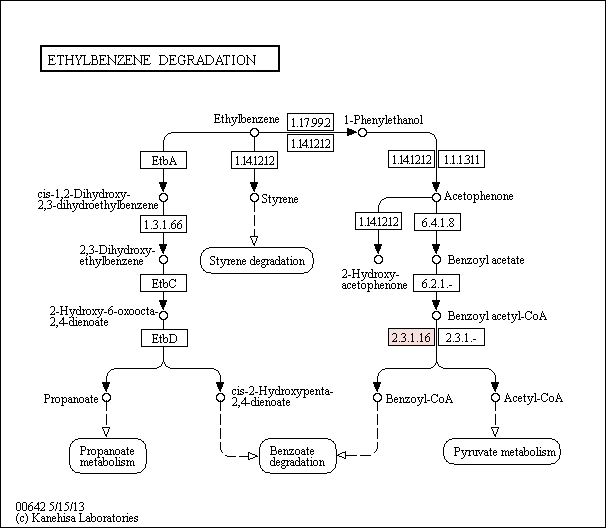

Supplement: Supplementary file 1 [file marinedrugs-16-00207-s001.zip › Supplementary Figures and Tables/Supplementary File 1 _ KEGG pathways/map00642 (Ethylbenzene degradation) [1 enz found].png]

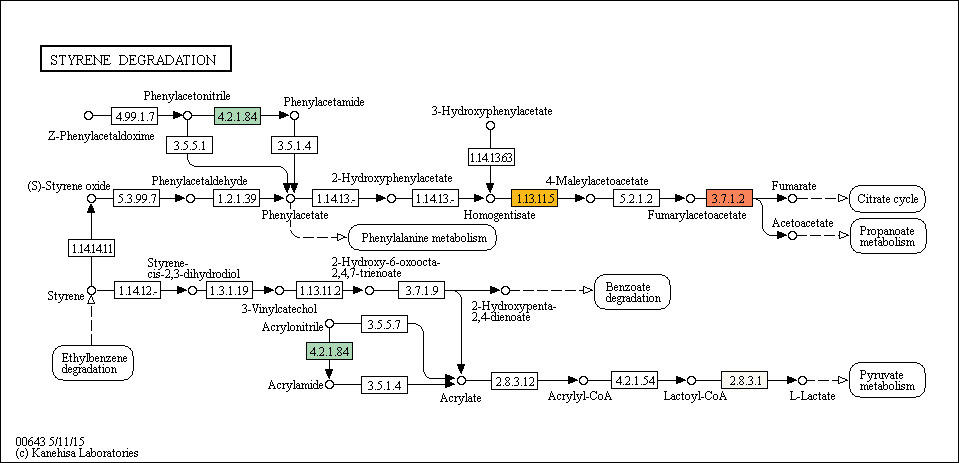

Supplement: Supplementary file 1 [file marinedrugs-16-00207-s001.zip › Supplementary Figures and Tables/Supplementary File 1 _ KEGG pathways/map00643 (Styrene degradation) [4 enz found].png]

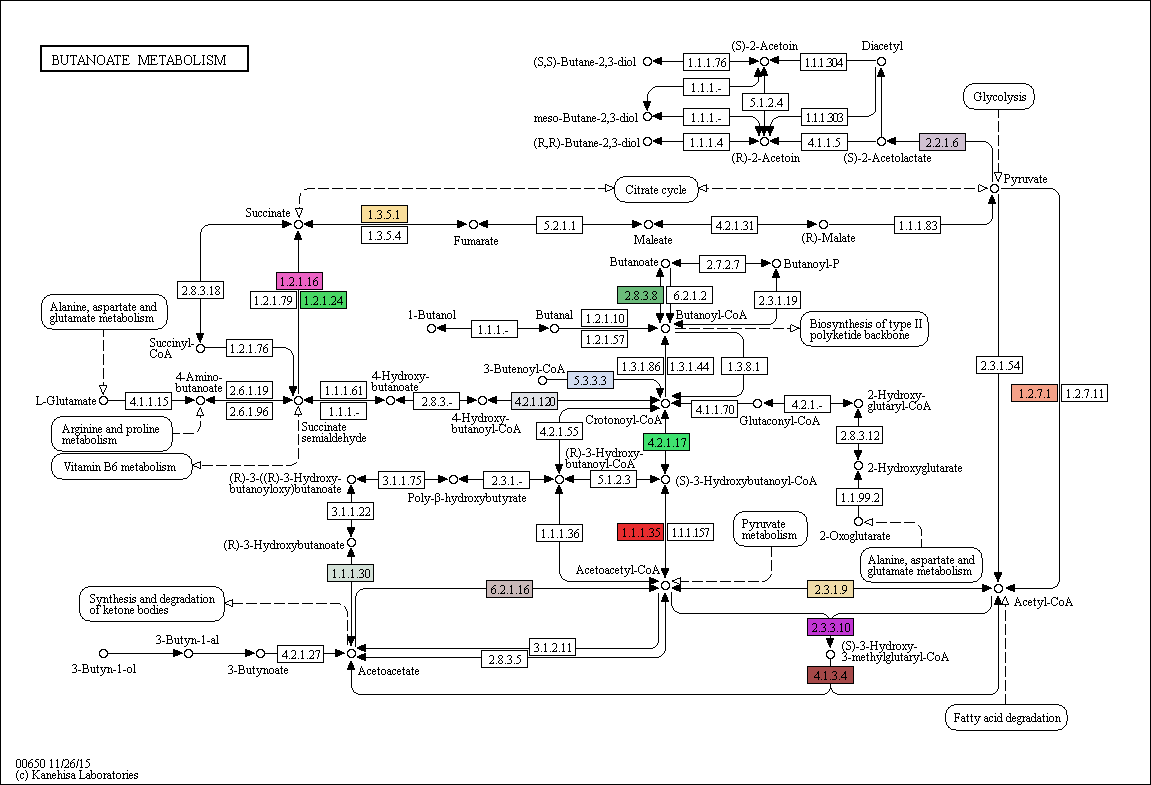

Supplement: Supplementary file 1 [file marinedrugs-16-00207-s001.zip › Supplementary Figures and Tables/Supplementary File 1 _ KEGG pathways/map00650 (Butanoate metabolism) [15 enz found].png]

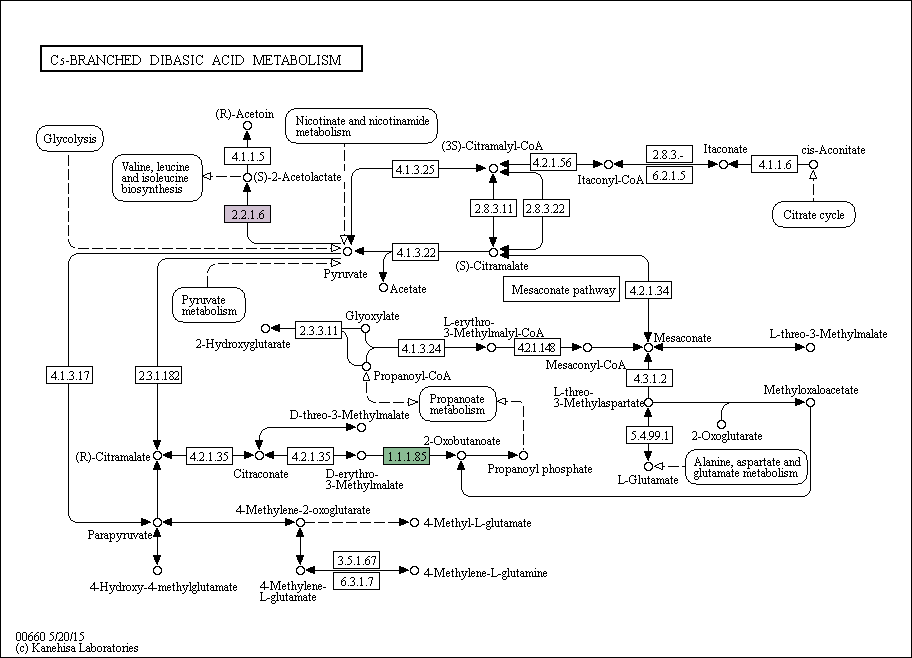

Supplement: Supplementary file 1 [file marinedrugs-16-00207-s001.zip › Supplementary Figures and Tables/Supplementary File 1 _ KEGG pathways/map00660 (C5-branched dibasic acid metabolism) [2 enz found].png]

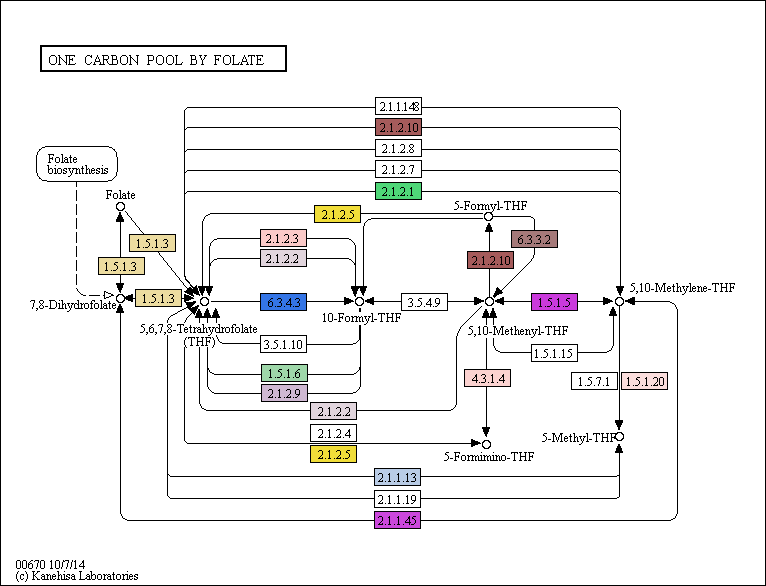

Supplement: Supplementary file 1 [file marinedrugs-16-00207-s001.zip › Supplementary Figures and Tables/Supplementary File 1 _ KEGG pathways/map00670 (One carbon pool by folate) [15 enz found].png]

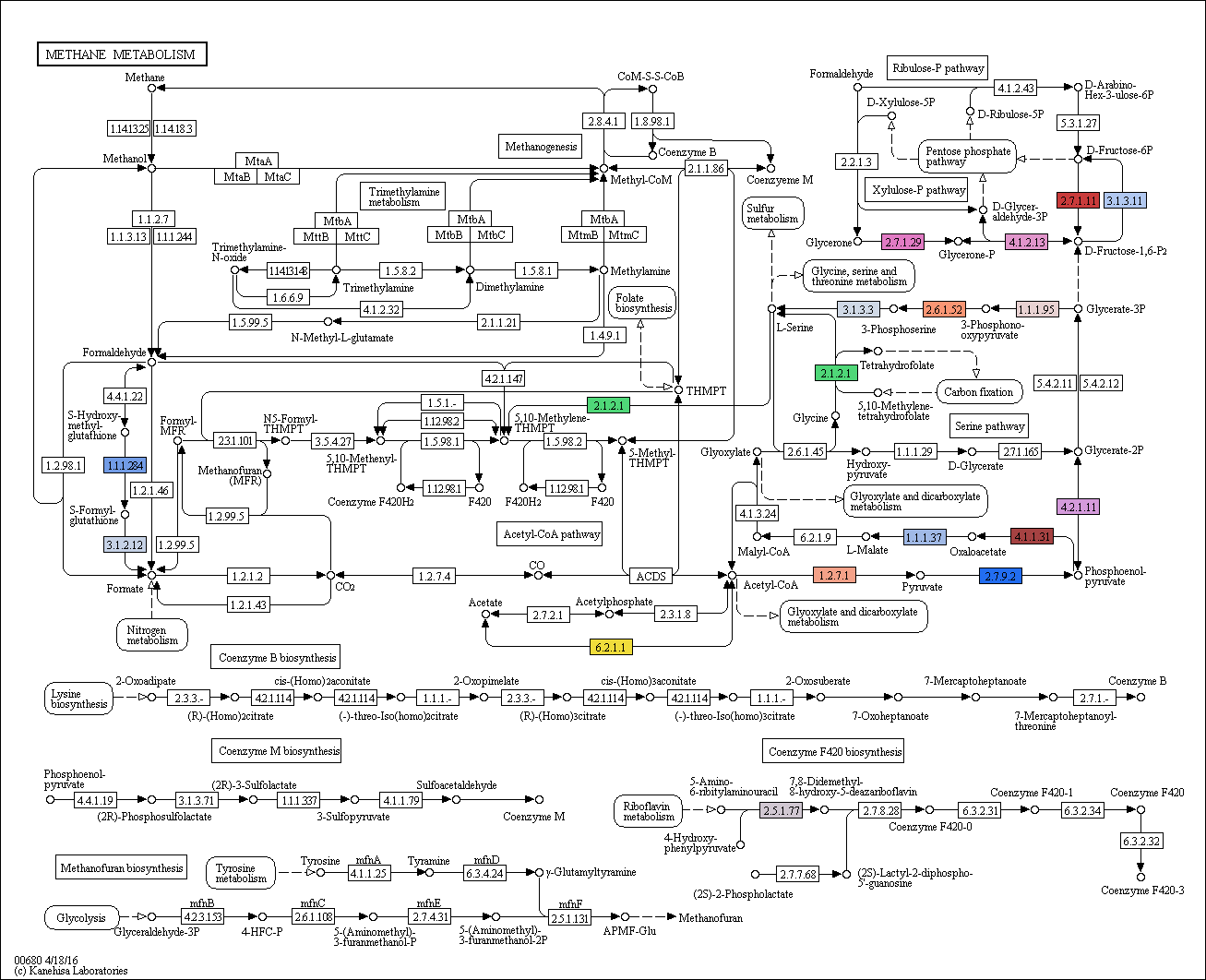

Supplement: Supplementary file 1 [file marinedrugs-16-00207-s001.zip › Supplementary Figures and Tables/Supplementary File 1 _ KEGG pathways/map00680 (Methane metabolism) [17 enz found].png]

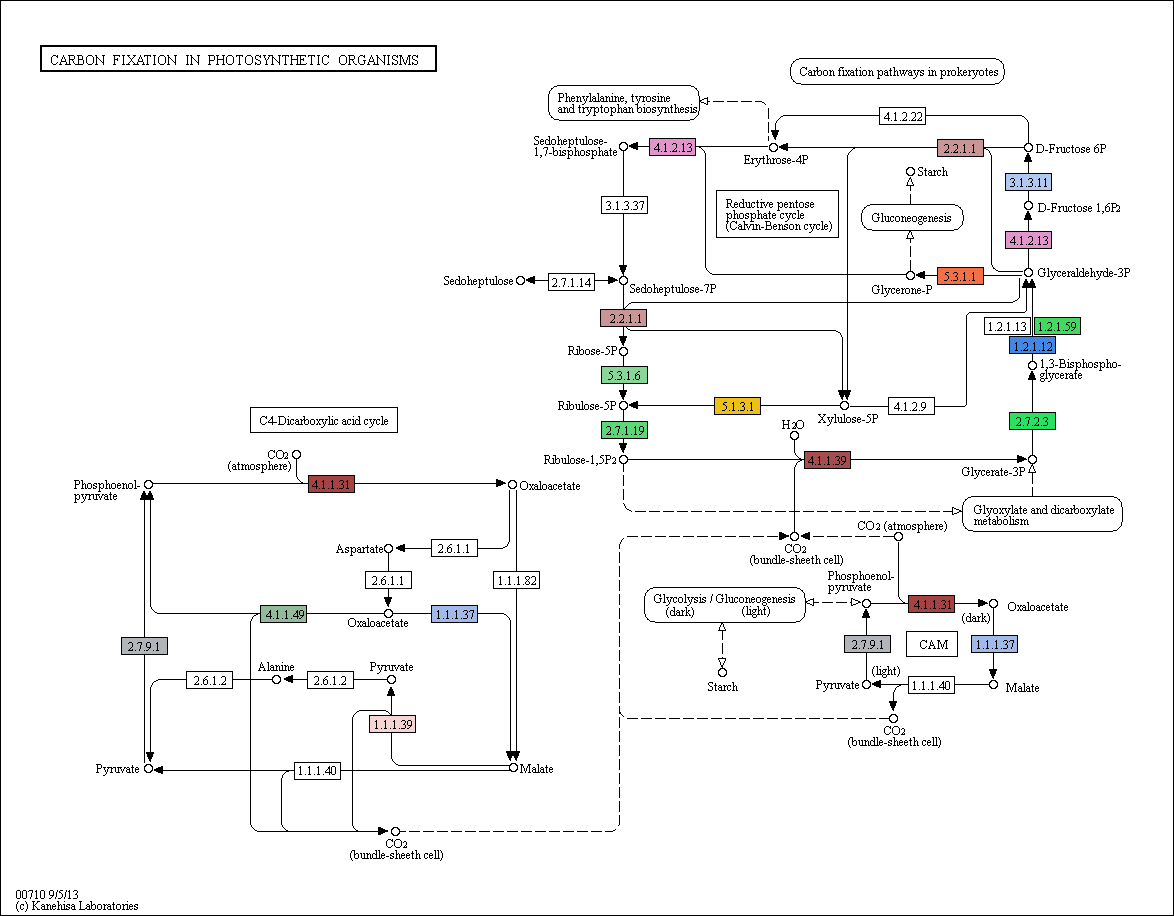

Supplement: Supplementary file 1 [file marinedrugs-16-00207-s001.zip › Supplementary Figures and Tables/Supplementary File 1 _ KEGG pathways/map00710 (Carbon fixation in photosynthetic organisms) [16 enz found].png]

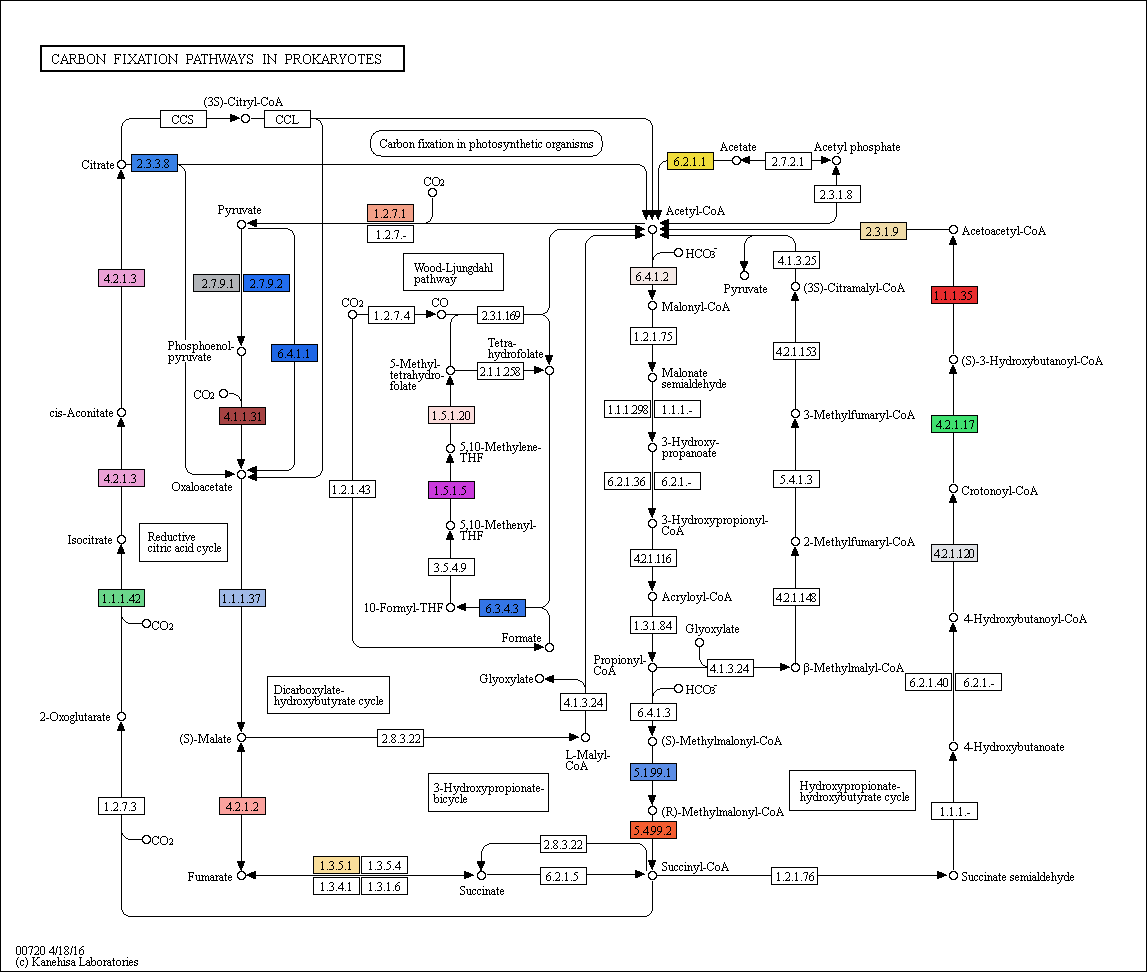

Supplement: Supplementary file 1 [file marinedrugs-16-00207-s001.zip › Supplementary Figures and Tables/Supplementary File 1 _ KEGG pathways/map00720 (Carbon fixation pathways in prokaryotes) [22 enz found].png]

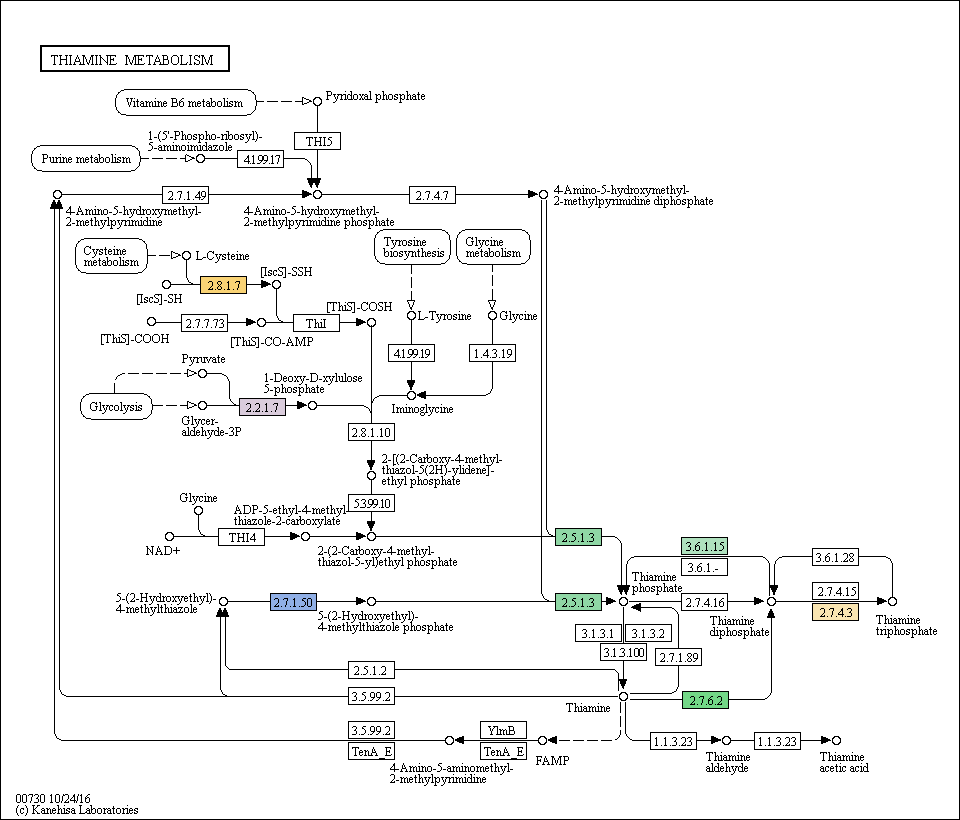

Supplement: Supplementary file 1 [file marinedrugs-16-00207-s001.zip › Supplementary Figures and Tables/Supplementary File 1 _ KEGG pathways/map00730 (Thiamine metabolism) [7 enz found].png]

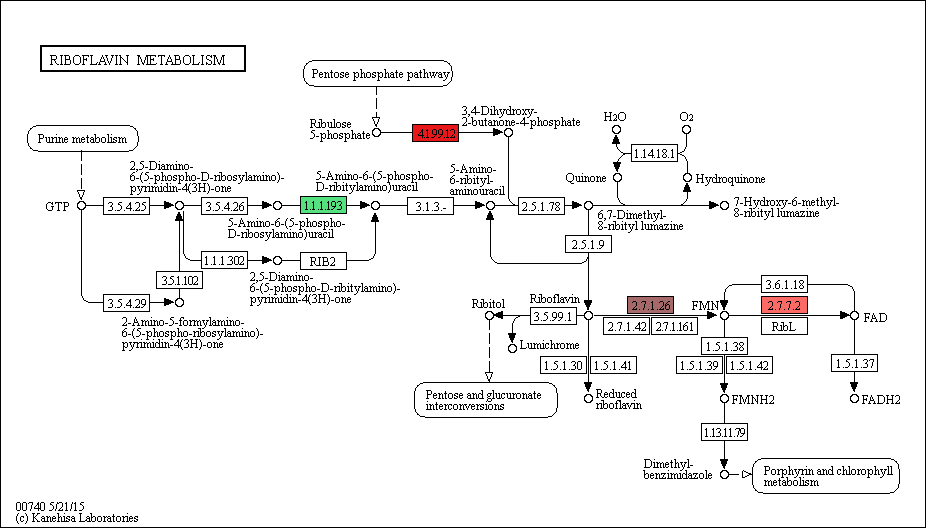

Supplement: Supplementary file 1 [file marinedrugs-16-00207-s001.zip › Supplementary Figures and Tables/Supplementary File 1 _ KEGG pathways/map00740 (Riboflavin metabolism) [4 enz found].png]

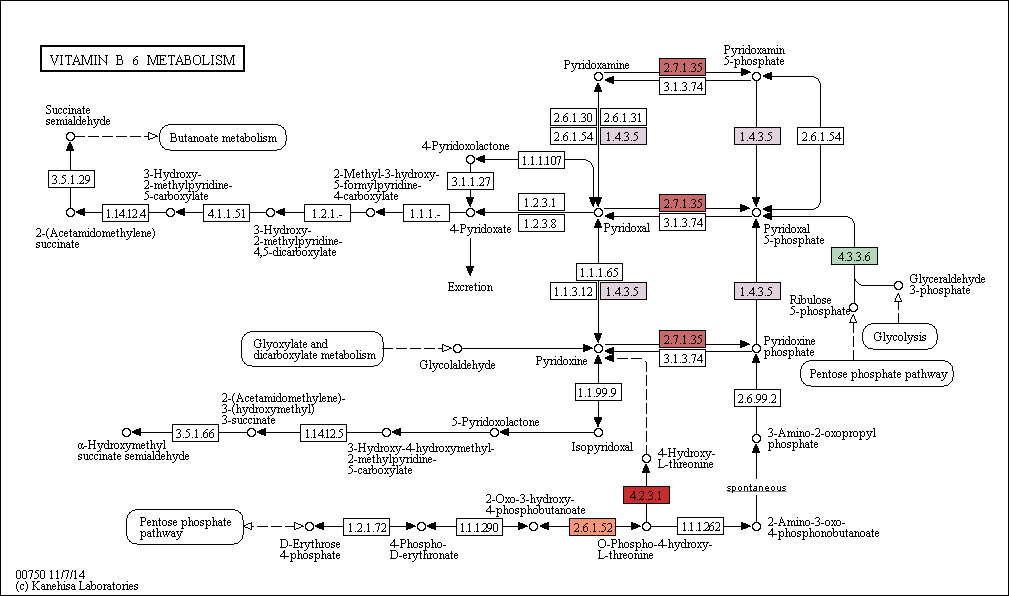

Supplement: Supplementary file 1 [file marinedrugs-16-00207-s001.zip › Supplementary Figures and Tables/Supplementary File 1 _ KEGG pathways/map00750 (Vitamin B6 metabolism) [5 enz found].png]

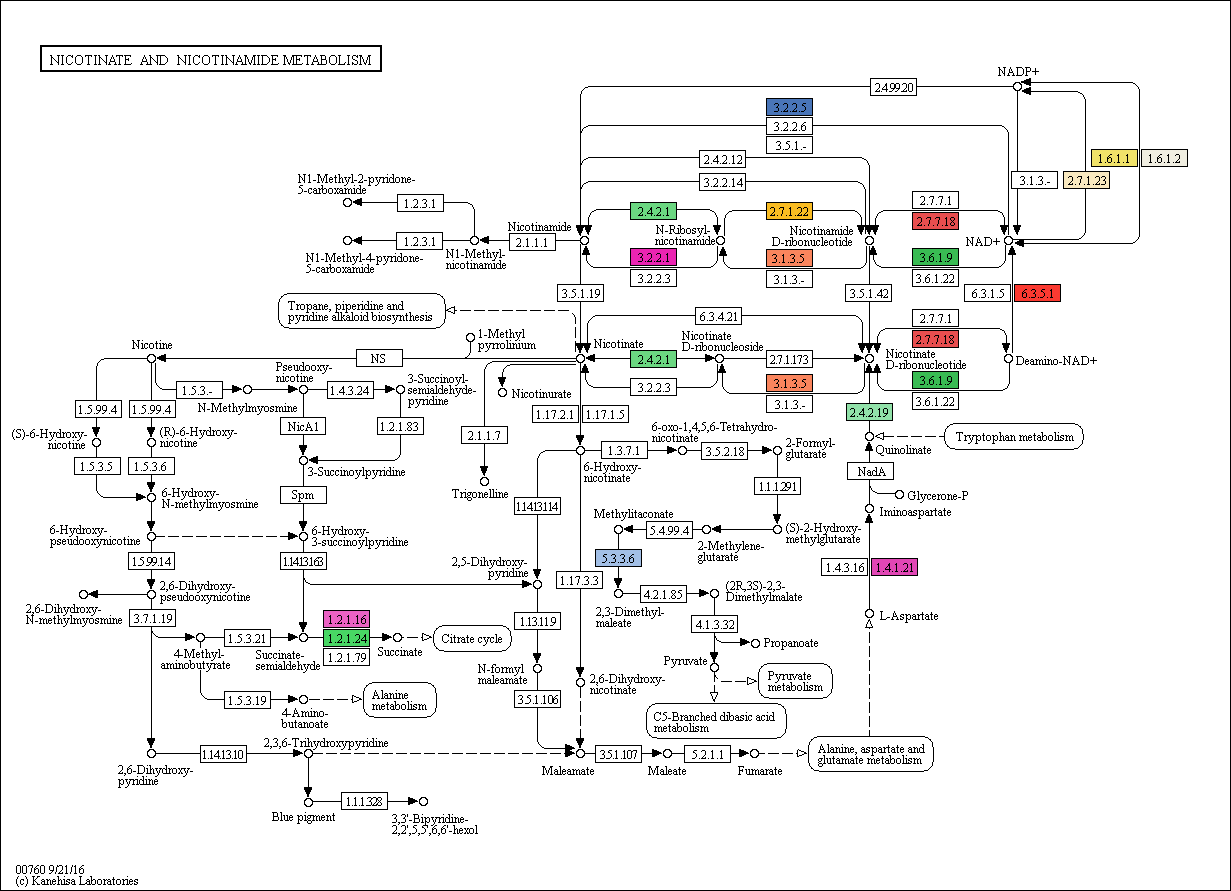

Supplement: Supplementary file 1 [file marinedrugs-16-00207-s001.zip › Supplementary Figures and Tables/Supplementary File 1 _ KEGG pathways/map00760 (Nicotinate and nicotinamide metabolism) [16 enz found].png]
